# Supplementary material for: In Silico Mass Spectrometric Fragmentation and Liquid Chromatography with Tandem Mass Spectrometry (LC-MS/MS) Betalainic Fingerprinting: Identification of Betalains in Red Pitaya
Source: Molecules. 2024 Nov 20;29(22):5485. doi: 10.3390/molecules29225485 (PMC11597464; doi:10.3390/molecules29225485)
Supplement: Supplementary file 1 [file molecules-29-05485-s001.zip › molecules-3250162-supplementary.pdf]

SUPPLEMENTARY MATERIAL

In Silico Mass Spectrometric Fragmentation and Liquid Chromatography with Tandem Mass Spectrometry (LC-MS/MS) Betalainic fingerprinting: Identification of Betalains in Red Pitaya

Jesús Alfredo Araujo-León[1], Ivonne Sánchez-del Pino[2], Ligia Guadalupe Brito-Argáez[1], Sergio R. Peraza-Sánchez[3], Rolffy Ortiz-Andrade[4], Víctor Aguilar-Hernández[1,*]

[1] Unidad de Biología Integrativa, Centro de Investigación Científica de Yucatán, A.C., Calle 43 No. 130 × 32 y 34, Col. Chuburná de Hidalgo, Mérida, Yucatán, México 97205.
[2] Unidad de Recursos Naturales, Centro de Investigación Científica de Yucatán, A.C., Calle 43 No. 130 × 32 y 34, Col. Chuburná de Hidalgo, Mérida, Yucatán, México 97205.
[3] Unidad de Biotecnología, Centro de Investigación Científica de Yucatán, A.C., Calle 43 No. 130 × 32 y 34. Col. Chuburná de Hidalgo, Mérida, Yucatán, México 97205.
[4] Facultad de Química, Universidad Autónoma de Yucatán, Calle 43 No. 613 × Calle 90, Col. Inalámbrica, Mérida, Yucatán, México 97069.
* Correspondence: victor.aguilar@cicy.mx

Table S1. *In silico* analysis of betalains, using MassFrontier (Thermo Scientific) software.

Betalains

Betacyanins **6**

Betanin-type **6**

Prebetanin 6
Isoprebetanin 7
Rivinianin 8
Isorivinianin 9
Betanin 10
Isobetanin 11
Betanidin 12
Phyllocactin 13
Isophyllocactin 14
Hylocerenin 15
Isohylocerenin 16
Lampranthin I 17
Isoampranthin I 18
Lampranthin II 19
Isolampranthin II 20
4'-*O*-malonyl-betanin 21
4'-*O*-malonyl-isobetanin 22
2-decarboxy-2,3-dehydro-betanin 23
17-decarboxy-betanin 24
2-decarboxy-2,3-dehydro-isobetanin 25
2,17-bidecarboxy-2,3-dehydro-isobetanin 26
17-decarboxy-isobetanin 27
17-decarboxy-phyllocactin 28
15-decarboxy-betanin 29
17-decarboxy-hylocerenin 30
2-decarboxy-betanin 31
17-decarboxy-isophyllocactin 32
17-decarboxy-isohylocerenin 33
2-decarboxy-phyllocactin 35
2-decarboxy-isophyllocactin 36
Neobetanin 37
17-decarboxy-neobetanin 38
2-decarboxy-neobetanin 39
2-decarboxy-2,3-dehydro-neobetanin 40
Neophyllocactin 41
2,15,17-tridecarboxy-neobetanin 42
17-decarboxy-neophyllocactin 43
Neohylocerenin 44
17-decarboxy-neohylocerenin 45
2,17-bidecarboxy-2,3-dehydro-neobetanin 46
2-decarboxy-neohylocerenin 47
2-decarboxy-neophyllocactin 48

Neolampranthin II 49
Dehydrogenated tridecarboxy-neobetanin 50

Amaranthin-type **51**
Amaranthin 51
Isoamaranthin 53
Iresinin I 55
Isoiresinin I 56
Celosianin I 57
Isocelosianin I 59
Celosianin II 61
Isocelosianin II 62
Sinapoyl-amaranthin 63
Sinapoyl-isoamaranthin 64
6´-*O*-malonyl-amaranthin 65
6´-*O*-malonyl-isoamaranthin 66
4´-*O*-malonyl-amaranthin 67
4´-*O*-malonyl-isoamaranthin 68
17-decarboxy-amaranthin 69
15-decarboxy-amaranthin 70
2-decarboxy-amaranthin 71
Neoamaranthin 72

Melocactin-type **73**
Melocactin 73
Isomelocactin 74
Bougainvillein-r III 75
Isobougainvillein-r III 76
Feruloyl-bougainvillein-r I 77
Feruloyl-isobougainvillein-r I 79
Mammillarinin 81
Isomammillarinin 82
4'-*O*-Malonyl-bougainvillein-r I 83
4'-*O*-Malonyl-isobougainvillein-r I 84
2-decarboxy-mammillarinin 85
17-decarboxy-mammillarinin 86
17-decarboxy-melocactin 87

Apiocactin-type **88**
Apiocactin 88
Isoapiocactin 90
2'-*O*-apiosyl-phyllocactin 91
2'-*O*-apiosyl-isophyllocactin 92
5''-*O*-*E*-Feruloyl-apiocactin 93
5''-*O*-*E*-Feruloyl-isoapiocactin 94
5''-*O*-*E*-Sinapoyl-apiocactin 95
5''-*O*-*E*-Sinapoyl-isoapiocactin 96
2'-*O*-β-(5''-*O*-*E*-Feruloyl)-apiosyl-phyllocactin 97
2'-*O*-β-(5''-*O*-*E*-Feruloyl)-apiosyl-isophyllocactin 99
17-decarboxy-2'-*O*-apiosyl-phyllocactin 101
17-decarboxy-2'-*O*-apiosyl-isophyllocactin 102

Gomphrenin-type **103**
Gomphrenin I 103

Isogomphrenin I 104
Gomphrenin II 105
Isogomphrenin-II 107
Gomphrenin-III 108
Isogomphrenin-III 109
Gomphrenin-IV 110
Isogomphrenin-IV 111
2-decarboxy-2,3-dehydro-gomphrenin 112
Neogomphrenin 113
Neogomphrenin III 114
Neogomphrenin IV 115

Glabranin-type 116
Glabranin 116
Isoglabranin 117
Coumglabranin 118
Isocoumglabranin 119
Neocoumglabranin 120
6''-*O*-Rhamnosy-glabranin 121
6''-*O*-Rhamnosy-isoglabranin 122
Cafglabranin 123
Isocafglabranin 124
Bicoumglabranin 125
2''-*O*-[(6´´-*O-E*-4-coumaroyl)]-glucosyl-cafglabranin 126
2''-*O*-glucosyl-bicoumglabranin 127
2''-*O*-[(6''-*O-E*-4-coumaroyl)]-sophorosyl-cafglabranin 128

Betaxanthins 129

Positively charged-type 129
Histamine-bx 129
Histidine-bx 130
Arginine-bx 131
Lysine-bx 132

Polar uncharged-type 133
Glutamine-bx 133
Asparagine-bx 134
Threonine-bx 135
Serine-bx 136

Negatively charged-type 137
Aspartic acid-bx 137
Glutamic acid-bx 138

Hydrophobic-type 139
Alanine-bx 139
Valine-bx 140
Isoleucine-bx 141
Leucine-bx 142
Methionine-bx 143
Phenylalanine-bx 144
Tyrosine-bx 145
Tryptophan-bx 146
Glycine-bx 147

Cysteine-bx 148
Proline-bx 149

Special cases **150**
γ-aminobutyric acid-bx 150
Dopamine-bx 151
L-DOPA-bx 152
Tyramine-bx 153
3-methoxy-tyramine-bx 154
5-hydroxynorvaline-bx 155
Methionine sulfoxide-bx 156

Betacyanins
Betanin-type
Prebetanin

**Chemical structure**	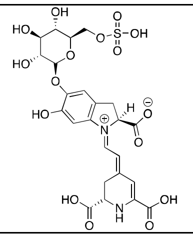
**Name:**	Prebetanin
**Chemical Formula**	$C_{24}H_{26}N_2O_{16}S$
**Molecular weight**	630.53
**Monoisotopic mass**	630.1003
***m/z* [M+H]**	631.1076
**Theorical fragments (*m/z*)**	631.11 630.1 629.09 628.08 615.11 614.1 613.1 612.09 611.08 603.11 602.1 601.1 599.12 598.11 597.1 596.09 595.09 594.08 593.07 591.11 589.1 588.09 587.12 586.11 585.1 584.09 583.09 581.11 580.1 579.09 578.08 577.08 575.06 573.1 571.12 570.12 569.11 568.1 567.09 566.08 565.08 563.1 561.1 559.09 558.08 557.11 556.1 555.09 553.11 552.1 551.1 550.09 549.08 545.11 543.09 542.08 541.08 540.07 539.1 538.09 537.08 533.14 532.13 531.12 527.1 525.08 523.07 517.15 516.14 515.13 514.12 513.11 509.09 507.07 505.15 504.14 503.13 501.15 499.13 498.13 497.12 495.1 493.15 491.13 489.15 488.14 487.13 486.13 485.12 483.14 481.12 479.11 475.13 473.16 471.14 470.13 469.12 463.13 462.07 461.12 460.11 459.14 458.13 457.12 449.06 448.05 447.14 446.08 445.12 444.06 443.11 438.07 437.06 436.05 435.05 432.06 431.05 429.13 427.11 425.1 423.06 422.05 421.04 420.06 419.05 418.04 417.04 410.08 408.06 405.05 404.04 403.03 402.05 400.03 393.05 392.06 391.06 390.05 389.1 388.09 387.08 386.03 380.06 379.03 378.05 377.05 376.05 375.12 374.05 373.1 372.1 371.09 370.08 369.07 368.06 366.05 365.05 364.1 361.1 360.05 359.12 357.11 356.1 355.09 354.08 353.05 351.04 350.03 349.1 348.05 347.04 345.11 344.1 343.13 342.08 341.11 340.1 339.1 338.09 337.08 335.04 334.04 333.03 332.02 331.09 330.04 329.11 327.1 326.09 325.08 324.08 323.08 322.09 320.08 319.09 317.08 316.07 315.1 314.09 313.08 307.08 306.07 303.1 301.08 300.07 299.07 298.06 297.09 294.1 287.1 285.09 284.08 283.07 282.06 281.08 280.07 279.09 271.11 269.09 268.08 267.06 265.08 264.07 263.07 262.06 259.01 255.09 253.08 252.06 251.07 250.08 249.05 247.07 245.06 243.02 237.09 235.07 233.06 231.04 225.01 223.07 222.06 220.06 211.07 209.06 208.05 207.08 205.06 196.06 195.05 194.04 193.04 192.03 191.05 182.08 181.05 180.04 179.03 178.05 177.04 176.03 167.07 166.09 165.05 164.05 163.04 150.05 147.04 146.04 145.05 135.04 134.04 130.04 124.04 123.04 119.05 111.04 110.06 109.03 108.02 107.05 106.04
**References**	Wyler, H.; Rösler, H.; Mercier, M.; Dreiding, A.S. Präbetanin, ein Schwefelsäure-halbester des Betanins. Ein Beitrag zur Kenntnis der Betacyane. *Helvetica Chimica Acta* **1967**, 50, 545–561.<br>Schliemann, W.; Joy, R.W.; Komamine, A.; Metzger, J.W.; Nimtz, M.; Wray, V.; Strack, D. Betacyanins from plants and cell cultures of Phytolacca americana. *Phytochemistry* **1996**, 42, 1039–1046.<br>Slimen, I.B.; Najar, T.; Abderrabba, M. Chemical and Antioxidant Properties of Betalains. *J. Agric. Food Chem.* **2017**, 65, 675–689.<br>Kumorkiewicz-Jamro, A.; Świergosz, T.; Sutor, K.; Spórna-Kucab, A.; Wybraniec, S. Multi-colored shades of betalains: Recent advances in betacyanin chemistry. *Nat. Prod. Rep.* **2021**, 38, 2315–2346.

Isoprebetanin

**Chemical structure**	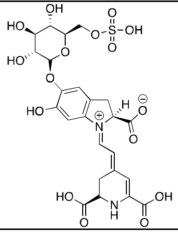
**Name:**	Isoprebetanin
**Chemical Formula**	$C_{24}H_{26}N_2O_{16}S$
**Molecular weight**	630.53
**Monoisotopic mass**	630.1003
***m/z* [M+H]**	631.1076
**Theorical fragments (*m/z*)**	631.11 630.1 629.09 628.08 615.11 614.1 613.1 612.09 611.08 603.11<br>602.1 601.1 599.12 598.11 597.1 596.09 595.09 594.08 593.07<br>591.11 589.1 588.09 587.12 586.11 585.1 584.09 583.09 581.11<br>580.1 579.09 578.08 577.08 575.06 573.1 571.12 570.12 569.11<br>568.1 567.09 566.08 565.08 563.1 561.1 559.09 558.08 557.11<br>556.1 555.09 553.11 552.1 551.1 550.09 549.08 545.11 543.09<br>542.08 541.08 540.07 539.1 538.09 537.08 533.14 532.13 531.12<br>527.1 525.08 523.07 517.15 516.14 515.13 514.12 513.11 509.09<br>507.07 505.15 504.14 503.13 501.15 499.13 498.13 497.12 495.1<br>493.15 491.13 489.15 488.14 487.13 486.13 485.12 483.14 481.12<br>479.11 475.13 473.16 471.14 470.13 469.12 463.13 462.07 461.12<br>460.11 459.14 458.13 457.12 449.06 448.05 447.14 446.08 445.12<br>444.06 443.11 438.07 437.06 436.05 435.05 432.06 431.05 429.13<br>427.11 425.1 423.06 422.05 421.04 420.06 419.05 418.04 417.04<br>410.08 408.06 405.05 404.04 403.03 402.05 400.03 393.05 392.06<br>391.06 390.05 389.1 388.09 387.08 386.03 380.06 379.03 378.05<br>377.05 376.05 375.12 374.05 373.1 372.1 371.09 370.08 369.07<br>368.06 366.05 365.05 364.1 361.1 360.05 359.12 357.11 356.1<br>355.09 354.08 353.05 351.04 350.03 349.1 348.05 347.04 345.11<br>344.1 343.13 342.08 341.11 340.1 339.1 338.09 337.08 335.04<br>334.04 333.03 332.02 331.09 330.04 329.11 327.1 326.09 325.08<br>324.08 323.08 322.09 320.08 319.09 317.08 316.07 315.1 314.09<br>313.08 307.08 306.07 303.1 301.08 300.07 299.07 298.06 297.09<br>294.1 287.1 285.09 284.08 283.07 282.06 281.08 280.07 279.09<br>271.11 269.09 268.08 267.06 265.08 264.07 263.07 262.06 259.01<br>255.09 253.08 252.06 251.07 250.08 249.05 247.07 245.06 243.02<br>237.09 235.07 233.06 231.04 225.01 223.07 222.06 220.06 211.07<br>209.06 208.05 207.08 205.06 196.06 195.05 194.04 193.04 192.03<br>191.05 182.08 181.05 180.04 179.03 178.05 177.04 176.03 167.07<br>166.09 165.05 164.05 163.04 150.05 147.04 146.04 145.05 135.04<br>134.04 130.04 124.04 123.04 119.05 111.04 110.06 109.03 108.02<br>107.05 106.04
**References**	Wyler, H.; Rösler, H.; Mercier, M.; Dreiding, A.S. Präbetanin, ein Schwefelsäure-halbester des Betanins. Ein Beitrag zur Kenntnis der Betacyane. *Helvetica Chimica Acta* **1967**, 50, 545–561.<br>Schliemann, W.; Joy, R.W.; Komamine, A.; Metzger, J.W.; Nimtz, M.; Wray, V.; Strack, D. Betacyanins from plants and cell cultures of Phytolacca americana. *Phytochemistry* **1996**, 42, 1039–1046.<br>Slimen, I.B.; Najar, T.; Abderrabba, M. Chemical and Antioxidant Properties of Betalains. *J. Agric. Food Chem.* **2017**, 65, 675–689.<br>Kumorkiewicz-Jamro, A.; Świergosz, T.; Sutor, K.; Spórna-Kucab, A.; Wybraniec, S. Multi-colored shades of betalains: Recent advances in betacyanin chemistry. *Nat. Prod. Rep.* **2021**, 38, 2315–2346.

Rivinianin

**Chemical structure**	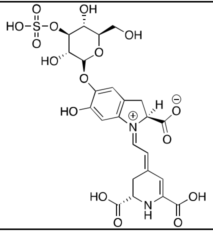
**Name:**	Rivinianin
**Chemical Formula**	$C_{24}H_{26}N_2O_{16}S$
**Molecular weight**	630.53
**Monoisotopic mass**	630.1003
***m/z* [M+H]**	631.1076
**Theorical fragments (*m/z*)**	631.11 630.1 629.09 628.08 615.11 614.1 613.1 612.09 611.08 603.11<br>602.1 601.1 599.12 598.11 597.1 596.09 595.09 594.08 593.07<br>591.11 589.1 588.09 587.12 586.11 585.1 584.09 583.09 581.11<br>580.1 579.09 578.08 577.08 575.06 573.1 571.12 570.12 569.11<br>568.1 567.09 566.08 565.08 563.1 561.1 559.09 558.08 557.11<br>556.1 555.09 553.11 552.1 551.1 550.09 549.08 545.11 543.09<br>542.08 541.08 540.07 539.1 538.09 537.08 533.14 532.13 531.12<br>527.1 525.08 523.07 517.15 516.14 515.13 514.12 513.11 509.09<br>507.07 505.15 504.14 503.13 501.15 499.13 498.13 497.12 495.1<br>493.15 491.13 489.15 488.14 487.13 486.13 485.12 483.14 481.12<br>479.11 475.13 473.16 471.14 470.13 469.12 463.13 462.07 461.12<br>460.11 459.14 458.13 457.12 449.06 448.05 447.14 446.08 445.12<br>444.06 443.11 438.07 437.06 436.05 435.05 432.06 431.05 429.13<br>427.11 425.1 423.06 422.05 421.04 420.06 419.05 418.04 417.04<br>410.08 408.06 405.05 404.04 403.03 402.05 400.03 393.05 392.06<br>391.06 390.05 389.1 388.09 387.08 386.03 380.06 379.03 378.05<br>377.05 376.05 375.12 374.05 373.1 372.1 371.09 370.08 369.07<br>368.06 366.05 365.05 364.1 361.1 360.05 359.12 357.11 356.1<br>355.09 354.08 353.05 351.04 350.03 349.1 348.05 347.04 345.11<br>344.1 343.13 342.08 341.11 340.1 339.1 338.09 337.08 335.04<br>334.04 333.03 332.02 331.09 330.04 329.11 327.1 326.09 325.08<br>324.08 323.08 322.09 320.08 319.09 317.08 316.07 315.1 314.09<br>313.08 307.08 306.07 303.1 301.08 300.07 299.07 298.06 297.09<br>294.1 287.1 285.09 284.08 283.07 282.06 281.08 280.07 279.09<br>271.11 269.09 268.08 267.06 265.08 264.07 263.07 262.06 259.01<br>255.09 253.08 252.06 251.07 250.08 249.05 247.07 245.06 243.02<br>237.09 235.07 233.06 231.04 225.01 223.07 222.06 220.06 211.07<br>209.06 208.05 207.08 205.06 196.06 195.05 194.04 193.04 192.03<br>191.05 182.08 181.05 180.04 179.03 178.05 177.04 176.03 167.07<br>166.09 165.05 164.05 163.04 150.05 147.04 146.04 145.05 135.04<br>134.04 130.04 124.04 123.04 119.05 111.04 110.06 109.03 108.02<br>107.05 106.04
**References**	Imperato, F. *Phytochemistry* **1975**, 14, 2526–2527.<br>Slimen, I.B.; Najar, T.; Abderrabba, M. Chemical and Antioxidant Properties of Betalains. *J. Agric. Food Chem.* **2017**, 65, 675–689.<br>Kumorkiewicz-Jamro, A.; Świergosz, T.; Sutor, K.; Spórna-Kucab, A.; Wybraniec, S. Multi-colored shades of betalains: Recent advances in betacyanin chemistry. *Nat. Prod. Rep.* **2021**, 38, 2315–2346.

Isorivinianin

**Chemical structure**	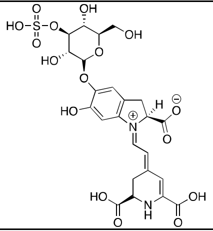
**Name:**	Isorivinianin
**Chemical Formula**	$C_{24}H_{26}N_2O_{16}S$
**Molecular weight**	630.53
**Monoisotopic mass**	630.1003
***m/z* [M+H]**	631.1076
**Theorical fragments (*m/z*)**	631.11 630.1 629.09 628.08 615.11 614.1 613.1 612.09 611.08 603.11<br>602.1 601.1 599.12 598.11 597.1 596.09 595.09 594.08 593.07<br>591.11 589.1 588.09 587.12 586.11 585.1 584.09 583.09 581.11<br>580.1 579.09 578.08 577.08 575.06 573.1 571.12 570.12 569.11<br>568.1 567.09 566.08 565.08 563.1 561.1 559.09 558.08 557.11<br>556.1 555.09 553.11 552.1 551.1 550.09 549.08 545.11 543.09<br>542.08 541.08 540.07 539.1 538.09 537.08 533.14 532.13 531.12<br>527.1 525.08 523.07 517.15 516.14 515.13 514.12 513.11 509.09<br>507.07 505.15 504.14 503.13 501.15 499.13 498.13 497.12 495.1<br>493.15 491.13 489.15 488.14 487.13 486.13 485.12 483.14 481.12<br>479.11 475.13 473.16 471.14 470.13 469.12 463.13 462.07 461.12<br>460.11 459.14 458.13 457.12 449.06 448.05 447.14 446.08 445.12<br>444.06 443.11 438.07 437.06 436.05 435.05 432.06 431.05 429.13<br>427.11 425.1 423.06 422.05 421.04 420.06 419.05 418.04 417.04<br>410.08 408.06 405.05 404.04 403.03 402.05 400.03 393.05 392.06<br>391.06 390.05 389.1 388.09 387.08 386.03 380.06 379.03 378.05<br>377.05 376.05 375.12 374.05 373.1 372.1 371.09 370.08 369.07<br>368.06 366.05 365.05 364.1 361.1 360.05 359.12 357.11 356.1<br>355.09 354.08 353.05 351.04 350.03 349.1 348.05 347.04 345.11<br>344.1 343.13 342.08 341.11 340.1 339.1 338.09 337.08 335.04<br>334.04 333.03 332.02 331.09 330.04 329.11 327.1 326.09 325.08<br>324.08 323.08 322.09 320.08 319.09 317.08 316.07 315.1 314.09<br>313.08 307.08 306.07 303.1 301.08 300.07 299.07 298.06 297.09<br>294.1 287.1 285.09 284.08 283.07 282.06 281.08 280.07 279.09<br>271.11 269.09 268.08 267.06 265.08 264.07 263.07 262.06 259.01<br>255.09 253.08 252.06 251.07 250.08 249.05 247.07 245.06 243.02<br>237.09 235.07 233.06 231.04 225.01 223.07 222.06 220.06 211.07<br>209.06 208.05 207.08 205.06 196.06 195.05 194.04 193.04 192.03<br>191.05 182.08 181.05 180.04 179.03 178.05 177.04 176.03 167.07<br>166.09 165.05 164.05 163.04 150.05 147.04 146.04 145.05 135.04<br>134.04 130.04 124.04 123.04 119.05 111.04 110.06 109.03 108.02<br>107.05 106.04
**References**	Imperato, F. *Phytochemistry* **1975**, 14, 2526–2527.<br>Slimen, I.B.; Najar, T.; Abderrabba, M. Chemical and Antioxidant Properties of Betalains. *J. Agric. Food Chem.* **2017**, 65, 675–689.<br>Kumorkiewicz-Jamro, A.; Świergosz, T.; Sutor, K.; Spórna-Kucab, A.; Wybraniec, S. Multi-colored shades of betalains: Recent advances in betacyanin chemistry. *Nat. Prod. Rep.* **2021**, 38, 2315–2346.

Betanin

**Chemical structure**	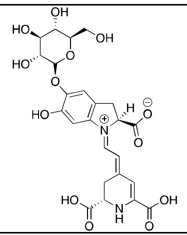
**Name:**	Betanin
**Chemical Formula**	$C_{24}H_{26}N_2O_{13}$
**Molecular weight**	550.47 g/mol
**Monoisotopic mass**	550.1435 Da
***m/z* [M+H]**	551.1508 Da
**Theorical fragments (*m/z*)**	551.15 550.14 549.14 548.13 535.16 534.15 533.14 532.13 531.12 523.16<br>522.15 521.14 519.16 518.15 517.15 516.14 515.13 514.12 513.11<br>511.16 509.14 508.13 507.16 506.15 505.15 504.14 503.13 501.15<br>500.14 499.13 498.13 497.12 495.1 493.15 491.17 490.16 489.15<br>488.14 487.13 486.13 485.12 483.14 481.15 479.13 478.12 477.15<br>476.14 475.13 473.16 472.15 471.14 470.13 469.12 465.15 463.13<br>462.13 461.12 460.11 459.14 458.13 457.12 447.14 445.12 443.11<br>429.13 427.11 425.1 419.11 417.09 389.1 388.09 387.08 386.07<br>382.11 375.12 373.1 372.1 371.09 370.08 369.07 368.1 366.12<br>364.1 361.1 360.1 359.12 358.11 357.11 356.1 355.09 354.08<br>353.08 352.1 351.06 349.1 347.09 345.11 344.1 343.13 342.09<br>341.09 340.1 339.1 338.09 337.08 335.09 331.09 330.12 329.11<br>328.1 327.1 326.09 325.09 324.08 323.08 322.09 320.08 319.09<br>317.08 316.07 315.1 314.09 313.09 312.11 311.1 310.09 307.08<br>306.07 303.1 301.08 300.07 299.07 298.06 297.1 296.09 294.1<br>288.11 287.1 286.09 285.1 284.08 283.07 282.06 281.08 280.07<br>279.09 273.1 271.08 270.07 269.09 268.09 267.06 265.08 264.07<br>263.07 262.06 255.09 254.08 253.08 252.06 251.07 250.08 249.05<br>247.07 245.06 237.09 235.07 233.06 231.04 223.07 222.06 220.06<br>211.07 209.06 208.05 207.08 205.06 196.06 195.05 194.04 193.04<br>192.03 191.05 182.08 181.05 180.04 179.03 178.05 177.04 176.03<br>167.07 166.09 165.05 164.05 163.04 150.05 147.04 146.04 145.05<br>135.04 134.04 130.04 124.04 123.04 119.05 111.04 110.06 109.03<br>108.02 107.05 106.04
**References**	Wyler, H.; Dreiding, A.S. Kristallisiertes Betanin. Vorläufige Mitteilung. *Helvetica Chimica Acta.* **1957**, 40, 191-192.<br>Piattelli, M.; Minale, L. Pigments of centrospermae—I. *Phytochemistry*, **1964**, 3, 307-311.<br>Heuer, S.; Wray, V.; Metzger, J.W.; Strack, D. Betacyanins from flowers of Gomphrena globosa. *Phytochemistry* **1992**, 31, 1801–1807.<br>Slimen, I.B.; Najar, T.; Abderrabba, M. Chemical and Antioxidant Properties of Betalains. *J. Agric. Food Chem.* **2017**, 65, 675–689.<br>Kumorkiewicz-Jamro, A.; Świergosz, T.; Sutor, K.; Spórna-Kucab, A.; Wybraniec, S. Multi-colored shades of betalains: Recent advances in betacyanin chemistry. *Nat. Prod. Rep.* **2021**, 38, 2315–2346.

Isobetanin

**Chemical structure**	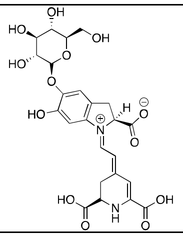
**Name:**	Isobetanin
**Chemical Formula**	$C_{24}H_{26}N_2O_{13}$
**Molecular weight**	550.47 g/mol
**Monoisotopic mass**	550.1435 Da
***m/z* [M+H]**	551.1508 Da
**Theorical fragments (*m/z*)**	551.15 550.14 549.14 548.13 535.16 534.15 533.14 532.13 531.12 523.16<br>522.15 521.14 519.16 518.15 517.15 516.14 515.13 514.12 513.11<br>511.16 509.14 508.13 507.16 506.15 505.15 504.14 503.13 501.15<br>500.14 499.13 498.13 497.12 495.1 493.15 491.17 490.16 489.15<br>488.14 487.13 486.13 485.12 483.14 481.15 479.13 478.12 477.15<br>476.14 475.13 473.16 472.15 471.14 470.13 469.12 465.15 463.13<br>462.13 461.12 460.11 459.14 458.13 457.12 447.14 445.12 443.11<br>429.13 427.11 425.1 419.11 417.09 389.1 388.09 387.08 386.07<br>382.11 375.12 373.1 372.1 371.09 370.08 369.07 368.1 366.12<br>364.1 361.1 360.1 359.12 358.11 357.11 356.1 355.09 354.08<br>353.08 352.1 351.06 349.1 347.09 345.11 344.1 343.13 342.09<br>341.09 340.1 339.1 338.09 337.08 335.09 331.09 330.12 329.11<br>328.1 327.1 326.09 325.09 324.08 323.08 322.09 320.08 319.09<br>317.08 316.07 315.1 314.09 313.09 312.11 311.1 310.09 307.08<br>306.07 303.1 301.08 300.07 299.07 298.06 297.1 296.09 294.1<br>288.11 287.1 286.09 285.1 284.08 283.07 282.06 281.08 280.07<br>279.09 273.1 271.08 270.07 269.09 268.09 267.06 265.08 264.07<br>263.07 262.06 255.09 254.08 253.08 252.06 251.07 250.08 249.05<br>247.07 245.06 237.09 235.07 233.06 231.04 223.07 222.06 220.06<br>211.07 209.06 208.05 207.08 205.06 196.06 195.05 194.04 193.04<br>192.03 191.05 182.08 181.05 180.04 179.03 178.05 177.04 176.03<br>167.07 166.09 165.05 164.05 163.04 150.05 147.04 146.04 145.05<br>135.04 134.04 130.04 124.04 123.04 119.05 111.04 110.06 109.03<br>108.02 107.05 106.04
**References**	Wyler, H.; Dreiding, A.S. Kristallisiertes Betanin. Vorläufige Mitteilung. *Helvetica Chimica Acta.* **1957**, 40, 191-192.<br>Piattelli, M.; Minale, L. Pigments of centrospermae—I. *Phytochemistry*, **1964**, 3, 307-311.<br>Heuer, S.; Wray, V.; Metzger, J.W.; Strack, D. Betacyanins from flowers of Gomphrena globosa. *Phytochemistry* **1992**, 31, 1801–1807.<br>Slimen, I.B.; Najar, T.; Abderrabba, M. Chemical and Antioxidant Properties of Betalains. *J. Agric. Food Chem.* **2017**, 65, 675–689.<br>Kumorkiewicz-Jamro, A.; Świergosz, T.; Sutor, K.; Spórna-Kucab, A.; Wybraniec, S. Multi-colored shades of betalains: Recent advances in betacyanin chemistry. *Nat. Prod. Rep.* **2021**, 38, 2315–2346.

Betanidin

**Chemical structure**	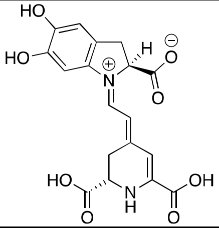									
**Name:**	Betanidin									
**Chemical Formula**	$C_{18}H_{16}N_2O_8$									
**Molecular weight**	388.33 g/mol									
**Monoisotopic mass**	388.0907 Da									
***m/z* [M+H]**	389.0979									
**Theorical fragments (*m/z*)**	389.1	388.09	387.08	386.07	374.09	373.1	372.1	371.09	370.08	369.07
		361.1	360.1	359.09	358.08	357.11	356.1	355.09	354.08	353.08
		352.07	351.06	349.1	347.09	346.08	345.11	344.1	343.09	342.08
		341.08	340.08	339.1	338.09	337.08	336.07	335.09	333.11	331.09
		330.08	329.11	328.11	327.1	326.09	325.08	324.07	323.07	319.09
		318.1	317.08	316.07	315.1	314.09	313.08	312.07	311.1	310.09
		309.09	308.08	307.07	305.11	304.08	303.1	302.1	301.08	300.07
		299.07	298.09	297.06	296.08	291.1	288.09	287.1	286.09	285.09
		284.12	283.07	282.1	281.09	280.07	279.06	275.1	274.09	273.09
		272.08	271.07	270.08	269.09	267.06	266.05	265.08	264.07	263.07
		262.06	260.09	259.08	258.08	257.09	256.1	255.08	254.08	253.08
		252.07	251.07	250.06	249.09	248.04	247.07	246.08	245.06	244.06
		242.08	240.07	235.07	234.08	233.06	232.06	230.08	228.07	223.07
		222.06	220.06	218.08	216.07	211.07	209.06	208.05	207.05	206.04
		205.06	204.07	202.05	196.06	195.05	194.04	193.04	192.05	191.05
		190.05	182.04	181.05	180.04	179.03	178.05	177.04	176.03	174.05
		169.04	168.07	167.08	166.05	165.05	164.05	163.04	162.03	152.07
		151.04	150.05	149.05	148.04	147.04	146.04	144.03	142.01	138.05
		137.02	136.04	135.04	134.04	132.04	126.05	124.04	123.04	119.05
		118.04	114.02	111.04	110.02	109.03	108.02	107.05	106.04	100.04
**References**	Wyler, H.; Dreiding, A.S. Darstellung und Abbauprodukte des Betanidins. 3. Über die Konstitution des Randenfarbstoffes Betanin. *Helv. Chim. Acta* **1959**, 42, 1699–702.<br>Wyler, H.; Dreiding, A.S. Deuterierung von Betanidin und Indicaxanthin. (E/Z)-stereoisomerie in Betalainen. *Helv. Chim. Acta* **1984**, 67, 1793–1800.<br>Slimen, I.B.; Najar, T.; Abderrabba, M. Chemical and Antioxidant Properties of Betalains. *J. Agric. Food Chem.* **2017**, 65, 675–689.<br>Kumorkiewicz-Jamro, A.; Świergosz, T.; Sutor, K.; Spórna-Kucab, A.; Wybraniec, S. Multi-colored shades of betalains: Recent advances in betacyanin chemistry. *Nat. Prod. Rep.* **2021**, 38, 2315–2346.									

Phyllocactin

**Chemical structure**	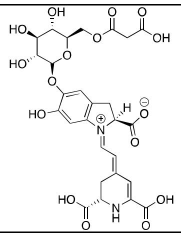
**Name:**	Phyllocactin
**Chemical Formula**	$C_{27}H_{28}N_2O_{16}$
**Molecular weight**	636.52
**Monoisotopic mass**	636.1439
***m/z* [M+H]**	637.1512
**Theorical fragments (*m/z*)**	637.15 636.14 635.14 634.13 621.16 620.15 619.14 618.13 617.12 609.16<br>608.15 607.14 605.16 604.15 603.15 602.14 601.13 600.12 599.11<br>597.16 595.14 594.13 593.16 592.15 591.15 590.14 589.13 587.15<br>586.14 585.14 584.13 583.12 581.1 579.15 577.17 575.15 574.14<br>573.14 572.13 571.12 569.14 567.15 565.13 564.12 563.15 562.14<br>561.14 559.16 558.15 557.14 556.13 555.12 551.15 550.14 549.14<br>548.13 547.12 546.11 545.14 544.13 543.12 535.16 534.15 533.14<br>532.13 531.12 529.11 523.16 522.15 521.14 519.16 517.15 516.14<br>515.13 514.12 513.11 511.1 509.14 507.16 505.15 504.14 503.13<br>501.15 499.13 498.13 497.12 495.1 493.15 491.13 489.15 488.14<br>487.13 486.13 485.12 481.15 479.13 478.12 477.15 476.14 475.13<br>473.16 471.14 470.13 469.12 468.11 465.15 463.13 461.12 460.11<br>459.14 458.13 457.12 455.11 447.14 445.12 444.11 443.11 442.1<br>441.09 429.1 428.09 427.09 426.1 425.1 424.09 423.08 412.1<br>411.09 410.08 409.08 408.09 406.08 399.09 398.11 397.1 396.09<br>395.1 394.09 393.08 392.07 389.1 388.09 387.08 386.07 385.08<br>383.1 382.09 380.1 375.12 373.1 372.09 371.1 370.08 369.07<br>367.1 366.09 365.09 361.1 360.1 359.12 358.11 357.11 356.07<br>355.09 354.09 353.09 351.06 349.1 347.09 345.11 343.13 342.09<br>341.11 340.1 339.1 338.09 337.08 336.08 335.08 331.09 329.11<br>327.1 326.09 325.09 324.08 323.08 321.06 320.08 319.09 317.08<br>316.07 315.1 314.09 313.08 312.11 307.08 306.07 303.1 301.08<br>300.07 299.07 298.06 297.1 294.1 287.1 285.1 284.08 283.07<br>282.06 281.08 280.07 279.09 273.1 271.08 270.07 269.09 268.09<br>267.06 265.08 264.07 263.07 262.06 255.09 253.08 252.06 251.07<br>250.08 249.05 247.07 245.06 237.09 235.07 233.06 231.04 223.07<br>222.06 211.07 207.08 205.06 196.06 194.04 193.04 192.03 182.08<br>180.04 179.03 178.05 177.04 165.05 164.05 163.04 150.05 147.04<br>146.04 135.04 134.04 130.04 123.04 119.05 111.04 109.03 108.02<br>107.05 106.04
**References**	Piattelli, M.; Minale, L. Pigments of centrospermae—I. *Phytochemistry*, **1964**, 3, 307-311.<br>Kobayashi, N.; Schmidt, J.; Nimtz, M.; Wray, V.; Schliemann, W. Betalains from Christmas cactus. *Phytochemistry* **2000**, 54, 419–426.<br>Slimen, I.B.; Najar, T.; Abderrabba, M. Chemical and Antioxidant Properties of Betalains. *J. Agric. Food Chem.* **2017**, 65, 675–689.<br>Kumorkiewicz-Jamro, A.; Świergosz, T.; Sutor, K.; Spórna-Kucab, A.; Wybraniec, S. Multi-colored shades of betalains: Recent advances in betacyanin chemistry. *Nat. Prod. Rep.* **2021**, 38, 2315–2346.

Isophyllocactin

**Chemical structure**	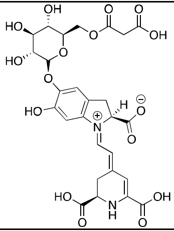
**Name:**	Isophyllocactin
**Chemical Formula**	$C_{27}H_{28}N_2O_{16}$
**Molecular weight**	636.52
**Monoisotopic mass**	636.1439
***m/z* [M+H]**	637.1512
**Theorical fragments (*m/z*)**	637.15 636.14 635.14 634.13 621.16 620.15 619.14 618.13 617.12 609.16<br>608.15 607.14 605.16 604.15 603.15 602.14 601.13 600.12 599.11<br>597.16 595.14 594.13 593.16 592.15 591.15 590.14 589.13 587.15<br>586.14 585.14 584.13 583.12 581.1 579.15 577.17 575.15 574.14<br>573.14 572.13 571.12 569.14 567.15 565.13 564.12 563.15 562.14<br>561.14 559.16 558.15 557.14 556.13 555.12 551.15 550.14 549.14<br>548.13 547.12 546.11 545.14 544.13 543.12 535.16 534.15 533.14<br>532.13 531.12 529.11 523.16 522.15 521.14 519.16 517.15 516.14<br>515.13 514.12 513.11 511.1 509.14 507.16 505.15 504.14 503.13<br>501.15 499.13 498.13 497.12 495.1 493.15 491.13 489.15 488.14<br>487.13 486.13 485.12 481.15 479.13 478.12 477.15 476.14 475.13<br>473.16 471.14 470.13 469.12 468.11 465.15 463.13 461.12 460.11<br>459.14 458.13 457.12 455.11 447.14 445.12 444.11 443.11 442.1<br>441.09 429.1 428.09 427.09 426.1 425.1 424.09 423.08 412.1<br>411.09 410.08 409.08 408.09 406.08 399.09 398.11 397.1 396.09<br>395.1 394.09 393.08 392.07 389.1 388.09 387.08 386.07 385.08<br>383.1 382.09 380.1 375.12 373.1 372.09 371.1 370.08 369.07<br>367.1 366.09 365.09 361.1 360.1 359.12 358.11 357.11 356.07<br>355.09 354.09 353.09 351.06 349.1 347.09 345.11 343.13 342.09<br>341.11 340.1 339.1 338.09 337.08 336.08 335.08 331.09 329.11<br>327.1 326.09 325.09 324.08 323.08 321.06 320.08 319.09 317.08<br>316.07 315.1 314.09 313.08 312.11 307.08 306.07 303.1 301.08<br>300.07 299.07 298.06 297.1 294.1 287.1 285.1 284.08 283.07<br>282.06 281.08 280.07 279.09 273.1 271.08 270.07 269.09 268.09<br>267.06 265.08 264.07 263.07 262.06 255.09 253.08 252.06 251.07<br>250.08 249.05 247.07 245.06 237.09 235.07 233.06 231.04 223.07<br>222.06 211.07 207.08 205.06 196.06 194.04 193.04 192.03 182.08<br>180.04 179.03 178.05 177.04 165.05 164.05 163.04 150.05 147.04<br>146.04 135.04 134.04 130.04 123.04 119.05 111.04 109.03 108.02<br>107.05 106.04
**References**	Piattelli, M.; Minale, L. Pigments of centrospermae—I. *Phytochemistry*, **1964**, 3, 307-311.<br>Kobayashi, N.; Schmidt, J.; Nimtz, M.; Wray, V.; Schliemann, W. Betalains from Christmas cactus. *Phytochemistry* **2000**, 54, 419–426.<br>Slimen, I.B.; Najar, T.; Abderrabba, M. Chemical and Antioxidant Properties of Betalains. *J. Agric. Food Chem.* **2017**, 65, 675–689.<br>Kumorkiewicz-Jamro, A.; Świergosz, T.; Sutor, K.; Spórna-Kucab, A.; Wybraniec, S. Multi-colored shades of betalains: Recent advances in betacyanin chemistry. *Nat. Prod. Rep.* **2021**, 38, 2315–2346.

Hylocerenin

**Chemical structure**	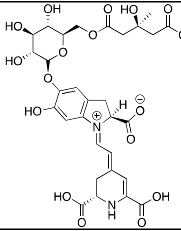
**Name:**	Hylocerenin
**Chemical Formula**	$C_{30}H_{34}N_2O_{17}$
**Molecular weight**	694.60
**Monoisotopic mass**	694.1857
***m/z* [M+H]**	695.1930
**Theorical fragments (*m/z*)**	695.19 694.19 693.18 692.17 679.2 678.19 677.18 676.17 675.17 667.2<br>666.19 665.18 663.2 662.2 661.19 660.18 659.17 658.16 657.16<br>655.2 653.18 652.17 651.2 650.2 649.19 648.18 647.17 645.19<br>644.18 643.18 642.17 641.16 639.15 637.19 635.21 633.19 632.18<br>631.18 630.17 629.16 627.18 625.19 623.17 622.16 621.19 620.18<br>619.18 617.2 616.19 615.18 614.17 613.17 609.19 607.18 606.17<br>605.16 604.15 603.18 602.17 601.17 593.2 591.18 589.17 587.15<br>575.19 573.17 571.16 569.14 551.15 550.14 549.14 535.16 534.15<br>533.14 532.13 531.12 526.16 523.16 522.15 521.14 519.16 517.15<br>516.14 515.13 514.12 513.11 511.16 509.14 507.16 505.15 504.14<br>503.13 502.16 501.15 500.14 499.13 498.13 497.12 495.1 493.15<br>491.13 489.15 488.14 487.13 486.14 485.13 484.14 483.14 482.13<br>481.15 479.13 478.12 477.15 476.14 475.13 471.14 470.14 469.13<br>468.13 467.12 465.15 463.13 461.12 460.11 459.14 458.13 457.13<br>456.15 455.14 454.13 453.14 452.13 451.12 450.12 447.14 445.12<br>443.11 441.14 440.13 430.13 429.14 427.11 425.14 424.14 423.13<br>417.14 415.12 414.12 413.14 412.14 411.13 401.14 399.13 398.12<br>397.11 396.11 395.13 394.13 393.12 389.1 388.09 387.08 386.07<br>385.15 383.13 381.12 379.1 375.12 373.1 372.1 371.09 370.08<br>369.07 361.1 360.1 359.12 358.11 357.11 356.1 355.09 354.08<br>353.08 351.06 349.1 347.09 345.11 343.13 342.09 341.11 340.1<br>339.1 338.09 337.08 335.09 331.09 329.11 327.1 326.09 325.09<br>324.08 323.08 319.09 317.08 316.07 315.1 314.09 313.08 307.08<br>306.07 303.1 301.08 300.07 299.07 298.06 297.1 287.1 285.1<br>284.08 283.07 282.06 281.08 280.07 279.09 273.1 271.08 270.07<br>269.09 268.09 267.06 265.08 264.07 263.07 262.06 255.09 253.08<br>252.06 251.07 250.08 249.05 247.07 245.06 237.09 235.07 233.06<br>231.04 223.07 211.07 207.08 205.06 196.06 194.04 193.04 182.08<br>180.04 179.03 178.05 177.04 165.05 164.05 163.04 147.04 146.04<br>135.04 134.04 130.04 123.04 119.05 111.04 109.03 108.02 107.05<br>106.04
**References**	Wybraniec, S.; Platzner, I.; Geresh, S.; Gottlieb, H.E.; Haimberg, M.; Mogilnitzki, M.; Mizrahi, Y. Betacyanins from vine cactus Hylocereus polyrhizus. *Phytochemistry* **2001**, 58, 1209–1212.<br>Slimen, I.B.; Najar, T.; Abderrabba, M. Chemical and Antioxidant Properties of Betalains. *J. Agric. Food Chem.* **2017**, 65, 675–689.<br>Kumorkiewicz-Jamro, A.; Świergosz, T.; Sutor, K.; Spórna-Kucab, A.; Wybraniec, S. Multi-colored shades of betalains: Recent advances in betacyanin chemistry. *Nat. Prod. Rep.* **2021**, 38, 2315–2346.

Isohylocerenin

**Chemical structure**	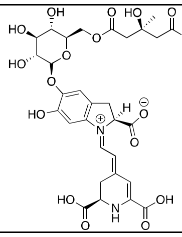
**Name:**	Isohylocerenin
**Chemical Formula**	$C_{30}H_{34}N_2O_{17}$
**Molecular weight**	694.60
**Monoisotopic mass**	694.1857
***m/z* [M+H]**	695.1930
**Theorical fragments (*m/z*)**	695.19 694.19 693.18 692.17 679.2 678.19 677.18 676.17 675.17 667.2<br>666.19 665.18 663.2 662.2 661.19 660.18 659.17 658.16 657.16<br>655.2 653.18 652.17 651.2 650.2 649.19 648.18 647.17 645.19<br>644.18 643.18 642.17 641.16 639.15 637.19 635.21 633.19 632.18<br>631.18 630.17 629.16 627.18 625.19 623.17 622.16 621.19 620.18<br>619.18 617.2 616.19 615.18 614.17 613.17 609.19 607.18 606.17<br>605.16 604.15 603.18 602.17 601.17 593.2 591.18 589.17 587.15<br>575.19 573.17 571.16 569.14 551.15 550.14 549.14 535.16 534.15<br>533.14 532.13 531.12 526.16 523.16 522.15 521.14 519.16 517.15<br>516.14 515.13 514.12 513.11 511.16 509.14 507.16 505.15 504.14<br>503.13 502.16 501.15 500.14 499.13 498.13 497.12 495.1 493.15<br>491.13 489.15 488.14 487.13 486.14 485.13 484.14 483.14 482.13<br>481.15 479.13 478.12 477.15 476.14 475.13 471.14 470.14 469.13<br>468.13 467.12 465.15 463.13 461.12 460.11 459.14 458.13 457.13<br>456.15 455.14 454.13 453.14 452.13 451.12 450.12 447.14 445.12<br>443.11 441.14 440.13 430.13 429.14 427.11 425.14 424.14 423.13<br>417.14 415.12 414.12 413.14 412.14 411.13 401.14 399.13 398.12<br>397.11 396.11 395.13 394.13 393.12 389.1 388.09 387.08 386.07<br>385.15 383.13 381.12 379.1 375.12 373.1 372.1 371.09 370.08<br>369.07 361.1 360.1 359.12 358.11 357.11 356.1 355.09 354.08<br>353.08 351.06 349.1 347.09 345.11 343.13 342.09 341.11 340.1<br>339.1 338.09 337.08 335.09 331.09 329.11 327.1 326.09 325.09<br>324.08 323.08 319.09 317.08 316.07 315.1 314.09 313.08 307.08<br>306.07 303.1 301.08 300.07 299.07 298.06 297.1 287.1 285.1<br>284.08 283.07 282.06 281.08 280.07 279.09 273.1 271.08 270.07<br>269.09 268.09 267.06 265.08 264.07 263.07 262.06 255.09 253.08<br>252.06 251.07 250.08 249.05 247.07 245.06 237.09 235.07 233.06<br>231.04 223.07 211.07 207.08 205.06 196.06 194.04 193.04 182.08<br>180.04 179.03 178.05 177.04 165.05 164.05 163.04 147.04 146.04<br>135.04 134.04 130.04 123.04 119.05 111.04 109.03 108.02 107.05<br>106.04
**References**	Wybraniec, S.; Platzner, I.; Geresh, S.; Gottlieb, H.E.; Haimberg, M.; Mogilnitzki, M.; Mizrahi, Y. Betacyanins from vine cactus Hylocereus polyrhizus. *Phytochemistry* **2001**, 58, 1209–1212.<br>Slimen, I.B.; Najar, T.; Abderrabba, M. Chemical and Antioxidant Properties of Betalains. *J. Agric. Food Chem.* **2017**, 65, 675–689.<br>Kumorkiewicz-Jamro, A.; Świergosz, T.; Sutor, K.; Spórna-Kucab, A.; Wybraniec, S. Multi-colored shades of betalains: Recent advances in betacyanin chemistry. *Nat. Prod. Rep.* **2021**, 38, 2315–2346.

Lampranthin I

**Chemical structure**	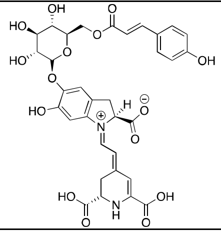
**Name:**	Lampranthin I
**Chemical Formula**	$C_{33}H_{32}N_2O_{15}$
**Molecular weight**	696.62
**Monoisotopic mass**	696.1803
***m/z* [M+H]**	697.1875
**Theorical fragments (*m/z*)**	697.19 696.18 695.17 694.16 681.19 680.18 679.18 678.17 677.16 669.19<br>668.18 667.18 665.2 664.19 663.18 662.17 661.17 660.16 659.15<br>657.19 655.18 654.17 653.2 652.19 651.18 650.17 649.17 647.19<br>646.18 645.17 644.16 643.16 641.14 639.18 637.2 636.19 635.19<br>634.18 633.17 632.16 631.16 629.18 627.18 625.17 624.16 623.19<br>622.18 621.17 619.19 618.18 617.18 616.17 615.16 611.19 609.17<br>608.16 607.16 606.15 605.18 604.17 603.16 593.18 591.16 589.15<br>575.17 573.15 571.13 533.14 532.13 531.12 528.15 517.15 516.14<br>515.13 514.12 513.11 512.16 510.14 505.15 504.15 503.13 502.13<br>501.13 499.13 498.13 497.12 495.1 493.15 491.13 489.15 488.13<br>487.12 486.14 485.12 484.12 483.12 481.12 479.11 476.16 475.13<br>474.14 473.16 471.13 470.12 469.11 468.13 466.11 463.13 461.12<br>460.11 459.13 458.14 457.14 456.13 453.12 452.11 447.14 446.14<br>445.12 444.13 443.13 442.13 440.13 434.14 432.13 431.13 429.13<br>427.11 426.13 425.12 419.13 417.12 416.11 414.13 413.12 401.12<br>400.12 399.11 398.1 396.12 395.11 389.1 388.09 387.08 386.07<br>383.11 381.1 375.12 373.1 372.1 371.09 370.08 369.07 364.1<br>361.1 360.1 359.12 357.11 356.1 355.09 354.08 353.08 351.06<br>349.1 347.09 345.11 344.1 343.13 342.08 341.11 340.1 339.1<br>338.09 337.08 335.09 331.09 329.11 327.1 326.09 325.08 324.08<br>323.08 322.09 320.08 319.09 317.08 316.07 315.1 314.09 313.08<br>309.1 307.08 306.07 303.1 301.08 300.07 299.07 298.06 297.09<br>294.1 291.09 287.1 285.09 284.08 283.07 282.06 281.08 280.07<br>279.09 271.11 269.09 268.08 267.06 265.08 264.07 263.07 262.06<br>255.09 253.08 252.06 251.07 250.08 249.05 247.07 245.06 237.09<br>235.07 233.06 231.04 223.07 222.06 220.06 211.07 209.06 208.05<br>207.08 205.06 196.06 195.05 194.04 193.04 192.03 191.05 182.08<br>181.05 180.04 179.03 178.05 177.04 176.03 167.07 166.09 165.05<br>164.05 163.04 150.05 147.04 146.04 145.05 135.04 134.04 130.04<br>124.04 123.04 119.05 111.04 110.06 109.03 108.02 107.05 106.04
**References**	Piattelli, M.; Impellizzeri, G. Betacyanins from Lampranthus sp. (aizoaceae). *Phytochemistry* **1969**, 8, 1595–1596.<br>Slimen, I.B.; Najar, T.; Abderrabba, M. Chemical and Antioxidant Properties of Betalains. *J. Agric. Food Chem.* **2017**, 65, 675–689.<br>Kumorkiewicz-Jamro, A.; Świergosz, T.; Sutor, K.; Spórna-Kucab, A.; Wybraniec, S. Multi-colored shades of betalains: Recent advances in betacyanin chemistry. *Nat. Prod. Rep.* **2021**, 38, 2315–2346.

Isoampranthin I

**Chemical structure**	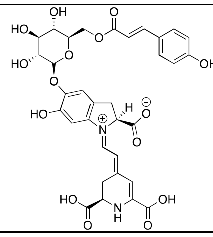
**Name:**	Isolampranthin I
**Chemical Formula**	$C_{33}H_{32}N_2O_{15}$
**Molecular weight**	696.62
**Monoisotopic mass**	696.1803
***m/z* [M+H]**	697.1875
**Theorical fragments (*m/z*)**	697.19 696.18 695.17 694.16 681.19 680.18 679.18 678.17 677.16 669.19<br>668.18 667.18 665.2 664.19 663.18 662.17 661.17 660.16 659.15<br>657.19 655.18 654.17 653.2 652.19 651.18 650.17 649.17 647.19<br>646.18 645.17 644.16 643.16 641.14 639.18 637.2 636.19 635.19<br>634.18 633.17 632.16 631.16 629.18 627.18 625.17 624.16 623.19<br>622.18 621.17 619.19 618.18 617.18 616.17 615.16 611.19 609.17<br>608.16 607.16 606.15 605.18 604.17 603.16 593.18 591.16 589.15<br>575.17 573.15 571.13 533.14 532.13 531.12 528.15 517.15 516.14<br>515.13 514.12 513.11 512.16 510.14 505.15 504.15 503.13 502.13<br>501.13 499.13 498.13 497.12 495.1 493.15 491.13 489.15 488.13<br>487.12 486.14 485.12 484.12 483.12 481.12 479.11 476.16 475.13<br>474.14 473.16 471.13 470.12 469.11 468.13 466.11 463.13 461.12<br>460.11 459.13 458.14 457.14 456.13 453.12 452.11 447.14 446.14<br>445.12 444.13 443.13 442.13 440.13 434.14 432.13 431.13 429.13<br>427.11 426.13 425.12 419.13 417.12 416.11 414.13 413.12 401.12<br>400.12 399.11 398.1 396.12 395.11 389.1 388.09 387.08 386.07<br>383.11 381.1 375.12 373.1 372.1 371.09 370.08 369.07 364.1<br>361.1 360.1 359.12 357.11 356.1 355.09 354.08 353.08 351.06<br>349.1 347.09 345.11 344.1 343.13 342.08 341.11 340.1 339.1<br>338.09 337.08 335.09 331.09 329.11 327.1 326.09 325.08 324.08<br>323.08 322.09 320.08 319.09 317.08 316.07 315.1 314.09 313.08<br>309.1 307.08 306.07 303.1 301.08 300.07 299.07 298.06 297.09<br>294.1 291.09 287.1 285.09 284.08 283.07 282.06 281.08 280.07<br>279.09 271.11 269.09 268.08 267.06 265.08 264.07 263.07 262.06<br>255.09 253.08 252.06 251.07 250.08 249.05 247.07 245.06 237.09<br>235.07 233.06 231.04 223.07 222.06 220.06 211.07 209.06 208.05<br>207.08 205.06 196.06 195.05 194.04 193.04 192.03 191.05 182.08<br>181.05 180.04 179.03 178.05 177.04 176.03 167.07 166.09 165.05<br>164.05 163.04 150.05 147.04 146.04 145.05 135.04 134.04 130.04<br>124.04 123.04 119.05 111.04 110.06 109.03 108.02 107.05 106.04
**References**	Piattelli, M.; Impellizzeri, G. Betacyanins from Lampranthus sp. (aizoaceae). *Phytochemistry* **1969**, 8, 1595–1596.<br>Slimen, I.B.; Najar, T.; Abderrabba, M. Chemical and Antioxidant Properties of Betalains. *J. Agric. Food Chem.* **2017**, 65, 675–689.<br>Kumorkiewicz-Jamro, A.; Świergosz, T.; Sutor, K.; Spórna-Kucab, A.; Wybraniec, S. Multi-colored shades of betalains: Recent advances in betacyanin chemistry. *Nat. Prod. Rep.* **2021**, 38, 2315–2346.

Lampranthin II

**Chemical structure**	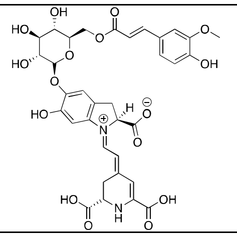
**Name:**	Lampranthin II
**Chemical Formula**	$C_{34}H_{34}N_2O_{16}$
**Molecular weight**	726.64
**Monoisotopic mass**	726.1908
***m/z* [M+H]**	727.1981
**Theorical fragments (*m/z*)**	727.2 726.19 725.18 724.17 711.2 710.2 709.19 708.18 707.17 699.2<br>698.2 697.19 695.21 694.2 693.19 692.18 691.18 690.17 689.16<br>687.2 685.19 684.18 683.21 682.2 681.19 680.18 679.18 677.2<br>676.19 675.18 674.17 673.17 671.15 669.19 667.21 666.21 665.2<br>664.19 663.18 662.17 661.17 659.19 657.19 655.18 654.17 653.2<br>652.19 651.18 649.2 648.19 647.19 646.18 645.17 641.2 639.18<br>638.17 637.17 636.16 635.19 634.18 633.17 623.19 621.17 619.16<br>605.18 603.16 601.15 558.16 545.15 544.14 542.17 540.15 534.16<br>533.14 532.14 531.14 528.15 527.14 519.15 518.14 517.15 516.15<br>515.13 514.13 513.11 506.17 505.15 504.14 503.13 501.14 500.13<br>499.13 498.13 497.12 496.12 495.1 493.15 491.13 489.15 488.16<br>487.13 486.14 485.12 483.13 482.12 481.12 479.11 476.16 475.13<br>474.14 473.14 472.14 471.14 470.13 469.12 464.16 463.13 462.14<br>461.14 460.11 459.14 458.13 457.12 456.14 455.13 449.14 447.13<br>446.12 445.12 444.14 443.13 431.13 430.13 429.12 428.11 427.11<br>426.13 425.12 419.11 417.09 413.12 411.11 389.1 388.09 387.08<br>386.07 375.12 373.1 372.1 371.09 370.08 369.07 364.1 361.1<br>360.1 359.12 357.11 356.1 355.09 354.08 353.08 351.06 349.1<br>347.09 345.11 344.1 343.13 342.08 341.11 340.1 339.1 338.09<br>337.08 335.09 331.09 329.11 327.1 326.09 325.08 324.08 323.08<br>322.09 321.1 320.08 319.09 317.08 316.07 315.1 314.09 313.08<br>307.08 306.07 303.1 301.08 300.07 299.07 298.06 297.09 294.1<br>287.1 285.09 284.08 283.07 282.06 281.08 280.07 279.09 271.11<br>269.09 268.08 267.06 265.08 264.07 263.07 262.06 255.09 253.08<br>252.06 251.07 250.08 249.05 247.07 245.06 237.09 235.07 233.06<br>231.04 223.07 222.06 220.06 211.07 209.06 208.05 207.08 205.06<br>196.06 195.05 194.04 193.04 192.03 191.05 182.08 181.05 180.04<br>179.03 178.05 177.04 176.03 167.07 166.09 165.05 164.05 163.04<br>150.05 147.04 146.04 145.05 135.04 134.04 130.04 124.04 123.04<br>119.05 111.04 110.06 109.03 108.02 107.05 106.04
**References**	Strack, D.; Bokern, M.; Marxen, N.; Wray, V. Feruloylbetanin from petals of Lampranthus and feruloylamaranthin from cell suspension cultures of Chenopodium rubrum. *Phytochemistry* **1988**, 27, 3529–3531.<br>Heuer, S.; Wray, V.; Metzger, J.W.; Strack, D. Betacyanins from flowers of Gomphrena globosa. *Phytochemistry* **1992**, 31, 1801–1807.<br>Piattelli, M.; Impellizzeri, G. Betacyanins from Lampranthus sp. (aizoaceae). *Phytochemistry* **1969**, 8, 1595–1596.<br>Slimen, I.B.; Najar, T.; Abderrabba, M. Chemical and Antioxidant Properties of Betalains. *J. Agric. Food Chem.* **2017**, 65, 675–689.<br>Kumorkiewicz-Jamro, A.; Świergosz, T.; Sutor, K.; Spórna-Kucab, A.; Wybraniec, S. Multi-colored shades of betalains: Recent advances in betacyanin chemistry. *Nat. Prod. Rep.* **2021**, 38, 2315–2346.

Isolampranthin II

**Chemical structure**	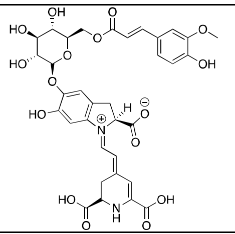
**Name:**	Isolampranthin II
**Chemical Formula**	$C_{34}H_{34}N_2O_{16}$
**Molecular weight**	726.64
**Monoisotopic mass**	726.1908
***m/z* [M+H]**	727.1981
**Theorical fragments (*m/z*)**	727.2 726.19 725.18 724.17 711.2 710.2 709.19 708.18 707.17 699.2<br>698.2 697.19 695.21 694.2 693.19 692.18 691.18 690.17 689.16<br>687.2 685.19 684.18 683.21 682.2 681.19 680.18 679.18 677.2<br>676.19 675.18 674.17 673.17 671.15 669.19 667.21 666.21 665.2<br>664.19 663.18 662.17 661.17 659.19 657.19 655.18 654.17 653.2<br>652.19 651.18 649.2 648.19 647.19 646.18 645.17 641.2 639.18<br>638.17 637.17 636.16 635.19 634.18 633.17 623.19 621.17 619.16<br>605.18 603.16 601.15 558.16 545.15 544.14 542.17 540.15 534.16<br>533.14 532.14 531.14 528.15 527.14 519.15 518.14 517.15 516.15<br>515.13 514.13 513.11 506.17 505.15 504.14 503.13 501.14 500.13<br>499.13 498.13 497.12 496.12 495.1 493.15 491.13 489.15 488.16<br>487.13 486.14 485.12 483.13 482.12 481.12 479.11 476.16 475.13<br>474.14 473.14 472.14 471.14 470.13 469.12 464.16 463.13 462.14<br>461.14 460.11 459.14 458.13 457.12 456.14 455.13 449.14 447.13<br>446.12 445.12 444.14 443.13 431.13 430.13 429.12 428.11 427.11<br>426.13 425.12 419.11 417.09 413.12 411.11 389.1 388.09 387.08<br>386.07 375.12 373.1 372.1 371.09 370.08 369.07 364.1 361.1<br>360.1 359.12 357.11 356.1 355.09 354.08 353.08 351.06 349.1<br>347.09 345.11 344.1 343.13 342.08 341.11 340.1 339.1 338.09<br>337.08 335.09 331.09 329.11 327.1 326.09 325.08 324.08 323.08<br>322.09 321.1 320.08 319.09 317.08 316.07 315.1 314.09 313.08<br>307.08 306.07 303.1 301.08 300.07 299.07 298.06 297.09 294.1<br>287.1 285.09 284.08 283.07 282.06 281.08 280.07 279.09 271.11<br>269.09 268.08 267.06 265.08 264.07 263.07 262.06 255.09 253.08<br>252.06 251.07 250.08 249.05 247.07 245.06 237.09 235.07 233.06<br>231.04 223.07 222.06 220.06 211.07 209.06 208.05 207.08 205.06<br>196.06 195.05 194.04 193.04 192.03 191.05 182.08 181.05 180.04<br>179.03 178.05 177.04 176.03 167.07 166.09 165.05 164.05 163.04<br>150.05 147.04 146.04 145.05 135.04 134.04 130.04 124.04 123.04<br>119.05 111.04 110.06 109.03 108.02 107.05 106.04
**References**	Strack, D.; Bokern, M.; Marxen, N.; Wray, V. Feruloylbetanin from petals of Lampranthus and feruloylamaranthin from cell suspension cultures of Chenopodium rubrum. *Phytochemistry* **1988**, 27, 3529–3531.<br>Heuer, S.; Wray, V.; Metzger, J.W.; Strack, D. Betacyanins from flowers of Gomphrena globosa. *Phytochemistry* **1992**, 31, 1801–1807.<br>Piattelli, M.; Impellizzeri, G. Betacyanins from Lampranthus sp. (aizoaceae). *Phytochemistry* **1969**, 8, 1595–1596.<br>Slimen, I.B.; Najar, T.; Abderrabba, M. Chemical and Antioxidant Properties of Betalains. *J. Agric. Food Chem.* **2017**, 65, 675–689.<br>Kumorkiewicz-Jamro, A.; Świergosz, T.; Sutor, K.; Spórna-Kucab, A.; Wybraniec, S. Multi-colored shades of betalains: Recent advances in betacyanin chemistry. *Nat. Prod. Rep.* **2021**, 38, 2315–2346.

4’-*O*-malonyl-betanin

**Chemical structure**	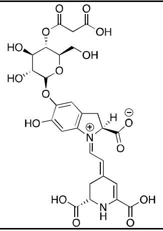
**Name:**	4´-*O*-malonyl-betanin
**Chemical Formula**	$C_{27}H_{28}N_2O_{16}$
**Molecular weight**	636.52
**Monoisotopic mass**	636.1439
***m/z* [M+H]**	637.1512
**Theorical fragments (*m/z*)**	637.15 636.14 635.14 634.13 621.16 620.15 619.14 618.13 617.12<br>609.16 608.15 607.14 605.16 604.15 603.15 602.14 601.13<br>600.12 599.11 597.16 595.14 594.13 593.16 592.15 591.15<br>590.14 589.13 587.15 586.14 585.14 584.13 583.12 581.1<br>579.15 577.17 575.15 574.14 573.14 572.13 571.12 569.14<br>567.15 565.13 564.12 563.15 562.14 561.14 559.16 558.15<br>557.14 556.13 555.12 551.15 550.14 549.14 548.13 547.12<br>546.11 545.14 544.13 543.12 535.16 534.15 533.14 532.13<br>531.12 529.11 523.16 522.15 521.14 519.16 517.15 516.14<br>515.13 514.12 513.11 511.1 509.14 507.16 505.15 504.14<br>503.13 501.15 499.13 498.13 497.12 495.1 493.15 491.13<br>489.15 488.14 487.13 486.13 485.12 481.15 479.13 478.12<br>477.15 476.14 475.13 473.16 471.14 470.13 469.12 468.11<br>465.15 463.13 461.12 460.11 459.14 458.13 457.12 455.11<br>447.14 445.12 444.11 443.11 442.1 441.09 429.1 428.09<br>427.09 426.1 425.1 424.09 423.08 412.1 411.09 410.08<br>409.08 408.09 406.08 399.09 398.11 397.1 396.09 395.1<br>394.09 393.08 392.07 389.1 388.09 387.08 386.07 385.08<br>383.1 382.09 380.1 375.12 373.1 372.09 371.1 370.08<br>369.07 367.1 366.09 365.09 361.1 360.1 359.12 358.11<br>357.11 356.07 355.09 354.09 353.09 351.06 349.1 347.09<br>345.11 343.13 342.09 341.11 340.1 339.1 338.09 337.08<br>336.08 335.08 331.09 329.11 327.1 326.09 325.09 324.08<br>323.08 321.06 320.08 319.09 317.08 316.07 315.1 314.09<br>313.08 312.11 307.08 306.07 303.1 301.08 300.07 299.07<br>298.06 297.1 294.1 287.1 285.1 284.08 283.07 282.06<br>281.08 280.07 279.09 273.1 271.08 270.07 269.09 268.09<br>267.06 265.08 264.07 263.07 262.06 255.09 253.08 252.06<br>251.07 250.08 249.05 247.07 245.06 237.09 235.07 233.06<br>231.04 223.07 222.06 211.07 207.08 205.06 196.06 194.04<br>193.04 192.03 182.08 180.04 179.03 178.05 177.04 165.05<br>164.05 163.04 150.05 147.04 146.04 135.04 134.04 130.04<br>123.04 119.05 111.04 109.03 108.02 107.05 106.04
**References**	Wybraniec, S.; Nowak-Wydra, B.; Mitka, K.; Kowalski, P.; Mizrahi, Y. Minor betalains in fruits of Hylocereus species. *Phytochemistry* **2007**, 68, 251–259.<br>Slimen, I.B.; Najar, T.; Abderrabba, M. Chemical and Antioxidant Properties of Betalains. *J. Agric. Food Chem.* **2017**, 65, 675–689.<br>Kumorkiewicz-Jamro, A.; Świergosz, T.; Sutor, K.; Spórna-Kucab, A.; Wybraniec, S. Multi-colored shades of betalains: Recent advances in betacyanin chemistry. *Nat. Prod. Rep.* **2021**, 38, 2315–2346.

4’-*O*-malonyl-isobetanin

**Chemical structure**	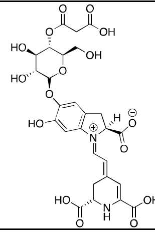
**Name:**	4´-*O*-malonyl-isobetanin
**Chemical Formula**	$C_{27}H_{28}N_2O_{16}$
**Molecular weight**	636.52
**Monoisotopic mass**	636.1439
***m/z* [M+H]**	637.1512
**Theorical fragments (*m/z*)**	637.15 636.14 635.14 634.13 621.16 620.15 619.14 618.13 617.12 609.16<br>608.15 607.14 605.16 604.15 603.15 602.14 601.13 600.12 599.11<br>597.16 595.14 594.13 593.16 592.15 591.15 590.14 589.13 587.15<br>586.14 585.14 584.13 583.12 581.1 579.15 577.17 575.15 574.14<br>573.14 572.13 571.12 569.14 567.15 565.13 564.12 563.15 562.14<br>561.14 559.16 558.15 557.14 556.13 555.12 551.15 550.14 549.14<br>548.13 547.12 546.11 545.14 544.13 543.12 535.16 534.15 533.14<br>532.13 531.12 529.11 523.16 522.15 521.14 519.16 517.15 516.14<br>515.13 514.12 513.11 511.1 509.14 507.16 505.15 504.14 503.13<br>501.15 499.13 498.13 497.12 495.1 493.15 491.13 489.15 488.14<br>487.13 486.13 485.12 481.15 479.13 478.12 477.15 476.14 475.13<br>473.16 471.14 470.13 469.12 468.11 465.15 463.13 461.12 460.11<br>459.14 458.13 457.12 455.11 447.14 445.12 444.11 443.11 442.1<br>441.09 429.1 428.09 427.09 426.1 425.1 424.09 423.08 412.1<br>411.09 410.08 409.08 408.09 406.08 399.09 398.11 397.1 396.09<br>395.1 394.09 393.08 392.07 389.1 388.09 387.08 386.07 385.08<br>383.1 382.09 380.1 375.12 373.1 372.09 371.1 370.08 369.07<br>367.1 366.09 365.09 361.1 360.1 359.12 358.11 357.11 356.07<br>355.09 354.09 353.09 351.06 349.1 347.09 345.11 343.13 342.09<br>341.11 340.1 339.1 338.09 337.08 336.08 335.08 331.09 329.11<br>327.1 326.09 325.09 324.08 323.08 321.06 320.08 319.09 317.08<br>316.07 315.1 314.09 313.08 312.11 307.08 306.07 303.1 301.08<br>300.07 299.07 298.06 297.1 294.1 287.1 285.1 284.08 283.07<br>282.06 281.08 280.07 279.09 273.1 271.08 270.07 269.09 268.09<br>267.06 265.08 264.07 263.07 262.06 255.09 253.08 252.06 251.07<br>250.08 249.05 247.07 245.06 237.09 235.07 233.06 231.04 223.07<br>222.06 211.07 207.08 205.06 196.06 194.04 193.04 192.03 182.08<br>180.04 179.03 178.05 177.04 165.05 164.05 163.04 150.05 147.04<br>146.04 135.04 134.04 130.04 123.04 119.05 111.04 109.03 108.02<br>107.05 106.04
**References**	Wybraniec, S.; Nowak-Wydra, B.; Mitka, K.; Kowalski, P.; Mizrahi, Y. Minor betalains in fruits of Hylocereus species. *Phytochemistry* **2007**, 68, 251–259.<br>Slimen, I.B.; Najar, T.; Abderrabba, M. Chemical and Antioxidant Properties of Betalains. *J. Agric. Food Chem.* **2017**, 65, 675–689.<br>Kumorkiewicz-Jamro, A.; Świergosz, T.; Sutor, K.; Spórna-Kucab, A.; Wybraniec, S. Multi-colored shades of betalains: Recent advances in betacyanin chemistry. *Nat. Prod. Rep.* **2021**, 38, 2315–2346.

2-decarboxy-2,3-dehydro-betanin

**Chemical structure**	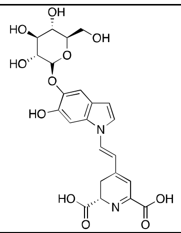
**Name:**	2-decarboxy-2,3-dehydro-betanin (2-decarboxy-xanbetanin)
**Chemical Formula**	$C_{23}H_{24}N_2O_{11}$
**Molecular weight**	504.45
**Monoisotopic mass**	504.1380
***m/z* [M+H]**	505.1453
**Theorical fragments (*m/z*)**	505.15 504.14 503.13 502.12 501.11 500.11 489.15 488.14 487.13 486.13<br>485.12 484.11 483.1 479.13 478.12 477.15 475.13 474.13 473.12<br>472.11 471.14 470.13 469.12 468.12 467.11 465.15 463.13 461.16<br>460.11 459.14 458.1 457.12 456.12 455.11 454.1 453.13 451.11<br>449.12 447.14 445.16 444.12 443.14 442.1 441.13 440.12 439.11<br>438.11 437.1 435.12 434.14 433.12 432.13 431.11 430.1 429.09<br>428.12 427.11 426.11 425.1 424.09 423.12 419.12 417.13 415.11<br>414.11 413.1 411.12 410.11 409.1 408.1 407.09 403.11 401.1<br>400.09 399.08 398.11 397.1 396.1 395.09 389.1 385.1 384.1<br>383.09 382.08 381.07 376.14 374.12 373.1 371.09 369.07 368.1<br>367.09 366.08 362.12 357.11 355.09 354.08 353.08 343.09 342.08<br>341.08 339.1 338.09 337.08 336.11 329.11 327.1 326.09 325.08<br>324.07 323.07 318.1 317.08 316.07 315.1 313.12 312.11 311.1<br>309.09 307.07 303.1 301.08 300.09 299.1 297.09 295.11 294.1<br>293.09 287.1 286.09 285.09 283.11 281.09 276.09 271.08 270.07<br>255.09 253.07 235.07 222.06 194.04 180.06 179.06 178.05 177.04<br>176.03 175.02 174.05 168.03 165.08 164.07 163.06 162.05 161.04<br>160.04 159.03 152.03 151.06 150.02 149.05 148.04 147.07 146.06<br>145.05 144.04 143.03 139.06 137.08 136.08 135.07 134.06 133.05<br>132.04 131.03 130.03 129.05 128.03 127.04 126.02 121.05 120.04<br>119.03 118.03 117.02 116.05 115.04 114.02 113.02 110.06 109.03<br>108.02 107.07 105.05 104.05 103.04 102.03 101.02 100.04
**References**	Amaya-CruzIz, D.M.; Pérez-Ramírez, I.F.; Delgado-García, J.; Mondragón-Jacobo, C.; Dector-Espinoza, A.; Reynoso-Camacho, R. An integral profile of bioactive compounds and functional properties of prickly pear (*Opuntia ficus indica* L.) peel with different tonalities. *Food Chem.* **2019**, 278, 568–578.<br>Xie, G.R.; Chen, H.J. Comprehensive Betalain Profiling of Djulis (*Chenopodium formosanum*) Cultivars Using HPLC-Q-Orbitrap High-Resolution Mass Spectrometry. *J. Agric. Food Chem.* **2021**, 69, 15699–15715.<br>Slimen, I.B.; Najar, T.; Abderrabba, M. Chemical and Antioxidant Properties of Betalains. *J. Agric. Food Chem.* **2017**, 65, 675–689.<br>Kumorkiewicz-Jamro, A.; Świergosz, T.; Sutor, K.; Spórna-Kucab, A.; Wybraniec, S. Multi-colored shades of betalains: Recent advances in betacyanin chemistry. *Nat. Prod. Rep.* **2021**, 38, 2315–2346.

17-decarboxy-betanin

**Chemical structure**	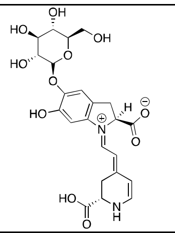
**Name:**	17-decarboxy-betanin
**Chemical Formula**	$C_{23}H_{26}N_{2}O_{11}$
**Molecular weight**	506.46
**Monoisotopic mass**	506.1537
***m/z* [M+H]**	507.1609
**Theorical fragments (*m/z*)**	507.16 506.15 505.15 504.14 491.17 490.16 489.15 488.14 487.13 479.17<br>478.16 477.15 475.17 474.16 473.16 472.15 471.14 470.13 469.12<br>467.17 465.15 464.14 463.17 462.16 461.16 460.15 459.14 457.16<br>456.15 455.14 454.14 453.13 451.11 449.16 447.18 446.17 445.16<br>444.15 443.14 442.14 441.13 439.15 437.16 435.14 434.13 433.16<br>432.15 431.14 429.17 428.16 427.15 426.14 425.13 421.16 419.14<br>418.14 417.13 416.12 415.15 414.14 413.13 403.15 401.13 399.12<br>385.14 383.12 382.11 381.11 375.12 373.1 369.11 368.1 366.12<br>364.1 358.11 357.11 356.1 355.09 352.1 351.09 345.11 344.1<br>343.09 342.09 341.09 340.1 339.09 338.09 337.08 331.13 330.12<br>329.11 328.11 327.1 326.09 325.09 324.08 323.08 322.09 320.08<br>317.11 316.11 315.13 313.12 312.11 311.1 310.09 309.09 307.08<br>306.07 305.11 303.1 301.12 300.11 299.14 298.09 297.12 296.09<br>295.11 294.1 293.09 291.1 288.11 287.1 286.09 285.1 283.11<br>282.1 281.09 280.09 279.09 275.1 273.1 272.08 271.08 270.07<br>269.09 268.09 267.09 259.11 257.09 256.08 255.09 254.08 253.07<br>252.06 250.08 249.08 243.11 241.1 240.09 239.08 238.07 237.09<br>236.08 235.06 227.12 225.1 223.07 221.09 220.08 219.08 218.07<br>209.09 207.08 205.06 203.08 201.07 196.06 195.05 194.04 193.1<br>192.03 191.08 189.07 187.05 182.08 181.05 180.04 179.08 178.05<br>177.04 167.08 166.09 165.05 164.05 163.09 161.07 152.07 151.09<br>150.05 149.07 147.04 146.04 145.05 136.08 135.04 134.04 132.04<br>130.04 124.04 123.04 119.05 111.04 110.06 109.03 108.02 107.05<br>106.04
**References**	Amaya-CruzIz, D.M.; Pérez-Ramírez, I.F.; Delgado-García, J.; Mondragón-Jacobo, C.; Dector-Espinoza, A.; Reynoso-Camacho, R. An integral profile of bioactive compounds and functional properties of prickly pear (*Opuntia ficus indica* L.) peel with different tonalities. *Food Chem.* **2019**, 278, 568–578.<br>Xie, G.R.; Chen, H.J. Comprehensive Betalain Profiling of Djulis (*Chenopodium formosanum*) Cultivars Using HPLC-Q-Orbitrap High-Resolution Mass Spectrometry. *J. Agric. Food Chem.* **2021**, 69, 15699–15715.<br>Slimen, I.B.; Najar, T.; Abderrabba, M. Chemical and Antioxidant Properties of Betalains. *J. Agric. Food Chem.* **2017**, 65, 675–689.<br>Kumorkiewicz-Jamro, A.; Świergosz, T.; Sutor, K.; Spórna-Kucab, A.; Wybraniec, S. Multi-colored shades of betalains: Recent advances in betacyanin chemistry. *Nat. Prod. Rep.* **2021**, 38, 2315–2346.

2-decarboxy-2,3-dehydro-isobetanin

Chemical structure	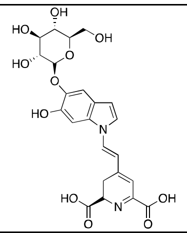
**Name:**	2-decarboxy-2,3-dehydro-isobetanin (2-decarboxy-isoxanbetanin)
**Chemical Formula**	$C_{23}H_{24}N_2O_{11}$
**Molecular weight**	504.45
**Monoisotopic mass**	504.1380
***m/z* [M+H]**	505.1453
**Theorical fragments (*m/z*)**	505.15 504.14 503.13 502.12 501.11 500.11 489.15 488.14 487.13 486.13<br>485.12 484.11 483.1 479.13 478.12 477.15 475.13 474.13 473.12<br>472.11 471.14 470.13 469.12 468.12 467.11 465.15 463.13 461.16<br>460.11 459.14 458.1 457.12 456.12 455.11 454.1 453.13 451.11<br>449.12 447.14 445.16 444.12 443.14 442.1 441.13 440.12 439.11<br>438.11 437.1 435.12 434.14 433.12 432.13 431.11 430.1 429.09<br>428.12 427.11 426.11 425.1 424.09 423.12 419.12 417.13 415.11<br>414.11 413.1 411.12 410.11 409.1 408.1 407.09 403.11 401.1<br>400.09 399.08 398.11 397.1 396.1 395.09 389.1 385.1 384.1<br>383.09 382.08 381.07 376.14 374.12 373.1 371.09 369.07 368.1<br>367.09 366.08 362.12 357.11 355.09 354.08 353.08 343.09 342.08<br>341.08 339.1 338.09 337.08 336.11 329.11 327.1 326.09 325.08<br>324.07 323.07 318.1 317.08 316.07 315.1 313.12 312.11 311.1<br>309.09 307.07 303.1 301.08 300.09 299.1 297.09 295.11 294.1<br>293.09 287.1 286.09 285.09 283.11 281.09 276.09 271.08 270.07<br>255.09 253.07 235.07 222.06 194.04 180.06 179.06 178.05 177.04<br>176.03 175.02 174.05 168.03 165.08 164.07 163.06 162.05 161.04<br>160.04 159.03 152.03 151.06 150.02 149.05 148.04 147.07 146.06<br>145.05 144.04 143.03 139.06 137.08 136.08 135.07 134.06 133.05<br>132.04 131.03 130.03 129.05 128.03 127.04 126.02 121.05 120.04<br>119.03 118.03 117.02 116.05 115.04 114.02 113.02 110.06 109.03<br>108.02 107.07 105.05 104.05 103.04 102.03 101.02 100.04
**References**	Amaya-CruzIz, D.M.; Pérez-Ramírez, I.F.; Delgado-García, J.; Mondragón-Jacobo, C.; Dector-Espinoza, A.; Reynoso-Camacho, R. An integral profile of bioactive compounds and functional properties of prickly pear (*Opuntia ficus indica* L.) peel with different tonalities. *Food Chem.* **2019**, 278, 568–578.<br>Xie, G.R.; Chen, H.J. Comprehensive Betalain Profiling of Djulis (*Chenopodium formosanum*) Cultivars Using HPLC-Q-Orbitrap High-Resolution Mass Spectrometry. *J. Agric. Food Chem.* **2021**, 69, 15699–15715.<br>Slimen, I.B.; Najar, T.; Abderrabba, M. Chemical and Antioxidant Properties of Betalains. *J. Agric. Food Chem.* **2017**, 65, 675–689.<br>Kumorkiewicz-Jamro, A.; Świergosz, T.; Sutor, K.; Spórna-Kucab, A.; Wybraniec, S. Multi-colored shades of betalains: Recent advances in betacyanin chemistry. *Nat. Prod. Rep.* **2021**, 38, 2315–2346.

2,17-bidecarboxy-2,3-dehydro-isobetanin

**Chemical structure**	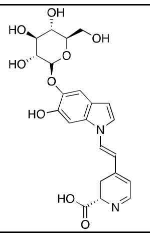
**Name:**	2,17-bidecarboxy-2,3-dehydro-betanin (2,17-bidecarboxy-xanbetanin)
**Chemical Formula**	$C_{22}H_{24}N_2O_9$
**Molecular weight**	460.44
**Monoisotopic mass**	460.1482
***m/z* [M+H]**	461.1555
**Theorical fragments (*m/z*)**	461.16 460.15 459.14 458.13 457.12 456.12 445.16 444.15 443.14 442.14 441.13 440.12 439.11 435.14 434.14 433.16 431.14 430.14 429.13 428.12 427.15 426.14 425.13 424.13 423.12 421.16 420.13 419.14 417.17 416.12 415.15 414.11 413.13 412.13 411.12 410.11 409.14 407.12 405.13 403.15 401.13 400.13 399.12 398.11 397.14 396.13 395.12 394.12 393.11 391.13 389.13 388.13 387.12 386.11 385.1 384.13 383.12 382.12 381.11 380.1 379.13 376.14 375.12 373.14 371.12 370.12 369.11 367.13 366.12 365.11 364.11 363.1 362.12 359.12 358.12 357.11 356.1 355.09 354.12 353.11 352.11 351.1 350.12 345.11 341.11 340.11 339.1 338.09 337.08 336.11 329.11 327.1 326.09 325.08 324.11 323.1 322.09 318.1 313.12 312.11 311.1 310.09 309.09 300.09 299.1 298.09 297.09 295.11 294.1 293.09 292.08 286.09 285.12 283.11 282.1 281.09 280.08 279.08 276.09 273.09 272.08 271.08 270.07 269.13 267.11 265.1 263.08 259.11 257.09 256.08 255.11 253.1 243.11 241.1 227.08 191.08 180.06 179.06 178.07 177.04 175.02 174.05 165.08 164.07 163.06 162.05 161.04 160.04 159.03 151.06 150.05 149.05 148.04 147.07 146.06 145.05 144.04 143.03 139.06 137.08 136.08 135.07 134.06 133.05 132.04 131.03 130.03 129.05 127.04 124.04 121.05 120.04 119.03 118.03 117.02 116.05 115.04 114.03 113.02 110.06 109.03 108.04 107.07 106.03 105.05 104.05 103.04 102.03 101.02 100.02
**References**	Amaya-CruzIz, D.M.; Pérez-Ramírez, I.F.; Delgado-García, J.; Mondragón-Jacobo, C.; Dector-Espinoza, A.; Reynoso-Camacho, R. An integral profile of bioactive compounds and functional properties of prickly pear (*Opuntia ficus indica* L.) peel with different tonalities. *Food Chem.* **2019**, 278, 568–578.<br>Xie, G.R.; Chen, H.J. Comprehensive Betalain Profiling of Djulis (*Chenopodium formosanum*) Cultivars Using HPLC-Q-Orbitrap High-Resolution Mass Spectrometry. *J. Agric. Food Chem.* **2021**, 69, 15699–15715.<br>Slimen, I.B.; Najar, T.; Abderrabba, M. Chemical and Antioxidant Properties of Betalains. *J. Agric. Food Chem.* **2017**, 65, 675–689.<br>Kumorkiewicz-Jamro, A.; Świergosz, T.; Sutor, K.; Spórna-Kucab, A.; Wybraniec, S. Multi-colored shades of betalains: Recent advances in betacyanin chemistry. *Nat. Prod. Rep.* **2021**, 38, 2315–2346.

17-decarboxy-isobetanin

**Chemical structure**	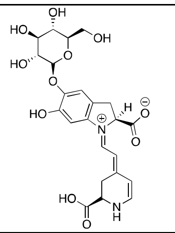
**Name:**	17-decarboxy-isobetanin
**Chemical Formula**	$C_{23}H_{26}N_2O_{11}$
**Molecular weight**	506.46
**Monoisotopic mass**	506.1537
***m/z* [M+H]**	507.1609
**Theorical fragments (*m/z*)**	507.16 506.15 505.15 504.14 491.17 490.16 489.15 488.14 487.13 479.17 478.16 477.15 475.17 474.16 473.16 472.15 471.14 470.13 469.12 467.17 465.15 464.14 463.17 462.16 461.16 460.15 459.14 457.16 456.15 455.14 454.14 453.13 451.11 449.16 447.18 446.17 445.16 444.15 443.14 442.14 441.13 439.15 437.16 435.14 434.13 433.16 432.15 431.14 429.17 428.16 427.15 426.14 425.13 421.16 419.14 418.14 417.13 416.12 415.15 414.14 413.13 403.15 401.13 399.12 385.14 383.12 382.11 381.11 375.12 373.1 369.11 368.1 366.12 364.1 358.11 357.11 356.1 355.09 352.1 351.09 345.11 344.1 343.09 342.09 341.09 340.1 339.09 338.09 337.08 331.13 330.12 329.11 328.11 327.1 326.09 325.09 324.08 323.08 322.09 320.08 317.11 316.11 315.13 313.12 312.11 311.1 310.09 309.09 307.08 306.07 305.11 303.1 301.12 300.11 299.14 298.09 297.12 296.09 295.11 294.1 293.09 291.1 288.11 287.1 286.09 285.1 283.11 282.1 281.09 280.09 279.09 275.1 273.1 272.08 271.08 270.07 269.09 268.09 267.09 259.11 257.09 256.08 255.09 254.08 253.07 252.06 250.08 249.08 243.11 241.1 240.09 239.08 238.07 237.09 236.08 235.06 227.12 225.1 223.07 221.09 220.08 219.08 218.07 209.09 207.08 205.06 203.08 201.07 196.06 195.05 194.04 193.1 192.03 191.08 189.07 187.05 182.08 181.05 180.04 179.08 178.05 177.04 167.08 166.09 165.05 164.05 163.09 161.07 152.07 151.09 150.05 149.07 147.04 146.04 145.05 136.08 135.04 134.04 132.04 130.04 124.04 123.04 119.05 111.04 110.06 109.03 108.02 107.05 106.04
**References**	Amaya-CruzIz, D.M.; Pérez-Ramírez, I.F.; Delgado-García, J.; Mondragón-Jacobo, C.; Dector-Espinoza, A.; Reynoso-Camacho, R. An integral profile of bioactive compounds and functional properties of prickly pear (*Opuntia ficus indica* L.) peel with different tonalities. *Food Chem.* **2019**, 278, 568–578.<br>Xie, G.R.; Chen, H.J. Comprehensive Betalain Profiling of Djulis (*Chenopodium formosanum*) Cultivars Using HPLC-Q-Orbitrap High-Resolution Mass Spectrometry. *J. Agric. Food Chem.* **2021**, 69, 15699–15715.<br>Slimen, I.B.; Najar, T.; Abderrabba, M. Chemical and Antioxidant Properties of Betalains. *J. Agric. Food Chem.* **2017**, 65, 675–689.<br>Kumorkiewicz-Jamro, A.; Świergosz, T.; Sutor, K.; Spórna-Kucab, A.; Wybraniec, S. Multi-colored shades of betalains: Recent advances in betacyanin chemistry. *Nat. Prod. Rep.* **2021**, 38, 2315–2346.

17-decarboxy-phyllocactin

**Chemical structure**	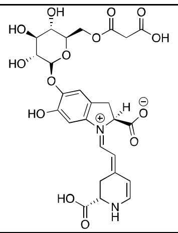
**Name:**	17-decarboxy-phyllocactin
**Chemical Formula**	$C_{26}H_{28}N_2O_{14}$
**Molecular weight**	592.51
**Monoisotopic mass**	592.1541
***m/z* [M+H]**	593.1613
**Theorical fragments (*m/z*)**	593.16 592.15 591.15 590.14 577.17 576.16 575.15 574.14 573.14 565.17<br>564.16 563.15 561.17 560.16 559.16 558.15 557.14 556.13 555.12<br>553.17 551.15 550.14 549.17 548.16 547.16 546.15 545.14 543.16<br>542.15 541.15 540.14 539.13 537.11 535.16 533.18 531.16 530.15<br>529.15 528.14 527.13 525.15 523.16 521.14 520.13 519.16 518.15<br>517.15 515.17 514.16 513.15 512.14 511.13 507.16 506.15 505.15<br>504.14 503.13 502.12 501.15 500.14 499.13 491.17 490.16 489.15<br>488.14 487.13 485.12 479.17 478.16 477.15 475.17 473.16 472.15<br>471.14 470.13 469.12 468.11 467.11 465.15 463.17 461.16 460.15<br>459.14 457.16 455.14 454.14 453.13 451.11 449.16 447.14 445.16<br>444.11 443.14 442.1 441.09 437.16 435.14 434.13 433.16 432.15<br>431.14 429.1 428.09 427.09 426.1 425.1 424.09 423.08 421.16<br>419.14 417.13 416.12 415.15 414.14 413.13 412.1 411.09 410.08<br>409.08 408.09 406.08 403.15 401.13 399.12 398.11 397.1 396.09<br>395.1 394.09 393.08 392.07 385.14 383.1 382.09 381.11 380.1<br>372.09 371.1 367.1 366.09 365.09 359.1 358.11 357.08 356.07<br>355.1 354.09 353.09 345.11 344.1 343.09 342.09 341.09 340.1<br>339.07 338.09 337.09 336.08 335.08 331.13 329.11 328.11 327.1<br>326.09 325.08 324.08 323.08 321.06 320.08 317.11 316.11 315.13<br>313.12 312.11 311.1 310.09 309.09 307.08 306.07 305.11 303.1<br>301.12 299.14 298.09 297.12 295.11 294.1 293.09 291.1 287.1<br>285.1 283.11 282.1 281.09 280.08 279.09 275.1 273.09 272.08<br>271.08 270.07 269.09 268.09 267.09 265.06 259.11 257.09 256.08<br>255.09 254.07 253.07 252.06 250.08 249.08 243.11 241.1 240.09<br>239.08 238.07 237.09 236.08 235.06 227.12 225.1 223.07 221.09<br>220.08 219.08 218.07 209.09 207.08 205.06 203.08 201.07 196.06<br>194.04 193.1 192.03 191.08 189.07 187.05 182.08 180.04 179.08<br>178.05 177.04 167.08 165.05 164.05 163.09 161.07 152.07 150.05<br>147.04 146.04 136.08 135.04 134.04 130.04 123.04 119.05 111.04<br>109.03 108.02 107.05 106.04
**References**	Amaya-CruzIz, D.M.; Pérez-Ramírez, I.F.; Delgado-García, J.; Mondragón-Jacobo, C.; Dector-Espinoza, A.; Reynoso-Camacho, R. An integral profile of bioactive compounds and functional properties of prickly pear (*Opuntia ficus indica* L.) peel with different tonalities. *Food Chem.* **2019**, 278, 568–578.<br>Xie, G.R.; Chen, H.J. Comprehensive Betalain Profiling of Djulis (*Chenopodium formosanum*) Cultivars Using HPLC-Q-Orbitrap High-Resolution Mass Spectrometry. *J. Agric. Food Chem.* **2021**, 69, 15699–15715.<br>Slimen, I.B.; Najar, T.; Abderrabba, M. Chemical and Antioxidant Properties of Betalains. *J. Agric. Food Chem.* **2017**, 65, 675–689.<br>Kumorkiewicz-Jamro, A.; Świergosz, T.; Sutor, K.; Spórna-Kucab, A.; Wybraniec, S. Multi-colored shades of betalains: Recent advances in betacyanin chemistry. *Nat. Prod. Rep.* **2021**, 38, 2315–2346.

15-decarboxy-betanin

**Chemical structure**	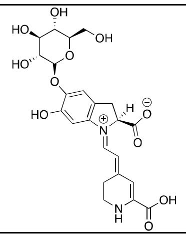
**Name:**	15-decarboxy-betanin
**Chemical Formula**	$C_{23}H_{26}N_2O_{11}$
**Molecular weight**	506.46
**Monoisotopic mass**	506.1537
***m/z* [M+H]**	507.1609
**Theorical fragments (*m/z*)**	507.16 506.15 505.15 504.14 492.15 491.17 490.16 489.15 488.14 487.13<br>479.17 478.16 477.15 475.17 473.16 472.15 471.14 470.13 469.12<br>467.17 465.15 464.14 463.17 462.16 461.16 460.15 459.14 455.14<br>454.14 453.13 451.11 449.16 448.15 447.14 445.16 444.15 443.14<br>442.14 441.13 437.16 435.14 434.13 432.15 431.14 429.17 427.15<br>426.14 425.13 419.14 418.14 417.13 416.12 414.14 413.13 409.14<br>408.13 401.13 399.12 396.13 383.12 382.11 381.11 375.12 373.1<br>369.11 368.1 366.12 364.1 358.11 357.11 356.1 355.09 352.1<br>351.09 348.11 346.09 345.11 344.1 343.09 342.09 341.09 340.1<br>339.09 338.09 337.08 334.09 331.13 330.12 329.11 328.11 327.1<br>326.09 325.09 324.08 323.08 322.09 321.08 320.08 319.07 317.11<br>316.11 315.13 314.13 313.12 312.11 311.1 310.09 307.08 306.07<br>305.11 303.1 302.07 301.12 300.11 299.1 298.09 297.1 296.09<br>295.11 294.1 293.09 291.1 288.11 287.1 286.09 285.1 284.12<br>283.11 282.1 281.09 280.09 279.09 276.09 275.1 273.1 272.08<br>271.08 270.07 269.13 268.09 267.09 259.11 257.09 256.08 255.09<br>254.08 253.07 252.06 250.08 249.08 243.11 241.1 240.09 237.09<br>236.08 235.06 223.07 221.09 220.06 219.08 209.09 207.08 205.06<br>204.07 202.05 196.06 195.05 194.04 193.1 192.03 191.08 190.05<br>189.07 182.08 181.05 180.04 179.08 178.05 177.04 176.03 167.08<br>166.09 165.05 164.05 163.04 162.05 152.07 150.05 149.05 148.04<br>147.04 146.04 145.05 136.08 135.04 134.04 132.04 130.04 124.04<br>123.04 119.05 111.04 110.06 109.03 108.02 107.05 106.04
**References**	Amaya-CruzIz, D.M.; Pérez-Ramírez, I.F.; Delgado-García, J.; Mondragón-Jacobo, C.; Dector-Espinoza, A.; Reynoso-Camacho, R. An integral profile of bioactive compounds and functional properties of prickly pear (*Opuntia ficus indica* L.) peel with different tonalities. *Food Chem.* **2019**, 278, 568–578.<br>Xie, G.R.; Chen, H.J. Comprehensive Betalain Profiling of Djulis (*Chenopodium formosanum*) Cultivars Using HPLC-Q-Orbitrap High-Resolution Mass Spectrometry. *J. Agric. Food Chem.* **2021**, 69, 15699–15715.<br>Slimen, I.B.; Najar, T.; Abderrabba, M. Chemical and Antioxidant Properties of Betalains. *J. Agric. Food Chem.* **2017**, 65, 675–689.<br>Kumorkiewicz-Jamro, A.; Świergosz, T.; Sutor, K.; Spórna-Kucab, A.; Wybraniec, S. Multi-colored shades of betalains: Recent advances in betacyanin chemistry. *Nat. Prod. Rep.* **2021**, 38, 2315–2346.

17-decarboxy-hylocerenin

**Chemical structure**	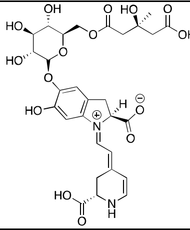
**Name:**	17-decarboxy-hylocerenin
**Chemical Formula**	$C_{29}H_{34}N_2O_{15}$
**Molecular weight**	650.59
**Monoisotopic mass**	650.1959
***m/z* [M+H]**	651.2032
**heorical fragments (*m/z*)**	651.2 650.2 649.19 648.18 635.21 634.2 633.19 632.18 631.18 623.21<br>622.2 621.19 619.21 618.21 617.2 616.19 615.18 614.17 613.17<br>611.21 609.19 608.18 607.21 606.21 605.2 604.19 603.18 601.2<br>600.19 599.19 598.18 597.17 595.16 593.2 591.22 589.2 588.19<br>587.19 586.18 585.17 583.19 581.2 579.18 578.17 577.2 576.19<br>575.19 573.21 572.2 571.19 570.18 569.18 565.2 563.19 562.18<br>561.17 560.16 559.19 558.18 557.18 549.21 547.19 545.18 543.16<br>531.2 529.18 527.17 526.16 525.15 513.15 507.16 506.15 505.15<br>502.16 500.14 499.13 491.17 490.16 489.15 488.14 487.13 486.14<br>485.13 484.14 483.14 482.13 481.12 479.17 478.16 477.15 475.17<br>473.16 472.15 471.14 470.14 469.13 468.13 467.12 465.15 463.17<br>461.16 460.15 459.14 457.16 456.15 455.14 454.13 453.13 452.13<br>451.12 450.12 449.16 447.14 445.16 444.15 443.14 442.14 441.14<br>440.13 437.16 435.14 434.13 433.16 432.15 431.14 430.13 429.14<br>427.15 426.14 425.14 424.14 423.13 421.16 419.14 417.14 416.12<br>415.12 414.12 413.14 412.14 411.13 403.15 401.14 399.13 398.12<br>397.11 396.11 395.13 394.13 393.12 385.15 383.13 381.12 379.1<br>358.11 356.1 355.09 345.11 344.1 343.09 342.09 341.09 340.1<br>338.09 337.08 331.13 329.11 328.11 327.1 326.09 325.08 324.08<br>323.08 317.11 316.11 315.13 313.12 312.11 311.1 310.09 309.09<br>307.08 306.07 305.11 303.1 301.12 299.14 298.09 297.12 295.11<br>293.09 291.1 287.1 285.1 283.11 282.1 281.09 280.08 279.09<br>275.1 273.09 272.08 271.08 270.07 269.09 268.09 267.09 259.11<br>257.09 256.08 255.09 254.07 253.07 252.06 250.08 249.08 243.11<br>241.1 240.09 239.08 238.07 237.09 236.08 235.06 227.12 225.1<br>223.07 221.09 220.08 219.08 218.07 209.09 207.08 205.06 203.08<br>201.07 196.06 194.04 193.1 191.08 189.07 187.05 182.08 180.04<br>179.08 178.05 177.04 167.08 165.05 164.05 163.09 161.07 152.07<br>150.05 147.04 146.04 136.08 135.04 134.04 130.04 123.04 119.05<br>111.04 109.03 108.02 107.05 106.04
**References**	Amaya-CruzIz, D.M.; Pérez-Ramírez, I.F.; Delgado-García, J.; Mondragón-Jacobo, C.; Dector-Espinoza, A.; Reynoso-Camacho, R. An integral profile of bioactive compounds and functional properties of prickly pear (*Opuntia ficus indica* L.) peel with different tonalities. *Food Chem.* **2019**, 278, 568–578.<br>Xie, G.R.; Chen, H.J. Comprehensive Betalain Profiling of Djulis (*Chenopodium formosanum*) Cultivars Using HPLC-Q-Orbitrap High-Resolution Mass Spectrometry. *J. Agric. Food Chem.* **2021**, 69, 15699–15715.<br>Slimen, I.B.; Najar, T.; Abderrabba, M. Chemical and Antioxidant Properties of Betalains. *J. Agric. Food Chem.* **2017**, 65, 675–689.<br>Kumorkiewicz-Jamro, A.; Świergosz, T.; Sutor, K.; Spórna-Kucab, A.; Wybraniec, S. Multi-colored shades of betalains: Recent advances in betacyanin chemistry. *Nat. Prod. Rep.* **2021**, 38, 2315–2346.

2-decarboxy-betanin

**Chemical structure**	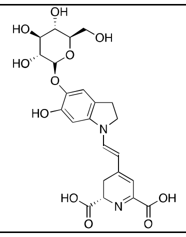
**Name:**	2-decarboxy-betanin
**Chemical Formula**	$C_{23}H_{26}N_2O_{11}$
**Molecular weight**	506.46
**Monoisotopic mass**	506.1537
***m/z* [M+H]**	507.1609
**Theorical fragments (*m/z*)**	507.16 506.15 505.15 504.14 503.13 502.12 493.15 492.14 491.17 490.16 489.15 488.14 487.13 486.13 485.12 481.15 479.17 478.12 477.15 476.14 475.13 474.13 473.16 472.15 471.14 470.13 469.12 467.17 465.15 463.17 462.13 461.16 460.15 459.14 458.13 457.12 456.12 455.14 453.13 451.13 449.16 447.18 446.13 445.16 444.12 443.14 441.13 439.11 437.13 436.16 435.14 434.13 433.12 432.12 431.11 430.14 429.13 428.12 427.11 426.11 425.13 421.14 419.14 417.13 416.12 415.11 413.13 412.13 411.12 410.11 409.1 405.13 403.11 402.11 401.1 400.13 399.12 398.11 397.1 391.11 387.12 386.11 385.1 384.1 383.09 376.14 375.12 373.1 371.09 370.12 369.11 368.1 364.14 359.12 357.11 356.1 355.09 345.11 344.1 343.09 341.11 340.11 339.1 338.12 331.13 329.11 328.11 327.1 326.09 325.08 320.11 319.09 317.08 316.07 315.13 314.12 313.12 312.11 311.1 310.09 309.09 303.1 302.1 301.12 300.11 299.1 297.1 296.11 294.1 288.11 287.1 286.09 285.1 283.11 282.1 279.09 278.1 276.09 273.09 271.08 270.07 268.09 267.09 255.11 237.09 236.08 235.07 223.07 222.06 211.07 207.08 205.06 196.06 194.04 180.06 179.06 178.05 177.04 176.07 175.02 168.03 165.08 164.07 163.06 162.05 161.04 160.04 159.03 152.03 151.06 150.02 149.04 148.04 147.07 146.06 145.05 144.04 143.03 139.06 138.09 137.08 136.08 135.04 134.06 133.05 132.04 131.03 130.03 129.05 127.04 124.04 123.04 122.06 121.05 120.04 119.03 118.03 117.02 116.05 115.04 113.02 111.04 110.06 108.04 107.07 105.05 104.05 103.04 102.03 101.02 100.04
**References**	Amaya-CruzIz, D.M.; Pérez-Ramírez, I.F.; Delgado-García, J.; Mondragón-Jacobo, C.; Dector-Espinoza, A.; Reynoso-Camacho, R. An integral profile of bioactive compounds and functional properties of prickly pear (*Opuntia ficus indica* L.) peel with different tonalities. *Food Chem.* **2019**, 278, 568–578.<br>Xie, G.R.; Chen, H.J. Comprehensive Betalain Profiling of Djulis (*Chenopodium formosanum*) Cultivars Using HPLC-Q-Orbitrap High-Resolution Mass Spectrometry. *J. Agric. Food Chem.* **2021**, 69, 15699–15715.<br>Slimen, I.B.; Najar, T.; Abderrabba, M. Chemical and Antioxidant Properties of Betalains. *J. Agric. Food Chem.* **2017**, 65, 675–689.<br>Kumorkiewicz-Jamro, A.; Świergosz, T.; Sutor, K.; Spórna-Kucab, A.; Wybraniec, S. Multi-colored shades of betalains: Recent advances in betacyanin chemistry. *Nat. Prod. Rep.* **2021**, 38, 2315–2346.

17-decarboxy-isophyllocactin

**Chemical structure**	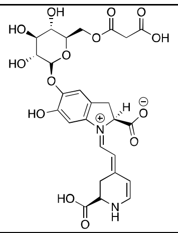
**Name:**	17-decarboxy-isophyllocactin
**Chemical Formula**	$C_{26}H_{28}N_2O_{14}$
**Molecular weight**	592.51
**Monoisotopic mass**	592.1541
***m/z* [M+H]**	593.1613
**Theorical fragments (*m/z*)**	593.16 592.15 591.15 590.14 577.17 576.16 575.15 574.14 573.14 565.17<br>564.16 563.15 561.17 560.16 559.16 558.15 557.14 556.13 555.12<br>553.17 551.15 550.14 549.17 548.16 547.16 546.15 545.14 543.16<br>542.15 541.15 540.14 539.13 537.11 535.16 533.18 531.16 530.15<br>529.15 528.14 527.13 525.15 523.16 521.14 520.13 519.16 518.15<br>517.15 515.17 514.16 513.15 512.14 511.13 507.16 506.15 505.15<br>504.14 503.13 502.12 501.15 500.14 499.13 491.17 490.16 489.15<br>488.14 487.13 485.12 479.17 478.16 477.15 475.17 473.16 472.15<br>471.14 470.13 469.12 468.11 467.11 465.15 463.17 461.16 460.15<br>459.14 457.16 455.14 454.14 453.13 451.11 449.16 447.14 445.16<br>444.11 443.14 442.1 441.09 437.16 435.14 434.13 433.16 432.15<br>431.14 429.1 428.09 427.09 426.1 425.1 424.09 423.08 421.16<br>419.14 417.13 416.12 415.15 414.14 413.13 412.1 411.09 410.08<br>409.08 408.09 406.08 403.15 401.13 399.12 398.11 397.1 396.09<br>395.1 394.09 393.08 392.07 385.14 383.1 382.09 381.11 380.1<br>372.09 371.1 367.1 366.09 365.09 359.1 358.11 357.08 356.07<br>355.1 354.09 353.09 345.11 344.1 343.09 342.09 341.09 340.1<br>339.07 338.09 337.09 336.08 335.08 331.13 329.11 328.11 327.1<br>326.09 325.08 324.08 323.08 321.06 320.08 317.11 316.11 315.13<br>313.12 312.11 311.1 310.09 309.09 307.08 306.07 305.11 303.1<br>301.12 299.14 298.09 297.12 295.11 294.1 293.09 291.1 287.1<br>285.1 283.11 282.1 281.09 280.08 279.09 275.1 273.09 272.08<br>271.08 270.07 269.09 268.09 267.09 265.06 259.11 257.09 256.08<br>255.09 254.07 253.07 252.06 250.08 249.08 243.11 241.1 240.09<br>239.08 238.07 237.09 236.08 235.06 227.12 225.1 223.07 221.09<br>220.08 219.08 218.07 209.09 207.08 205.06 203.08 201.07 196.06<br>194.04 193.1 192.03 191.08 189.07 187.05 182.08 180.04 179.08<br>178.05 177.04 167.08 165.05 164.05 163.09 161.07 152.07 150.05<br>147.04 146.04 136.08 135.04 134.04 130.04 123.04 119.05 111.04<br>109.03 108.02 107.05 106.04
**References**	Amaya-CruzIz, D.M.; Pérez-Ramírez, I.F.; Delgado-García, J.; Mondragón-Jacobo, C.; Dector-Espinoza, A.; Reynoso-Camacho, R. An integral profile of bioactive compounds and functional properties of prickly pear (*Opuntia ficus indica* L.) peel with different tonalities. *Food Chem.* **2019**, 278, 568–578.<br>Xie, G.R.; Chen, H.J. Comprehensive Betalain Profiling of Djulis (*Chenopodium formosanum*) Cultivars Using HPLC-Q-Orbitrap High-Resolution Mass Spectrometry. *J. Agric. Food Chem.* **2021**, 69, 15699–15715.<br>Slimen, I.B.; Najar, T.; Abderrabba, M. Chemical and Antioxidant Properties of Betalains. *J. Agric. Food Chem.* **2017**, 65, 675–689.<br>Kumorkiewicz-Jamro, A.; Świergosz, T.; Sutor, K.; Spórna-Kucab, A.; Wybraniec, S. Multi-colored shades of betalains: Recent advances in betacyanin chemistry. *Nat. Prod. Rep.* **2021**, 38, 2315–2346.

17-decarboxy-isohylocerenin

**Chemical structure**	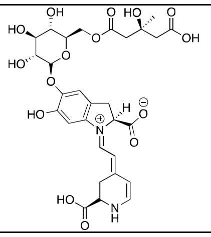
**Name:**	17-decarboxy-isohylocerenin
**Chemical Formula**	$C_{29}H_{34}N_2O_{15}$
**Molecular weight**	650.59
**Monoisotopic mass**	650.1959
***m/z* [M+H]**	651.2032
**Theorical fragments (*m/z*)**	651.2 650.2 649.19 648.18 635.21 634.2 633.19 632.18 631.18 623.21<br>622.2 621.19 619.21 618.21 617.2 616.19 615.18 614.17<br>613.17 611.21 609.19 608.18 607.21 606.21 605.2 604.19<br>603.18 601.2 600.19 599.19 598.18 597.17 595.16 593.2<br>591.22 589.2 588.19 587.19 586.18 585.17 583.19 581.2<br>579.18 578.17 577.2 576.19 575.19 573.21 572.2 571.19<br>570.18 569.18 565.2 563.19 562.18 561.17 560.16 559.19<br>558.18 557.18 549.21 547.19 545.18 543.16 531.2 529.18<br>527.17 526.16 525.15 513.15 507.16 506.15 505.15 502.16<br>500.14 499.13 491.17 490.16 489.15 488.14 487.13 486.14<br>485.13 484.14 483.14 482.13 481.12 479.17 478.16 477.15<br>475.17 473.16 472.15 471.14 470.14 469.13 468.13 467.12<br>465.15 463.17 461.16 460.15 459.14 457.16 456.15 455.14<br>454.13 453.13 452.13 451.12 450.12 449.16 447.14 445.16<br>444.15 443.14 442.14 441.14 440.13 437.16 435.14 434.13<br>433.16 432.15 431.14 430.13 429.14 427.15 426.14 425.14<br>424.14 423.13 421.16 419.14 417.14 416.12 415.12 414.12<br>413.14 412.14 411.13 403.15 401.14 399.13 398.12 397.11<br>396.11 395.13 394.13 393.12 385.15 383.13 381.12 379.1<br>358.11 356.1 355.09 345.11 344.1 343.09 342.09 341.09<br>340.1 338.09 337.08 331.13 329.11 328.11 327.1 326.09<br>325.08 324.08 323.08 317.11 316.11 315.13 313.12 312.11<br>311.1 310.09 309.09 307.08 306.07 305.11 303.1 301.12<br>299.14 298.09 297.12 295.11 293.09 291.1 287.1 285.1<br>283.11 282.1 281.09 280.08 279.09 275.1 273.09 272.08<br>271.08 270.07 269.09 268.09 267.09 259.11 257.09 256.08<br>255.09 254.07 253.07 252.06 250.08 249.08 243.11 241.1<br>240.09 239.08 238.07 237.09 236.08 235.06 227.12 225.1<br>223.07 221.09 220.08 219.08 218.07 209.09 207.08 205.06<br>203.08 201.07 196.06 194.04 193.1 191.08 189.07 187.05<br>182.08 180.04 179.08 178.05 177.04 167.08 165.05 164.05<br>163.09 161.07 152.07 150.05 147.04 146.04 136.08 135.04<br>134.04 130.04 123.04 119.05 111.04 109.03 108.02 107.05<br>106.04
**References**	Amaya-CruzIz, D.M.; Pérez-Ramírez, I.F.; Delgado-García, J.; Mondragón-Jacobo, C.; Dector-Espinoza, A.; Reynoso-Camacho, R. An integral profile of bioactive compounds and functional properties of prickly pear (*Opuntia ficus indica* L.) peel with different tonalities. *Food Chem.* **2019**, 278, 568–578.<br>Xie, G.R.; Chen, H.J. Comprehensive Betalain Profiling of Djulis (*Chenopodium formosanum*) Cultivars Using HPLC-Q-Orbitrap High-Resolution Mass Spectrometry. *J. Agric. Food Chem.* **2021**, 69, 15699–15715.<br>Slimen, I.B.; Najar, T.; Abderrabba, M. Chemical and Antioxidant Properties of Betalains. *J. Agric. Food Chem.* **2017**, 65, 675–689.<br>Kumorkiewicz-Jamro, A.; Świergosz, T.; Sutor, K.; Spórna-Kucab, A.; Wybraniec, S. Multi-colored shades of betalains: Recent advances in betacyanin chemistry. *Nat. Prod. Rep.* **2021**, 38, 2315–2346.

2-decarboxy-phyllocactin

**Chemical structure**	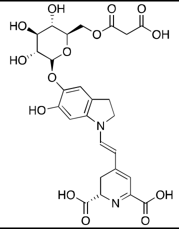
**Name:**	2-decarboxy-phyllocactin
**Chemical Formula**	$C_{26}H_{28}N_2O_{14}$
**Molecular weight**	592.51
**Monoisotopic mass**	592.1541
***m/z* [M+H]**	593.1613
**Theorical fragments (*m/z*)**	593.16 592.15 591.15 590.14 579.15 577.17 576.16 575.15 574.14 573.14<br>567.15 565.13 564.12 563.15 562.14 561.14 559.16 557.14<br>555.12 551.15 549.14 548.13 547.12 546.15 545.14 544.13<br>543.12 541.15 539.13 535.16 533.14 532.17 531.12 530.12<br>529.11 528.14 527.13 526.12 525.15 523.13 521.12 520.14<br>517.15 515.13 513.11 511.1 507.16 505.15 493.15 491.17<br>489.15 487.13 481.15 479.13 478.12 477.15 476.14 475.13<br>473.16 471.14 469.12 465.15 463.13 462.13 461.12 460.11<br>459.14 458.13 457.12 455.14 453.13 447.14 445.12 444.12<br>443.11 442.14 441.13 440.12 439.11 437.13 435.12 429.13<br>427.11 425.1 424.12 411.12 408.13 406.11 400.12 399.12<br>398.11 397.1 396.09 395.12 393.11 386.11 384.13 383.1<br>382.11 381.11 380.1 379.09 375.12 374.11 373.1 372.09<br>371.1 370.11 369.11 368.1 367.1 366.12 365.09 364.1<br>362.09 359.1 357.08 356.07 355.1 354.09 353.09 349.09<br>347.08 345.11 344.1 343.09 342.08 341.09 340.08 339.07<br>338.06 337.09 336.08 335.08 331.13 329.11 328.11 327.1<br>326.09 325.08 323.08 321.06 320.11 319.09 317.08 316.07<br>315.13 314.09 313.12 312.11 311.1 309.09 307.11 303.1<br>301.08 300.07 299.07 298.09 297.1 296.11 295.11 294.1<br>293.09 287.1 286.09 285.1 284.08 283.07 282.06 279.09<br>278.1 276.09 273.1 271.08 270.07 269.09 268.09 267.09<br>261.08 255.09 253.07 252.06 250.08 249.08 237.09 236.08<br>235.06 233.07 231.05 223.07 222.06 221.09 220.08 219.08<br>218.07 211.07 207.08 205.06 196.06 195.08 194.04 193.06<br>191.05 180.07 178.05 176.07 163.06 161.04 152.07 151.06<br>150.05 149.05 148.04 147.07 145.05 138.09 136.08 135.04<br>134.06 133.05 124.04 123.04 122.06 121.06 119.05 111.04<br>110.06 109.03 108.02 107.05 106.04 103.05
**References**	Amaya-CruzIz, D.M.; Pérez-Ramírez, I.F.; Delgado-García, J.; Mondragón-Jacobo, C.; Dector-Espinoza, A.; Reynoso-Camacho, R. An integral profile of bioactive compounds and functional properties of prickly pear (*Opuntia ficus indica* L.) peel with different tonalities. *Food Chem.* **2019**, 278, 568–578.<br>Xie, G.R.; Chen, H.J. Comprehensive Betalain Profiling of Djulis (*Chenopodium formosanum*) Cultivars Using HPLC-Q-Orbitrap High-Resolution Mass Spectrometry. *J. Agric. Food Chem.* **2021**, 69, 15699–15715.<br>Slimen, I.B.; Najar, T.; Abderrabba, M. Chemical and Antioxidant Properties of Betalains. *J. Agric. Food Chem.* **2017**, 65, 675–689.<br>Kumorkiewicz-Jamro, A.; Świergosz, T.; Sutor, K.; Spórna-Kucab, A.; Wybraniec, S. Multi-colored shades of betalains: Recent advances in betacyanin chemistry. *Nat. Prod. Rep.* **2021**, 38, 2315–2346.

2-decarboxy-isophyllocactin

**Chemical structure**	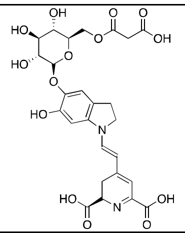
**Name:**	2-decarboxy-isophyllocactin
**Chemical Formula**	$C_{26}H_{28}N_2O_{14}$
**Molecular weight**	592.51
**Monoisotopic mass**	592.1541
***m/z* [M+H]**	593.1613
**Theorical fragments (*m/z*)**	593.16 592.15 591.15 590.14 579.15 577.17 576.16 575.15 574.14 573.14 567.15 565.13 564.12 563.15 562.14 561.14 559.16 557.14 555.12 551.15 549.14 548.13 547.12 546.15 545.14 544.13 543.12 541.15 539.13 535.16 533.14 532.17 531.12 530.12 529.11 528.14 527.13 526.12 525.15 523.13 521.12 520.14 517.15 515.13 513.11 511.1 507.16 505.15 493.15 491.17 489.15 487.13 481.15 479.13 478.12 477.15 476.14 475.13 473.16 471.14 469.12 465.15 463.13 462.13 461.12 460.11 459.14 458.13 457.12 455.14 453.13 447.14 445.12 444.12 443.11 442.14 441.13 440.12 439.11 437.13 435.12 429.13 427.11 425.1 424.12 411.12 408.13 406.11 400.12 399.12 398.11 397.1 396.09 395.12 393.11 386.11 384.13 383.1 382.11 381.11 380.1 379.09 375.12 374.11 373.1 372.09 371.1 370.11 369.11 368.1 367.1 366.12 365.09 364.1 362.09 359.1 357.08 356.07 355.1 354.09 353.09 349.09 347.08 345.11 344.1 343.09 342.08 341.09 340.08 339.07 338.06 337.09 336.08 335.08 331.13 329.11 328.11 327.1 326.09 325.08 323.08 321.06 320.11 319.09 317.08 316.07 315.13 314.09 313.12 312.11 311.1 309.09 307.11 303.1 301.08 300.07 299.07 298.09 297.1 296.11 295.11 294.1 293.09 287.1 286.09 285.1 284.08 283.07 282.06 279.09 278.1 276.09 273.1 271.08 270.07 269.09 268.09 267.09 261.08 255.09 253.07 252.06 250.08 249.08 237.09 236.08 235.06 233.07 231.05 223.07 222.06 221.09 220.08 219.08 218.07 211.07 207.08 205.06 196.06 195.08 194.04 193.06 191.05 180.07 178.05 176.07 163.06 161.04 152.07 151.06 150.05 149.05 148.04 147.07 145.05 138.09 136.08 135.04 134.06 133.05 124.04 123.04 122.06 121.06 119.05 111.04 110.06 109.03 108.02 107.05 106.04 103.05
**References**	Amaya-CruzIz, D.M.; Pérez-Ramírez, I.F.; Delgado-García, J.; Mondragón-Jacobo, C.; Dector-Espinoza, A.; Reynoso-Camacho, R. An integral profile of bioactive compounds and functional properties of prickly pear (*Opuntia ficus indica* L.) peel with different tonalities. *Food Chem.* **2019**, 278, 568–578.<br>Xie, G.R.; Chen, H.J. Comprehensive Betalain Profiling of Djulis (*Chenopodium formosanum*) Cultivars Using HPLC-Q-Orbitrap High-Resolution Mass Spectrometry. *J. Agric. Food Chem.* **2021**, 69, 15699–15715.<br>Slimen, I.B.; Najar, T.; Abderrabba, M. Chemical and Antioxidant Properties of Betalains. *J. Agric. Food Chem.* **2017**, 65, 675–689.<br>Kumorkiewicz-Jamro, A.; Świergosz, T.; Sutor, K.; Spórna-Kucab, A.; Wybraniec, S. Multi-colored shades of betalains: Recent advances in betacyanin chemistry. *Nat. Prod. Rep.* **2021**, 38, 2315–2346.

Neobetanin

**Chemical structure**	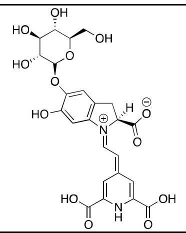
**Name:**	Neobetanin
**Chemical Formula**	$C_{24}H_{24}N_2O_{13}$
**Molecular weight**	548.46
**Monoisotopic mass**	548.1278
***m/z* [M+H]**	549.1351
**Theorical fragments (*m/z*)**	549.14 548.13 547.12 546.11 533.14 532.13 531.12 530.12 529.11 521.14 520.13 519.12 517.15 516.14 515.13 514.12 513.11 512.11 511.1 509.14 507.12 506.12 505.15 504.14 503.13 502.12 501.11 497.12 496.11 495.1 493.09 491.13 490.12 489.11 488.14 487.13 486.13 485.12 484.11 483.1 479.13 477.11 476.11 474.13 473.12 471.14 469.12 468.12 467.11 461.12 460.11 459.1 458.1 456.12 455.11 451.11 443.11 441.09 433.12 425.1 423.08 417.09 415.08 387.08 386.07 385.07 384.06 373.1 371.09 370.08 369.07 368.06 367.06 359.09 358.08 357.11 356.1 355.09 354.08 353.08 352.07 351.09 350.09 347.09 345.07 343.09 342.09 341.09 340.07 339.1 338.09 337.08 336.07 334.09 333.07 329.08 327.1 326.09 325.09 324.08 323.08 322.09 321.08 320.08 319.07 317.08 315.06 314.05 313.08 312.11 311.1 310.09 308.09 307.08 306.07 302.07 301.08 299.07 298.06 297.1 296.09 294.1 293.09 285.1 283.07 282.06 280.09 279.06 278.05 277.05 276.09 273.1 271.08 270.07 268.09 267.09 265.05 264.04 263.07 262.06 261.05 255.09 254.08 253.07 252.06 251.07 250.08 249.05 247.03 237.08 235.07 233.06 231.04 221.06 209.06 207.05 206.03 194.04 193.04 192.03 191.06 190.05 181.05 180.04 179.03 178.05 177.04 176.03 167.07 165.05 164.05 163.04 162.05 150.05 149.05 148.04 147.04 146.04 145.05 136.08 135.04 134.04 132.04 130.04 123.04 119.05 111.04 109.03 108.02 107.05 106.04
**References**	Amaya-CruzIz, D.M.; Pérez-Ramírez, I.F.; Delgado-García, J.; Mondragón-Jacobo, C.; Dector-Espinoza, A.; Reynoso-Camacho, R. An integral profile of bioactive compounds and functional properties of prickly pear (*Opuntia ficus indica* L.) peel with different tonalities. *Food Chem.* **2019**, 278, 568–578.<br>Xie, G.R.; Chen, H.J. Comprehensive Betalain Profiling of Djulis (*Chenopodium formosanum*) Cultivars Using HPLC-Q-Orbitrap High-Resolution Mass Spectrometry. *J. Agric. Food Chem.* **2021**, 69, 15699–15715.<br>Slimen, I.B.; Najar, T.; Abderrabba, M. Chemical and Antioxidant Properties of Betalains. *J. Agric. Food Chem.* **2017**, 65, 675–689.<br>Kumorkiewicz-Jamro, A.; Świergosz, T.; Sutor, K.; Spórna-Kucab, A.; Wybraniec, S. Multi-colored shades of betalains: Recent advances in betacyanin chemistry. *Nat. Prod. Rep.* **2021**, 38, 2315–2346.

17-decarboxy-neobetanin

**Chemical structure**	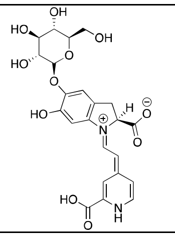
**Name:**	17-decarboxy-neobetanin
**Chemical Formula**	$C_{23}H_{24}N_2O_{11}$
**Molecular weight**	504.45
**Monoisotopic mass**	504.1380
***m/z* [M+H]**	505.1453
**Theorical fragments (*m/z*)**	505.15 504.14 503.13 502.12 501.11 500.11 491.13 490.12 489.15 488.14<br>487.13 486.13 485.12 484.11 483.1 479.13 477.15 476.14<br>475.13 474.13 473.12 472.11 471.14 470.13 469.12 468.12<br>467.11 465.15 463.13 461.16 460.15 459.14 458.13 457.12<br>456.12 455.11 454.1 453.13 452.12 451.11 449.12 448.15<br>447.14 446.13 445.16 444.12 443.14 442.1 441.13 439.11<br>437.1 435.14 433.12 432.12 431.11 430.14 429.09 428.12<br>427.11 426.11 425.1 424.09 423.12 419.11 417.13 415.11<br>414.11 413.1 411.12 410.11 409.1 408.1 403.11 401.1<br>400.09 399.08 398.11 397.1 396.1 395.09 389.1 385.1<br>384.1 383.09 382.11 381.07 373.1 371.09 369.07 368.1<br>367.09 366.12 364.1 358.11 357.11 356.1 355.09 354.08<br>353.08 348.11 346.09 343.09 342.08 341.08 340.1 339.1<br>338.09 337.08 336.07 330.12 329.11 328.1 327.1 326.09<br>325.08 324.07 323.07 322.09 320.08 315.1 313.12 312.11<br>311.1 310.09 309.09 308.08 307.07 301.12 300.11 299.1<br>298.09 297.09 296.08 295.07 294.1 288.11 286.09 285.1<br>283.11 281.09 279.08 273.1 271.07 270.07 269.09 268.09<br>267.09 257.09 255.08 254.07 241.1 239.08 235.07 234.06<br>233.06 221.06 220.06 219.08 217.06 207.08 205.06 204.07<br>203.05 202.05 196.06 195.05 194.04 193.06 192.05 191.05<br>182.08 180.07 179.06 178.05 177.04 176.06 175.02 166.09<br>165.08 164.07 163.06 162.05 161.04 160.04 159.03 151.06<br>150.05 149.04 148.04 147.07 146.06 145.05 144.04 143.03<br>139.06 136.04 135.07 134.06 133.05 132.04 131.03 130.03<br>129.05 127.04 124.04 123.04 122.02 121.05 120.04 119.03<br>118.03 117.02 116.05 115.04 113.02 111.04 110.06 108.04<br>107.07 105.05 104.05 103.04 102.03 101.02 100.02
**References**	Amaya-CruzIz, D.M.; Pérez-Ramírez, I.F.; Delgado-García, J.; Mondragón-Jacobo, C.; Dector-Espinoza, A.; Reynoso-Camacho, R. An integral profile of bioactive compounds and functional properties of prickly pear (*Opuntia ficus indica* L.) peel with different tonalities. *Food Chem.* **2019**, 278, 568–578.<br>Xie, G.R.; Chen, H.J. Comprehensive Betalain Profiling of Djulis (*Chenopodium formosanum*) Cultivars Using HPLC-Q-Orbitrap High-Resolution Mass Spectrometry. *J. Agric. Food Chem.* **2021**, 69, 15699–15715.<br>Slimen, I.B.; Najar, T.; Abderrabba, M. Chemical and Antioxidant Properties of Betalains. *J. Agric. Food Chem.* **2017**, 65, 675–689.<br>Kumorkiewicz-Jamro, A.; Świergosz, T.; Sutor, K.; Spórna-Kucab, A.; Wybraniec, S. Multi-colored shades of betalains: Recent advances in betacyanin chemistry. *Nat. Prod. Rep.* **2021**, 38, 2315–2346.

2-decarboxy-neobetanin

**Chemical structure**	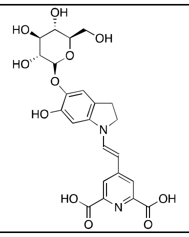
**Name:**	2-decarboxy-neobetanin
**Chemical Formula**	$C_{23}H_{24}N_2O_{11}$
**Molecular weight**	504.45
**Monoisotopic mass**	504.1380
***m/z* [M+H]**	505.1453
**Theorical fragments (*m/z*)**	505.15 504.14 503.13 502.12 491.13 490.12 489.15 488.14 487.13 486.13<br>485.12 479.13 477.11 476.11 474.13 473.12 472.15 471.14 470.13<br>469.12 468.12 467.11 461.12 460.11 459.1 458.1 457.12 456.12<br>455.11 454.14 453.13 452.12 451.11 450.11 447.14 444.15 443.11<br>442.14 441.09 438.11 437.1 435.14 434.11 433.12 432.12 431.11<br>430.14 429.13 426.14 425.1 424.13 423.08 417.17 416.16 415.15<br>413.1 408.13 407.12 399.16 398.15 397.14 373.1 371.09 355.09<br>354.08 343.09 342.08 341.08 340.07 329.11 328.11 327.1 326.09<br>325.08 324.07 323.1 317.08 315.06 314.05 313.08 312.11 311.1<br>310.09 307.11 306.1 303.1 301.08 299.07 298.06 297.1 296.11<br>294.1 293.09 285.1 283.07 282.06 281.1 280.08 279.09 278.1<br>276.09 275.08 273.1 271.08 270.07 269.09 268.09 267.09 261.08<br>258.08 257.07 255.09 254.08 253.07 252.06 251.08 250.08 249.08<br>237.08 235.07 234.06 233.06 221.06 220.05 209.06 208.05 207.04<br>206.03 204.05 194.04 193.04 192.05 180.03 178.05 177.07 176.06<br>175.05 165.05 163.06 162.05 161.04 160.06 150.05 149.05 148.04<br>147.07 145.05 143.03 136.08 135.04 134.06 133.05 132.04 127.04<br>123.04 121.06 120.08 119.05 118.07 111.04 109.03 108.02 107.05<br>106.04 103.05 101.02
**References**	Amaya-CruzIz, D.M.; Pérez-Ramírez, I.F.; Delgado-García, J.; Mondragón-Jacobo, C.; Dector-Espinoza, A.; Reynoso-Camacho, R. An integral profile of bioactive compounds and functional properties of prickly pear (*Opuntia ficus indica* L.) peel with different tonalities. *Food Chem.* **2019**, 278, 568–578.<br>Xie, G.R.; Chen, H.J. Comprehensive Betalain Profiling of Djulis (*Chenopodium formosanum*) Cultivars Using HPLC-Q-Orbitrap High-Resolution Mass Spectrometry. *J. Agric. Food Chem.* **2021**, 69, 15699–15715.<br>Slimen, I.B.; Najar, T.; Abderrabba, M. Chemical and Antioxidant Properties of Betalains. *J. Agric. Food Chem.* **2017**, 65, 675–689.<br>Kumorkiewicz-Jamro, A.; Świergosz, T.; Sutor, K.; Spórna-Kucab, A.; Wybraniec, S. Multi-colored shades of betalains: Recent advances in betacyanin chemistry. *Nat. Prod. Rep.* **2021**, 38, 2315–2346.

2-decarboxy-2,3-dehydro-neobetanin

**Chemical structure**	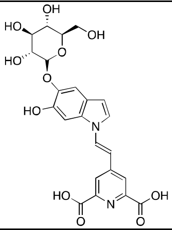
**Name:**	2-Decarboxy-2,3-dehydro-neobetanin
**Chemical Formula**	$C_{23}H_{22}N_2O_{11}$
**Molecular weight**	502.43
**Monoisotopic mass**	502.1224
***m/z* [M+H]**	503.1296
**Theorical fragments (*m/z*)**	503.13 502.12 487.13 486.13 485.12 484.11 477.11 476.11 475.13 470.13<br>469.12 468.12 467.11 466.1 461.12 460.11 459.14 458.13 457.12<br>455.11 452.12 451.11 450.11 449.1 448.09 444.12 443.11 442.1<br>441.13 440.12 439.11 437.1 434.11 433.12 432.1 431.11 428.09<br>426.11 425.1 424.13 423.12 422.11 421.1 417.13 416.12 415.15<br>414.14 413.1 412.09 411.12 406.12 405.11 404.1 403.09 399.08<br>397.14 396.13 395.12 389.13 388.11 387.12 379.13 378.12 377.11<br>370.08 369.07 361.12 353.08 341.08 340.07 339.06 327.1 326.09<br>325.08 324.07 323.07 315.06 314.05 313.08 310.09 309.09 308.08<br>307.07 301.08 300.07 299.07 298.06 297.09 296.08 295.07 294.1<br>292.08 285.09 284.08 283.07 282.06 281.09 280.08 279.08 277.06<br>271.08 270.07 268.08 266.1 265.1 262.08 257.09 256.08 255.09<br>254.08 253.07 252.06 251.08 241.1 240.09 239.12 238.11 237.08<br>235.06 234.05 233.06 220.05 216.04 213.1 209.06 208.05 207.04<br>206.03 203.05 194.04 193.04 192.05 190.04 189.07 180.03 176.03<br>175.05 166.01 165.07 163.06 162.04 150.02 148.04 147.07 146.06<br>136.04 134.06 132.04 122.02 119.05 118.04 109.03 108.02 107.05<br>106.03 102.05
**References**	Amaya-CruzIz, D.M.; Pérez-Ramírez, I.F.; Delgado-García, J.; Mondragón-Jacobo, C.; Dector-Espinoza, A.; Reynoso-Camacho, R. An integral profile of bioactive compounds and functional properties of prickly pear (*Opuntia ficus indica* L.) peel with different tonalities. *Food Chem.* **2019**, 278, 568–578.<br>Xie, G.R.; Chen, H.J. Comprehensive Betalain Profiling of Djulis (*Chenopodium formosanum*) Cultivars Using HPLC-Q-Orbitrap High-Resolution Mass Spectrometry. *J. Agric. Food Chem.* **2021**, 69, 15699–15715.<br>Slimen, I.B.; Najar, T.; Abderrabba, M. Chemical and Antioxidant Properties of Betalains. *J. Agric. Food Chem.* **2017**, 65, 675–689.<br>Kumorkiewicz-Jamro, A.; Świergosz, T.; Sutor, K.; Spórna-Kucab, A.; Wybraniec, S. Multi-colored shades of betalains: Recent advances in betacyanin chemistry. *Nat. Prod. Rep.* **2021**, 38, 2315–2346.

Neophyllocactin

**Chemical structure**	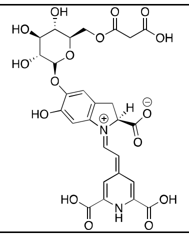
**Name:**	Neophyllocactin
**Chemical Formula**	$C_{27}H_{26}N_2O_{16}$
**Molecular weight**	634.50
**Monoisotopic mass**	634.1282
***m/z* [M+H]**	635.1355
**Theorical fragments (*m/z*)**	635.14 634.13 633.12 632.11 619.14 618.13 617.12 616.12 615.11 607.14<br>606.13 605.12 603.15 602.14 601.13 600.12 599.11 598.11 597.1<br>595.14 593.12 592.12 591.15 590.14 589.13 588.12 587.11 585.14<br>584.13 583.12 582.11 581.1 579.09 577.13 575.15 573.14 572.13<br>571.12 570.11 569.1 567.12 565.13 563.11 562.11 561.14 560.13<br>559.12 557.14 556.13 555.12 554.12 553.11 549.14 548.13 547.12<br>546.11 545.1 544.1 543.12 542.12 541.11 533.14 532.13 531.12<br>530.12 529.11 527.09 521.14 520.13 519.12 515.13 514.12 513.11<br>512.11 511.1 509.08 507.12 505.15 503.13 502.12 501.11 497.12<br>496.11 495.1 493.09 491.13 489.11 487.13 486.13 485.12 484.11<br>483.1 479.13 477.11 476.11 474.13 473.12 469.12 468.12 467.11<br>461.12 459.1 458.1 456.12 455.11 454.1 443.11 442.1 441.09<br>439.11 438.1 437.1 429.1 428.09 427.09 426.1 425.1 424.09<br>423.08 417.09 415.08 413.11 412.1 411.09 410.08 409.08 408.09<br>406.08 399.09 398.11 397.1 396.09 395.1 394.09 393.08 392.07<br>387.08 386.07 385.07 384.06 383.1 382.09 380.1 373.1 371.09<br>370.08 369.07 368.06 367.06 366.09 365.09 359.1 358.08 357.11<br>356.07 355.09 354.09 353.08 352.07 351.09 347.09 345.07 343.1<br>342.09 341.09 340.07 339.07 338.09 337.08 336.08 335.08 333.07<br>329.08 327.1 325.08 324.08 323.08 321.06 320.08 317.08 315.06<br>314.05 313.08 312.07 307.08 306.07 301.08 299.07 298.06 297.1<br>294.1 285.1 283.07 282.06 279.06 278.05 277.05 273.1 271.08<br>270.07 268.09 267.09 265.05 263.07 261.05 255.09 253.07 252.06<br>251.07 250.08 249.05 247.03 237.08 235.07 233.06 231.04 221.06<br>209.06 207.05 206.03 194.04 193.04 192.03 191.06 181.05 180.04<br>179.03 178.05 177.04 167.07 165.05 164.05 163.04 150.05 147.04<br>146.04 145.05 135.04 134.04 130.04 123.04 119.05 111.04 109.03<br>108.02 107.05 106.04
**References**	Amaya-CruzIz, D.M.; Pérez-Ramírez, I.F.; Delgado-García, J.; Mondragón-Jacobo, C.; Dector-Espinoza, A.; Reynoso-Camacho, R. An integral profile of bioactive compounds and functional properties of prickly pear (*Opuntia ficus indica* L.) peel with different tonalities. *Food Chem.* **2019**, 278, 568–578.<br>Xie, G.R.; Chen, H.J. Comprehensive Betalain Profiling of Djulis (*Chenopodium formosanum*) Cultivars Using HPLC-Q-Orbitrap High-Resolution Mass Spectrometry. *J. Agric. Food Chem.* **2021**, 69, 15699–15715.<br>Slimen, I.B.; Najar, T.; Abderrabba, M. Chemical and Antioxidant Properties of Betalains. *J. Agric. Food Chem.* **2017**, 65, 675–689.<br>Kumorkiewicz-Jamro, A.; Świergosz, T.; Sutor, K.; Spórna-Kucab, A.; Wybraniec, S. Multi-colored shades of betalains: Recent advances in betacyanin chemistry. *Nat. Prod. Rep.* **2021**, 38, 2315–2346.

2,15,17-tridecarboxy-neobetanin

**Chemical structure**	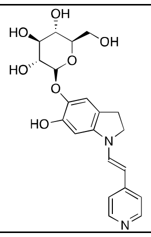
**Name:**	2,15,17-tridecarboxy-neobetanin
**Chemical Formula**	$C_{21}H_{24}N_2O_7$
**Molecular weight**	416.43
**Monoisotopic mass**	416.1584
***m/z* [M+H]**	417.1656
**Theorical fragments (*m/z*)**	417.17 416.16 415.15 414.14 413.13 412.13 403.15 402.14 401.13 400.16 399.16 398.15 397.14 396.13 395.12 391.15 390.14 389.13 388.13 387.16 386.15 385.14 384.13 383.12 382.15 381.14 380.14 379.13 375.16 373.14 372.13 371.12 370.12 369.14 368.14 367.13 366.12 365.11 363.13 361.14 359.12 357.14 356.14 355.13 354.12 353.11 351.13 350.13 349.12 345.14 344.14 343.13 342.12 341.11 340.11 339.13 338.12 337.12 331.13 329.15 327.13 326.13 325.12 324.11 323.1 321.12 320.11 319.11 315.13 314.12 313.12 312.11 311.1 310.09 309.12 308.12 307.11 302.1 301.12 300.11 299.11 298.09 297.12 296.11 295.11 294.1 293.09 288.11 286.09 285.1 284.09 283.11 282.1 281.09 279.09 278.1 276.09 273.1 271.08 270.07 269.13 268.09 267.11 266.1 255.11 254.1 253.1 241.13 240.09 239.12 237.1 236.09 229.1 227.08 226.07 225.1 224.09 223.12 213.1 211.09 210.08 197.11 195.09 180.06 179.06 178.05 177.04 176.07 175.02 165.08 164.07 163.06 162.05 161.04 160.04 159.03 152.07 151.06 150.05 149.04 148.04 147.07 146.08 145.05 144.04 143.03 139.06 138.09 137.08 136.08 135.04 134.06 133.08 132.07 131.03 130.03 129.05 127.04 124.04 123.04 122.06 121.05 120.04 119.03 118.03 117.02 116.05 115.04 114.03 113.02 111.04 110.06 109.03 108.04 107.07 106.07 105.05 104.05 103.04 102.03 101.02 100.02 99.01 97.03 95.05 93.03 92.03 91.04 90.03 89.02 88.02 87.01 86.04 85.03 83.01 78.03 77.06 76.02 75.04 74.04 73.03 72.02 71.01 69 67.02 63.04 62.04 61.03 60.02 59.01 58 57.03 56.03 55.02 53 51.02 49.03 47.01 46 45 44.03 43.02 42.03 41.03 33.03 31.02 30.01
**References**	Amaya-CruzIz, D.M.; Pérez-Ramírez, I.F.; Delgado-García, J.; Mondragón-Jacobo, C.; Dector-Espinoza, A.; Reynoso-Camacho, R. An integral profile of bioactive compounds and functional properties of prickly pear (*Opuntia ficus indica* L.) peel with different tonalities. *Food Chem.* **2019**, 278, 568–578.<br>Xie, G.R.; Chen, H.J. Comprehensive Betalain Profiling of Djulis (*Chenopodium formosanum*) Cultivars Using HPLC-Q-Orbitrap High-Resolution Mass Spectrometry. *J. Agric. Food Chem.* **2021**, 69, 15699–15715.<br>Slimen, I.B.; Najar, T.; Abderrabba, M. Chemical and Antioxidant Properties of Betalains. *J. Agric. Food Chem.* **2017**, 65, 675–689.<br>Kumorkiewicz-Jamro, A.; Świergosz, T.; Sutor, K.; Spórna-Kucab, A.; Wybraniec, S. Multi-colored shades of betalains: Recent advances in betacyanin chemistry. *Nat. Prod. Rep.* **2021**, 38, 2315–2346.

17-decarboxy-neophyllocactin

**Chemical structure**	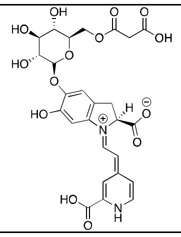
**Name:**	17-decarboxy-neophyllocactin
**Chemical Formula**	$C_{26}H_{26}N_2O_{14}$
**Molecular weight**	590.49
**Monoisotopic mass**	590.1384
***m/z* [M+H]**	591.1457
**Theorical fragments (*m/z*)**	591.15 590.14 589.13 588.12 587.11 577.13 576.12 575.15 574.14 573.14<br>572.13 571.12 563.15 562.14 561.14 560.13 559.16 558.11 557.14<br>556.13 555.12 547.16 546.15 545.14 543.12 541.15 539.13 537.11<br>533.14 532.13 531.12 530.15 529.15 528.14 527.13 521.14 519.12<br>515.13 513.11 511.13 505.15 504.14 503.13 501.11 489.15 488.14<br>487.13 486.13 485.12 477.15 475.13 474.13 473.12 472.11 471.14<br>470.13 469.12 468.11 461.16 460.11 459.14 457.12 456.12 455.11<br>453.13 452.12 451.11 450.1 445.12 444.11 443.14 442.1 441.09<br>440.12 436.12 434.11 433.12 432.09 431.11 429.09 428.12 427.11<br>426.1 425.1 424.09 415.11 414.11 413.1 403.11 401.1 400.09<br>399.08 398.11 397.1 385.1 384.1 383.09 382.11 373.1 372.09<br>371.09 369.07 366.12 364.1 359.1 358.11 357.08 356.1 355.09<br>354.08 348.11 346.09 343.09 342.08 341.08 340.1 339.1 338.09<br>337.08 336.07 329.11 327.1 326.09 325.08 323.07 315.1 313.12<br>311.1 309.09 307.07 299.1 298.09 297.09 283.11 281.09 271.07<br>266.06 265.06 263.04 261.02 257.09 255.08 251.08 250.07 249.06<br>248.05 247.04 245.03 236.05 235.04 234.06 233.07 231.05 221.06<br>220.06 219.05 217.03 215.06 213.04 207.05 206.04 205.06 204.07<br>203.02 202.05 201.04 196.06 194.04 193.07 191.06 190.05 189.04<br>188.03 187.02 182.08 180.07 179.06 178.05 177.04 175.05 173.04<br>171.03 165.05 163.06 161.04 159.03 158.02 157.01 150.05 148.04<br>147.03 145.05 143.03 141.02 133.01 131.03 130.03 129.02 127.04<br>122.02 121.05 120.04 119.03 117.02 116.01 113.02 105.02 104.01<br>103 101.02
**References**	Amaya-CruzIz, D.M.; Pérez-Ramírez, I.F.; Delgado-García, J.; Mondragón-Jacobo, C.; Dector-Espinoza, A.; Reynoso-Camacho, R. An integral profile of bioactive compounds and functional properties of prickly pear (*Opuntia ficus indica* L.) peel with different tonalities. *Food Chem.* **2019**, 278, 568–578.<br>Xie, G.R.; Chen, H.J. Comprehensive Betalain Profiling of Djulis (*Chenopodium formosanum*) Cultivars Using HPLC-Q-Orbitrap High-Resolution Mass Spectrometry. *J. Agric. Food Chem.* **2021**, 69, 15699–15715.<br>Slimen, I.B.; Najar, T.; Abderrabba, M. Chemical and Antioxidant Properties of Betalains. *J. Agric. Food Chem.* **2017**, 65, 675–689.<br>Kumorkiewicz-Jamro, A.; Świergosz, T.; Sutor, K.; Spórna-Kucab, A.; Wybraniec, S. Multi-colored shades of betalains: Recent advances in betacyanin chemistry. *Nat. Prod. Rep.* **2021**, 38, 2315–2346.

Neohylocerenin

**Chemical structure**	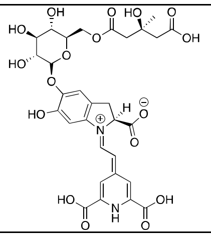
**Name:**	Neohylocerenin
**Chemical Formula**	$C_{30}H_{32}N_2O_{17}$
**Molecular weight**	692.58
**Monoisotopic mass**	692.1701
***m/z* [M+H]**	693.1774
**Theorical fragments (*m/z*)**	693.18 692.17 691.16 690.15 677.18 676.17 675.17 674.16 673.15 665.18 664.17 663.17 661.19 660.18 659.17 658.16 657.16 656.15 655.14 653.18 651.17 650.16 649.19 648.18 647.17 646.16 645.16 643.18 642.17 641.16 640.15 639.15 637.13 635.17 633.19 631.18 630.17 629.16 628.15 627.15 625.17 623.17 621.16 620.15 619.18 618.17 617.16 615.18 614.17 613.17 612.16 611.15 607.18 605.16 604.15 603.15 602.14 601.17 600.16 599.15 591.18 589.17 587.15 585.14 573.17 571.16 569.14 567.12 549.14 548.13 547.12 533.14 532.13 531.12 530.12 529.11 521.14 520.13 519.12 515.13 514.12 513.11 512.11 511.1 509.14 507.12 505.15 503.13 502.12 501.11 500.14 499.13 497.12 496.11 495.1 493.09 491.13 489.11 487.13 486.14 485.13 484.14 483.14 482.13 481.12 479.13 477.11 476.11 474.13 473.12 471.15 470.14 469.13 468.13 467.12 466.13 464.12 461.12 459.1 458.1 457.13 456.15 455.14 454.13 453.14 452.13 451.12 450.12 443.11 441.14 440.13 438.14 429.14 425.14 424.14 423.13 417.14 415.12 414.12 413.14 412.14 411.13 401.14 399.13 398.12 397.11 396.11 395.13 394.13 393.12 387.08 386.07 385.07 384.06 383.13 381.12 379.1 373.1 371.09 370.08 369.07 368.06 367.06 359.09 358.08 357.11 356.1 355.09 354.08 353.08 352.07 351.09 347.09 345.07 343.09 342.09 341.08 340.07 339.1 338.09 337.08 333.07 329.08 327.1 325.08 324.08 323.08 320.08 317.08 315.06 314.05 313.08 312.07 307.08 306.07 301.08 299.07 298.06 297.1 294.1 291.11 289.09 285.1 283.07 282.06 279.06 278.05 273.1 271.08 270.07 268.09 267.09 265.05 263.07 261.05 255.09 253.07 252.06 251.07 250.08 249.05 247.03 237.08 235.07 233.06 231.04 221.06 209.06 207.05 206.03 194.04 193.04 192.03 191.06 181.05 180.04 179.03 178.05 177.04 167.07 165.05 164.05 163.04 150.05 147.04 146.04 145.05 135.04 134.04 130.04 123.04 119.05 111.04 109.03 108.02 107.05 106.04
**References**	Amaya-CruzIz, D.M.; Pérez-Ramírez, I.F.; Delgado-García, J.; Mondragón-Jacobo, C.; Dector-Espinoza, A.; Reynoso-Camacho, R. An integral profile of bioactive compounds and functional properties of prickly pear (*Opuntia ficus indica* L.) peel with different tonalities. *Food Chem.* **2019**, 278, 568–578.<br>Xie, G.R.; Chen, H.J. Comprehensive Betalain Profiling of Djulis (*Chenopodium formosanum*) Cultivars Using HPLC-Q-Orbitrap High-Resolution Mass Spectrometry. *J. Agric. Food Chem.* **2021**, 69, 15699–15715.<br>Slimen, I.B.; Najar, T.; Abderrabba, M. Chemical and Antioxidant Properties of Betalains. *J. Agric. Food Chem.* **2017**, 65, 675–689.<br>Kumorkiewicz-Jamro, A.; Świergosz, T.; Sutor, K.; Spórna-Kucab, A.; Wybraniec, S. Multi-colored shades of betalains: Recent advances in betacyanin chemistry. *Nat. Prod. Rep.* **2021**, 38, 2315–2346.

17-decarboxy-neohylocerenin

**Chemical structure**	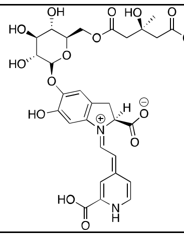
**Name:**	17-decarboxy-neohylocerenin
**Chemical Formula**	$C_{29}H_{32}N_2O_{15}$
**Molecular weight**	648.57
**Monoisotopic mass**	648.1803
***m/z* [M+H]**	649.1875
**Theorical fragments (*m/z*)**	649.19 648.18 647.17 646.16 645.16 635.17 634.16 633.16 632.18 631.18<br>630.17 629.16 621.19 620.18 619.18 618.17 617.2 616.15 615.18<br>614.17 613.17 605.2 604.19 603.18 602.17 601.17 599.19 597.17<br>595.16 591.15 590.17 589.17 588.16 587.19 586.18 585.17 583.16<br>579.18 577.17 575.15 573.14 571.16 570.15 569.18 567.16 563.15<br>561.17 559.16 558.15 556.13 547.16 546.15 545.14 543.16 541.15<br>531.12 529.15 527.13 526.16 517.15 515.13 513.11 510.16 508.14<br>505.15 504.14 503.13 502.16 501.15 500.14 499.13 498.16 494.17<br>492.15 490.13 489.15 487.13 486.16 485.12 484.14 482.13 477.15<br>475.13 474.13 473.12 472.11 471.14 469.12 461.16 460.11 459.14<br>457.12 456.15 455.11 453.13 451.11 445.12 444.12 443.14 442.1<br>441.13 433.12 431.11 430.13 429.14 428.12 427.11 426.11 425.1<br>417.14 415.11 414.11 413.1 403.11 401.1 400.09 399.08 397.1<br>385.1 384.1 383.09 382.11 373.1 371.09 369.07 366.12 364.1<br>358.11 356.1 355.09 354.08 348.11 346.09 343.09 342.08 341.08<br>340.1 339.1 338.09 337.08 336.07 329.11 327.1 325.08 324.11<br>323.1 321.08 319.07 315.1 313.12 311.1 309.12 308.11 307.1<br>306.09 305.09 303.07 299.1 298.09 297.09 294.09 293.09 291.11<br>289.09 283.11 281.09 278.1 277.09 275.11 273.1 271.07 265.09<br>264.08 263.08 261.06 259.08 257.09 255.08 251.11 249.1 248.09<br>247.08 246.07 245.07 243.09 235.08 234.06 233.06 231.09 229.07<br>221.06 220.06 219.09 217.07 216.06 215.06 205.06 204.07 203.06<br>202.05 201.08 199.06 196.06 194.04 191.06 189.08 188.07 187.06<br>182.08 180.07 179.06 178.05 177.08 175.06 174.05 173.04 165.05<br>163.06 162.05 161.04 159.07 157.05 150.05 148.04 147.07 146.02<br>145.05 144.04 143.03 135.07 134.06 133.05 131.03 129.05 128.05<br>127.04 122.02 121.05 120.04 119.03 118.06 117.05 116.05 115.04<br>113.02 111.04 109.03 105.02 104.05 103.04 102.03 101.06 100.05
**References**	Amaya-CruzIz, D.M.; Pérez-Ramírez, I.F.; Delgado-García, J.; Mondragón-Jacobo, C.; Dector-Espinoza, A.; Reynoso-Camacho, R. An integral profile of bioactive compounds and functional properties of prickly pear (*Opuntia ficus indica* L.) peel with different tonalities. *Food Chem.* **2019**, 278, 568–578.<br>Xie, G.R.; Chen, H.J. Comprehensive Betalain Profiling of Djulis (*Chenopodium formosanum*) Cultivars Using HPLC-Q-Orbitrap High-Resolution Mass Spectrometry. *J. Agric. Food Chem.* **2021**, 69, 15699–15715.<br>Slimen, I.B.; Najar, T.; Abderrabba, M. Chemical and Antioxidant Properties of Betalains. *J. Agric. Food Chem.* **2017**, 65, 675–689.<br>Kumorkiewicz-Jamro, A.; Świergosz, T.; Sutor, K.; Spórna-Kucab, A.; Wybraniec, S. Multi-colored shades of betalains: Recent advances in betacyanin chemistry. *Nat. Prod. Rep.* **2021**, 38, 2315–2346.

2,17-bidecarboxy-2,3-dehydro-neobetanin

**Chemical structure**	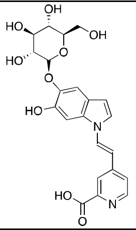
**Name:**	2,17-bidecarboxy-2,3-dehydro-neobetanin
**Chemical Formula**	$C_{22}H_{22}N_2O_9$
**Molecular weight**	458.42
**Monoisotopic mass**	458.1325
***m/z* [M+H]**	459.1398
**Theorical fragments (*m/z*)**	459.14 458.13 443.14 442.14 441.13 440.12 433.12 432.12 431.14 425.13<br>424.13 423.12 422.11 417.13 416.12 415.15 414.14 413.13 411.12<br>407.12 406.12 405.11 404.1 400.13 399.12 398.11 397.14 396.13<br>395.12 393.11 389.13 388.11 387.12 384.1 382.12 381.11 380.14<br>379.13 378.12 377.11 373.14 371.12 369.11 368.1 362.13 361.12<br>360.11 359.1 355.09 353.11 326.09 325.08 323.1 310.09 309.09<br>298.09 297.09 296.08 295.07 292.08 283.11 282.1 281.09 280.08<br>279.08 271.08 270.07 269.09 266.1 265.1 264.09 263.08 262.08<br>257.09 256.08 255.08 254.07 253.1 252.06 251.08 241.1 240.09<br>239.08 238.07 237.08 236.09 235.06 234.05 227.08 226.07 225.07<br>224.09 221.11 216.04 213.1 212.09 211.09 210.08 197.11 195.09<br>189.07 176.06 165.07 164.06 163.05 162.04 159.06 150.05 149.05<br>148.04 147.07 146.06 145.08 136.04 134.06 132.04 122.02 121.08<br>120.06 119.05 118.04 109.03 108.02 107.05 106.03 105.02 104.05<br>102.05
**References**	Amaya-CruzIz, D.M.; Pérez-Ramírez, I.F.; Delgado-García, J.; Mondragón-Jacobo, C.; Dector-Espinoza, A.; Reynoso-Camacho, R. An integral profile of bioactive compounds and functional properties of prickly pear (*Opuntia ficus indica* L.) peel with different tonalities. *Food Chem.* **2019**, 278, 568–578.<br>Xie, G.R.; Chen, H.J. Comprehensive Betalain Profiling of Djulis (*Chenopodium formosanum*) Cultivars Using HPLC-Q-Orbitrap High-Resolution Mass Spectrometry. *J. Agric. Food Chem.* **2021**, 69, 15699–15715.<br>Slimen, I.B.; Najar, T.; Abderrabba, M. Chemical and Antioxidant Properties of Betalains. *J. Agric. Food Chem.* **2017**, 65, 675–689.<br>Kumorkiewicz-Jamro, A.; Świergosz, T.; Sutor, K.; Spórna-Kucab, A.; Wybraniec, S. Multi-colored shades of betalains: Recent advances in betacyanin chemistry. *Nat. Prod. Rep.* **2021**, 38, 2315–2346.

2-decarboxy-neohylocerenin

**Chemical structure**	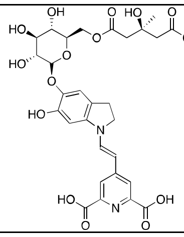
**Name:**	2-decarboxy-neohylocerenin
**Chemical Formula**	$C_{29}H_{32}N_2O_{15}$
**Molecular weight**	648.57
**Monoisotopic mass**	648.1803
***m/z* [M+H]**	649.1875
**Theorical fragments (*m/z*)**	649.19 648.18 647.17 646.16 645.16 635.17 634.16 633.16 632.18 631.18 630.17 629.16 623.17 621.19 620.15 619.18 618.17 617.2 616.19 615.18 614.17 613.17 607.18 605.2 604.19 603.18 602.17 601.17 600.16 599.19 597.17 595.16 591.15 589.17 588.16 587.19 585.17 583.16 575.15 573.14 571.16 570.15 569.18 567.16 563.15 561.14 559.16 558.15 556.13 547.16 546.15 545.14 543.16 541.15 531.12 529.15 527.13 517.15 513.11 505.15 504.14 503.13 491.13 489.15 488.14 487.13 486.13 485.12 482.17 479.13 477.11 475.13 474.13 473.12 472.11 471.14 470.13 469.12 467.11 466.17 464.16 461.16 460.11 459.1 458.17 457.12 456.15 455.14 454.13 453.13 451.11 450.18 448.16 446.14 445.12 444.12 443.14 442.17 441.14 440.16 438.14 432.15 431.11 430.13 429.14 428.16 427.11 426.11 425.1 424.16 423.13 422.14 420.13 417.14 415.11 414.11 413.1 412.14 411.13 409.1 403.11 401.1 400.09 399.08 397.1 385.1 384.1 383.09 373.1 371.09 369.07 355.09 354.08 343.09 342.08 341.08 338.12 329.11 327.1 326.09 325.08 324.11 323.1 322.09 321.08 320.11 319.07 317.08 315.06 314.12 313.08 312.11 311.1 309.12 308.11 307.1 306.09 305.09 303.07 302.1 301.08 300.11 299.1 298.06 297.1 296.11 295.1 294.1 293.09 291.11 289.09 287.08 286.09 285.1 283.07 282.1 279.09 278.1 277.09 276.09 275.11 273.1 271.08 268.08 267.09 265.09 264.08 263.08 261.06 259.08 257.1 255.09 253.07 251.11 249.1 248.09 247.08 246.07 245.07 243.09 235.08 234.06 233.06 231.09 229.07 221.06 220.05 219.09 217.07 216.06 215.06 209.06 205.07 203.06 201.08 199.06 194.04 192.03 191.06 189.08 188.07 187.06 179.06 177.08 176.07 175.06 174.05 173.04 166.01 165.08 163.06 162.05 161.04 159.07 157.05 152.07 151.06 150.05 149.04 147.07 146.02 145.05 144.04 143.03 138.09 136.08 135.07 134.06 133.05 132.04 131.03 129.05 128.05 127.04 126.03 124.04 123.04 122.06 121.06 120.04 119.03 118.06 117.05 116.05 115.04 113.02 111.04 110.06 109.03 108.04 107.05 105.02 104.05 103.04 102.03 101.06 100.05
**References**	Amaya-CruzIz, D.M.; Pérez-Ramírez, I.F.; Delgado-García, J.; Mondragón-Jacobo, C.; Dector-Espinoza, A.; Reynoso-Camacho, R. An integral profile of bioactive compounds and functional properties of prickly pear (*Opuntia ficus indica* L.) peel with different tonalities. *Food Chem.* **2019**, 278, 568–578.<br>Xie, G.R.; Chen, H.J. Comprehensive Betalain Profiling of Djulis (*Chenopodium formosanum*) Cultivars Using HPLC-Q-Orbitrap High-Resolution Mass Spectrometry. *J. Agric. Food Chem.* **2021**, 69, 15699–15715.<br>Slimen, I.B.; Najar, T.; Abderrabba, M. Chemical and Antioxidant Properties of Betalains. *J. Agric. Food Chem.* **2017**, 65, 675–689.<br>Kumorkiewicz-Jamro, A.; Świergosz, T.; Sutor, K.; Spórna-Kucab, A.; Wybraniec, S. Multi-colored shades of betalains: Recent advances in betacyanin chemistry. *Nat. Prod. Rep.* **2021**, 38, 2315–2346.

2-decarboxy-neophyllocactin

**Chemical structure**	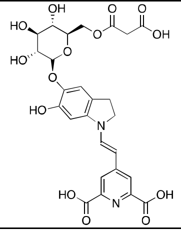
**Name:**	2-decarboxy-neophyllocactin
**Chemical Formula**	$C_{26}H_{26}N_2O_{14}$
**Molecular weight**	590.49
**Monoisotopic mass**	590.1384
***m/z* [M+H]**	591.1457
**Theorical fragments (*m/z*)**	591.15 590.14 589.13 588.12 587.11 577.13 576.12 575.15 574.14 573.14<br>572.13 571.12 565.13 563.15 562.11 561.14 560.13 559.16 558.15<br>557.14 556.13 555.12 554.12 553.11 549.14 547.16 546.15 545.14<br>544.13 543.12 542.12 541.15 539.13 537.11 531.12 530.15 529.15<br>528.14 527.13 517.11 513.11 511.13 505.15 504.14 503.13 502.12<br>501.11 491.13 489.15 488.14 487.13 486.13 485.12 484.11 479.13<br>477.11 475.13 474.13 473.12 472.11 471.14 470.13 469.12 467.11<br>463.13 461.16 460.11 459.1 458.1 457.12 456.12 455.11 453.13<br>451.11 449.12 447.1 445.12 444.12 443.14 442.1 441.09 439.11<br>433.12 431.11 430.1 429.09 427.11 426.11 425.1 424.12 419.11<br>415.11 414.11 413.1 411.12 409.1 408.13 406.11 403.11 401.1<br>400.12 399.08 398.11 397.1 396.09 395.09 390.12 389.1 388.1<br>386.11 385.1 384.13 383.09 382.11 380.1 379.09 374.11 373.1<br>372.09 371.09 370.11 369.07 368.1 367.1 366.12 365.09 364.1<br>362.09 359.1 357.08 356.07 355.09 354.08 353.09 343.09 342.08<br>341.08 338.12 329.11 327.1 326.09 325.08 324.07 320.11 317.08<br>315.06 314.12 313.08 312.11 311.1 302.1 301.08 300.11 299.1<br>298.06 297.1 296.11 294.1 286.09 285.1 283.07 282.1 279.09<br>278.1 276.09 273.1 268.08 267.09 266.06 265.06 264.05 263.04<br>261.02 255.09 251.08 250.07 249.06 248.05 247.04 245.03 237.06<br>236.05 235.04 234.06 233.07 232.06 231.05 230.04 229.03 225.06<br>221.06 220.06 219.05 218.04 217.03 215.06 213.04 209.06 207.05<br>206.04 205.03 204.06 203.02 202.05 201.04 197.04 195.03 194.04<br>193.07 192.03 191.06 190.05 189.04 188.03 187.02 180.06 179.06<br>177.04 176.07 175.06 174.05 173.04 172.04 171.03 170.02 169.01<br>166.01 165.08 163.06 162.05 161.04 160.04 159.03 158.02 157.01<br>155.03 153.02 152.07 151.06 150.05 149.04 147.03 145.05 143.03<br>141.02 138.09 136.08 135.04 134.06 133.01 132.04 131.03 130.03<br>129.02 127.04 125.02 124.04 123.04 122.06 121.06 120.04 119.03<br>118.03 117.02 116.01 115 113.02 111.04 110.06 108.04 107.05<br>105.02 104.01 103 102.03 101.02 100.02
**References**	Amaya-CruzIz, D.M.; Pérez-Ramírez, I.F.; Delgado-García, J.; Mondragón-Jacobo, C.; Dector-Espinoza, A.; Reynoso-Camacho, R. An integral profile of bioactive compounds and functional properties of prickly pear (*Opuntia ficus indica* L.) peel with different tonalities. *Food Chem.* **2019**, 278, 568–578.<br>Xie, G.R.; Chen, H.J. Comprehensive Betalain Profiling of Djulis (*Chenopodium formosanum*) Cultivars Using HPLC-Q-Orbitrap High-Resolution Mass Spectrometry. *J. Agric. Food Chem.* **2021**, 69, 15699–15715.<br>Slimen, I.B.; Najar, T.; Abderrabba, M. Chemical and Antioxidant Properties of Betalains. *J. Agric. Food Chem.* **2017**, 65, 675–689.<br>Kumorkiewicz-Jamro, A.; Świergosz, T.; Sutor, K.; Spórna-Kucab, A.; Wybraniec, S. Multi-colored shades of betalains: Recent advances in betacyanin chemistry. *Nat. Prod. Rep.* **2021**, 38, 2315–2346.

Neolampranthin II

**Chemical structure**	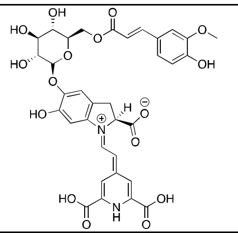
**Name:**	Neolampranthin II
**Chemical Formula**	$C_{34}H_{32}N_2O_{16}$
**Molecular weight**	724.63
**Monoisotopic mass**	724.1752
***m/z* [M+H]**	725.1825
**Theorical fragments (*m/z*)**	725.18 724.17 723.17 722.16 709.19 708.18 707.17 706.16 705.16 697.19<br>696.18 695.17 693.19 692.18 691.18 690.17 689.16 688.15 687.15<br>685.19 683.17 682.16 681.19 680.18 679.18 678.17 677.16 673.17<br>672.16 671.15 669.14 667.18 666.17 665.16 664.19 663.18 662.17<br>661.17 660.16 659.15 655.18 653.16 652.15 650.17 649.17 647.19<br>645.17 644.16 643.16 637.17 636.16 635.15 634.14 632.16 631.16<br>619.16 617.14 609.17 601.15 545.15 544.14 543.14 532.14 531.12<br>530.12 529.11 528.15 527.14 526.13 519.15 518.14 517.13 516.15<br>515.13 514.13 513.11 512.11 511.1 510.14 503.13 502.12 501.14<br>500.13 499.12 498.14 497.12 496.11 495.1 493.09 491.13 489.14<br>488.16 487.15 486.14 485.12 484.11 483.13 482.12 479.11 477.09<br>475.12 474.14 473.14 472.14 471.14 470.14 469.12 468.12 467.11<br>461.14 459.1 458.1 456.14 455.13 452.13 451.11 449.14 447.13<br>446.12 444.14 443.13 441.09 431.13 430.13 429.12 428.11 426.13<br>425.12 423.08 417.09 415.08 413.12 411.11 387.08 386.07 385.07<br>384.06 373.1 371.09 370.08 369.07 368.06 367.06 359.09 358.08<br>357.11 356.1 355.09 354.08 353.08 352.07 351.09 350.09 347.09<br>345.07 343.09 342.08 341.08 340.07 339.1 338.09 337.08 336.07<br>334.09 333.07 329.08 327.1 326.09 325.08 324.08 323.08 322.09<br>321.1 320.08 319.07 317.08 315.06 314.05 313.08 312.07 311.1<br>307.08 306.07 302.07 301.08 299.07 298.06 294.1 293.09 285.09<br>283.07 282.06 279.06 278.05 277.05 276.09 267.09 265.05 264.04<br>263.07 262.06 261.05 255.09 253.07 252.06 251.07 250.08 249.05<br>247.03 237.08 235.07 233.06 231.04 221.06 209.06 207.05 206.03<br>194.04 193.04 192.03 191.06 190.05 181.05 180.04 179.03 178.05<br>177.04 176.03 167.07 165.05 164.05 163.04 162.05 150.05 149.05<br>148.04 147.04 146.04 145.05 136.08 135.04 134.04 132.04 130.04<br>123.04 119.05 111.04 109.03 108.02 107.05 106.04
**References**	Amaya-CruzIz, D.M.; Pérez-Ramírez, I.F.; Delgado-García, J.; Mondragón-Jacobo, C.; Dector-Espinoza, A.; Reynoso-Camacho, R. An integral profile of bioactive compounds and functional properties of prickly pear (*Opuntia ficus indica* L.) peel with different tonalities. *Food Chem.* **2019**, 278, 568–578.<br>Xie, G.R.; Chen, H.J. Comprehensive Betalain Profiling of Djulis (*Chenopodium formosanum*) Cultivars Using HPLC-Q-Orbitrap High-Resolution Mass Spectrometry. *J. Agric. Food Chem.* **2021**, 69, 15699–15715.<br>Slimen, I.B.; Najar, T.; Abderrabba, M. Chemical and Antioxidant Properties of Betalains. *J. Agric. Food Chem.* **2017**, 65, 675–689.<br>Kumorkiewicz-Jamro, A.; Świergosz, T.; Sutor, K.; Spórna-Kucab, A.; Wybraniec, S. Multi-colored shades of betalains: Recent advances in betacyanin chemistry. *Nat. Prod. Rep.* **2021**, 38, 2315–2346.

Dehydrogenated tridecarboxy-neobetanin

**Chemical structure**	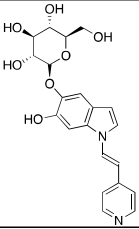
**Name:**	Dehydrogenated tridecarboxy-neobetanin
**Chemical Formula**	$C_{21}H_{22}N_2O_7$
**Molecular weight**	414.41
**Monoisotopic mass**	414.1427
***m/z* [M+H]**	415.1499
**Theorical fragments (*m/z*)**	415.15 414.14 413.13 412.13 411.12 410.11 399.16 398.15 397.14 396.13<br>395.12 394.12 393.11 389.13 388.13 387.16 385.14 384.13 383.12<br>382.12 381.11 380.14 379.13 378.12 377.11 373.14 371.12 370.12<br>369.14 368.1 367.13 366.12 365.11 364.11 363.1 361.12 359.12<br>357.11 355.13 354.12 353.11 352.11 351.1 349.12 348.11 347.1<br>343.13 342.12 341.11 340.11 339.1 338.09 337.12 336.11 335.1<br>329.11 327.13 325.12 324.11 323.1 322.09 321.09 319.11 318.1<br>317.09 313.12 312.11 311.1 310.09 309.09 308.08 307.11 306.1<br>305.09 300.09 299.1 296.09 295.11 294.1 293.09 292.08 291.08<br>286.09 285.1 283.11 282.08 281.09 280.08 279.08 277.1 276.09<br>275.08 273.1 271.08 270.07 267.11 265.1 264.09 255.09 254.08<br>253.1 252.09 251.08 239.12 237.1 236.09 235.09 234.08 227.08<br>226.07 223.12 221.11 220.1 213.1 211.09 210.08 197.11 195.09<br>180.06 179.06 178.05 177.04 175.02 174.05 165.08 164.07 163.06<br>162.05 161.04 160.04 159.03 151.06 150.05 149.05 148.04 147.07<br>146.06 145.05 144.04 143.03 139.06 137.08 136.08 135.07 134.06<br>133.05 132.07 131.03 130.03 129.05 127.04 122.06 121.05 120.04<br>119.03 118.03 117.02 116.05 115.04 114.03 113.02 110.06 109.03<br>108.02 107.07 105.05 104.05 103.04 102.03 101.02 100.02
**References**	Amaya-CruzIz, D.M.; Pérez-Ramírez, I.F.; Delgado-García, J.; Mondragón-Jacobo, C.; Dector-Espinoza, A.; Reynoso-Camacho, R. An integral profile of bioactive compounds and functional properties of prickly pear (*Opuntia ficus indica* L.) peel with different tonalities. *Food Chem.* **2019**, 278, 568–578.<br>Xie, G.R.; Chen, H.J. Comprehensive Betalain Profiling of Djulis (*Chenopodium formosanum*) Cultivars Using HPLC-Q-Orbitrap High-Resolution Mass Spectrometry. *J. Agric. Food Chem.* **2021**, 69, 15699–15715.<br>Slimen, I.B.; Najar, T.; Abderrabba, M. Chemical and Antioxidant Properties of Betalains. *J. Agric. Food Chem.* **2017**, 65, 675–689.<br>Kumorkiewicz-Jamro, A.; Świergosz, T.; Sutor, K.; Spórna-Kucab, A.; Wybraniec, S. Multi-colored shades of betalains: Recent advances in betacyanin chemistry. *Nat. Prod. Rep.* **2021**, 38, 2315–2346.

Amaranthin-type
Amaranthin

**Chemical structure**	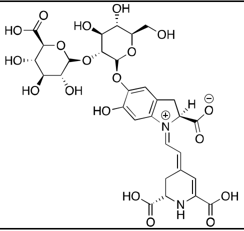
**Name:**	Amaranthin
**Chemical Formula**	$C_{30}H_{34}N_2O_{19}$
**Molecular weight**	726.60
**Monoisotopic mass**	726.1756
***m/z* [M+H]**	727.1829
**Theorical fragments (*m/z*)**	727.18 726.18 725.17 724.16 711.19 710.18 709.17 708.16 707.16 699.19<br>698.18 697.17 695.19 694.19 693.18 692.17 691.16 690.15 689.15<br>687.19 685.17 683.19 682.19 681.18 680.17 679.16 677.18 676.17<br>675.17 674.16 673.15 671.14 669.18 667.2 665.18 664.17 663.17<br>662.16 661.15 659.17 657.18 655.16 654.15 653.18 652.17 651.17<br>649.19 648.18 647.17 646.16 645.16 641.18 639.17 638.16 637.15<br>636.14 635.17 634.16 633.16 625.19 623.17 621.16 619.14 607.18<br>605.16 603.15 601.13 558.15 551.15 550.14 549.14 545.14 535.16<br>534.15 533.14 532.13 531.12 523.16 522.15 521.14 519.16 518.13<br>517.15 516.13 515.13 514.12 513.11 511.16 509.14 507.16 505.15<br>504.14 503.13 502.13 501.12 500.12 499.13 498.13 497.12 495.1<br>493.15 491.13 489.15 488.14 487.13 486.12 485.13 484.12 483.11<br>482.11 481.15 479.13 478.12 477.15 476.14 475.13 473.13 472.12<br>471.14 470.13 469.12 465.15 463.13 462.12 461.13 460.11 459.14<br>458.13 457.13 456.13 455.12 449.13 447.11 446.11 445.13 444.13<br>443.12 433.13 431.12 430.11 429.1 428.09 427.12 426.12 425.11<br>415.12 413.11 411.09 389.1 388.09 387.08 386.07 375.12 373.1<br>372.1 371.09 370.08 369.07 361.1 360.1 359.12 358.11 357.11<br>356.1 355.09 354.08 353.08 351.06 349.1 347.09 345.11 343.13<br>342.09 341.11 340.1 339.1 338.09 337.08 335.09 331.09 329.11<br>327.1 326.09 325.09 324.08 323.08 319.09 317.08 316.07 315.1<br>314.09 313.08 307.08 306.07 303.1 301.08 300.07 299.07 298.06<br>297.1 287.1 285.1 284.08 283.07 282.06 281.08 280.07 279.09<br>273.1 271.08 270.07 269.09 268.09 267.06 265.08 264.07 263.07<br>262.06 255.09 253.08 252.06 251.07 250.08 249.05 247.07 245.06<br>237.09 235.07 233.06 231.04 223.07 211.07 207.08 205.06 196.06<br>194.04 193.04 182.08 180.04 179.03 178.05 177.04 165.05 164.05<br>163.04 147.04 146.04 135.04 134.04 130.04 123.04 119.05 111.04<br>109.03 108.02 107.05 106.04
**References**	Piattelli, M.; Minale, L. Pigments of centrospermae—I. *Phytochemistry*, **1964**, 3, 307-311.<br>Minale, L.; Piattelli, M.; de Stefano, S.; Nicolaus, R.A. Pigments of Centrospermae VI. Acylated betacyanins. *Phytochemistry* **1966**, 5, 1037–1052.<br>Cai, Y.; Sun, M.; Corke, H. Identification and Distribution of Simple and Acylated Betacyanins in the Amaranthaceae. *J. Agric. Food Chem.* **2001**, 49, 1971–1978.<br>Slimen, I.B.; Najar, T.; Abderrabba, M. Chemical and Antioxidant Properties of Betalains. *J. Agric. Food Chem.* **2017**, 65, 675–689.<br>Kumorkiewicz-Jamro, A.; Świergosz, T.; Sutor, K.; Spórna-Kucab, A.; Wybraniec, S. Multi-colored shades of betalains: Recent advances in betacyanin chemistry. *Nat. Prod. Rep.* **2021**, 38, 2315–2346.<br>Miguel, M.G. Betalains in Some Species of the Amaranthaceae Family: A Review. *Antioxidants* **2018**, 7, 53.<br>Xie, G.R.; Chen, H.J. Comprehensive Betalain Profiling of Djulis (*Chenopodium formosanum*) Cultivars Using HPLC-Q-Orbitrap High-Resolution Mass Spectrometry. *J. Agric. Food Chem.* **2021**, 69, 15699–15715.

	Araujo-León, J.A.; Aguilar-Hernández, V.; Sánchez-del Pino, I.; Brito-Argáez, L.; Peraza-Sánchez, S.R.; Xingú-López, A.; Ortiz-Andrade, R. Analysis of Red Amaranth (*Amaranthus cruentus* L.) Betalains by LC-MS. *J. Mex. Chem. Soc.* **2023**, 67, 227–239.

Isoamaranthin

Chemical structure	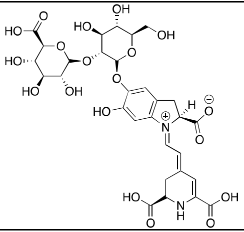
**Name:**	Isoamaranthin
**Chemical Formula**	$C_{30}H_{34}N_2O_{19}$
**Molecular weight**	726.60
**Monoisotopic mass**	726.1756
***m/z* [M+H]**	727.1829
**Theorical fragments (*m/z*)**	727.18 726.18 725.17 724.16 711.19 710.18 709.17 708.16 707.16 699.19<br>698.18 697.17 695.19 694.19 693.18 692.17 691.16 690.15 689.15<br>687.19 685.17 683.19 682.19 681.18 680.17 679.16 677.18 676.17<br>675.17 674.16 673.15 671.14 669.18 667.2 665.18 664.17 663.17<br>662.16 661.15 659.17 657.18 655.16 654.15 653.18 652.17 651.17<br>649.19 648.18 647.17 646.16 645.16 641.18 639.17 638.16 637.15<br>636.14 635.17 634.16 633.16 625.19 623.17 621.16 619.14 607.18<br>605.16 603.15 601.13 558.15 551.15 550.14 549.14 545.14 535.16<br>534.15 533.14 532.13 531.12 523.16 522.15 521.14 519.16 518.13<br>517.15 516.13 515.13 514.12 513.11 511.16 509.14 507.16 505.15<br>504.14 503.13 502.13 501.12 500.12 499.13 498.13 497.12 495.1<br>493.15 491.13 489.15 488.14 487.13 486.12 485.13 484.12 483.11<br>482.11 481.15 479.13 478.12 477.15 476.14 475.13 473.13 472.12<br>471.14 470.13 469.12 465.15 463.13 462.12 461.13 460.11 459.14<br>458.13 457.13 456.13 455.12 449.13 447.11 446.11 445.13 444.13<br>443.12 433.13 431.12 430.11 429.1 428.09 427.12 426.12 425.11<br>415.12 413.11 411.09 389.1 388.09 387.08 386.07 375.12 373.1<br>372.1 371.09 370.08 369.07 361.1 360.1 359.12 358.11 357.11<br>356.1 355.09 354.08 353.08 351.06 349.1 347.09 345.11 343.13<br>342.09 341.11 340.1 339.1 338.09 337.08 335.09 331.09 329.11<br>327.1 326.09 325.09 324.08 323.08 319.09 317.08 316.07 315.1<br>314.09 313.08 307.08 306.07 303.1 301.08 300.07 299.07 298.06<br>297.1 287.1 285.1 284.08 283.07 282.06 281.08 280.07 279.09<br>273.1 271.08 270.07 269.09 268.09 267.06 265.08 264.07 263.07<br>262.06 255.09 253.08 252.06 251.07 250.08 249.05 247.07 245.06<br>237.09 235.07 233.06 231.04 223.07 211.07 207.08 205.06 196.06<br>194.04 193.04 182.08 180.04 179.03 178.05 177.04 165.05 164.05<br>163.04 147.04 146.04 135.04 134.04 130.04 123.04 119.05 111.04<br>109.03 108.02 107.05 106.04
**References**	Piattelli, M.; Minale, L. Pigments of centrospermae—I. *Phytochemistry*, **1964**, 3, 307-311.<br>Minale, L.; Piattelli, M.; de Stefano, S.; Nicolaus, R.A. Pigments of Centrospermae VI. Acylated betacyanins. *Phytochemistry* **1966**, 5, 1037–1052.<br>Cai, Y.; Sun, M.; Corke, H. Identification and Distribution of Simple and Acylated Betacyanins in the Amaranthaceae. *J. Agric. Food Chem.* **2001**, 49, 1971–1978.<br>Slimen, I.B.; Najar, T.; Abderrabba, M. Chemical and Antioxidant Properties of Betalains. *J. Agric. Food Chem.* **2017**, 65, 675–689.<br>Kumorkiewicz-Jamro, A.; Świergosz, T.; Sutor, K.; Spórna-Kucab, A.; Wybraniec, S. Multi-colored shades of betalains: Recent advances in betacyanin chemistry. *Nat. Prod. Rep.* **2021**, 38, 2315–2346.<br>Miguel, M.G. Betalains in Some Species of the Amaranthaceae Family: A Review. *Antioxidants* **2018**, 7, 53.<br>Xie, G.R.; Chen, H.J. Comprehensive Betalain Profiling of Djulis (*Chenopodium formosanum*) Cultivars Using HPLC-Q-Orbitrap High-Resolution Mass Spectrometry. *J. Agric. Food Chem.* **2021**, 69, 15699–15715.<br>Araujo-León, J.A.; Aguilar-Hernández, V.; Sánchez-del Pino, I.; Brito-Argáez, L.; Peraza-Sánchez, S.R.; Xingú-López, A.; Ortiz-Andrade, R. Analysis of Red Amaranth (*Amaranthus*

	cruentus L.) Betalains by LC-MS. *J. Mex. Chem. Soc.* **2023**, 67, 227–239.

Iresinin I

Chemical structure	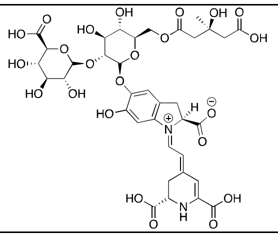
**Name:**	Iresinin I
**Chemical Formula**	$C_{36}H_{42}N_2O_{23}$
**Molecular weight**	870.72
**Monoisotopic mass**	870.2178
***m/z* [M+H]**	871.2251
**Theorical fragments (*m/z*)**	871.23 870.22 869.21 868.2 855.23 854.22 853.21 852.21 851.2 843.23 842.22 841.21 839.24 837.22 836.21 835.2 833.19 831.23 829.21 827.24 826.23 825.22 824.21 823.2 821.22 819.21 817.19 813.22 811.24 809.22 808.22 807.21 801.22 799.2 798.2 797.22 796.22 795.21 785.22 783.21 782.2 781.19 767.21 765.2 763.18 727.18 726.18 725.17 711.19 709.17 708.16 707.16 702.19 699.19 697.17 695.19 694.19 693.18 691.16 689.15 683.19 681.18 679.2 678.19 677.18 676.17 675.16 673.15 669.18 667.2 665.18 663.2 662.17 661.19 660.18 659.17 658.16 657.16 655.16 651.17 649.19 647.17 646.17 645.17 644.16 643.18 641.16 639.17 637.15 633.19 632.18 631.18 630.17 629.17 628.16 627.16 626.15 625.19 623.17 621.16 619.18 617.17 616.16 607.18 606.17 605.17 601.18 600.17 599.16 593.17 591.16 590.15 589.18 588.17 587.16 577.18 575.16 574.15 573.15 572.14 571.17 570.16 569.15 561.18 559.17 557.15 551.15 549.14 535.16 534.15 533.14 532.13 531.12 518.13 517.15 516.13 515.13 514.12 513.11 502.16 501.12 500.12 499.13 497.12 486.14 485.13 484.14 483.11 482.13 481.12 479.13 473.13 470.14 469.13 468.13 467.12 461.13 455.12 452.13 451.12 450.12 449.13 447.11 446.11 445.13 444.13 443.12 441.14 433.13 431.12 429.14 428.09 427.12 426.12 425.11 423.13 417.14 415.12 414.12 413.14 412.14 411.13 401.14 399.13 397.11 396.11 395.13 394.13 393.12 389.1 388.09 387.08 386.07 383.13 381.12 375.12 373.1 372.1 371.09 370.08 369.07 361.1 360.1 359.12 357.11 356.1 355.09 354.08 353.08 351.06 349.1 347.09 345.11 343.13 342.08 341.11 339.1 337.08 335.09 331.09 329.11 327.1 326.09 325.08 324.08 319.09 317.08 316.07 315.1 314.09 313.08 306.07 303.1 301.08 300.07 299.07 287.1 285.09 284.08 283.07 281.08 280.07 273.1 271.11 269.09 267.06 265.08 264.07 263.07 262.06 255.09 253.08 251.07 249.05 247.07 245.06 237.09 235.07 233.06 231.04 223.07 211.07 207.08 205.06 196.06 194.04 193.04 182.08 180.04 179.03 178.05 177.04 165.05 164.05 163.04 147.04 146.04 135.04 134.04 130.04 123.04 119.05 111.04 109.03 108.02 107.05 106.04
**References**	Minale, L.; Piattelli, M.; de Stefano, S.; Nicolaus, R.A. Pigments of Centrospermae VI. Acylated betacyanins. *Phytochemistry* **1966**, 5, 1037–1052.<br>Cai, Y.; Sun, M.; Corke, H. Identification and Distribution of Simple and Acylated Betacyanins in the Amaranthaceae. *J. Agric. Food Chem.* **2001**, 49, 1971–1978.<br>Slimen, I.B.; Najar, T.; Abderrabba, M. Chemical and Antioxidant Properties of Betalains. *J. Agric. Food Chem.* **2017**, 65, 675–689.<br>Kumorkiewicz-Jamro, A.; Świergosz, T.; Sutor, K.; Spórna-Kucab, A.; Wybraniec, S. Multi-colored shades of betalains: Recent advances in betacyanin chemistry. *Nat. Prod. Rep.* **2021**, 38, 2315–2346.

Isoiresinin I

**Chemical structure**	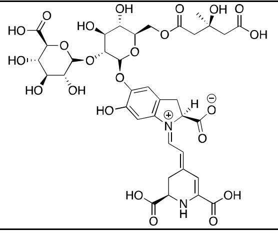
**Name:**	Isoiresinin I
**Chemical Formula**	$C_{36}H_{42}N_2O_{23}$
**Molecular weight**	870.72
**Monoisotopic mass**	870.2178
***m/z* [M+H]**	871.2251
**Theorical fragments (*m/z*)**	871.23 870.22 869.21 868.2 855.23 854.22 853.21 852.21 851.2 843.23<br>842.22 841.21 839.24 837.22 836.21 835.2 833.19 831.23 829.21<br>827.24 826.23 825.22 824.21 823.2 821.22 819.21 817.19 813.22<br>811.24 809.22 808.22 807.21 801.22 799.2 798.2 797.22 796.22<br>795.21 785.22 783.21 782.2 781.19 767.21 765.2 763.18 727.18<br>726.18 725.17 711.19 709.17 708.16 707.16 702.19 699.19 697.17<br>695.19 694.19 693.18 691.16 689.15 683.19 681.18 679.2 678.19<br>677.18 676.17 675.16 673.15 669.18 667.2 665.18 663.2 662.17<br>661.19 660.18 659.17 658.16 657.16 655.16 651.17 649.19 647.17<br>646.17 645.17 644.16 643.18 641.16 639.17 637.15 633.19 632.18<br>631.18 630.17 629.17 628.16 627.16 626.15 625.19 623.17 621.16<br>619.18 617.17 616.16 607.18 606.17 605.17 601.18 600.17 599.16<br>593.17 591.16 590.15 589.18 588.17 587.16 577.18 575.16 574.15<br>573.15 572.14 571.17 570.16 569.15 561.18 559.17 557.15 551.15<br>549.14 535.16 534.15 533.14 532.13 531.12 518.13 517.15 516.13<br>515.13 514.12 513.11 502.16 501.12 500.12 499.13 497.12 486.14<br>485.13 484.14 483.11 482.13 481.12 479.13 473.13 470.14 469.13<br>468.13 467.12 461.13 455.12 452.13 451.12 450.12 449.13 447.11<br>446.11 445.13 444.13 443.12 441.14 433.13 431.12 429.14 428.09<br>427.12 426.12 425.11 423.13 417.14 415.12 414.12 413.14 412.14<br>411.13 401.14 399.13 397.11 396.11 395.13 394.13 393.12 389.1<br>388.09 387.08 386.07 383.13 381.12 375.12 373.1 372.1 371.09<br>370.08 369.07 361.1 360.1 359.12 357.11 356.1 355.09 354.08<br>353.08 351.06 349.1 347.09 345.11 343.13 342.08 341.11 339.1<br>337.08 335.09 331.09 329.11 327.1 326.09 325.08 324.08 319.09<br>317.08 316.07 315.1 314.09 313.08 306.07 303.1 301.08 300.07<br>299.07 287.1 285.09 284.08 283.07 281.08 280.07 273.1 271.11<br>269.09 267.06 265.08 264.07 263.07 262.06 255.09 253.08 251.07<br>249.05 247.07 245.06 237.09 235.07 233.06 231.04 223.07 211.07<br>207.08 205.06 196.06 194.04 193.04 182.08 180.04 179.03 178.05<br>177.04 165.05 164.05 163.04 147.04 146.04 135.04 134.04 130.04<br>123.04 119.05 111.04 109.03 108.02 107.05 106.04
**References**	Minale, L.; Piattelli, M.; de Stefano, S.; Nicolaus, R.A. Pigments of Centrospermae VI. Acylated betacyanins. *Phytochemistry* **1966**, 5, 1037–1052.<br>Cai, Y.; Sun, M.; Corke, H. Identification and Distribution of Simple and Acylated Betacyanins in the Amaranthaceae. *J. Agric. Food Chem.* **2001**, 49, 1971–1978.<br>Slimen, I.B.; Najar, T.; Abderrabba, M. Chemical and Antioxidant Properties of Betalains. *J. Agric. Food Chem.* **2017**, 65, 675–689.<br>Kumorkiewicz-Jamro, A.; Świergosz, T.; Sutor, K.; Spórna-Kucab, A.; Wybraniec, S. Multi-colored shades of betalains: Recent advances in betacyanin chemistry. *Nat. Prod. Rep.* **2021**, 38, 2315–2346.

Celosianin I

**Chemical structure**	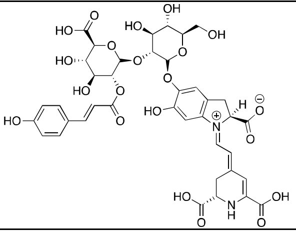
**Name:**	Celosianin I (argentianin)
**Chemical Formula**	$C_{39}H_{40}N_2O_{21}$
**Molecular weight**	872.74
**Monoisotopic mass**	872.2129
***m/z* [M+H]**	873.2196
**Theorical fragments (*m/z*)**	873.22 872.21 871.2 870.2 857.22 856.22 855.21 854.2 853.19 845.22<br>844.22 843.21 841.23 840.22 839.21 838.21 837.2 836.19 835.18<br>833.22 831.21 829.23 828.22 827.21 826.21 825.2 823.22 822.21<br>821.2 820.2 819.19 817.17 815.21 813.23 811.22 810.21 809.2<br>808.2 807.19 805.21 803.21 801.2 800.19 799.22 798.21 797.2<br>795.22 794.22 793.21 792.2 791.19 787.22 785.2 784.2 783.19<br>782.18 781.21 780.2 779.19 771.22 769.21 767.19 765.18 753.21<br>751.2 749.18 747.17 709.17 708.16 707.16 704.18 693.18 692.17<br>691.16 690.15 689.15 681.18 680.18 679.16 678.17 677.16 675.17<br>673.15 671.14 665.18 664.16 663.16 662.17 661.16 660.16 659.15<br>657.16 655.14 651.17 648.17 647.16 646.15 645.15 639.17 637.15<br>635.16 634.18 633.17 632.16 631.17 630.16 629.15 628.14 623.17<br>621.16 619.17 618.16 608.16 607.17 605.16 603.17 602.16 601.16<br>595.17 593.15 592.14 591.17 590.16 589.16 579.17 577.16 576.15<br>575.14 574.13 573.16 572.15 571.14 561.16 559.14 557.13 551.15<br>550.14 549.14 535.16 534.15 533.14 532.13 531.12 523.16 522.15<br>521.14 519.16 517.15 516.13 515.13 514.12 513.11 511.16 509.14<br>507.16 505.15 504.14 503.13 501.15 500.12 499.13 498.13 497.12<br>495.1 493.15 491.13 489.15 488.14 487.13 486.13 485.12 484.12<br>483.11 482.11 481.15 479.13 478.12 477.15 476.14 475.13 471.14<br>470.13 469.12 465.15 463.13 461.12 460.11 459.14 458.13 457.12<br>455.12 447.14 445.12 443.12 431.12 429.1 428.09 427.12 426.12<br>425.11 415.12 413.11 411.09 389.1 388.09 387.08 386.07 375.12<br>373.1 372.1 371.09 370.08 369.07 361.1 360.1 359.12 358.11<br>357.11 356.1 355.09 354.08 353.08 351.06 349.1 347.09 345.11<br>343.13 342.09 341.11 340.1 339.1 338.09 337.08 335.09 331.09<br>329.11 327.1 326.09 325.09 324.08 323.08 319.09 317.08 316.07<br>315.1 314.09 313.08 307.08 306.07 303.1 301.08 300.07 299.07<br>298.06 297.1 287.1 285.1 284.08 283.07 282.06 281.08 280.07<br>279.09 273.1 271.08 270.07 269.09 268.09 267.06 265.08 264.07<br>263.07 262.06 255.09 253.08 252.06 251.07 250.08 249.05 247.07<br>245.06 237.09 235.07 233.06 231.04 223.07 211.07 207.08 205.06<br>196.06 194.04 193.04 182.08 180.04 179.03 178.05 177.04 165.05<br>164.05 163.04 147.04 146.04 135.04 134.04 130.04 123.04 119.05<br>111.04 109.03 108.02 107.05 106.04
**References**	Cai, Y.; Sun, M.; Corke, H. Identification and Distribution of Simple and Acylated Betacyanins in the Amaranthaceae. *J. Agric. Food Chem.* **2001**, 49, 1971–1978.<br>Spórna-Kucab, A.; Wróbel, N.; Kumorkiewicz-Jamro, A.; Wybraniec, S. Separation of betacyanins from Iresine herbstii Hook. ex Lindl. leaves by high-speed countercurrent chromatography in a polar solvent system. *J. Chromatogr. A* **2020**, 1626, 461370.<br>Slimen, I.B.; Najar, T.; Abderrabba, M. Chemical and Antioxidant Properties of Betalains. *J. Agric. Food Chem.* **2017**, 65, 675–689.<br>Kumorkiewicz-Jamro, A.; Świergosz, T.; Sutor, K.; Spórna-Kucab, A.; Wybraniec, S. Multi-colored shades of betalains: Recent advances in betacyanin chemistry. *Nat. Prod. Rep.* **2021**, 38, 2315–2346.

Isocelosianin I

**Chemical structure**	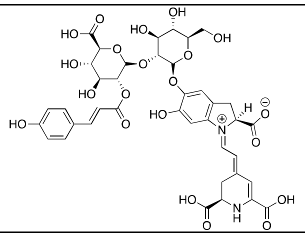
**Name:**	Isocelosianin I (isoargentianin)
**Chemical Formula**	$C_{39}H_{40}N_{2}O_{21}$
**Molecular weight**	872.74
**Monoisotopic mass**	872.2124
***m/z* [M+H]**	873.2196
**Theorical fragments (*m/z*)**	873.22 872.21 871.2 870.2 857.22 856.22 855.21 854.2 853.19 845.22<br>844.22 843.21 841.23 840.22 839.21 838.21 837.2 836.19 835.18<br>833.22 831.21 829.23 828.22 827.21 826.21 825.2 823.22 822.21<br>821.2 820.2 819.19 817.17 815.21 813.23 811.22 810.21 809.2<br>808.2 807.19 805.21 803.21 801.2 800.19 799.22 798.21 797.2<br>795.22 794.22 793.21 792.2 791.19 787.22 785.2 784.2 783.19<br>782.18 781.21 780.2 779.19 771.22 769.21 767.19 765.18 753.21<br>751.2 749.18 747.17 709.17 708.16 707.16 704.18 693.18 692.17<br>691.16 690.15 689.15 681.18 680.18 679.16 678.17 677.16 675.17<br>673.15 671.14 665.18 664.16 663.16 662.17 661.16 660.16 659.15<br>657.16 655.14 651.17 648.17 647.16 646.15 645.15 639.17 637.15<br>635.16 634.18 633.17 632.16 631.17 630.16 629.15 628.14 623.17<br>621.16 619.17 618.16 608.16 607.17 605.16 603.17 602.16 601.16<br>595.17 593.15 592.14 591.17 590.16 589.16 579.17 577.16 576.15<br>575.14 574.13 573.16 572.15 571.14 561.16 559.14 557.13 551.15<br>550.14 549.14 535.16 534.15 533.14 532.13 531.12 523.16 522.15<br>521.14 519.16 517.15 516.13 515.13 514.12 513.11 511.16 509.14<br>507.16 505.15 504.14 503.13 501.15 500.12 499.13 498.13 497.12<br>495.1 493.15 491.13 489.15 488.14 487.13 486.13 485.12 484.12<br>483.11 482.11 481.15 479.13 478.12 477.15 476.14 475.13 471.14<br>470.13 469.12 465.15 463.13 461.12 460.11 459.14 458.13 457.12<br>455.12 447.14 445.12 443.12 431.12 429.1 428.09 427.12 426.12<br>425.11 415.12 413.11 411.09 389.1 388.09 387.08 386.07 375.12<br>373.1 372.1 371.09 370.08 369.07 361.1 360.1 359.12 358.11<br>357.11 356.1 355.09 354.08 353.08 351.06 349.1 347.09 345.11<br>343.13 342.09 341.11 340.1 339.1 338.09 337.08 335.09 331.09<br>329.11 327.1 326.09 325.09 324.08 323.08 319.09 317.08 316.07<br>315.1 314.09 313.08 307.08 306.07 303.1 301.08 300.07 299.07<br>298.06 297.1 287.1 285.1 284.08 283.07 282.06 281.08 280.07<br>279.09 273.1 271.08 270.07 269.09 268.09 267.06 265.08 264.07<br>263.07 262.06 255.09 253.08 252.06 251.07 250.08 249.05 247.07<br>245.06 237.09 235.07 233.06 231.04 223.07 211.07 207.08 205.06<br>196.06 194.04 193.04 182.08 180.04 179.03 178.05 177.04 165.05<br>164.05 163.04 147.04 146.04 135.04 134.04 130.04 123.04 119.05<br>111.04 109.03 108.02 107.05 106.04
**References**	Cai, Y.; Sun, M.; Corke, H. Identification and Distribution of Simple and Acylated Betacyanins in the Amaranthaceae. *J. Agric. Food Chem.* **2001**, 49, 1971–1978.<br>Spórna-Kucab, A.; Wróbel, N.; Kumorkiewicz-Jamro, A.; Wybraniec, S. Separation of betacyanins from Iresine herbstii Hook. ex Lindl. leaves by high-speed countercurrent chromatography in a polar solvent system. *J. Chromatogr. A* **2020**, 1626, 461370.<br>Slimen, I.B.; Najar, T.; Abderrabba, M. Chemical and Antioxidant Properties of Betalains. *J. Agric. Food Chem.* **2017**, 65, 675–689.<br>Kumorkiewicz-Jamro, A.; Świergosz, T.; Sutor, K.; Spórna-Kucab, A.; Wybraniec, S. Multi-colored shades of betalains: Recent advances in betacyanin chemistry. *Nat. Prod. Rep.* **2021**, 38, 2315–2346.

Celosianin II

**Chemical structure**	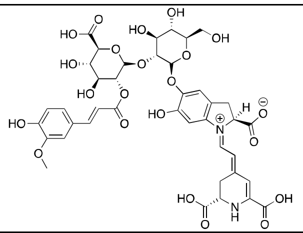
**Name:**	Celosianin II
**Chemical Formula**	$C_{40}H_{42}N_2O_{22}$
**Molecular weight**	902.77
**Monoisotopic mass**	902.2229
***m/z* [M+H]**	903.2302
**Theorical fragments (*m/z*)**	903.23 902.22 901.21 900.21 887.24 886.23 885.22 884.21 883.2 875.24 874.23 873.22 871.24 870.23 869.22 868.22 867.21 866.2 865.19 863.24 861.22 859.24 858.23 857.22 856.22 855.21 853.23 852.22 851.21 850.21 849.2 847.18 845.22 843.25 841.23 840.22 839.21 838.21 837.2 835.22 833.22 831.21 830.2 829.23 828.22 827.21 825.23 824.23 823.22 822.21 821.2 817.23 815.21 814.21 813.2 812.19 811.22 810.21 809.2 801.23 799.22 797.2 795.19 783.22 781.21 779.19 777.18 734.19 721.18 710.19 709.17 708.18 707.17 695.18 694.17 693.18 692.18 691.16 690.17 689.15 681.18 679.16 678.18 677.17 676.16 675.17 673.15 671.14 665.18 664.19 663.17 662.17 661.18 660.17 659.16 658.15 657.16 655.14 651.17 649.18 648.17 647.17 645.16 639.17 638.17 637.18 635.17 633.18 632.17 631.17 625.18 623.16 622.15 621.18 620.17 619.17 609.18 607.17 606.16 605.15 604.14 603.17 602.16 601.16 591.17 589.16 587.14 551.15 550.14 549.14 535.16 534.15 533.14 532.13 531.12 523.16 522.15 521.14 519.16 517.15 516.13 515.13 514.12 513.11 511.16 509.14 507.16 505.15 504.14 503.13 501.15 500.12 499.13 498.13 497.12 495.1 493.15 491.13 489.15 488.14 487.13 486.13 485.12 484.12 483.11 482.11 481.15 479.13 478.12 477.15 476.14 475.13 471.14 470.13 469.12 465.15 463.13 461.12 460.11 459.14 458.13 457.12 455.12 447.14 445.12 443.12 431.12 429.1 428.09 427.12 426.12 425.11 415.12 413.11 411.09 389.1 388.09 387.08 386.07 375.12 373.1 372.1 371.09 370.08 369.07 361.1 360.1 359.12 358.11 357.11 356.1 355.09 354.08 353.08 351.06 349.1 347.09 345.11 343.13 342.09 341.11 340.1 339.1 338.09 337.08 335.09 331.09 329.11 327.1 326.09 325.09 324.08 323.08 319.09 317.08 316.07 315.1 314.09 313.08 307.08 306.07 303.1 301.08 300.07 299.07 298.06 297.1 287.1 285.1 284.08 283.07 282.06 281.08 280.07 279.09 273.1 271.08 270.07 269.09 268.09 267.06 265.08 264.07 263.07 262.06 255.09 253.08 252.06 251.07 250.08 249.05 247.07 245.06 237.09 235.07 233.06 231.04 223.07 211.07 207.08 205.06 196.06 194.04 193.04 182.08 180.04 179.03 178.05 177.04 165.05 164.05 163.04 147.04 146.04 135.04 134.04 130.04 123.04 119.05 111.04 109.03 108.02 107.05 106.04
**References**	Strack, D.; Bokern, M.; Marxen, N.; Wray, V. Feruloylbetanin from petals of Lampranthus and feruloylamaranthin from cell suspension cultures of *Chenopodium rubrum*. *Phytochemistry* **1988**, 27, 3529–3531.<br>Cai, Y.; Sun, M.; Corke, H. Identification and Distribution of Simple and Acylated Betacyanins in the Amaranthaceae. *J. Agric. Food Chem.* **2001**, 49, 1971–1978.<br>Slimen, I.B.; Najar, T.; Abderrabba, M. Chemical and Antioxidant Properties of Betalains. *J. Agric. Food Chem.* **2017**, 65, 675–689.<br>Kumorkiewicz-Jamro, A.; Świergosz, T.; Sutor, K.; Spórna-Kucab, A.; Wybraniec, S. Multi-colored shades of betalains: Recent advances in betacyanin chemistry. *Nat. Prod. Rep.* **2021**, 38, 2315–2346.

Isocelosianin II

**Chemical structure**	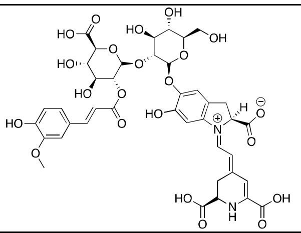
**Name:**	Isocelosianin II
**Chemical Formula**	$C_{40}H_{42}N_2O_{22}$
**Molecular weight**	902.77
**Monoisotopic mass**	902.2229
***m/z* [M+H]**	903.2302
**Theorical fragments (*m/z*)**	903.23 902.22 901.21 900.21 887.24 886.23 885.22 884.21 883.2 875.24<br>874.23 873.22 871.24 870.23 869.22 868.22 867.21 866.2 865.19<br>863.24 861.22 859.24 858.23 857.22 856.22 855.21 853.23 852.22<br>851.21 850.21 849.2 847.18 845.22 843.25 841.23 840.22 839.21<br>838.21 837.2 835.22 833.22 831.21 830.2 829.23 828.22 827.21<br>825.23 824.23 823.22 822.21 821.2 817.23 815.21 814.21 813.2<br>812.19 811.22 810.21 809.2 801.23 799.22 797.2 795.19 783.22<br>781.21 779.19 777.18 734.19 721.18 710.19 709.17 708.18 707.17<br>695.18 694.17 693.18 692.18 691.16 690.17 689.15 681.18 679.16<br>678.18 677.17 676.16 675.17 673.15 671.14 665.18 664.19 663.17<br>662.17 661.18 660.17 659.16 658.15 657.16 655.14 651.17 649.18<br>648.17 647.17 645.16 639.17 638.17 637.18 635.17 633.18 632.17<br>631.17 625.18 623.16 622.15 621.18 620.17 619.17 609.18 607.17<br>606.16 605.15 604.14 603.17 602.16 601.16 591.17 589.16 587.14<br>551.15 550.14 549.14 535.16 534.15 533.14 532.13 531.12 523.16<br>522.15 521.14 519.16 517.15 516.13 515.13 514.12 513.11 511.16<br>509.14 507.16 505.15 504.14 503.13 501.15 500.12 499.13 498.13<br>497.12 495.1 493.15 491.13 489.15 488.14 487.13 486.13 485.12<br>484.12 483.11 482.11 481.15 479.13 478.12 477.15 476.14 475.13<br>471.14 470.13 469.12 465.15 463.13 461.12 460.11 459.14 458.13<br>457.12 455.12 447.14 445.12 443.12 431.12 429.1 428.09 427.12<br>426.12 425.11 415.12 413.11 411.09 389.1 388.09 387.08 386.07<br>375.12 373.1 372.1 371.09 370.08 369.07 361.1 360.1 359.12<br>358.11 357.11 356.1 355.09 354.08 353.08 351.06 349.1 347.09<br>345.11 343.13 342.09 341.11 340.1 339.1 338.09 337.08 335.09<br>331.09 329.11 327.1 326.09 325.09 324.08 323.08 319.09 317.08<br>316.07 315.1 314.09 313.08 307.08 306.07 303.1 301.08 300.07<br>299.07 298.06 297.1 287.1 285.1 284.08 283.07 282.06 281.08<br>280.07 279.09 273.1 271.08 270.07 269.09 268.09 267.06 265.08<br>264.07 263.07 262.06 255.09 253.08 252.06 251.07 250.08 249.05<br>247.07 245.06 237.09 235.07 233.06 231.04 223.07 211.07 207.08<br>205.06 196.06 194.04 193.04 182.08 180.04 179.03 178.05 177.04<br>165.05 164.05 163.04 147.04 146.04 135.04 134.04 130.04 123.04<br>119.05 111.04 109.03 108.02 107.05 106.04
**References**	Strack, D.; Bokern, M.; Marxen, N.; Wray, V. Feruloylbetanin from petals of Lampranthus and feruloylamaranthin from cell suspension cultures of *Chenopodium rubrum*. *Phytochemistry* **1988**, 27, 3529–3531.<br>Cai, Y.; Sun, M.; Corke, H. Identification and Distribution of Simple and Acylated Betacyanins in the Amaranthaceae. *J. Agric. Food Chem.* **2001**, 49, 1971–1978.<br>Slimen, I.B.; Najar, T.; Abderrabba, M. Chemical and Antioxidant Properties of Betalains. *J. Agric. Food Chem.* **2017**, 65, 675–689.<br>Kumorkiewicz-Jamro, A.; Świergosz, T.; Sutor, K.; Spórna-Kucab, A.; Wybraniec, S. Multi-colored shades of betalains: Recent advances in betacyanin chemistry. *Nat. Prod. Rep.* **2021**, 38, 2315–2346.

Sinapoyl-amaranthin

**Chemical structure**	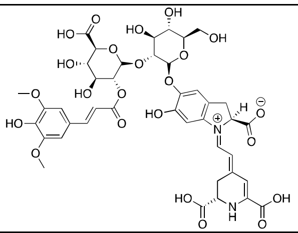
**Name:**	Sinapoyl-amaranthin
**Chemical Formula**	$C_{41}H_{44}N_2O_{23}$
**Molecular weight**	932.79
**Monoisotopic mass**	932.2335
***m/z* [M+H]**	933.2408
**Theorical fragments (*m/z*)**	933.24 932.23 931.23 930.22 917.25 916.24 915.23 914.22 913.21 905.25<br>904.24 903.23 901.25 900.24 899.24 898.23 897.22 896.21 895.2<br>893.25 891.23 889.25 888.24 887.24 886.23 885.22 883.24 882.23<br>881.22 880.22 879.21 877.19 875.24 873.26 871.24 870.23 869.22<br>868.22 867.21 865.23 863.24 861.22 860.21 859.24 858.23 857.22<br>855.25 854.24 853.23 852.22 851.21 847.24 845.22 844.22 843.21<br>842.2 841.23 840.22 839.21 831.25 829.23 827.21 825.2 813.23<br>811.22 809.2 807.19 764.2 751.2 740.2 738.19 737.18 725.19<br>724.18 723.18 722.19 721.18 720.18 719.17 709.17 708.19 707.16<br>706.17 705.17 695.18 694.2 693.18 692.18 691.16 690.18 689.15<br>688.16 681.18 679.19 678.18 677.18 675.17 673.15 671.14 668.18<br>667.19 665.18 663.17 662.18 661.18 659.17 657.16 655.19 653.17<br>652.16 651.19 650.18 649.18 647.17 645.16 639.19 637.15 636.17<br>635.16 634.15 633.18 632.17 631.17 623.17 621.16 619.14 617.15<br>605.16 603.15 601.13 551.15 550.14 549.14 535.16 534.15 533.14<br>532.13 531.12 523.16 522.15 521.14 519.16 517.15 516.13 515.13<br>514.12 513.11 511.16 509.14 507.16 505.15 504.14 503.13 501.15<br>500.12 499.13 498.13 497.12 495.1 493.15 491.13 489.15 488.14<br>487.13 486.13 485.12 484.12 483.11 482.11 481.15 479.13 478.12<br>477.15 476.14 475.13 471.14 470.13 469.12 465.15 463.13 461.12<br>460.11 459.14 458.13 457.12 455.12 447.14 445.12 443.12 431.12<br>429.1 428.09 427.12 426.12 425.11 415.12 413.11 411.09 389.1<br>388.09 387.08 386.07 375.12 373.1 372.1 371.09 370.08 369.07<br>361.1 360.1 359.12 358.11 357.11 356.1 355.09 354.08 353.08<br>351.06 349.1 347.09 345.11 343.13 342.09 341.11 340.1 339.1<br>338.09 337.08 335.09 331.09 329.11 327.1 326.09 325.09 324.08<br>323.08 319.09 317.08 316.07 315.1 314.09 313.08 307.08 306.07<br>303.1 301.08 300.07 299.07 298.06 297.1 287.1 285.1 284.08<br>283.07 282.06 281.08 280.07 279.09 273.1 271.08 270.07 269.09<br>268.09 267.06 265.08 264.07 263.07 262.06 255.09 253.08 252.06<br>251.07 250.08 249.05 247.07 245.06 237.09 235.07 233.06 231.04<br>223.07 211.07 207.08 205.06 196.06 194.04 193.04 182.08 180.04<br>179.03 178.05 177.04 165.05 164.05 163.04 147.04 146.04 135.04<br>134.04 130.04 123.04 119.05 111.04 109.03 108.02 107.05 106.04
**References**	Cai, Y.; Sun, M.; Corke, H. Identification and Distribution of Simple and Acylated Betacyanins in the Amaranthaceae. *J. Agric. Food Chem.* **2001**, 49, 1971–1978.<br>Slimen, I.B.; Najar, T.; Abderrabba, M. Chemical and Antioxidant Properties of Betalains. *J. Agric. Food Chem.* **2017**, 65, 675–689.<br>Kumorkiewicz-Jamro, A.; Świergosz, T.; Sutor, K.; Spórna-Kucab, A.; Wybraniec, S. Multi-colored shades of betalains: Recent advances in betacyanin chemistry. *Nat. Prod. Rep.* **2021**, 38, 2315–2346.

Sinapoyl-isoamaranthin

**Chemical structure**	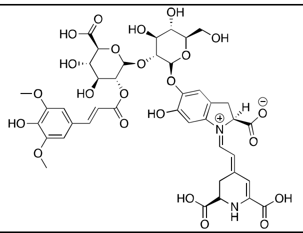
**Name:**	Sinapoyl-isoamaranthin
**Chemical Formula**	$C_{41}H_{44}N_2O_{23}$
**Molecular weight**	932.79
**Monoisotopic mass**	932.2335
***m/z* [M+H]**	933.2408
**Theorical fragments (*m/z*)**	933.24 932.23 931.23 930.22 917.25 916.24 915.23 914.22 913.21 905.25<br>904.24 903.23 901.25 900.24 899.24 898.23 897.22 896.21 895.2<br>893.25 891.23 889.25 888.24 887.24 886.23 885.22 883.24 882.23<br>881.22 880.22 879.21 877.19 875.24 873.26 871.24 870.23 869.22<br>868.22 867.21 865.23 863.24 861.22 860.21 859.24 858.23 857.22<br>855.25 854.24 853.23 852.22 851.21 847.24 845.22 844.22 843.21<br>842.2 841.23 840.22 839.21 831.25 829.23 827.21 825.2 813.23<br>811.22 809.2 807.19 764.2 751.2 740.2 738.19 737.18 725.19<br>724.18 723.18 722.19 721.18 720.18 719.17 709.17 708.19 707.16<br>706.17 705.17 695.18 694.2 693.18 692.18 691.16 690.18 689.15<br>688.16 681.18 679.19 678.18 677.18 675.17 673.15 671.14 668.18<br>667.19 665.18 663.17 662.18 661.18 659.17 657.16 655.19 653.17<br>652.16 651.19 650.18 649.18 647.17 645.16 639.19 637.15 636.17<br>635.16 634.15 633.18 632.17 631.17 623.17 621.16 619.14 617.15<br>605.16 603.15 601.13 551.15 550.14 549.14 535.16 534.15 533.14<br>532.13 531.12 523.16 522.15 521.14 519.16 517.15 516.13 515.13<br>514.12 513.11 511.16 509.14 507.16 505.15 504.14 503.13 501.15<br>500.12 499.13 498.13 497.12 495.1 493.15 491.13 489.15 488.14<br>487.13 486.13 485.12 484.12 483.11 482.11 481.15 479.13 478.12<br>477.15 476.14 475.13 471.14 470.13 469.12 465.15 463.13 461.12<br>460.11 459.14 458.13 457.12 455.12 447.14 445.12 443.12 431.12<br>429.1 428.09 427.12 426.12 425.11 415.12 413.11 411.09 389.1<br>388.09 387.08 386.07 375.12 373.1 372.1 371.09 370.08 369.07<br>361.1 360.1 359.12 358.11 357.11 356.1 355.09 354.08 353.08<br>351.06 349.1 347.09 345.11 343.13 342.09 341.11 340.1 339.1<br>338.09 337.08 335.09 331.09 329.11 327.1 326.09 325.09 324.08<br>323.08 319.09 317.08 316.07 315.1 314.09 313.08 307.08 306.07<br>303.1 301.08 300.07 299.07 298.06 297.1 287.1 285.1 284.08<br>283.07 282.06 281.08 280.07 279.09 273.1 271.08 270.07 269.09<br>268.09 267.06 265.08 264.07 263.07 262.06 255.09 253.08 252.06<br>251.07 250.08 249.05 247.07 245.06 237.09 235.07 233.06 231.04<br>223.07 211.07 207.08 205.06 196.06 194.04 193.04 182.08 180.04<br>179.03 178.05 177.04 165.05 164.05 163.04 147.04 146.04 135.04<br>134.04 130.04 123.04 119.05 111.04 109.03 108.02 107.05 106.04
**References**	Cai, Y.; Sun, M.; Corke, H. Identification and Distribution of Simple and Acylated Betacyanins in the Amaranthaceae. *J. Agric. Food Chem.* **2001**, 49, 1971–1978.<br>Slimen, I.B.; Najar, T.; Abderrabba, M. Chemical and Antioxidant Properties of Betalains. *J. Agric. Food Chem.* **2017**, 65, 675–689.<br>Kumorkiewicz-Jamro, A.; Świergosz, T.; Sutor, K.; Spórna-Kucab, A.; Wybraniec, S. Multi-colored shades of betalains: Recent advances in betacyanin chemistry. *Nat. Prod. Rep.* **2021**, 38, 2315–2346.

6´-*O*-malonyl-amaranthin

**Chemical structure**	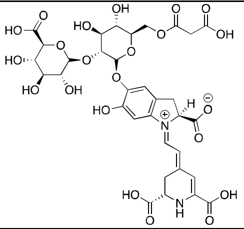
**Name:**	6´-*O*-malonyl-amaranthin (Celoscristatin)
**Chemical Formula**	$C_{33}H_{36}N_2O_{22}$
**Molecular weight**	812.64
**Monoisotopic mass**	812.1760
***m/z* [M+H]**	813.1832
**Theorical fragments (*m/z*)**	813.18 812.18 811.17 810.16 797.19 796.18 795.17 794.16 793.16 785.19<br>784.18 783.17 781.19 779.18 778.17 777.16 775.15 773.19 771.17<br>769.19 768.19 767.18 766.17 765.16 763.18 761.17 759.15 755.18<br>753.2 751.18 750.18 749.17 743.18 741.16 740.15 739.18 738.18<br>737.17 727.18 726.18 725.17 724.16 723.15 711.19 709.17 708.16<br>707.16 705.14 699.19 697.17 695.19 693.18 691.16 689.15 683.19<br>681.18 679.16 677.18 675.17 673.15 669.18 665.18 663.17 657.18<br>655.16 651.17 644.15 639.17 637.15 636.14 635.14 631.14 621.16<br>620.15 619.14 618.13 617.12 609.16 607.14 605.16 604.13 603.15<br>602.14 601.13 600.12 599.11 593.16 591.15 589.13 588.13 587.12<br>586.12 585.14 583.12 579.15 575.15 574.14 573.14 572.12 571.13<br>570.12 569.11 568.11 567.15 565.13 561.14 559.13 558.12 551.15<br>549.14 548.12 547.13 543.13 542.13 541.12 535.13 534.15 533.11<br>532.11 531.13 530.13 529.12 519.13 518.13 517.12 516.13 515.13<br>514.12 513.11 512.12 511.11 503.14 502.13 501.12 500.12 499.13<br>497.12 484.12 483.11 482.11 479.13 473.13 461.13 455.12 449.13<br>447.11 446.11 445.13 444.11 443.12 442.1 441.09 433.13 431.12<br>429.1 428.09 427.09 426.1 425.11 424.09 423.08 415.12 413.11<br>412.1 411.09 410.08 409.08 394.09 393.08 392.07 389.1 388.09<br>387.08 386.07 383.1 375.12 373.1 372.1 371.09 370.08 369.07<br>365.09 361.1 360.1 359.12 357.11 356.1 355.09 354.09 353.09<br>351.06 349.1 347.09 345.11 343.13 342.08 341.11 339.1 338.06<br>337.08 336.08 335.08 331.09 329.11 327.1 326.09 325.09 324.08<br>323.08 319.09 317.08 316.07 315.1 314.09 313.08 306.07 303.1<br>301.08 300.07 299.07 287.1 285.09 284.08 283.07 281.08 280.07<br>273.1 271.11 269.09 267.06 265.08 264.07 263.07 262.06 255.09<br>253.08 251.07 249.05 247.07 245.06 237.09 235.07 233.06 231.04<br>223.07 211.07 207.08 205.06 196.06 194.04 193.04 182.08 180.04<br>179.03 178.05 177.04 165.05 164.05 163.04 147.04 146.04 135.04<br>134.04 130.04 123.04 119.05 111.04 109.03 108.02 107.05 106.04
**References**	Lystvan, K.; Kumorkiewicz, A.; Szneler, E.; Wybraniec, S. Study on Betalains in Celosia Cristata Linn. Callus Culture and Identification of New Malonylated Amaranthins. *J. Agric. Food Chem.* **2018**, 66, 3870–3879.<br>Slimen, I.B.; Najar, T.; Abderrabba, M. Chemical and Antioxidant Properties of Betalains. *J. Agric. Food Chem.* **2017**, 65, 675–689.<br>Kumorkiewicz-Jamro, A.; Świergosz, T.; Sutor, K.; Spórna-Kucab, A.; Wybraniec, S. Multi-colored shades of betalains: Recent advances in betacyanin chemistry. *Nat. Prod. Rep.* **2021**, 38, 2315–2346.

6´-*O*-malonyl-isoamaranthin

**Chemical structure**	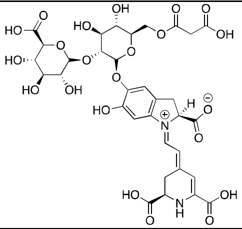
**Name:**	6´-*O*-malonyl-isoamaranthin (isoceloscristatin)
**Chemical Formula**	$C_{33}H_{36}N_2O_{22}$
**Molecular weight**	812.64
**Monoisotopic mass**	812.1760
***m/z* [M+H]**	813.1832
**Theorical fragments (*m/z*)**	813.18 812.18 811.17 810.16 797.19 796.18 795.17 794.16 793.16 785.19<br>784.18 783.17 781.19 779.18 778.17 777.16 775.15 773.19 771.17<br>769.19 768.19 767.18 766.17 765.16 763.18 761.17 759.15 755.18<br>753.2 751.18 750.18 749.17 743.18 741.16 740.15 739.18 738.18<br>737.17 727.18 726.18 725.17 724.16 723.15 711.19 709.17 708.16<br>707.16 705.14 699.19 697.17 695.19 693.18 691.16 689.15 683.19<br>681.18 679.16 677.18 675.17 673.15 669.18 665.18 663.17 657.18<br>655.16 651.17 644.15 639.17 637.15 636.14 635.14 631.14 621.16<br>620.15 619.14 618.13 617.12 609.16 607.14 605.16 604.13 603.15<br>602.14 601.13 600.12 599.11 593.16 591.15 589.13 588.13 587.12<br>586.12 585.14 583.12 579.15 575.15 574.14 573.14 572.12 571.13<br>570.12 569.11 568.11 567.15 565.13 561.14 559.13 558.12 551.15<br>549.14 548.12 547.13 543.13 542.13 541.12 535.13 534.15 533.11<br>532.11 531.13 530.13 529.12 519.13 518.13 517.12 516.13 515.13<br>514.12 513.11 512.12 511.11 503.14 502.13 501.12 500.12 499.13<br>497.12 484.12 483.11 482.11 479.13 473.13 461.13 455.12 449.13<br>447.11 446.11 445.13 444.11 443.12 442.1 441.09 433.13 431.12<br>429.1 428.09 427.09 426.1 425.11 424.09 423.08 415.12 413.11<br>412.1 411.09 410.08 409.08 394.09 393.08 392.07 389.1 388.09<br>387.08 386.07 383.1 375.12 373.1 372.1 371.09 370.08 369.07<br>365.09 361.1 360.1 359.12 357.11 356.1 355.09 354.09 353.09<br>351.06 349.1 347.09 345.11 343.13 342.08 341.11 339.1 338.06<br>337.08 336.08 335.08 331.09 329.11 327.1 326.09 325.09 324.08<br>323.08 319.09 317.08 316.07 315.1 314.09 313.08 306.07 303.1<br>301.08 300.07 299.07 287.1 285.09 284.08 283.07 281.08 280.07<br>273.1 271.11 269.09 267.06 265.08 264.07 263.07 262.06 255.09<br>253.08 251.07 249.05 247.07 245.06 237.09 235.07 233.06 231.04<br>223.07 211.07 207.08 205.06 196.06 194.04 193.04 182.08 180.04<br>179.03 178.05 177.04 165.05 164.05 163.04 147.04 146.04 135.04<br>134.04 130.04 123.04 119.05 111.04 109.03 108.02 107.05 106.04
**References**	Lystvan, K.; Kumorkiewicz, A.; Szneler, E.; Wybraniec, S. Study on Betalains in Celosia Cristata Linn. Callus Culture and Identification of New Malonylated Amaranthins. *J. Agric. Food Chem.* **2018**, 66, 3870–3879.<br>Slimen, I.B.; Najar, T.; Abderrabba, M. Chemical and Antioxidant Properties of Betalains. *J. Agric. Food Chem.* **2017**, 65, 675–689.<br>Kumorkiewicz-Jamro, A.; Świergosz, T.; Sutor, K.; Spórna-Kucab, A.; Wybraniec, S. Multi-colored shades of betalains: Recent advances in betacyanin chemistry. *Nat. Prod. Rep.* **2021**, 38, 2315–2346.

4´-*O*-malonyl-amaranthin

**Chemical structure**	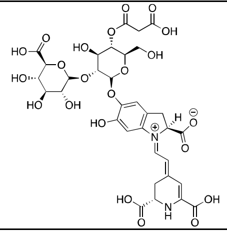
**Name:**	4´-*O*-malonyl-amaranthin
**Chemical Formula**	$C_{33}H_{36}N_2O_{22}$
**Molecular weight**	812.64
**Monoisotopic mass**	812.1760
***m/z* [M+H]**	813.1832
**Theorical fragments (*m/z*)**	813.18 812.18 811.17 810.16 797.19 796.18 795.17 794.16 793.16 785.19<br>784.18 783.17 781.19 779.18 778.17 777.16 775.15 773.19 771.17<br>769.19 768.19 767.18 766.17 765.16 763.18 761.17 759.15 755.18<br>753.2 751.18 750.18 749.17 743.18 741.16 740.15 739.18 738.18<br>737.17 727.18 726.18 725.17 724.16 723.15 711.19 709.17 708.16<br>707.16 705.14 699.19 697.17 695.19 693.18 691.16 689.15 683.19<br>681.18 679.16 677.18 675.17 673.15 669.18 665.18 663.17 657.18<br>655.16 651.17 644.15 639.17 637.15 636.14 635.14 631.14 621.16<br>620.15 619.14 618.13 617.12 609.16 607.14 605.16 604.13 603.15<br>602.14 601.13 600.12 599.11 593.16 591.15 589.13 588.13 587.12<br>586.12 585.14 583.12 579.15 575.15 574.14 573.14 572.12 571.13<br>570.12 569.11 568.11 567.15 565.13 561.14 559.13 558.12 551.15<br>549.14 548.12 547.13 543.13 542.13 541.12 535.13 534.15 533.11<br>532.11 531.13 530.13 529.12 519.13 518.13 517.12 516.13 515.13<br>514.12 513.11 512.12 511.11 503.14 502.13 501.12 500.12 499.13<br>497.12 484.12 483.11 482.11 479.13 473.13 461.13 455.12 449.13<br>447.11 446.11 445.13 444.11 443.12 442.1 441.09 433.13 431.12<br>429.1 428.09 427.09 426.1 425.11 424.09 423.08 415.12 413.11<br>412.1 411.09 410.08 409.08 394.09 393.08 392.07 389.1 388.09<br>387.08 386.07 383.1 375.12 373.1 372.1 371.09 370.08 369.07<br>365.09 361.1 360.1 359.12 357.11 356.1 355.09 354.09 353.09<br>351.06 349.1 347.09 345.11 343.13 342.08 341.11 339.1 338.06<br>337.08 336.08 335.08 331.09 329.11 327.1 326.09 325.09 324.08<br>323.08 319.09 317.08 316.07 315.1 314.09 313.08 306.07 303.1<br>301.08 300.07 299.07 287.1 285.09 284.08 283.07 281.08 280.07<br>273.1 271.11 269.09 267.06 265.08 264.07 263.07 262.06 255.09<br>253.08 251.07 249.05 247.07 245.06 237.09 235.07 233.06 231.04<br>223.07 211.07 207.08 205.06 196.06 194.04 193.04 182.08 180.04<br>179.03 178.05 177.04 165.05 164.05 163.04 147.04 146.04 135.04<br>134.04 130.04 123.04 119.05 111.04 109.03 108.02 107.05 106.04
**References**	Lystvan, K.; Kumorkiewicz, A.; Szneler, E.; Wybraniec, S. Study on Betalains in Celosia Cristata Linn. Callus Culture and Identification of New Malonylated Amaranthins. *J. Agric. Food Chem.* **2018**, 66, 3870–3879.<br>Slimen, I.B.; Najar, T.; Abderrabba, M. Chemical and Antioxidant Properties of Betalains. *J. Agric. Food Chem.* **2017**, 65, 675–689.<br>Kumorkiewicz-Jamro, A.; Świergosz, T.; Sutor, K.; Spórna-Kucab, A.; Wybraniec, S. Multi-colored shades of betalains: Recent advances in betacyanin chemistry. *Nat. Prod. Rep.* **2021**, 38, 2315–2346.

4´-*O*-malonyl-isoamaranthin

**Chemical structure**	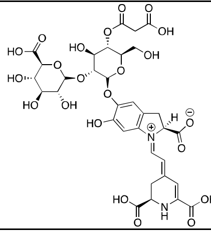
**Name:**	4´-*O*-malonyl-amaranthin
**Chemical Formula**	$C_{33}H_{36}N_2O_{22}$
**Molecular weight**	812.64
**Monoisotopic mass**	812.1760
***m/z* [M+H]**	813.1832
**Theorical fragments (*m/z*)**	813.18 812.18 811.17 810.16 797.19 796.18 795.17 794.16 793.16 785.19<br>784.18 783.17 781.19 779.18 778.17 777.16 775.15 773.19 771.17<br>769.19 768.19 767.18 766.17 765.16 763.18 761.17 759.15 755.18<br>753.2 751.18 750.18 749.17 743.18 741.16 740.15 739.18 738.18<br>737.17 727.18 726.18 725.17 724.16 723.15 711.19 709.17 708.16<br>707.16 705.14 699.19 697.17 695.19 693.18 691.16 689.15 683.19<br>681.18 679.16 677.18 675.17 673.15 669.18 665.18 663.17 657.18<br>655.16 651.17 644.15 639.17 637.15 636.14 635.14 631.14 621.16<br>620.15 619.14 618.13 617.12 609.16 607.14 605.16 604.13 603.15<br>602.14 601.13 600.12 599.11 593.16 591.15 589.13 588.13 587.12<br>586.12 585.14 583.12 579.15 575.15 574.14 573.14 572.12 571.13<br>570.12 569.11 568.11 567.15 565.13 561.14 559.13 558.12 551.15<br>549.14 548.12 547.13 543.13 542.13 541.12 535.13 534.15 533.11<br>532.11 531.13 530.13 529.12 519.13 518.13 517.12 516.13 515.13<br>514.12 513.11 512.12 511.11 503.14 502.13 501.12 500.12 499.13<br>497.12 484.12 483.11 482.11 479.13 473.13 461.13 455.12 449.13<br>447.11 446.11 445.13 444.11 443.12 442.1 441.09 433.13 431.12<br>429.1 428.09 427.09 426.1 425.11 424.09 423.08 415.12 413.11<br>412.1 411.09 410.08 409.08 394.09 393.08 392.07 389.1 388.09<br>387.08 386.07 383.1 375.12 373.1 372.1 371.09 370.08 369.07<br>365.09 361.1 360.1 359.12 357.11 356.1 355.09 354.09 353.09<br>351.06 349.1 347.09 345.11 343.13 342.08 341.11 339.1 338.06<br>337.08 336.08 335.08 331.09 329.11 327.1 326.09 325.09 324.08<br>323.08 319.09 317.08 316.07 315.1 314.09 313.08 306.07 303.1<br>301.08 300.07 299.07 287.1 285.09 284.08 283.07 281.08 280.07<br>273.1 271.11 269.09 267.06 265.08 264.07 263.07 262.06 255.09<br>253.08 251.07 249.05 247.07 245.06 237.09 235.07 233.06 231.04<br>223.07 211.07 207.08 205.06 196.06 194.04 193.04 182.08 180.04<br>179.03 178.05 177.04 165.05 164.05 163.04 147.04 146.04 135.04<br>134.04 130.04 123.04 119.05 111.04 109.03 108.02 107.05 106.04
**References**	Lystvan, K.; Kumorkiewicz, A.; Szneler, E.; Wybraniec, S. Study on Betalains in Celosia Cristata Linn. Callus Culture and Identification of New Malonylated Amaranthins. *J. Agric. Food Chem.* **2018**, 66, 3870–3879.<br>Slimen, I.B.; Najar, T.; Abderrabba, M. Chemical and Antioxidant Properties of Betalains. *J. Agric. Food Chem.* **2017**, 65, 675–689.<br>Kumorkiewicz-Jamro, A.; Świergosz, T.; Sutor, K.; Spórna-Kucab, A.; Wybraniec, S. Multi-colored shades of betalains: Recent advances in betacyanin chemistry. *Nat. Prod. Rep.* **2021**, 38, 2315–2346.

17-decarboxy-amaranthin

Chemical structure	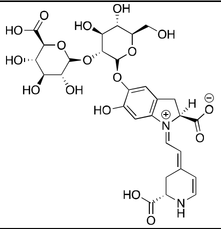
**Name:**	17-decarboxy-amaranthin
**Chemical Formula**	$C_{29}H_{34}N_2O_{17}$
**Molecular weight**	682.59
**Monoisotopic mass**	682.1857
m/z **[M+H]**	683.1930
**Theorical fragments (*m/z*)**	683.19 682.19 681.18 680.17 667.2 666.19 665.18 664.17 663.17 655.2 654.19 653.18 651.2 650.2 649.19 648.18 647.17 646.16 645.16 643.2 641.18 639.2 638.2 637.19 636.18 635.17 633.19 632.18 631.18 630.17 629.16 627.15 625.19 623.21 621.19 620.18 619.18 618.17 617.16 615.18 613.19 611.17 610.16 609.19 608.18 607.18 605.2 604.19 603.18 602.17 601.17 597.19 595.18 594.17 593.16 592.15 591.18 590.17 589.17 581.2 579.18 577.17 575.15 563.19 561.17 559.16 558.15 557.14 545.14 534.15 532.13 531.12 519.13 518.13 517.12 516.13 515.13 514.12 513.11 507.16 506.15 505.15 502.13 501.12 500.12 499.11 491.17 490.16 489.15 488.14 487.13 486.12 485.13 484.12 483.11 482.11 479.17 478.16 477.15 475.17 473.16 472.12 471.14 470.13 469.12 467.17 465.15 463.17 462.12 461.13 460.15 459.14 457.16 456.13 455.14 454.14 453.13 451.11 449.13 447.11 446.11 445.13 444.13 443.12 442.14 441.13 437.16 435.14 434.13 433.13 432.15 431.12 430.11 429.1 428.09 427.12 426.12 425.11 421.16 419.14 417.13 416.12 415.12 414.14 413.11 411.09 403.15 401.13 399.12 385.14 383.12 381.11 358.11 356.1 355.09 345.11 344.1 343.09 342.09 341.09 340.1 338.09 337.08 331.13 329.11 328.11 327.1 326.09 325.08 324.08 323.08 317.11 316.11 315.13 313.12 312.11 311.1 310.09 309.09 307.08 306.07 305.11 303.1 301.12 299.14 298.09 297.12 295.11 293.09 291.1 287.1 285.1 283.11 282.1 281.09 279.09 275.1 273.09 272.08 271.08 270.07 269.09 268.09 267.09 259.11 257.09 256.08 255.09 254.07 253.07 252.06 250.08 249.08 243.11 241.1 240.09 239.08 238.07 237.09 236.08 235.06 227.12 225.1 223.07 221.09 220.08 219.08 218.07 209.09 207.08 205.06 203.08 201.07 196.06 194.04 193.1 191.08 189.07 187.05 182.08 180.04 179.08 178.05 177.04 167.08 165.05 164.05 163.09 161.07 152.07 150.05 147.04 146.04 135.04 134.04 130.04 123.04 119.05 111.04 109.03 108.02 107.05 106.04
**References**	Miguel, M.G. Betalains in Some Species of the Amaranthaceae Family: A Review. *Antioxidants* **2018**, 7, 53.<br>Xie, G.R.; Chen, H.J. Comprehensive Betalain Profiling of Djulis (*Chenopodium formosanum*) Cultivars Using HPLC-Q-Orbitrap High-Resolution Mass Spectrometry. *J. Agric. Food Chem.* **2021**, 69, 15699–15715.<br>Araujo-León, J.A.; Aguilar-Hernández, V.; Sánchez-del Pino, I.; Brito-Argáez, L.; Peraza-Sánchez, S.R.; Xingú-López, A.; Ortiz-Andrade, R. Analysis of Red Amaranth (*Amaranthus cruentus* L.) Betalains by LC-MS. *J. Mex. Chem. Soc.* **2023**, 67, 227–239.

15-decarboxy-amaranthin

**Chemical structure**	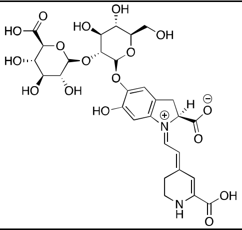
**Name:**	15-decarboxy-amaranthin
**Chemical Formula**	$C_{29}H_{34}N_2O_{17}$
**Molecular weight**	682.59
**Monoisotopic mass**	682.1857
***m/z* [M+H]**	683.1930
**Theorical fragments (*m/z*)**	683.19 682.19 681.18 680.17 667.2 666.19 665.18 664.17 663.17 655.2 654.19 653.18 651.2 650.2 649.19 648.18 647.17 646.16 645.16 643.2 641.18 639.2 638.2 637.19 636.18 635.17 633.19 632.18 631.18 630.17 629.16 627.15 625.19 623.21 621.19 620.18 619.18 618.17 617.16 615.18 613.19 611.17 610.16 609.19 608.18 607.18 605.2 604.19 603.18 602.17 601.17 597.19 595.18 594.17 593.16 592.15 591.18 590.17 589.17 581.2 579.18 577.17 575.15 563.19 561.17 559.16 558.15 557.14 545.14 534.15 532.13 531.12 519.13 518.13 517.12 516.13 515.13 514.12 513.11 507.16 506.15 505.15 502.13 501.12 500.12 499.11 491.17 490.16 489.15 488.14 487.13 486.12 485.13 484.12 483.11 482.11 479.17 478.16 477.15 475.17 473.16 472.12 471.14 470.13 469.12 467.17 465.15 463.17 462.12 461.13 460.15 459.14 457.16 456.13 455.14 454.14 453.13 451.11 449.13 447.11 446.11 445.13 444.13 443.12 442.14 441.13 437.16 435.14 434.13 433.13 432.15 431.12 430.11 429.1 428.09 427.12 426.12 425.11 421.16 419.14 417.13 416.12 415.12 414.14 413.11 411.09 403.15 401.13 399.12 385.14 383.12 381.11 358.11 356.1 355.09 345.11 344.1 343.09 342.09 341.09 340.1 338.09 337.08 331.13 329.11 328.11 327.1 326.09 325.08 324.08 323.08 317.11 316.11 315.13 313.12 312.11 311.1 310.09 309.09 307.08 306.07 305.11 303.1 301.12 299.14 298.09 297.12 295.11 293.09 291.1 287.1 285.1 283.11 282.1 281.09 279.09 275.1 273.09 272.08 271.08 270.07 269.09 268.09 267.09 259.11 257.09 256.08 255.09 254.07 253.07 252.06 250.08 249.08 243.11 241.1 240.09 239.08 238.07 237.09 236.08 235.06 227.12 225.1 223.07 221.09 220.08 219.08 218.07 209.09 207.08 205.06 203.08 201.07 196.06 194.04 193.1 191.08 189.07 187.05 182.08 180.04 179.08 178.05 177.04 167.08 165.05 164.05 163.09 161.07 152.07 150.05 147.04 146.04 135.04 134.04 130.04 123.04 119.05 111.04 109.03 108.02 107.05 106.04
**References**	Miguel, M.G. Betalains in Some Species of the Amaranthaceae Family: A Review. *Antioxidants* **2018**, 7, 53.<br>Xie, G.R.; Chen, H.J. Comprehensive Betalain Profiling of Djulis (*Chenopodium formosanum*) Cultivars Using HPLC-Q-Orbitrap High-Resolution Mass Spectrometry. *J. Agric. Food Chem.* **2021**, 69, 15699–15715.<br>Araujo-León, J.A.; Aguilar-Hernández, V.; Sánchez-del Pino, I.; Brito-Argáez, L.; Peraza-Sánchez, S.R.; Xingú-López, A.; Ortiz-Andrade, R. Analysis of Red Amaranth (*Amaranthus cruentus* L.) Betalains by LC-MS. *J. Mex. Chem. Soc.* **2023**, 67, 227–239.

2-decarboxy-amaranthin

**Chemical structure**	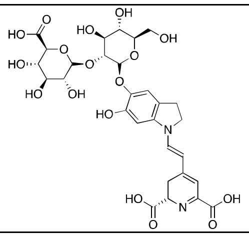
**Name:**	2-decarboxy-amaranthin
**Chemical Formula**	$C_{29}H_{34}N_2O_{17}$
**Molecular weight**	682.59
**Monoisotopic mass**	682.1857
***m/z* [M+H]**	683.1930
**Theorical fragments (*m/z*)**	683.19 682.19 681.18 680.17 667.2 666.19 665.18 664.17 663.17 655.2<br>654.19 653.18 651.2 650.2 649.19 648.18 647.17 646.16 645.16<br>643.2 641.18 639.2 638.2 637.19 636.18 635.17 633.19 632.18<br>631.18 630.17 629.16 627.15 625.19 623.21 621.19 620.18 619.18<br>618.17 617.16 615.18 613.19 611.17 610.16 609.19 608.18 607.18<br>605.2 604.19 603.18 602.17 601.17 597.19 595.18 594.17 593.16<br>592.15 591.18 590.17 589.17 581.2 579.18 577.17 575.15 563.19<br>561.17 559.16 558.15 557.14 545.14 534.15 532.13 531.12 519.13<br>518.13 517.12 516.13 515.13 514.12 513.11 507.16 506.15 505.15<br>502.13 501.12 500.12 499.11 491.17 490.16 489.15 488.14 487.13<br>486.12 485.13 484.12 483.11 482.11 479.17 478.16 477.15 475.17<br>473.16 472.12 471.14 470.13 469.12 467.17 465.15 463.17 462.12<br>461.13 460.15 459.14 457.16 456.13 455.14 454.14 453.13 451.11<br>449.13 447.11 446.11 445.13 444.13 443.12 442.14 441.13 437.16<br>435.14 434.13 433.13 432.15 431.12 430.11 429.1 428.09 427.12<br>426.12 425.11 421.16 419.14 417.13 416.12 415.12 414.14 413.11<br>411.09 403.15 401.13 399.12 385.14 383.12 381.11 358.11 356.1<br>355.09 345.11 344.1 343.09 342.09 341.09 340.1 338.09 337.08<br>331.13 329.11 328.11 327.1 326.09 325.08 324.08 323.08 317.11<br>316.11 315.13 313.12 312.11 311.1 310.09 309.09 307.08 306.07<br>305.11 303.1 301.12 299.14 298.09 297.12 295.11 293.09 291.1<br>287.1 285.1 283.11 282.1 281.09 279.09 275.1 273.09 272.08<br>271.08 270.07 269.09 268.09 267.09 259.11 257.09 256.08 255.09<br>254.07 253.07 252.06 250.08 249.08 243.11 241.1 240.09 239.08<br>238.07 237.09 236.08 235.06 227.12 225.1 223.07 221.09 220.08<br>219.08 218.07 209.09 207.08 205.06 203.08 201.07 196.06 194.04<br>193.1 191.08 189.07 187.05 182.08 180.04 179.08 178.05 177.04<br>167.08 165.05 164.05 163.09 161.07 152.07 150.05 147.04 146.04<br>135.04 134.04 130.04 123.04 119.05 111.04 109.03 108.02 107.05<br>106.04
**References**	Miguel, M.G. Betalains in Some Species of the Amaranthaceae Family: A Review. *Antioxidants* **2018**, 7, 53.<br>Xie, G.R.; Chen, H.J. Comprehensive Betalain Profiling of Djulis (*Chenopodium formosanum*) Cultivars Using HPLC-Q-Orbitrap High-Resolution Mass Spectrometry. *J. Agric. Food Chem.* **2021**, 69, 15699–15715.<br>Araujo-León, J.A.; Aguilar-Hernández, V.; Sánchez-del Pino, I.; Brito-Argáez, L.; Peraza-Sánchez, S.R.; Xingú-López, A.; Ortiz-Andrade, R. Analysis of Red Amaranth (*Amaranthus cruentus* L.) Betalains by LC-MS. *J. Mex. Chem. Soc.* **2023**, 67, 227–239.

Neoamaranthin

**Chemical structure**	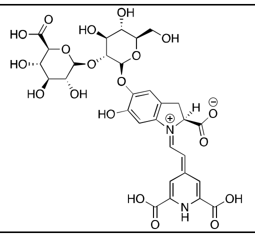
**Name:**	Neoamaranthin
**Chemical Formula**	$C_{30}H_{32}N_2O_{19}$
**Molecular weight**	724.58
**Monoisotopic mass**	724.1599
***m/z* [M+H]**	725.1672
**Theorical fragments (*m/z*)**	725.17 724.16 723.15 722.14 709.17 708.16 707.16 706.15 705.14 697.17 696.16 695.16 693.18 692.17 691.16 690.15 689.15 688.14 687.13 685.17 683.16 682.15 681.18 680.17 679.16 678.15 677.15 675.17 674.16 673.15 672.14 671.14 669.12 667.16 665.18 663.17 662.16 661.15 660.14 659.14 657.16 655.16 653.15 652.14 651.17 650.16 649.15 647.17 646.16 645.16 644.15 643.14 639.17 637.15 636.14 635.14 634.13 633.16 632.15 631.14 621.16 619.14 617.12 603.15 601.13 599.11 549.14 548.13 547.12 545.14 533.14 532.13 531.12 530.12 529.11 528.13 521.14 520.13 519.12 518.13 517.12 516.13 515.13 514.12 513.11 512.11 511.1 509.14 507.12 505.15 503.13 502.13 501.12 500.12 499.11 498.12 497.12 496.11 495.1 493.09 491.13 489.12 488.14 487.13 486.12 485.12 484.12 483.11 482.11 479.13 477.11 476.11 475.11 474.13 473.13 472.12 470.13 469.12 468.12 467.11 461.13 459.1 458.1 457.13 456.13 455.12 449.13 447.11 446.11 445.13 444.13 443.12 441.09 433.13 431.12 430.11 429.1 428.09 427.12 426.12 425.11 423.08 415.12 413.11 411.09 387.08 386.07 385.07 384.06 373.1 371.09 370.08 369.07 368.06 367.06 359.09 358.08 357.11 356.1 355.09 354.08 353.08 352.07 347.09 345.07 343.09 342.09 341.08 340.07 339.1 338.09 337.08 333.07 329.08 327.1 325.08 324.08 323.08 320.08 317.08 315.06 314.05 313.08 312.07 307.08 306.07 301.08 299.07 298.06 297.1 294.1 285.1 283.07 282.06 279.06 278.05 273.1 271.08 270.07 268.09 267.09 265.05 263.07 261.05 255.09 253.07 252.06 251.07 250.08 249.05 247.03 237.08 235.07 233.06 231.04 221.06 209.06 207.05 194.04 193.04 192.03 191.06 181.05 180.04 179.03 178.05 177.04 167.07 165.05 164.05 163.04 150.05 147.04 146.04 135.04 134.04 130.04 123.04 119.05 111.04 109.03 108.02 107.05 106.04
**References**	Miguel, M.G. Betalains in Some Species of the Amaranthaceae Family: A Review. *Antioxidants* **2018**, 7, 53.<br>Xie, G.R.; Chen, H.J. Comprehensive Betalain Profiling of Djulis (*Chenopodium formosanum*) Cultivars Using HPLC-Q-Orbitrap High-Resolution Mass Spectrometry. *J. Agric. Food Chem.* **2021**, 69, 15699–15715.<br>Araujo-León, J.A.; Aguilar-Hernández, V.; Sánchez-del Pino, I.; Brito-Argáez, L.; Peraza-Sánchez, S.R.; Xingú-López, A.; Ortiz-Andrade, R. Analysis of Red Amaranth (*Amaranthus cruentus* L.) Betalains by LC-MS. *J. Mex. Chem. Soc.* **2023**, 67, 227–239.

Melocactin-type
Melocactin

**Chemical structure**	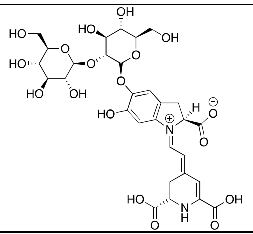
**Name:**	Melocactin (2'-O-glycosyl- betanin / Bougainvillein-r)
**Chemical Formula**	$C_{30}H_{36}N_{2}O_{18}$
**Molecular weight**	712.61
**Monoisotopic mass**	712.1963
***m/z* [M+H]**	713.2036
**Theorical fragments (*m/z*)**	713.2 712.2 711.19 710.18 697.21 696.2 695.19 694.19 693.18 685.21<br>684.2 683.19 681.21 679.2 678.19 677.18 676.17 675.17 673.21<br>671.19 669.21 668.21 667.2 666.19 665.18 663.2 661.19 660.18<br>659.17 657.16 655.2 653.22 651.2 650.2 649.19 648.18 647.17<br>643.2 641.18 640.17 639.2 638.2 637.19 633.19 632.18 631.18<br>627.2 625.19 624.18 623.17 622.16 621.19 620.18 619.18 609.19<br>607.18 605.16 591.18 589.17 587.15 551.15 550.14 549.14 544.17<br>535.16 534.15 533.14 532.13 531.12 523.16 522.15 521.14 520.17<br>519.16 518.15 517.14 516.14 515.13 514.12 513.11 511.16 509.14<br>507.16 505.15 504.15 503.14 502.16 501.15 500.14 499.13 498.13<br>497.12 495.1 493.15 491.13 489.15 488.14 487.14 486.14 485.13<br>481.15 479.13 478.12 477.15 476.14 475.13 474.16 473.15 472.14<br>471.14 470.13 469.13 468.13 465.15 463.13 461.12 460.11 459.15<br>458.14 457.12 448.14 447.15 445.12 443.11 442.15 441.14 435.15<br>433.13 432.13 430.15 429.14 427.11 425.1 417.14 416.13 415.12<br>414.12 412.14 411.13 399.13 397.11 389.1 388.09 387.08 386.07<br>375.12 373.1 372.1 371.09 370.08 369.07 361.1 360.1 359.12<br>358.11 357.11 356.1 355.09 354.08 353.08 351.06 349.1 347.09<br>345.11 343.13 342.09 341.11 340.1 339.1 338.09 337.08 335.09<br>331.09 329.11 327.1 326.09 325.09 324.08 323.08 319.09 317.08<br>316.07 315.1 314.09 313.08 307.08 306.07 303.1 301.08 300.07<br>299.07 297.1 287.1 285.1 284.08 283.07 281.08 280.07 279.09<br>273.1 271.08 270.07 269.09 268.09 267.06 265.08 264.07 263.07<br>262.06 255.09 253.08 252.06 251.07 250.08 249.05 247.07 245.06<br>237.09 235.07 233.06 231.04 223.07 211.07 207.08 205.06 196.06<br>194.04 193.04 182.08 180.04 179.03 178.05 177.04 165.05 164.05<br>163.04 147.04 146.04 135.04 134.04 130.04 123.04 119.05 111.04<br>109.03 108.02 107.05 106.04
**References**	Piattelli, M; Imperato, F. Betacyanins from Bougainvillea. *Phytochemistry* **1970**, 9, 455–458.<br>Wybraniec, S.; Nowak-Wydra, B.; Mitka, K.; Kowalski, P.; Mizrahi, Y. Minor betalains in fruits of *Hylocereus* species. *Phytochemistry* **2007**, 68, 251–259.<br>Slimen, I.B.; Najar, T.; Abderrabba, M. Chemical and Antioxidant Properties of Betalains. *J. Agric. Food Chem.* **2017**, 65, 675–689.<br>Kumorkiewicz-Jamro, A.; Świergosz, T.; Sutor, K.; Spórna-Kucab, A.; Wybraniec, S. Multi-colored shades of betalains: Recent advances in betacyanin chemistry. *Nat. Prod. Rep.* **2021**, 38, 2315–2346.

Isomelocactin

**Chemical structure**	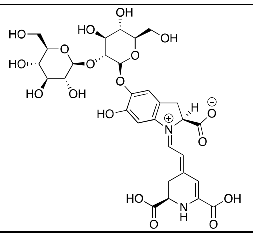
**Name:**	Isomelocactin (2'-O-glycosyl- isobetanin / isobougainvillein-r)
**Chemical Formula**	$C_{30}H_{36}N_2O_{18}$
**Molecular weight**	712.61
**Monoisotopic mass**	712.1963
***m/z* [M+H]**	713.2036
**Theorical fragments (*m/z*)**	713.2 712.2 711.19 710.18 697.21 696.2 695.19 694.19 693.18 685.21<br>684.2 683.19 681.21 679.2 678.19 677.18 676.17 675.17 673.21<br>671.19 669.21 668.21 667.2 666.19 665.18 663.2 661.19 660.18<br>659.17 657.16 655.2 653.22 651.2 650.2 649.19 648.18 647.17<br>643.2 641.18 640.17 639.2 638.2 637.19 633.19 632.18 631.18<br>627.2 625.19 624.18 623.17 622.16 621.19 620.18 619.18 609.19<br>607.18 605.16 591.18 589.17 587.15 551.15 550.14 549.14 544.17<br>535.16 534.15 533.14 532.13 531.12 523.16 522.15 521.14 520.17<br>519.16 518.15 517.14 516.14 515.13 514.12 513.11 511.16 509.14<br>507.16 505.15 504.15 503.14 502.16 501.15 500.14 499.13 498.13<br>497.12 495.1 493.15 491.13 489.15 488.14 487.14 486.14 485.13<br>481.15 479.13 478.12 477.15 476.14 475.13 474.16 473.15 472.14<br>471.14 470.13 469.13 468.13 465.15 463.13 461.12 460.11 459.15<br>458.14 457.12 448.14 447.15 445.12 443.11 442.15 441.14 435.15<br>433.13 432.13 430.15 429.14 427.11 425.1 417.14 416.13 415.12<br>414.12 412.14 411.13 399.13 397.11 389.1 388.09 387.08 386.07<br>375.12 373.1 372.1 371.09 370.08 369.07 361.1 360.1 359.12<br>358.11 357.11 356.1 355.09 354.08 353.08 351.06 349.1 347.09<br>345.11 343.13 342.09 341.11 340.1 339.1 338.09 337.08 335.09<br>331.09 329.11 327.1 326.09 325.09 324.08 323.08 319.09 317.08<br>316.07 315.1 314.09 313.08 307.08 306.07 303.1 301.08 300.07<br>299.07 297.1 287.1 285.1 284.08 283.07 281.08 280.07 279.09<br>273.1 271.08 270.07 269.09 268.09 267.06 265.08 264.07 263.07<br>262.06 255.09 253.08 252.06 251.07 250.08 249.05 247.07 245.06<br>237.09 235.07 233.06 231.04 223.07 211.07 207.08 205.06 196.06<br>194.04 193.04 182.08 180.04 179.03 178.05 177.04 165.05 164.05<br>163.04 147.04 146.04 135.04 134.04 130.04 123.04 119.05 111.04<br>109.03 108.02 107.05 106.04
**References**	Piattelli, M; Imperato, F. Betacyanins from Bougainvillea. *Phytochemistry* **1970**, 9, 455–458.<br>Wybraniec, S.; Nowak-Wydra, B.; Mitka, K.; Kowalski, P.; Mizrahi, Y. Minor betalains in fruits of *Hylocereus* species. *Phytochemistry* **2007**, 68, 251–259.<br>Slimen, I.B.; Najar, T.; Abderrabba, M. Chemical and Antioxidant Properties of Betalains. *J. Agric. Food Chem.* **2017**, 65, 675–689.<br>Kumorkiewicz-Jamro, A.; Świergosz, T.; Sutor, K.; Spórna-Kucab, A.; Wybraniec, S. Multi-colored shades of betalains: Recent advances in betacyanin chemistry. *Nat. Prod. Rep.* **2021**, 38, 2315–2346.

Bougainvillein-r III

**Chemical structure**	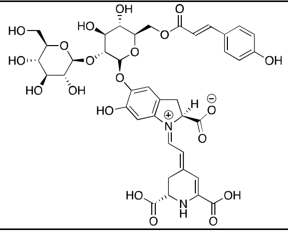
**Name:**	Bougainvillein-r III
**Chemical Formula**	$C_{39}H_{42}N_{2}O_{20}$
**Molecular weight**	858.76
**Monoisotopic mass**	858.2331
***m/z* [M+H]**	859.2404
**Theorical fragments (*m/z*)**	859.24 858.23 857.22 856.22 843.25 842.24 841.23 840.22 839.21 831.25<br>830.24 829.23 827.25 825.23 824.23 823.22 822.21 821.2 819.25<br>817.23 815.25 814.24 813.23 812.23 811.22 809.24 807.22 806.22<br>805.21 803.19 801.23 799.26 797.24 796.23 795.22 794.22 793.21<br>789.23 787.22 786.21 785.24 784.23 783.22 779.23 778.22 777.21<br>773.24 771.22 770.22 769.21 768.2 767.23 766.22 765.21 755.23<br>753.21 751.2 737.22 735.2 733.19 697.19 696.18 695.19 694.19<br>693.18 690.2 681.19 680.18 679.18 678.17 677.16 676.17 675.17<br>669.19 668.18 667.18 666.2 665.18 664.19 663.18 662.17 661.17<br>660.16 659.15 657.19 655.18 653.2 651.18 650.18 649.18 648.19<br>647.19 646.18 645.17 644.16 643.16 641.14 639.18 637.19 635.19<br>634.18 633.18 632.17 631.17 627.18 625.17 624.16 623.17 622.18<br>621.17 620.2 619.19 618.18 617.18 616.17 615.17 614.16 611.19<br>609.17 607.16 606.15 605.19 604.18 603.16 594.18 593.19 591.16<br>589.15 588.18 587.18 581.19 579.17 578.16 576.18 575.18 573.15<br>571.13 563.18 562.17 561.16 560.15 558.17 557.17 545.17 543.15<br>533.14 532.13 531.12 517.15 515.13 514.12 513.11 505.15 504.15<br>503.13 502.13 501.13 500.14 499.13 497.12 495.1 488.13 487.12<br>486.14 485.13 484.12 483.12 481.12 479.11 475.13 471.13 470.12<br>469.11 468.13 463.13 461.12 457.12 453.12 452.11 445.12 443.11<br>441.14 431.13 429.14 427.11 425.12 419.13 417.14 416.11 415.12<br>414.13 413.12 412.14 411.13 401.12 399.11 398.1 397.11 396.12<br>395.11 389.1 388.09 387.08 386.07 383.11 381.1 375.12 373.1<br>372.1 371.09 370.08 369.07 361.1 360.1 359.12 357.11 356.1<br>355.09 354.08 353.08 351.06 349.1 347.09 345.11 343.13 342.08<br>341.11 339.1 337.08 335.09 331.09 329.11 327.1 326.09 325.08<br>324.08 319.09 317.08 316.07 315.1 314.09 313.08 306.07 303.1<br>301.08 300.07 299.07 287.1 285.09 284.08 283.07 281.08 280.07<br>271.11 269.09 267.06 265.08 264.07 263.07 262.06 255.09 253.08<br>251.07 249.05 247.07 245.06 237.09 235.07 233.06 231.04 223.07<br>211.07 207.08 205.06 196.06 194.04 193.04 182.08 180.04 179.03<br>178.05 177.04 165.05 164.05 163.04 147.04 146.04 135.04 134.04<br>130.04 123.04 119.05 111.04 109.03 108.02 107.05 106.04
**References**	Piattelli, M; Imperato, F. Betacyanins from Bougainvillea. *Phytochemistry* **1970**, 9, 455–458.<br>Slimen, I.B.; Najar, T.; Abderrabba, M. Chemical and Antioxidant Properties of Betalains. *J. Agric. Food Chem.* **2017**, 65, 675–689.<br>Kumorkiewicz-Jamro, A.; Świergosz, T.; Sutor, K.; Spórna-Kucab, A.; Wybraniec, S. Multi-colored shades of betalains: Recent advances in betacyanin chemistry. *Nat. Prod. Rep.* **2021**, 38, 2315–2346.

Isobougainvillein-r III

**Chemical structure**	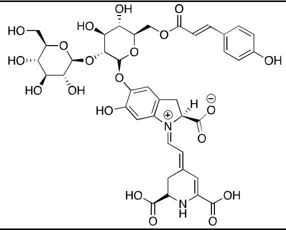
**Name:**	Isobougainvillein-r III
**Chemical Formula**	$C_{39}H_{42}N_2O_{20}$
**Molecular weight**	858.76
**Monoisotopic mass**	858.2331
***m/z* [M+H]**	859.2404
**Theorical fragments (*m/z*)**	859.24 858.23 857.22 856.22 843.25 842.24 841.23 840.22 839.21 831.25<br>830.24 829.23 827.25 825.23 824.23 823.22 822.21 821.2 819.25<br>817.23 815.25 814.24 813.23 812.23 811.22 809.24 807.22 806.22<br>805.21 803.19 801.23 799.26 797.24 796.23 795.22 794.22 793.21<br>789.23 787.22 786.21 785.24 784.23 783.22 779.23 778.22 777.21<br>773.24 771.22 770.22 769.21 768.2 767.23 766.22 765.21 755.23<br>753.21 751.2 737.22 735.2 733.19 697.19 696.18 695.19 694.19<br>693.18 690.2 681.19 680.18 679.18 678.17 677.16 676.17 675.17<br>669.19 668.18 667.18 666.2 665.18 664.19 663.18 662.17 661.17<br>660.16 659.15 657.19 655.18 653.2 651.18 650.18 649.18 648.19<br>647.19 646.18 645.17 644.16 643.16 641.14 639.18 637.19 635.19<br>634.18 633.18 632.17 631.17 627.18 625.17 624.16 623.17 622.18<br>621.17 620.2 619.19 618.18 617.18 616.17 615.17 614.16 611.19<br>609.17 607.16 606.15 605.19 604.18 603.16 594.18 593.19 591.16<br>589.15 588.18 587.18 581.19 579.17 578.16 576.18 575.18 573.15<br>571.13 563.18 562.17 561.16 560.15 558.17 557.17 545.17 543.15<br>533.14 532.13 531.12 517.15 515.13 514.12 513.11 505.15 504.15<br>503.13 502.13 501.13 500.14 499.13 497.12 495.1 488.13 487.12<br>486.14 485.13 484.12 483.12 481.12 479.11 475.13 471.13 470.12<br>469.11 468.13 463.13 461.12 457.12 453.12 452.11 445.12 443.11<br>441.14 431.13 429.14 427.11 425.12 419.13 417.14 416.11 415.12<br>414.13 413.12 412.14 411.13 401.12 399.11 398.1 397.11 396.12<br>395.11 389.1 388.09 387.08 386.07 383.11 381.1 375.12 373.1<br>372.1 371.09 370.08 369.07 361.1 360.1 359.12 357.11 356.1<br>355.09 354.08 353.08 351.06 349.1 347.09 345.11 343.13 342.08<br>341.11 339.1 337.08 335.09 331.09 329.11 327.1 326.09 325.08<br>324.08 319.09 317.08 316.07 315.1 314.09 313.08 306.07 303.1<br>301.08 300.07 299.07 287.1 285.09 284.08 283.07 281.08 280.07<br>271.11 269.09 267.06 265.08 264.07 263.07 262.06 255.09 253.08<br>251.07 249.05 247.07 245.06 237.09 235.07 233.06 231.04 223.07<br>211.07 207.08 205.06 196.06 194.04 193.04 182.08 180.04 179.03<br>178.05 177.04 165.05 164.05 163.04 147.04 146.04 135.04 134.04<br>130.04 123.04 119.05 111.04 109.03 108.02 107.05 106.04
**References**	Piattelli, M; Imperato, F. Betacyanins from Bougainvillea. *Phytochemistry* **1970**, 9, 455–458.<br>Slimen, I.B.; Najar, T.; Abderrabba, M. Chemical and Antioxidant Properties of Betalains. *J. Agric. Food Chem.* **2017**, 65, 675–689.<br>Kumorkiewicz-Jamro, A.; Świergosz, T.; Sutor, K.; Spórna-Kucab, A.; Wybraniec, S. Multi-colored shades of betalains: Recent advances in betacyanin chemistry. *Nat. Prod. Rep.* **2021**, 38, 2315–2346.

Feruloyl-bougainvillein-r I

**Chemical structure**	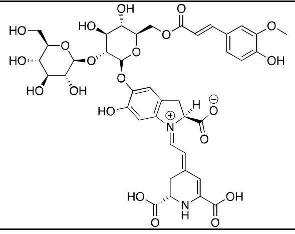
**Name:**	Feruloyl-bougainvillein-r I
**Chemical Formula**	$C_{40}H_{44}N_2O_{21}$
**Molecular weight**	888.79
**Monoisotopic mass**	888.2437
***m/z* [M+H]**	889.2509
**Theorical fragments (*m/z*)**	889.25 888.24 887.24 886.23 873.26 872.25 871.24 870.23 869.22 861.26<br>860.25 859.24 857.26 855.25 854.24 853.23 852.22 851.21 849.26<br>847.24 845.26 844.25 843.25 842.24 841.23 839.25 837.23 836.23<br>835.22 833.2 831.25 829.27 827.25 826.24 825.23 824.23 823.22<br>819.25 817.23 816.22 815.25 814.24 813.23 809.24 808.23 807.22<br>803.25 801.23 800.23 799.22 798.21 797.24 796.23 795.22 785.24<br>783.22 781.21 767.23 765.21 763.2 727.2 726.19 725.18 720.21<br>711.2 710.2 709.19 708.18 707.17 699.2 698.2 697.19 696.21<br>695.19 694.2 693.19 692.18 691.18 690.17 689.16 687.2 685.19<br>683.21 681.19 680.19 679.2 678.2 677.18 676.19 675.18 674.17<br>673.17 671.15 669.19 667.2 666.19 665.18 664.19 663.19 662.18<br>661.19 660.18 659.17 657.19 655.18 654.17 653.2 652.19 651.18<br>650.21 649.19 648.19 647.19 646.18 645.18 644.17 643.18 641.2<br>639.18 637.17 636.16 635.2 634.19 633.17 632.18 631.18 625.19<br>624.19 623.2 622.16 621.17 620.18 619.16 618.19 617.19 611.2<br>609.18 608.17 607.18 606.19 605.19 603.16 601.15 593.19 592.18<br>591.17 590.16 589.17 588.18 587.18 575.18 573.16 534.16 533.14<br>532.14 531.12 518.14 517.15 516.15 515.13 514.13 513.11 505.15<br>503.13 502.16 501.14 500.13 499.13 497.12 495.1 487.13 486.14<br>485.13 483.13 482.12 481.12 479.11 475.13 473.14 469.13 468.13<br>463.13 461.14 457.12 455.13 449.14 447.13 446.12 445.12 444.14<br>443.13 441.14 431.13 429.14 428.11 427.11 426.13 425.12 417.14<br>415.12 414.12 413.12 412.14 411.13 399.13 397.11 389.1 388.09<br>387.08 386.07 375.12 373.1 372.1 371.09 370.08 369.07 361.1<br>360.1 359.12 357.11 356.1 355.09 354.08 353.08 351.06 349.1<br>347.09 345.11 343.13 342.08 341.11 339.1 337.08 335.09 331.09<br>329.11 327.1 326.09 325.08 324.08 319.09 317.08 316.07 315.1<br>314.09 313.08 306.07 303.1 301.08 300.07 299.07 287.1 285.09<br>284.08 283.07 281.08 280.07 271.11 269.09 267.06 265.08 264.07<br>263.07 262.06 255.09 253.08 251.07 249.05 247.07 245.06 237.09<br>235.07 233.06 231.04 223.07 211.07 207.08 205.06 196.06 194.04<br>193.04 182.08 180.04 179.03 178.05 177.04 165.05 164.05 163.04<br>147.04 146.04 135.04 134.04 130.04 123.04 119.05 111.04 109.03<br>108.02 107.05 106.04
**References**	Vogt, T.; Ibdah, M.; Schmidt, J.; Wray, V.; Nimtz, M.; Strack, D. Light-induced betacyanin and flavonol accumulation in bladder cells of *Mesembryanthemum crystallinum*. *Phytochemistry* **1999**, 52, 583–592.<br>Kugler, F.; Stintzing, F.C.; Carle, R. Identification of betalains from petioles of differently colored Swiss chard (*Beta vulgaris* L. ssp. *cicla* Alef. Cv. Bright Lights) by high-performance liquid chromatography-electrospray ionization mass spectrometry. *J. Agric. Food Chem.* **2004**, 52, 2975–2981.<br>Svenson, J.; Smallfield, B.M.; Joyce, N.I.; Sanson, C.E.; Perry, N.B. Betalains in red and yellow varieties of the Andean tuber crop ulluco (*Ullucus tuberosus*). *J. Agric. Food Chem.* **2008**, 56, 7730–7737.<br>Slimen, I.B.; Najar, T.; Abderrabba, M. Chemical and Antioxidant Properties of Betalains. *J. Agric. Food Chem.* **2017**, 65, 675–689.

	Kumorkiewicz-Jamro, A.; Świergosz, T.; Sutor, K.; Spórna-Kucab, A.; Wybraniec, S. Multi-colored shades of betalains: Recent advances in betacyanin chemistry. *Nat. Prod. Rep.* **2021**, 38, 2315–2346.

Feruloyl-isobougainvillein-r I

**Chemical structure**	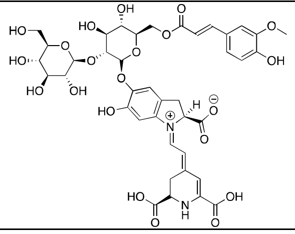
**Name:**	Feruloyl-isobougainvillein-r I
**Chemical Formula**	$C_{40}H_{44}N_{2}O_{21}$
**Molecular weight**	888.79
**Monoisotopic mass**	888.2437
***m/z* [M+H]**	889.2509
**Theorical fragments (*m/z*)**	889.25 888.24 887.24 886.23 873.26 872.25 871.24 870.23 869.22 861.26<br>860.25 859.24 857.26 855.25 854.24 853.23 852.22 851.21 849.26<br>847.24 845.26 844.25 843.25 842.24 841.23 839.25 837.23 836.23<br>835.22 833.2 831.25 829.27 827.25 826.24 825.23 824.23 823.22<br>819.25 817.23 816.22 815.25 814.24 813.23 809.24 808.23 807.22<br>803.25 801.23 800.23 799.22 798.21 797.24 796.23 795.22 785.24<br>783.22 781.21 767.23 765.21 763.2 727.2 726.19 725.18 720.21<br>711.2 710.2 709.19 708.18 707.17 699.2 698.2 697.19 696.21<br>695.19 694.2 693.19 692.18 691.18 690.17 689.16 687.2 685.19<br>683.21 681.19 680.19 679.2 678.2 677.18 676.19 675.18 674.17<br>673.17 671.15 669.19 667.2 666.19 665.18 664.19 663.19 662.18<br>661.19 660.18 659.17 657.19 655.18 654.17 653.2 652.19 651.18<br>650.21 649.19 648.19 647.19 646.18 645.18 644.17 643.18 641.2<br>639.18 637.17 636.16 635.2 634.19 633.17 632.18 631.18 625.19<br>624.19 623.2 622.16 621.17 620.18 619.16 618.19 617.19 611.2<br>609.18 608.17 607.18 606.19 605.19 603.16 601.15 593.19 592.18<br>591.17 590.16 589.17 588.18 587.18 575.18 573.16 534.16 533.14<br>532.14 531.12 518.14 517.15 516.15 515.13 514.13 513.11 505.15<br>503.13 502.16 501.14 500.13 499.13 497.12 495.1 487.13 486.14<br>485.13 483.13 482.12 481.12 479.11 475.13 473.14 469.13 468.13<br>463.13 461.14 457.12 455.13 449.14 447.13 446.12 445.12 444.14<br>443.13 441.14 431.13 429.14 428.11 427.11 426.13 425.12 417.14<br>415.12 414.12 413.12 412.14 411.13 399.13 397.11 389.1 388.09<br>387.08 386.07 375.12 373.1 372.1 371.09 370.08 369.07 361.1<br>360.1 359.12 357.11 356.1 355.09 354.08 353.08 351.06 349.1<br>347.09 345.11 343.13 342.08 341.11 339.1 337.08 335.09 331.09<br>329.11 327.1 326.09 325.08 324.08 319.09 317.08 316.07 315.1<br>314.09 313.08 306.07 303.1 301.08 300.07 299.07 287.1 285.09<br>284.08 283.07 281.08 280.07 271.11 269.09 267.06 265.08 264.07<br>263.07 262.06 255.09 253.08 251.07 249.05 247.07 245.06 237.09<br>235.07 233.06 231.04 223.07 211.07 207.08 205.06 196.06 194.04<br>193.04 182.08 180.04 179.03 178.05 177.04 165.05 164.05 163.04<br>147.04 146.04 135.04 134.04 130.04 123.04 119.05 111.04 109.03<br>108.02 107.05 106.04
**References**	Vogt, T.; Ibdah, M.; Schmidt, J.; Wray, V.; Nimtz, M.; Strack, D. Light-induced betacyanin and flavonol accumulation in bladder cells of *Mesembryanthemum crystallinum*. *Phytochemistry* **1999**, 52, 583–592.<br>Kugler, F.; Stintzing, F.C.; Carle, R. Identification of betalains from petioles of differently colored Swiss chard (*Beta vulgaris* L. ssp. *cicla* Alef. Cv. Bright Lights) by high-performance liquid chromatography-electrospray ionization mass spectrometry. *J. Agric. Food Chem.* **2004**, 52, 2975–2981.<br>Svenson, J.; Smallfield, B.M.; Joyce, N.I.; Sanson, C.E.; Perry, N.B. Betalains in red and yellow varieties of the Andean tuber crop ulluco (*Ullucus tuberosus*). *J. Agric. Food Chem.* **2008**, 56, 7730–7737.<br>Slimen, I.B.; Najar, T.; Abderrabba, M. Chemical and Antioxidant Properties of Betalains. *J. Agric. Food Chem.* **2017**, 65, 675–689.

	Kumorkiewicz-Jamro, A.; Świergosz, T.; Sutor, K.; Spórna-Kucab, A.; Wybraniec, S. Multi-colored shades of betalains: Recent advances in betacyanin chemistry. *Nat. Prod. Rep.* **2021**, 38, 2315–2346.

Mammillarinin

**Chemical structure**	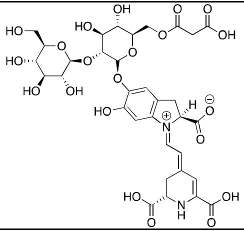
**Name:**	Mammillarinin
**Chemical Formula**	$C_{33}H_{38}N_2O_{21}$
**Molecular weight**	798.66
**Monoisotopic mass**	798.1967
***m/z* [M+H]**	799.2040
**Theorical fragments (*m/z*)**	799.2 798.2 797.19 796.18 783.21 782.2 781.19 780.19 779.18 771.21 770.2 769.19 767.21 765.2 764.19 763.18 761.17 759.21 757.19 755.21 754.21 753.2 752.19 751.18 749.2 747.19 745.17 741.2 739.22 737.2 736.2 735.19 729.2 727.18 726.18 725.2 724.2 723.19 713.2 712.2 711.19 710.18 709.17 697.21 695.19 694.19 693.18 691.16 685.21 683.19 681.21 679.2 677.18 675.17 669.21 667.2 665.18 663.2 661.19 659.17 655.2 651.2 649.19 643.2 641.18 637.15 636.14 635.14 630.17 625.19 623.17 621.16 619.14 618.13 617.12 609.16 607.14 606.17 605.16 604.15 603.14 601.13 599.11 593.16 591.15 590.15 589.14 588.16 587.15 586.14 585.14 583.12 579.15 575.15 574.15 573.15 572.14 571.13 567.15 565.13 561.14 560.16 559.15 558.15 557.15 556.14 555.13 554.13 551.15 549.14 547.12 545.15 544.14 535.16 534.15 533.15 531.12 529.11 528.15 527.14 521.15 520.17 519.13 518.13 517.16 516.15 515.13 513.11 505.16 504.15 503.14 502.16 501.12 500.14 499.13 498.14 497.12 487.14 486.14 485.13 479.13 469.13 468.13 461.12 459.15 447.15 444.11 443.11 442.1 441.09 435.15 433.13 432.13 430.15 429.14 428.09 427.09 426.1 424.09 423.08 417.14 415.12 414.12 412.1 411.09 410.08 409.08 399.13 394.09 393.08 392.07 389.1 388.09 387.08 386.07 383.1 375.12 373.1 372.1 371.09 370.08 369.07 365.09 361.1 360.1 359.12 357.11 356.1 355.09 354.09 353.09 351.06 349.1 347.09 345.11 343.13 342.08 341.11 339.1 338.06 337.08 336.08 335.08 331.09 329.11 327.1 326.09 325.09 324.08 323.08 319.09 317.08 316.07 315.1 314.09 313.08 306.07 303.1 301.08 300.07 299.07 287.1 285.09 284.08 283.07 281.08 280.07 273.1 271.11 269.09 267.06 265.08 264.07 263.07 262.06 255.09 253.08 251.07 249.05 247.07 245.06 237.09 235.07 233.06 231.04 223.07 211.07 207.08 205.06 196.06 194.04 193.04 182.08 180.04 179.03 178.05 177.04 165.05 164.05 163.04 147.04 146.04 135.04 134.04 130.04 123.04 119.05 111.04 109.03 108.02 107.05 106.04
**References**	Wybraniec, S.; Nowak-Wydra, B. Mammillarinin—A new malonylated betacyanin in fruits of Mammillaria. *J. Agric. Food Chem.* **2007**, 55, 8138–8143.<br>Slimen, I.B.; Najar, T.; Abderrabba, M. Chemical and Antioxidant Properties of Betalains. *J. Agric. Food Chem.* **2017**, 65, 675–689.<br>Kumorkiewicz-Jamro, A.; Świergosz, T.; Sutor, K.; Spórna-Kucab, A.; Wybraniec, S. Multi-colored shades of betalains: Recent advances in betacyanin chemistry. *Nat. Prod. Rep.* **2021**, 38, 2315–2346.

Isomammillarinin

**Chemical structure**	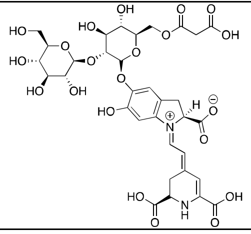
**Name:**	Isomammillarinin
**Chemical Formula**	$C_{33}H_{38}N_2O_{21}$
**Molecular weight**	798.66
**Monoisotopic mass**	798.1967
***m/z* [M+H]**	799.2040
**Theorical fragments (*m/z*)**	799.2 798.2 797.19 796.18 783.21 782.2 781.19 780.19 779.18 771.21<br>770.2 769.19 767.21 765.2 764.19 763.18 761.17 759.21 757.19<br>755.21 754.21 753.2 752.19 751.18 749.2 747.19 745.17 741.2<br>739.22 737.2 736.2 735.19 729.2 727.18 726.18 725.2 724.2<br>723.19 713.2 712.2 711.19 710.18 709.17 697.21 695.19 694.19<br>693.18 691.16 685.21 683.19 681.21 679.2 677.18 675.17 669.21<br>667.2 665.18 663.2 661.19 659.17 655.2 651.2 649.19 643.2<br>641.18 637.15 636.14 635.14 630.17 625.19 623.17 621.16 619.14<br>618.13 617.12 609.16 607.14 606.17 605.16 604.15 603.14 601.13<br>599.11 593.16 591.15 590.15 589.14 588.16 587.15 586.14 585.14<br>583.12 579.15 575.15 574.15 573.15 572.14 571.13 567.15 565.13<br>561.14 560.16 559.15 558.15 557.15 556.14 555.13 554.13 551.15<br>549.14 547.12 545.15 544.14 535.16 534.15 533.15 531.12 529.11<br>528.15 527.14 521.15 520.17 519.13 518.13 517.16 516.15 515.13<br>513.11 505.16 504.15 503.14 502.16 501.12 500.14 499.13 498.14<br>497.12 487.14 486.14 485.13 479.13 469.13 468.13 461.12 459.15<br>447.15 444.11 443.11 442.1 441.09 435.15 433.13 432.13 430.15<br>429.14 428.09 427.09 426.1 424.09 423.08 417.14 415.12 414.12<br>412.1 411.09 410.08 409.08 399.13 394.09 393.08 392.07 389.1<br>388.09 387.08 386.07 383.1 375.12 373.1 372.1 371.09 370.08<br>369.07 365.09 361.1 360.1 359.12 357.11 356.1 355.09 354.09<br>353.09 351.06 349.1 347.09 345.11 343.13 342.08 341.11 339.1<br>338.06 337.08 336.08 335.08 331.09 329.11 327.1 326.09 325.09<br>324.08 323.08 319.09 317.08 316.07 315.1 314.09 313.08 306.07<br>303.1 301.08 300.07 299.07 287.1 285.09 284.08 283.07 281.08<br>280.07 273.1 271.11 269.09 267.06 265.08 264.07 263.07 262.06<br>255.09 253.08 251.07 249.05 247.07 245.06 237.09 235.07 233.06<br>231.04 223.07 211.07 207.08 205.06 196.06 194.04 193.04 182.08<br>180.04 179.03 178.05 177.04 165.05 164.05 163.04 147.04 146.04<br>135.04 134.04 130.04 123.04 119.05 111.04 109.03 108.02 107.05<br>106.04
**References**	Wybraniec, S.; Nowak-Wydra, B. Mammillarinin—A new malonylated betacyanin in fruits of Mammillaria. *J. Agric. Food Chem.* **2007**, 55, 8138–8143.<br>Slimen, I.B.; Najar, T.; Abderrabba, M. Chemical and Antioxidant Properties of Betalains. *J. Agric. Food Chem.* **2017**, 65, 675–689.<br>Kumorkiewicz-Jamro, A.; Świergosz, T.; Sutor, K.; Spórna-Kucab, A.; Wybraniec, S. Multi-colored shades of betalains: Recent advances in betacyanin chemistry. *Nat. Prod. Rep.* **2021**, 38, 2315–2346.

4’-*O*-Malonyl-bougainvillein-r I

**Chemical structure**	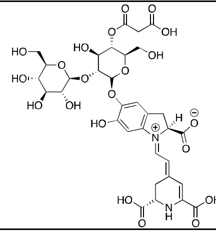
**Name:**	4’-*O*-Malonyl-bougainvillein-r I
**Chemical Formula**	$C_{33}H_{38}N_2O_{21}$
**Molecular weight**	798.66
**Monoisotopic mass**	798.1967
***m/z* [M+H]**	799.2040
**Theorical fragments (*m/z*)**	799.2 798.2 797.19 796.18 783.21 782.2 781.19 780.19 779.18 771.21 770.2 769.19 767.21 765.2 764.19 763.18 761.17 759.21 757.19 755.21 754.21 753.2 752.19 751.18 749.2 747.19 745.17 741.2 739.22 737.2 736.2 735.19 729.2 727.18 726.18 725.2 724.2 723.19 713.2 712.2 711.19 710.18 709.17 697.21 695.19 694.19 693.18 691.16 685.21 683.19 681.21 679.2 677.18 675.17 669.21 667.2 665.18 663.2 661.19 659.17 655.2 651.2 649.19 643.2 641.18 637.15 636.14 635.14 630.17 625.19 623.17 621.16 619.14 618.13 617.12 609.16 607.14 606.17 605.16 604.15 603.14 601.13 599.11 593.16 591.15 590.15 589.14 588.16 587.15 586.14 585.14 583.12 579.15 575.15 574.15 573.15 572.14 571.13 567.15 565.13 561.14 560.16 559.15 558.15 557.15 556.14 555.13 554.13 551.15 549.14 547.12 545.15 544.14 535.16 534.15 533.15 531.12 529.11 528.15 527.14 521.15 520.17 519.13 518.13 517.16 516.15 515.13 513.11 505.16 504.15 503.14 502.16 501.12 500.14 499.13 498.14 497.12 487.14 486.14 485.13 479.13 469.13 468.13 461.12 459.15 447.15 444.11 443.11 442.1 441.09 435.15 433.13 432.13 430.15 429.14 428.09 427.09 426.1 424.09 423.08 417.14 415.12 414.12 412.1 411.09 410.08 409.08 399.13 394.09 393.08 392.07 389.1 388.09 387.08 386.07 383.1 375.12 373.1 372.1 371.09 370.08 369.07 365.09 361.1 360.1 359.12 357.11 356.1 355.09 354.09 353.09 351.06 349.1 347.09 345.11 343.13 342.08 341.11 339.1 338.06 337.08 336.08 335.08 331.09 329.11 327.1 326.09 325.09 324.08 323.08 319.09 317.08 316.07 315.1 314.09 313.08 306.07 303.1 301.08 300.07 299.07 287.1 285.09 284.08 283.07 281.08 280.07 273.1 271.11 269.09 267.06 265.08 264.07 263.07 262.06 255.09 253.08 251.07 249.05 247.07 245.06 237.09 235.07 233.06 231.04 223.07 211.07 207.08 205.06 196.06 194.04 193.04 182.08 180.04 179.03 178.05 177.04 165.05 164.05 163.04 147.04 146.04 135.04 134.04 130.04 123.04 119.05 111.04 109.03 108.02 107.05 106.04
**References**	Wybraniec, S.; Nowak-Wydra, B. Mammillarinin—A new malonylated betacyanin in fruits of Mammillaria. *J. Agric. Food Chem.* **2007**, 55, 8138–8143.<br>Slimen, I.B.; Najar, T.; Abderrabba, M. Chemical and Antioxidant Properties of Betalains. *J. Agric. Food Chem.* **2017**, 65, 675–689.<br>Kumorkiewicz-Jamro, A.; Świergosz, T.; Sutor, K.; Spórna-Kucab, A.; Wybraniec, S. Multi-colored shades of betalains: Recent advances in betacyanin chemistry. *Nat. Prod. Rep.* **2021**, 38, 2315–2346.

4’-*O*-Malonyl-isobougainvillein-r I

**Chemical structure**	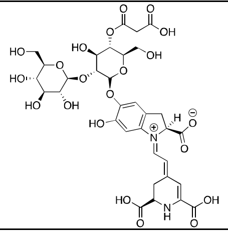
**Name:**	4’-*O*-Malonyl-isobougainvillein-r I
**Chemical Formula**	$C_{33}H_{38}N_{2}O_{21}$
**Molecular weight**	798.66
**Monoisotopic mass**	798.1967
***m/z* [M+H]**	799.2040
**Theorical fragments (*m/z*)**	799.2 798.2 797.19 796.18 783.21 782.2 781.19 780.19 779.18 771.21<br>770.2 769.19 767.21 765.2 764.19 763.18 761.17 759.21 757.19<br>755.21 754.21 753.2 752.19 751.18 749.2 747.19 745.17 741.2<br>739.22 737.2 736.2 735.19 729.2 727.18 726.18 725.2 724.2<br>723.19 713.2 712.2 711.19 710.18 709.17 697.21 695.19 694.19<br>693.18 691.16 685.21 683.19 681.21 679.2 677.18 675.17 669.21<br>667.2 665.18 663.2 661.19 659.17 655.2 651.2 649.19 643.2<br>641.18 637.15 636.14 635.14 630.17 625.19 623.17 621.16 619.14<br>618.13 617.12 609.16 607.14 606.17 605.16 604.15 603.14 601.13<br>599.11 593.16 591.15 590.15 589.14 588.16 587.15 586.14 585.14<br>583.12 579.15 575.15 574.15 573.15 572.14 571.13 567.15 565.13<br>561.14 560.16 559.15 558.15 557.15 556.14 555.13 554.13 551.15<br>549.14 547.12 545.15 544.14 535.16 534.15 533.15 531.12 529.11<br>528.15 527.14 521.15 520.17 519.13 518.13 517.16 516.15 515.13<br>513.11 505.16 504.15 503.14 502.16 501.12 500.14 499.13 498.14<br>497.12 487.14 486.14 485.13 479.13 469.13 468.13 461.12 459.15<br>447.15 444.11 443.11 442.1 441.09 435.15 433.13 432.13 430.15<br>429.14 428.09 427.09 426.1 424.09 423.08 417.14 415.12 414.12<br>412.1 411.09 410.08 409.08 399.13 394.09 393.08 392.07 389.1<br>388.09 387.08 386.07 383.1 375.12 373.1 372.1 371.09 370.08<br>369.07 365.09 361.1 360.1 359.12 357.11 356.1 355.09 354.09<br>353.09 351.06 349.1 347.09 345.11 343.13 342.08 341.11 339.1<br>338.06 337.08 336.08 335.08 331.09 329.11 327.1 326.09 325.09<br>324.08 323.08 319.09 317.08 316.07 315.1 314.09 313.08 306.07<br>303.1 301.08 300.07 299.07 287.1 285.09 284.08 283.07 281.08<br>280.07 273.1 271.11 269.09 267.06 265.08 264.07 263.07 262.06<br>255.09 253.08 251.07 249.05 247.07 245.06 237.09 235.07 233.06<br>231.04 223.07 211.07 207.08 205.06 196.06 194.04 193.04 182.08<br>180.04 179.03 178.05 177.04 165.05 164.05 163.04 147.04 146.04<br>135.04 134.04 130.04 123.04 119.05 111.04 109.03 108.02 107.05<br>106.04
**References**	Wybraniec, S.; Nowak-Wydra, B. Mammillarinin—A new malonylated betacyanin in fruits of Mammillaria. *J. Agric. Food Chem.* **2007**, 55, 8138–8143.<br>Slimen, I.B.; Najar, T.; Abderrabba, M. Chemical and Antioxidant Properties of Betalains. *J. Agric. Food Chem.* **2017**, 65, 675–689.<br>Kumorkiewicz-Jamro, A.; Świergosz, T.; Sutor, K.; Spórna-Kucab, A.; Wybraniec, S. Multi-colored shades of betalains: Recent advances in betacyanin chemistry. *Nat. Prod. Rep.* **2021**, 38, 2315–2346.

2-decarboxy-mammillarinin

**Chemical structure**	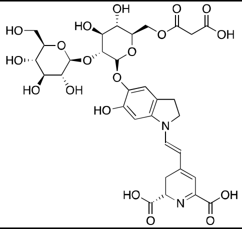
**Name:**	2-decarboxy-mammillarinin
**Chemical Formula**	$C_{32}H_{38}N_2O_{19}$
**Molecular weight**	754.65
**Monoisotopic mass**	754.2069
***m/z* [M+H]**	755.2142
**Theorical fragments (*m/z*)**	755.21 754.21 753.2 752.19 741.2 739.22 738.21 737.2 735.19 729.2 727.18 726.18 725.2 724.2 723.19 721.21 719.19 717.18 713.2 711.19 710.18 709.17 708.2 707.19 706.19 705.18 703.2 701.18 695.19 693.18 692.17 691.16 690.19 689.18 688.17 687.2 685.19 683.17 677.18 675.17 673.15 669.21 667.2 655.2 653.22 651.2 649.19 643.2 641.18 639.2 638.2 637.19 635.21 633.19 631.18 625.19 623.17 621.19 620.18 619.18 617.2 615.18 607.18 605.16 603.18 602.17 601.17 599.19 597.17 593.16 591.15 589.17 587.15 586.18 579.15 577.17 575.15 573.14 567.15 565.13 564.12 563.15 562.18 561.14 560.16 559.15 557.14 555.12 551.15 549.14 548.16 547.12 546.18 545.15 544.13 543.12 542.15 541.15 539.13 534.15 533.15 531.12 530.12 529.11 528.14 527.14 526.12 525.15 523.13 521.15 519.13 518.13 517.16 516.15 515.14 513.11 511.1 509.13 507.16 505.16 503.14 502.13 501.12 500.12 499.14 498.14 497.13 493.15 491.17 489.15 487.13 485.13 483.11 481.15 479.13 477.15 476.18 475.13 474.16 473.16 471.14 469.12 463.13 461.12 459.15 458.17 457.12 456.15 455.14 453.13 447.15 445.12 443.11 441.14 440.12 439.11 437.13 435.15 433.13 432.13 430.15 429.14 427.11 425.1 423.13 417.14 415.12 414.12 412.14 411.13 400.12 399.13 398.11 397.1 383.1 382.11 380.1 379.09 371.1 367.1 365.09 359.1 357.08 356.07 355.1 354.09 353.09 349.09 347.08 345.11 344.1 343.09 342.08 341.09 339.07 338.06 337.09 336.08 335.08 331.13 329.11 328.11 327.1 326.09 325.08 323.08 321.06 319.09 317.08 316.07 315.13 314.09 313.12 312.11 311.1 309.09 303.1 301.08 300.07 299.07 298.09 297.09 296.08 295.11 287.1 285.09 284.08 283.07 279.09 273.1 271.11 269.09 267.09 261.08 255.09 253.07 249.08 237.09 236.08 235.06 223.07 221.09 220.08 219.08 218.07 211.07 207.08 205.06 196.06 194.04 152.07 150.05 149.05 138.09 136.08 135.04 134.06 133.05 123.04 121.06 119.05 111.04 109.03 108.02 107.05 106.04
**References**	Wybraniec, S.; Nowak-Wydra, B. Mammillarinin—A new malonylated betacyanin in fruits of Mammillaria. *J. Agric. Food Chem.* **2007**, 55, 8138–8143.<br>Slimen, I.B.; Najar, T.; Abderrabba, M. Chemical and Antioxidant Properties of Betalains. *J. Agric. Food Chem.* **2017**, 65, 675–689.<br>Kumorkiewicz-Jamro, A.; Świergosz, T.; Sutor, K.; Spórna-Kucab, A.; Wybraniec, S. Multi-colored shades of betalains: Recent advances in betacyanin chemistry. *Nat. Prod. Rep.* **2021**, 38, 2315–2346.

17-decarboxy-mammillarinin

**Chemical structure**	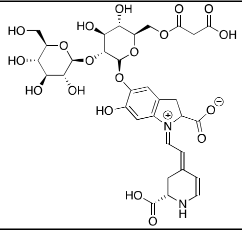
**Name:**	17-decarboxy-mammillarinin
**Chemical Formula**	$C_{32}H_{38}N_2O_{19}$
**Molecular weight**	754.65
**Monoisotopic mass**	754.2069
***m/z* [M+H]**	755.2142
**Theorical fragments (*m/z*)**	755.21 754.21 753.2 752.19 741.2 739.22 738.21 737.2 735.19 729.2 727.18 726.18 725.2 724.2 723.19 721.21 719.19 717.18 713.2 711.19 710.18 709.17 708.2 707.19 706.19 705.18 703.2 701.18 695.19 693.18 692.17 691.16 690.19 689.18 688.17 687.2 685.19 683.17 677.18 675.17 673.15 669.21 667.2 655.2 653.22 651.2 649.19 643.2 641.18 639.2 638.2 637.19 635.21 633.19 631.18 625.19 623.17 621.19 620.18 619.18 617.2 615.18 607.18 605.16 603.18 602.17 601.17 599.19 597.17 593.16 591.15 589.17 587.15 586.18 579.15 577.17 575.15 573.14 567.15 565.13 564.12 563.15 562.18 561.14 560.16 559.15 557.14 555.12 551.15 549.14 548.16 547.12 546.18 545.15 544.13 543.12 542.15 541.15 539.13 534.15 533.15 531.12 530.12 529.11 528.14 527.14 526.12 525.15 523.13 521.15 519.13 518.13 517.16 516.15 515.14 513.11 511.1 509.13 507.16 505.16 503.14 502.13 501.12 500.12 499.14 498.14 497.13 493.15 491.17 489.15 487.13 485.13 483.11 481.15 479.13 477.15 476.18 475.13 474.16 473.16 471.14 469.12 463.13 461.12 459.15 458.17 457.12 456.15 455.14 453.13 447.15 445.12 443.11 441.14 440.12 439.11 437.13 435.15 433.13 432.13 430.15 429.14 427.11 425.1 423.13 417.14 415.12 414.12 412.14 411.13 400.12 399.13 398.11 397.1 383.1 382.11 380.1 379.09 371.1 367.1 365.09 359.1 357.08 356.07 355.1 354.09 353.09 349.09 347.08 345.11 344.1 343.09 342.08 341.09 339.07 338.06 337.09 336.08 335.08 331.13 329.11 328.11 327.1 326.09 325.08 323.08 321.06 319.09 317.08 316.07 315.13 314.09 313.12 312.11 311.1 309.09 303.1 301.08 300.07 299.07 298.09 297.09 296.08 295.11 287.1 285.09 284.08 283.07 279.09 273.1 271.11 269.09 267.09 261.08 255.09 253.07 249.08 237.09 236.08 235.06 223.07 221.09 220.08 219.08 218.07 211.07 207.08 205.06 196.06 194.04 152.07 150.05 149.05 138.09 136.08 135.04 134.06 133.05 123.04 121.06 119.05 111.04 109.03 108.02 107.05 106.04
**References**	Wybraniec, S.; Nowak-Wydra, B. Mammillarinin—A new malonylated betacyanin in fruits of Mammillaria. *J. Agric. Food Chem.* **2007**, 55, 8138–8143.<br>Slimen, I.B.; Najar, T.; Abderrabba, M. Chemical and Antioxidant Properties of Betalains. *J. Agric. Food Chem.* **2017**, 65, 675–689.<br>Kumorkiewicz-Jamro, A.; Świergosz, T.; Sutor, K.; Spórna-Kucab, A.; Wybraniec, S. Multi-colored shades of betalains: Recent advances in betacyanin chemistry. *Nat. Prod. Rep.* **2021**, 38, 2315–2346.

17-decarboxy-melocactin

**Chemical structure**	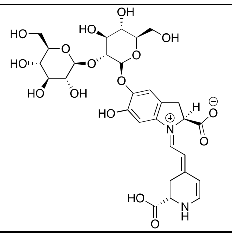
**Name:**	17-decarboxy-melocactin (17-decarboxy-bougainvillein-r)
**Chemical Formula**	$C_{29}H_{36}N_2O_{16}$
**Molecular weight**	668.61
**Monoisotopic mass**	668.2065
***m/z* [M+H]**	669.2138
**Theorical fragments (*m/z*)**	669.21 668.21 667.2 666.19 653.22 652.21 651.2 650.2 649.19 641.22 640.21 639.2 637.22 635.21 634.2 633.19 632.18 631.18 629.22 627.2 625.22 624.22 623.21 622.2 621.19 619.21 617.2 616.19 615.18 613.17 611.21 609.23 607.21 606.21 605.2 604.19 603.18 599.21 597.19 596.18 595.21 594.21 593.2 589.2 588.19 587.19 583.21 581.2 580.19 579.18 578.17 577.2 576.19 575.19 565.2 563.19 561.17 547.19 545.18 544.17 543.16 531.16 520.17 518.15 517.14 507.16 506.15 505.15 504.15 503.14 502.16 500.14 499.13 491.17 490.16 489.15 488.14 487.13 486.14 485.13 479.17 478.16 477.15 475.17 474.16 473.16 472.14 471.14 470.13 469.12 468.13 467.17 465.15 463.17 461.16 460.15 459.15 458.14 457.16 455.14 454.14 453.13 451.11 449.16 448.14 447.15 445.16 444.15 443.14 442.15 441.14 437.16 435.15 434.13 433.13 432.13 431.14 430.15 429.14 427.15 426.14 425.13 421.16 419.14 417.13 416.13 415.12 414.12 413.13 412.14 411.13 403.15 401.13 399.12 397.11 385.14 383.12 381.11 358.11 356.1 355.09 345.11 344.1 343.09 342.09 341.09 340.1 338.09 337.08 331.13 329.11 328.11 327.1 326.09 325.08 324.08 323.08 317.11 316.11 315.13 313.12 312.11 311.1 310.09 309.09 307.08 306.07 305.11 303.1 301.12 299.14 298.09 297.12 295.11 293.09 291.1 287.1 285.1 283.11 282.1 281.09 279.09 275.1 273.09 272.08 271.08 270.07 269.09 268.09 267.09 259.11 257.09 256.08 255.09 253.07 252.06 250.08 249.08 243.11 241.1 240.09 239.08 237.09 236.08 235.06 227.12 225.1 223.07 221.09 220.08 219.08 218.07 209.09 207.08 205.06 203.08 201.07 196.06 194.04 193.1 191.08 189.07 187.05 182.08 180.04 179.08 178.05 177.04 167.08 165.05 164.05 163.09 161.07 152.07 150.05 147.04 146.04 135.04 134.04 130.04 123.04 119.05 111.04 109.03 108.02 107.05 106.04
**References**	Wybraniec, S.; Nowak-Wydra, B. Mammillarinin—A new malonylated betacyanin in fruits of Mammillaria. *J. Agric. Food Chem.* **2007**, 55, 8138–8143.<br>Slimen, I.B.; Najar, T.; Abderrabba, M. Chemical and Antioxidant Properties of Betalains. *J. Agric. Food Chem.* **2017**, 65, 675–689.<br>Kumorkiewicz-Jamro, A.; Świergosz, T.; Sutor, K.; Spórna-Kucab, A.; Wybraniec, S. Multi-colored shades of betalains: Recent advances in betacyanin chemistry. *Nat. Prod. Rep.* **2021**, 38, 2315–2346.

Apiocactin-type
Apiocactin

**Chemical structure**	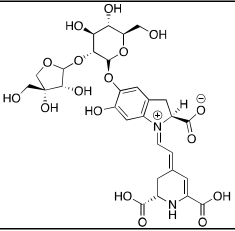
**Name:**	Apiocactin (2'-O-Apiosyl- betanin)
**Chemical Formula**	$C_{29}H_{34}N_2O_{17}$
**Molecular weight**	682.59
**Monoisotopic mass**	682.1857
***m/z* [M+H]**	683.1930
**Theorical fragments (*m/z*)**	683.19 682.19 681.18 680.17 667.2 666.19 665.18 664.17 663.17 655.2 654.19 653.18 651.2 650.2 649.19 648.18 647.17 646.16 645.16 643.2 641.18 640.17 639.2 638.2 637.19 636.18 635.17 633.19 632.18 631.18 630.17 629.16 627.15 625.19 623.21 621.19 620.18 619.18 618.17 617.16 615.18 613.19 611.17 610.16 609.19 608.18 607.18 605.2 604.19 603.18 602.17 601.17 597.19 595.18 594.17 593.16 592.15 591.18 590.17 589.17 579.18 577.17 575.15 561.17 559.16 557.14 551.15 550.14 549.14 535.16 534.15 533.14 532.13 531.12 523.16 522.15 521.14 519.16 517.15 516.14 515.13 514.16 513.11 511.16 509.14 507.16 505.15 504.14 503.13 501.15 499.13 498.13 497.12 496.14 495.1 493.15 491.13 490.16 489.15 488.14 487.13 486.13 485.12 483.14 481.15 479.13 478.12 477.15 476.14 475.13 474.14 473.13 472.14 471.14 470.13 469.12 465.15 463.13 462.16 461.12 460.11 459.14 458.13 457.13 456.13 455.12 454.13 452.12 447.14 445.12 444.15 443.11 442.13 439.12 438.12 432.15 431.12 429.14 428.13 427.11 426.14 425.1 420.15 418.13 417.14 412.14 411.13 405.14 403.12 402.12 400.14 399.13 389.1 388.09 387.08 386.07 385.11 384.11 382.13 381.12 375.12 373.1 372.1 371.09 370.08 369.07 367.1 361.1 360.1 359.12 358.11 357.11 356.1 355.09 354.08 353.08 351.06 349.1 347.09 345.11 344.1 343.13 342.09 341.11 340.1 339.1 338.09 337.08 335.09 331.09 329.11 327.1 326.09 325.09 324.08 323.08 322.09 320.08 319.09 317.08 316.07 315.1 314.09 313.08 312.11 311.1 307.08 306.07 303.1 301.08 300.07 299.07 298.06 297.1 295.1 294.1 287.1 286.09 285.1 284.08 283.07 282.06 281.08 280.07 279.09 273.1 271.08 270.07 269.09 268.09 267.06 265.08 264.07 263.07 262.06 255.09 253.08 252.06 251.07 250.08 249.05 247.07 245.06 237.09 235.07 233.06 231.04 223.07 222.06 220.06 211.07 207.08 205.06 196.06 195.05 194.04 193.04 192.03 182.08 180.04 179.03 178.05 177.04 166.09 165.05 164.05 163.04 150.05 147.04 146.04 135.04 134.04 130.04 124.04 123.04 119.05 111.04 110.06 109.03 108.02 107.05 106.04
**References**	Kobayashi, N.; Schmidt, J.; Nimtz, M.; Wray, V.; Schliemann, W. Betalains from *Christmas cactus*. *Phytochemistry* **2000**, 54, 419–426.<br>Wybraniec, S.; Nowak-Wydra, B.; Mitka, K.; Kowalski, P.; Mizrahi, Y. Minor betalains in fruits of *Hylocereus* species. *Phytochemistry* **2007**, 68, 251–259.<br>Slimen, I.B.; Najar, T.; Abderrabba, M. Chemical and Antioxidant Properties of Betalains. *J. Agric. Food Chem.* **2017**, 65, 675–689.<br>Kumorkiewicz-Jamro, A.; Świergosz, T.; Sutor, K.; Spórna-Kucab, A.; Wybraniec, S. Multi-colored shades of betalains: Recent advances in betacyanin chemistry. *Nat. Prod. Rep.* **2021**, 38, 2315–2346.

Isoapiocactin

**Chemical structure**	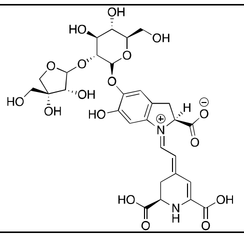
**Name:**	Isoapiocactin (2'-O-Apiosyl- betanin)
**Chemical Formula**	$C_{29}H_{34}N_2O_{17}$
**Molecular weight**	682.59
**Monoisotopic mass**	682.1857
***m/z* [M+H]**	683.1930
**Theorical fragments (*m/z*)**	683.19 682.19 681.18 680.17 667.2 666.19 665.18 664.17 663.17 655.2<br>654.19 653.18 651.2 650.2 649.19 648.18 647.17 646.16 645.16<br>643.2 641.18 640.17 639.2 638.2 637.19 636.18 635.17 633.19<br>632.18 631.18 630.17 629.16 627.15 625.19 623.21 621.19 620.18<br>619.18 618.17 617.16 615.18 613.19 611.17 610.16 609.19 608.18<br>607.18 605.2 604.19 603.18 602.17 601.17 597.19 595.18 594.17<br>593.16 592.15 591.18 590.17 589.17 579.18 577.17 575.15 561.17<br>559.16 557.14 551.15 550.14 549.14 535.16 534.15 533.14 532.13<br>531.12 523.16 522.15 521.14 519.16 517.15 516.14 515.13 514.16<br>513.11 511.16 509.14 507.16 505.15 504.14 503.13 501.15 499.13<br>498.13 497.12 496.14 495.1 493.15 491.13 490.16 489.15 488.14<br>487.13 486.13 485.12 483.14 481.15 479.13 478.12 477.15 476.14<br>475.13 474.14 473.13 472.14 471.14 470.13 469.12 465.15 463.13<br>462.16 461.12 460.11 459.14 458.13 457.13 456.13 455.12 454.13<br>452.12 447.14 445.12 444.15 443.11 442.13 439.12 438.12 432.15<br>431.12 429.14 428.13 427.11 426.14 425.1 420.15 418.13 417.14<br>412.14 411.13 405.14 403.12 402.12 400.14 399.13 389.1 388.09<br>387.08 386.07 385.11 384.11 382.13 381.12 375.12 373.1 372.1<br>371.09 370.08 369.07 367.1 361.1 360.1 359.12 358.11 357.11<br>356.1 355.09 354.08 353.08 351.06 349.1 347.09 345.11 344.1<br>343.13 342.09 341.11 340.1 339.1 338.09 337.08 335.09 331.09<br>329.11 327.1 326.09 325.09 324.08 323.08 322.09 320.08 319.09<br>317.08 316.07 315.1 314.09 313.08 312.11 311.1 307.08 306.07<br>303.1 301.08 300.07 299.07 298.06 297.1 295.1 294.1 287.1<br>286.09 285.1 284.08 283.07 282.06 281.08 280.07 279.09 273.1<br>271.08 270.07 269.09 268.09 267.06 265.08 264.07 263.07 262.06<br>255.09 253.08 252.06 251.07 250.08 249.05 247.07 245.06 237.09<br>235.07 233.06 231.04 223.07 222.06 220.06 211.07 207.08 205.06<br>196.06 195.05 194.04 193.04 192.03 182.08 180.04 179.03 178.05<br>177.04 166.09 165.05 164.05 163.04 150.05 147.04 146.04 135.04<br>134.04 130.04 124.04 123.04 119.05 111.04 110.06 109.03 108.02<br>107.05 106.04
**References**	Kobayashi, N.; Schmidt, J.; Nimtz, M.; Wray, V.; Schliemann, W. Betalains from *Christmas cactus. Phytochemistry* **2000**, 54, 419–426.<br>Wybraniec, S.; Nowak-Wydra, B.; Mitka, K.; Kowalski, P.; Mizrahi, Y. Minor betalains in fruits of *Hylocereus* species. *Phytochemistry* **2007**, 68, 251–259.<br>Slimen, I.B.; Najar, T.; Abderrabba, M. Chemical and Antioxidant Properties of Betalains. *J. Agric. Food Chem.* **2017**, 65, 675–689.<br>Kumorkiewicz-Jamro, A.; Świergosz, T.; Sutor, K.; Spórna-Kucab, A.; Wybraniec, S. Multi-colored shades of betalains: Recent advances in betacyanin chemistry. *Nat. Prod. Rep.* **2021**, 38, 2315–2346.

2’-*O*-apiosyl-phyllocactin

**Chemical structure**	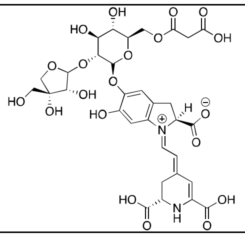
**Name:**	2'-*O*-apiosyl- phyllocactin
**Chemical Formula**	$C_{32}H_{36}N_2O_{20}$
**Molecular weight**	768.63
**Monoisotopic mass**	768.1861
***m/z* [M+H]**	769.1934
**Theorical fragments (*m/z*)**	769.19 768.19 767.18 766.17 753.2 752.19 751.18 750.18 749.17 741.2<br>740.19 739.18 737.2 735.19 734.18 733.17 732.16 731.16 729.2<br>727.18 725.2 724.2 723.19 722.18 721.17 719.19 717.18 716.17<br>715.16 713.15 711.19 709.21 707.19 706.19 705.18 704.17 703.16<br>701.18 699.19 697.17 696.16 695.19 694.19 693.18 689.18 688.17<br>687.17 683.19 682.19 681.18 680.17 679.16 678.15 677.18 676.17<br>675.17 667.2 665.18 664.17 663.17 661.15 655.2 653.18 651.2<br>649.19 647.17 646.16 645.16 643.14 639.2 637.15 636.14 635.14<br>633.19 631.18 629.16 627.15 625.19 621.16 620.15 619.14 618.13<br>617.12 613.19 611.17 609.16 608.15 607.14 605.16 603.15 602.14<br>601.13 600.16 599.11 597.16 595.18 593.16 591.15 590.14 589.13<br>587.15 585.14 584.13 583.12 581.1 579.15 577.17 576.16 575.15<br>574.14 573.13 572.13 571.12 569.14 567.15 565.13 564.12 563.15<br>562.14 561.14 560.14 559.13 558.15 557.14 556.13 555.12 551.15<br>550.14 549.14 547.12 546.11 545.14 544.14 543.13 542.13 541.12<br>535.16 533.14 532.13 531.12 530.15 529.11 528.13 527.14 526.13<br>525.12 524.12 523.16 521.14 517.15 515.13 514.13 513.11 511.1<br>505.15 504.13 503.14 499.13 498.14 497.12 495.1 493.15 491.14<br>490.16 489.12 488.12 487.14 486.14 485.13 481.12 479.13 475.14<br>474.14 473.13 472.14 471.11 470.13 469.13 468.13 467.12 463.13<br>461.12 457.13 456.13 455.12 453.1 445.12 444.11 443.11 442.1<br>441.09 439.12 438.12 429.14 428.09 427.09 426.1 425.1 424.09<br>423.08 417.14 412.1 411.09 410.08 409.08 405.14 403.12 402.12<br>400.14 399.13 394.09 393.08 392.07 389.1 388.09 387.08 386.07<br>385.11 384.11 383.1 382.13 381.12 375.12 373.1 372.1 371.09<br>370.08 369.07 367.1 365.09 361.1 360.1 359.12 357.11 356.1<br>355.09 354.09 353.09 351.06 349.1 347.09 345.11 343.13 342.08<br>341.11 339.1 338.06 337.08 336.08 335.08 331.09 329.11 327.1<br>326.09 325.09 324.08 323.08 321.06 319.09 317.08 316.07 315.1<br>314.09 313.08 306.07 303.1 301.08 300.07 299.07 287.1 285.09<br>284.08 283.07 281.08 280.07 273.1 271.11 269.09 267.06 265.08<br>264.07 263.07 262.06 255.09 253.08 251.07 249.05 247.07 245.06<br>237.09 235.07 233.06 231.04 223.07 211.07 207.08 205.06 196.06<br>194.04 193.04 182.08 180.04 179.03 178.05 177.04 165.05 164.05<br>163.04 147.04 146.04 135.04 134.04 130.04 123.04 119.05 111.04<br>109.03 108.02 107.05 106.04
**References**	Kobayashi, N.; Schmidt, J.; Nimtz, M.; Wray, V.; Schliemann, W. Betalains from *Christmas cactus. Phytochemistry* **2000**, 54, 419–426.<br>Kumorkiewicz-Jamro, A.; Świergosz, T.; Sutor, K.; Spórna-Kucab, A.; Wybraniec, S. Multi-colored shades of betalains: Recent advances in betacyanin chemistry. *Nat. Prod. Rep.* **2021**, 38, 2315–2346.

2’-*O*-apiosyl-isophyllocactin

**Chemical structure**	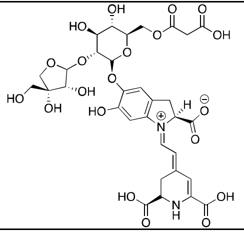
**Name:**	2'-O-apiosyl-isophyllocactin
**Chemical Formula**	$C_{32}H_{36}N_2O_{20}$
**Molecular weight**	768.63
**Monoisotopic mass**	768.1861
***m/z* [M+H]**	769.1934
**Theorical fragments (*m/z*)**	769.19 768.19 767.18 766.17 753.2 752.19 751.18 750.18 749.17 741.2<br>740.19 739.18 737.2 735.19 734.18 733.17 732.16 731.16 729.2<br>727.18 725.2 724.2 723.19 722.18 721.17 719.19 717.18 716.17<br>715.16 713.15 711.19 709.21 707.19 706.19 705.18 704.17 703.16<br>701.18 699.19 697.17 696.16 695.19 694.19 693.18 689.18 688.17<br>687.17 683.19 682.19 681.18 680.17 679.16 678.15 677.18 676.17<br>675.17 667.2 665.18 664.17 663.17 661.15 655.2 653.18 651.2<br>649.19 647.17 646.16 645.16 643.14 639.2 637.15 636.14 635.14<br>633.19 631.18 629.16 627.15 625.19 621.16 620.15 619.14 618.13<br>617.12 613.19 611.17 609.16 608.15 607.14 605.16 603.15 602.14<br>601.13 600.16 599.11 597.16 595.18 593.16 591.15 590.14 589.13<br>587.15 585.14 584.13 583.12 581.1 579.15 577.17 576.16 575.15<br>574.14 573.13 572.13 571.12 569.14 567.15 565.13 564.12 563.15<br>562.14 561.14 560.14 559.13 558.15 557.14 556.13 555.12 551.15<br>550.14 549.14 547.12 546.11 545.14 544.14 543.13 542.13 541.12<br>535.16 533.14 532.13 531.12 530.15 529.11 528.13 527.14 526.13<br>525.12 524.12 523.16 521.14 517.15 515.13 514.13 513.11 511.1<br>505.15 504.13 503.14 499.13 498.14 497.12 495.1 493.15 491.14<br>490.16 489.12 488.12 487.14 486.14 485.13 481.12 479.13 475.14<br>474.14 473.13 472.14 471.11 470.13 469.13 468.13 467.12 463.13<br>461.12 457.13 456.13 455.12 453.1 445.12 444.11 443.11 442.1<br>441.09 439.12 438.12 429.14 428.09 427.09 426.1 425.1 424.09<br>423.08 417.14 412.1 411.09 410.08 409.08 405.14 403.12 402.12<br>400.14 399.13 394.09 393.08 392.07 389.1 388.09 387.08 386.07<br>385.11 384.11 383.1 382.13 381.12 375.12 373.1 372.1 371.09<br>370.08 369.07 367.1 365.09 361.1 360.1 359.12 357.11 356.1<br>355.09 354.09 353.09 351.06 349.1 347.09 345.11 343.13 342.08<br>341.11 339.1 338.06 337.08 336.08 335.08 331.09 329.11 327.1<br>326.09 325.09 324.08 323.08 321.06 319.09 317.08 316.07 315.1<br>314.09 313.08 306.07 303.1 301.08 300.07 299.07 287.1 285.09<br>284.08 283.07 281.08 280.07 273.1 271.11 269.09 267.06 265.08<br>264.07 263.07 262.06 255.09 253.08 251.07 249.05 247.07 245.06<br>237.09 235.07 233.06 231.04 223.07 211.07 207.08 205.06 196.06<br>194.04 193.04 182.08 180.04 179.03 178.05 177.04 165.05 164.05<br>163.04 147.04 146.04 135.04 134.04 130.04 123.04 119.05 111.04<br>109.03 108.02 107.05 106.04
**References**	Kobayashi, N.; Schmidt, J.; Nimtz, M.; Wray, V.; Schliemann, W. Betalains from *Christmas cactus. Phytochemistry* **2000**, 54, 419–426.<br>Kumorkiewicz-Jamro, A.; Świergosz, T.; Sutor, K.; Spórna-Kucab, A.; Wybraniec, S. Multi-colored shades of betalains: Recent advances in betacyanin chemistry. *Nat. Prod. Rep.* **2021**, 38, 2315–2346.

5’’-*O*-*E*-Feruloyl-apiocactin

**Chemical structure**	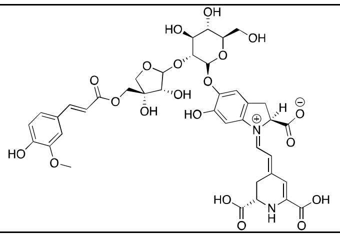
**Name:**	5´´-*O*-*E*-Feruloyl-apiocactin
**Chemical Formula**	$C_{39}H_{42}N_2O_{20}$
**Molecular weight**	858.76
**Monoisotopic mass**	858.2331
***m/z* [M+H]**	859.2404
**Theorical fragments (*m/z*)**	859.24 858.23 857.22 856.22 843.25 842.24 841.23 840.22 839.21 831.25 830.24 829.23 827.25 826.24 825.23 824.23 823.22 822.21 821.2 819.25 817.23 816.22 815.25 814.24 813.23 812.23 811.22 809.24 808.23 807.22 806.22 805.21 803.19 801.23 799.26 797.24 796.23 795.22 794.22 793.21 789.23 787.22 786.21 785.24 784.23 783.22 781.25 780.24 779.23 778.22 777.21 773.24 771.22 770.22 769.21 768.2 767.23 766.22 765.21 755.23 753.21 751.2 737.22 735.2 733.19 690.2 677.2 666.2 665.2 664.19 663.18 651.19 650.18 649.18 648.19 646.18 645.17 638.21 636.19 633.18 632.17 631.17 630.18 628.17 621.18 620.2 619.19 618.18 615.17 614.16 608.2 607.17 605.19 604.18 602.19 596.2 594.18 593.19 588.18 587.18 581.19 579.17 578.16 576.18 575.18 563.18 562.17 561.16 560.15 558.17 557.17 551.15 550.14 549.14 545.17 543.15 535.16 534.15 533.14 532.13 531.12 523.16 522.15 521.14 519.16 517.15 516.14 515.13 514.12 513.11 511.16 509.14 507.16 505.15 504.14 503.13 501.15 499.13 498.13 497.12 495.1 493.15 491.13 489.15 488.14 487.13 486.13 485.12 483.14 481.15 479.13 478.12 477.15 476.14 475.13 473.16 471.14 470.13 469.12 465.15 463.13 461.12 460.11 459.14 458.13 457.12 447.14 445.12 443.11 429.13 427.11 425.1 389.1 388.09 387.08 386.07 375.12 373.1 372.1 371.09 370.08 369.07 361.1 360.1 359.12 358.11 357.11 356.1 355.09 354.08 353.08 351.06 349.1 347.09 345.11 344.1 343.13 342.09 341.11 340.1 339.1 338.09 337.08 335.09 331.09 329.11 327.1 326.09 325.09 324.08 323.08 322.09 320.08 319.09 317.08 316.07 315.1 314.09 313.08 312.11 307.08 306.07 303.1 301.08 300.07 299.07 298.06 297.1 294.1 287.1 286.09 285.1 284.08 283.07 282.06 281.08 280.07 279.09 273.1 271.08 270.07 269.09 268.09 267.06 265.08 264.07 263.07 262.06 255.09 253.08 252.06 251.07 250.08 249.05 247.07 245.06 237.09 235.07 233.06 231.04 223.07 222.06 220.06 211.07 207.08 205.06 196.06 195.05 194.04 193.04 192.03 182.08 180.04 179.03 178.05 177.04 166.09 165.05 164.05 163.04 150.05 147.04 146.04 135.04 134.04 130.04 124.04 123.04 119.05 111.04 110.06 109.03 108.02 107.05 106.04
**References**	Schliemann, W.; Joy, R.W.; Komamine, A.; Metzger, J.W.; Nimtz, M.; Wray, V.; Strack, D. Betacyanins from plants and cell cultures of *Phytolacca americana*. *Phytochemistry* **1996**, 42, 1039–1046.<br>Slimen, I.B.; Najar, T.; Abderrabba, M. Chemical and Antioxidant Properties of Betalains. *J. Agric. Food Chem.* **2017**, 65, 675–689.<br>Kumorkiewicz-Jamro, A.; Świergosz, T.; Sutor, K.; Spórna-Kucab, A.; Wybraniec, S. Multi-colored shades of betalains: Recent advances in betacyanin chemistry. *Nat. Prod. Rep.* **2021**, 38, 2315–2346.

5’’-*O*-*E*-Feruloyl-isoapiocactin

**Chemical structure**	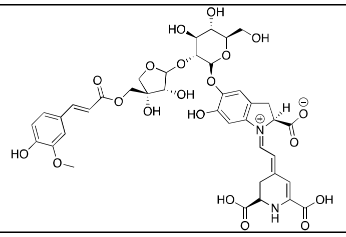
**Name:**	5´´-*O*-*E*-Feruloyl-isoapiocactin
**Chemical Formula**	$C_{39}H_{42}N_2O_{20}$
**Molecular weight**	858.76
**Monoisotopic mass**	858.2331
***m/z* [M+H]**	859.2404
**Theorical fragments (*m/z*)**	859.24 858.23 857.22 856.22 843.25 842.24 841.23 840.22 839.21 831.25<br>830.24 829.23 827.25 826.24 825.23 824.23 823.22 822.21 821.2<br>819.25 817.23 816.22 815.25 814.24 813.23 812.23 811.22 809.24<br>808.23 807.22 806.22 805.21 803.19 801.23 799.26 797.24 796.23<br>795.22 794.22 793.21 789.23 787.22 786.21 785.24 784.23 783.22<br>781.25 780.24 779.23 778.22 777.21 773.24 771.22 770.22 769.21<br>768.2 767.23 766.22 765.21 755.23 753.21 751.2 737.22 735.2<br>733.19 690.2 677.2 666.2 665.2 664.19 663.18 651.19 650.18<br>649.18 648.19 646.18 645.17 638.21 636.19 633.18 632.17 631.17<br>630.18 628.17 621.18 620.2 619.19 618.18 615.17 614.16 608.2<br>607.17 605.19 604.18 602.19 596.2 594.18 593.19 588.18 587.18<br>581.19 579.17 578.16 576.18 575.18 563.18 562.17 561.16 560.15<br>558.17 557.17 551.15 550.14 549.14 545.17 543.15 535.16 534.15<br>533.14 532.13 531.12 523.16 522.15 521.14 519.16 517.15 516.14<br>515.13 514.12 513.11 511.16 509.14 507.16 505.15 504.14 503.13<br>501.15 499.13 498.13 497.12 495.1 493.15 491.13 489.15 488.14<br>487.13 486.13 485.12 483.14 481.15 479.13 478.12 477.15 476.14<br>475.13 473.16 471.14 470.13 469.12 465.15 463.13 461.12 460.11<br>459.14 458.13 457.12 447.14 445.12 443.11 429.13 427.11 425.1<br>389.1 388.09 387.08 386.07 375.12 373.1 372.1 371.09 370.08<br>369.07 361.1 360.1 359.12 358.11 357.11 356.1 355.09 354.08<br>353.08 351.06 349.1 347.09 345.11 344.1 343.13 342.09 341.11<br>340.1 339.1 338.09 337.08 335.09 331.09 329.11 327.1 326.09<br>325.09 324.08 323.08 322.09 320.08 319.09 317.08 316.07 315.1<br>314.09 313.08 312.11 307.08 306.07 303.1 301.08 300.07 299.07<br>298.06 297.1 294.1 287.1 286.09 285.1 284.08 283.07 282.06<br>281.08 280.07 279.09 273.1 271.08 270.07 269.09 268.09 267.06<br>265.08 264.07 263.07 262.06 255.09 253.08 252.06 251.07 250.08<br>249.05 247.07 245.06 237.09 235.07 233.06 231.04 223.07 222.06<br>220.06 211.07 207.08 205.06 196.06 195.05 194.04 193.04 192.03<br>182.08 180.04 179.03 178.05 177.04 166.09 165.05 164.05 163.04<br>150.05 147.04 146.04 135.04 134.04 130.04 124.04 123.04 119.05<br>111.04 110.06 109.03 108.02 107.05 106.04
**References**	Schliemann, W.; Joy, R.W.; Komamine, A.; Metzger, J.W.; Nimtz, M.; Wray, V.; Strack, D. Betacyanins from plants and cell cultures of *Phytolacca americana*. *Phytochemistry* **1996**, 42, 1039–1046.<br>Slimen, I.B.; Najar, T.; Abderrabba, M. Chemical and Antioxidant Properties of Betalains. *J. Agric. Food Chem.* **2017**, 65, 675–689.<br>Kumorkiewicz-Jamro, A.; Świergosz, T.; Sutor, K.; Spórna-Kucab, A.; Wybraniec, S. Multi-colored shades of betalains: Recent advances in betacyanin chemistry. *Nat. Prod. Rep.* **2021**, 38, 2315–2346.

5''-*O*-*E*-Sinapoyl-apiocactin

**Chemical structure**	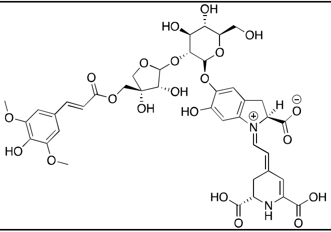
**Name:**	5''-*O*-*E*-Sinapoyl-apiocactin
**Chemical Formula**	$C_{40}H_{44}N_2O_{21}$
**Molecular weight**	888.79
**Monoisotopic mass**	888.2437
***m/z* [M+H]**	889.2509
**Theorical fragments (*m/z*)**	889.25 888.24 887.24 886.23 873.26 872.25 871.24 870.23 869.22 861.26 860.25 859.24 857.26 856.25 855.25 854.24 853.23 852.22 851.21 849.26 847.24 846.23 845.26 844.25 843.25 842.24 841.23 839.25 838.24 837.23 836.23 835.22 833.2 831.25 829.27 827.25 826.24 825.23 824.23 823.22 819.25 817.23 816.22 815.25 814.24 813.23 811.26 810.25 809.24 808.23 807.22 803.25 801.23 800.23 799.22 798.21 797.24 796.23 795.22 785.24 783.22 781.21 767.23 765.21 763.2 720.21 707.21 696.21 695.21 694.2 693.19 681.2 680.19 679.19 678.2 676.19 675.18 668.22 666.2 663.19 662.18 661.18 660.19 658.18 651.19 650.21 649.2 648.19 645.18 644.17 638.21 637.18 635.2 634.19 632.2 626.21 624.19 623.2 618.19 617.19 611.2 609.18 608.17 606.19 605.19 593.19 592.18 591.17 590.16 588.18 587.18 575.18 573.16 551.15 550.14 549.14 535.16 534.15 533.14 532.13 531.12 523.16 522.15 521.14 519.16 517.15 516.14 515.13 514.12 513.11 511.16 509.14 507.16 505.15 504.14 503.13 501.15 499.13 498.13 497.12 495.1 493.15 491.13 489.15 488.14 487.13 486.13 485.12 483.14 481.15 479.13 478.12 477.15 476.14 475.13 473.16 471.14 470.13 469.12 465.15 463.13 461.12 460.11 459.14 458.13 457.12 447.14 445.12 443.11 429.13 427.11 425.1 389.1 388.09 387.08 386.07 375.12 373.1 372.1 371.09 370.08 369.07 361.1 360.1 359.12 358.11 357.11 356.1 355.09 354.08 353.08 351.06 349.1 347.09 345.11 344.1 343.13 342.09 341.11 340.1 339.1 338.09 337.08 335.09 331.09 329.11 327.1 326.09 325.09 324.08 323.08 322.09 320.08 319.09 317.08 316.07 315.1 314.09 313.08 312.11 307.08 306.07 303.1 301.08 300.07 299.07 298.06 297.1 294.1 287.1 286.09 285.1 284.08 283.07 282.06 281.08 280.07 279.09 273.1 271.08 270.07 269.09 268.09 267.06 265.08 264.07 263.07 262.06 255.09 253.08 252.06 251.07 250.08 249.05 247.07 245.06 237.09 235.07 233.06 231.04 223.07 222.06 220.06 211.07 207.08 205.06 196.06 195.05 194.04 193.04 192.03 182.08 180.04 179.03 178.05 177.04 166.09 165.05 164.05 163.04 150.05 147.04 146.04 135.04 134.04 130.04 124.04 123.04 119.05 111.04 110.06 109.03 108.02 107.05 106.04
**References**	Wybraniec, S.; Nowak-Wydra, B.; Mitka, K.; Kowalski, P.; Mizrahi, Y. Minor betalains in fruits of *Hylocereus* species. *Phytochemistry* **2007**, 68, 251–259.<br>Sutor, K.; Wybraniec, S. Identification and determination of betacyanins in fruit extracts of *Melocactus* species. *J. Agric. Food Chem.* **2020**, 68, 11459–11467.<br>Slimen, I.B.; Najar, T.; Abderrabba, M. Chemical and Antioxidant Properties of Betalains. *J. Agric. Food Chem.* **2017**, 65, 675–689.<br>Kumorkiewicz-Jamro, A.; Świergosz, T.; Sutor, K.; Spórna-Kucab, A.; Wybraniec, S. Multi-colored shades of betalains: Recent advances in betacyanin chemistry. *Nat. Prod. Rep.* **2021**, 38, 2315–2346.

5’’-*O*-*E*-Sinapoyl-isoapiocactin

**Chemical structure**	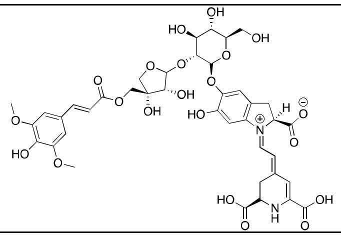
**Name:**	5’’-*O*-*E*-Sinapoyl-isoapiocactin
**Chemical Formula**	$C_{40}H_{44}N_2O_{21}$
**Molecular weight**	888.79
**Monoisotopic mass**	888.2437
***m/z* [M+H]**	889.2509
**Theorical fragments (*m/z*)**	889.25 888.24 887.24 886.23 873.26 872.25 871.24 870.23 869.22 861.26<br>860.25 859.24 857.26 856.25 855.25 854.24 853.23 852.22 851.21<br>849.26 847.24 846.23 845.26 844.25 843.25 842.24 841.23 839.25<br>838.24 837.23 836.23 835.22 833.2 831.25 829.27 827.25 826.24<br>825.23 824.23 823.22 819.25 817.23 816.22 815.25 814.24 813.23<br>811.26 810.25 809.24 808.23 807.22 803.25 801.23 800.23 799.22<br>798.21 797.24 796.23 795.22 785.24 783.22 781.21 767.23 765.21<br>763.2 720.21 707.21 696.21 695.21 694.2 693.19 681.2 680.19<br>679.19 678.2 676.19 675.18 668.22 666.2 663.19 662.18 661.18<br>660.19 658.18 651.19 650.21 649.2 648.19 645.18 644.17 638.21<br>637.18 635.2 634.19 632.2 626.21 624.19 623.2 618.19 617.19<br>611.2 609.18 608.17 606.19 605.19 593.19 592.18 591.17 590.16<br>588.18 587.18 575.18 573.16 551.15 550.14 549.14 535.16 534.15<br>533.14 532.13 531.12 523.16 522.15 521.14 519.16 517.15 516.14<br>515.13 514.12 513.11 511.16 509.14 507.16 505.15 504.14 503.13<br>501.15 499.13 498.13 497.12 495.1 493.15 491.13 489.15 488.14<br>487.13 486.13 485.12 483.14 481.15 479.13 478.12 477.15 476.14<br>475.13 473.16 471.14 470.13 469.12 465.15 463.13 461.12 460.11<br>459.14 458.13 457.12 447.14 445.12 443.11 429.13 427.11 425.1<br>389.1 388.09 387.08 386.07 375.12 373.1 372.1 371.09 370.08<br>369.07 361.1 360.1 359.12 358.11 357.11 356.1 355.09 354.08<br>353.08 351.06 349.1 347.09 345.11 344.1 343.13 342.09 341.11<br>340.1 339.1 338.09 337.08 335.09 331.09 329.11 327.1 326.09<br>325.09 324.08 323.08 322.09 320.08 319.09 317.08 316.07 315.1<br>314.09 313.08 312.11 307.08 306.07 303.1 301.08 300.07 299.07<br>298.06 297.1 294.1 287.1 286.09 285.1 284.08 283.07 282.06<br>281.08 280.07 279.09 273.1 271.08 270.07 269.09 268.09 267.06<br>265.08 264.07 263.07 262.06 255.09 253.08 252.06 251.07 250.08<br>249.05 247.07 245.06 237.09 235.07 233.06 231.04 223.07 222.06<br>220.06 211.07 207.08 205.06 196.06 195.05 194.04 193.04 192.03<br>182.08 180.04 179.03 178.05 177.04 166.09 165.05 164.05 163.04<br>150.05 147.04 146.04 135.04 134.04 130.04 124.04 123.04 119.05<br>111.04 110.06 109.03 108.02 107.05 106.04
**References**	Wybraniec, S.; Nowak-Wydra, B.; Mitka, K.; Kowalski, P.; Mizrahi, Y. Minor betalains in fruits of *Hylocereus* species. *Phytochemistry* **2007**, 68, 251–259.<br>Sutor, K.; Wybraniec, S. Identification and determination of betacyanins in fruit extracts of *Melocactus* species. *J. Agric. Food Chem.* **2020**, 68, 11459–11467.<br>Slimen, I.B.; Najar, T.; Abderrabba, M. Chemical and Antioxidant Properties of Betalains. *J. Agric. Food Chem.* **2017**, 65, 675–689.<br>Kumorkiewicz-Jamro, A.; Świergosz, T.; Sutor, K.; Spórna-Kucab, A.; Wybraniec, S. Multi-colored shades of betalains: Recent advances in betacyanin chemistry. *Nat. Prod. Rep.* **2021**, 38, 2315–2346.

2’-*O*-β-(5’’-*O*-*E*-Feruloyl)-apiosyl-phyllocactin

**Chemical structure**	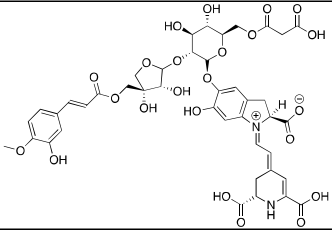
**Name:**	2’-*O*-b-(5’’-*O*-*E*-Feruloyl)-apiosyl-phyllocactin
**Chemical Formula**	$C_{42}H_{44}N_2O_{23}$
**Molecular weight**	944.81
**Monoisotopic mass**	944.2335
***m/z* [M+H]**	945.2408
**Theorical fragments (*m/z*)**	945.24 944.23 943.23 942.22 929.25 928.24 927.23 926.22 925.21 917.25 916.24 915.23 913.25 911.24 910.23 909.22 908.21 907.2 905.25 903.23 901.25 900.24 899.24 898.23 897.22 895.24 893.22 892.22 891.21 889.19 887.24 885.26 883.24 882.23 881.22 880.22 879.21 877.23 875.24 873.22 872.21 871.24 870.23 869.22 865.23 864.22 863.21 859.24 858.23 857.22 856.22 855.21 854.2 853.23 852.22 851.21 843.25 841.23 840.22 839.21 837.2 831.25 829.23 827.25 825.23 823.22 822.21 821.2 819.19 815.25 813.23 811.22 809.24 807.22 805.21 803.19 801.23 797.24 795.22 793.21 789.23 787.22 783.22 777.21 776.2 771.22 769.21 765.21 763.2 753.21 752.2 751.2 750.19 749.18 737.19 736.18 735.18 734.19 733.18 732.18 731.17 720.19 719.18 718.17 717.17 707.18 706.2 705.19 704.18 703.19 702.18 701.17 700.16 691.19 690.18 680.18 679.19 675.19 674.18 673.18 667.19 666.2 665.17 664.16 663.19 662.18 661.18 651.19 650.18 649.18 648.19 647.16 646.18 645.18 644.17 643.17 637.15 636.14 635.14 633.18 632.17 631.17 629.15 621.16 620.15 619.14 618.13 617.12 615.17 614.16 609.16 608.15 607.14 605.16 603.15 602.14 601.13 600.12 599.11 597.16 595.14 593.19 591.15 590.14 589.13 587.15 585.14 584.13 583.12 581.19 579.15 578.16 577.13 576.18 575.18 574.14 573.14 572.13 571.12 569.14 567.15 565.13 564.12 563.18 562.14 561.14 560.15 558.17 557.17 556.13 555.12 551.15 550.14 549.14 547.12 546.11 545.17 544.13 543.12 535.16 533.14 532.13 531.12 529.11 523.16 521.14 517.15 515.13 514.12 513.11 511.1 505.15 503.13 499.13 497.12 495.1 493.15 487.13 485.12 481.12 479.13 475.13 469.12 463.13 461.12 457.12 445.12 444.11 443.11 442.1 441.09 428.09 427.09 426.1 425.1 424.09 423.08 412.1 411.09 410.08 409.08 394.09 393.08 392.07 389.1 388.09 387.08 386.07 383.1 375.12 373.1 372.1 371.09 370.08 369.07 365.09 361.1 360.1 359.12 357.11 356.1 355.09 354.09 353.09 351.06 349.1 347.09 345.11 343.13 342.08 341.11 339.1 338.06 337.08 336.08 335.08 331.09 329.11 327.1 326.09 325.09 324.08 323.08 321.06 319.09 317.08 316.07 315.1 314.09 313.08 306.07 303.1 301.08 300.07 299.07 287.1 285.09 284.08 283.07 281.08 280.07 273.1 271.11 269.09 267.06 265.08 264.07 263.07 262.06 255.09 253.08 251.07 249.05 247.07 245.06 237.09 235.07 233.06 231.04 223.07 211.07 207.08 205.06 196.06 194.04 193.04 182.08 180.04 179.03 178.05 177.04 165.05 164.05 163.04 147.04 146.04 135.04 134.04 130.04 123.04 119.05 111.04 109.03 108.02 107.05 106.04
**References**	Kobayashi, N.; Schmidt, J.; Nimtz, M.; Wray, V.; Schliemann, W. Betalains from *Christmas cactus*. *Phytochemistry* **2000**, 54, 419–426.<br>Schliemann, W.; Joy, R.W.; Komamine, A.; Metzger, J.W.; Nimtz, M.; Wray, V.; Strack, D. Betacyanins from plants and cell cultures of *Phytolacca americana*. *Phytochemistry* **1996**, 42, 1039–1046.<br>Slimen, I.B.; Najar, T.; Abderrabba, M. Chemical and Antioxidant Properties of Betalains. *J. Agric. Food Chem.* **2017**, 65, 675–689.<br>Kumorkiewicz-Jamro, A.; Świergosz, T.; Sutor, K.; Spórna-Kucab, A.; Wybraniec, S. Multi-

	colored shades of betalains: Recent advances in betacyanin chemistry. *Nat. Prod. Rep.* **2021**, 38, 2315–2346.

2’-*O*-β-(5’’-*O*-*E*-Feruloyl)-apiosyl-isophyllocactin

**Chemical structure**	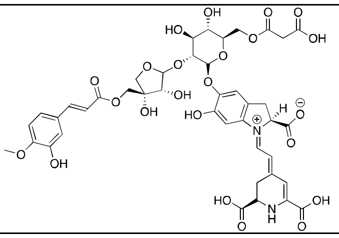
**Name:**	2’-*O*-β-(5’’-*O*-*E*-Feruloyl)-apiosyl-isophyllocactin
**Chemical Formula**	$C_{42}H_{44}N_2O_{23}$
**Molecular weight**	944.81
**Monoisotopic mass**	944.2335
***m/z* [M+H]**	945.2408
**Theorical fragments (*m/z*)**	945.24 944.23 943.23 942.22 929.25 928.24 927.23 926.22 925.21 917.25<br>916.24 915.23 913.25 911.24 910.23 909.22 908.21 907.2 905.25<br>903.23 901.25 900.24 899.24 898.23 897.22 895.24 893.22 892.22<br>891.21 889.19 887.24 885.26 883.24 882.23 881.22 880.22 879.21<br>877.23 875.24 873.22 872.21 871.24 870.23 869.22 865.23 864.22<br>863.21 859.24 858.23 857.22 856.22 855.21 854.2 853.23 852.22<br>851.21 843.25 841.23 840.22 839.21 837.2 831.25 829.23 827.25<br>825.23 823.22 822.21 821.2 819.19 815.25 813.23 811.22 809.24<br>807.22 805.21 803.19 801.23 797.24 795.22 793.21 789.23 787.22<br>783.22 777.21 776.2 771.22 769.21 765.21 763.2 753.21 752.2<br>751.2 750.19 749.18 737.19 736.18 735.18 734.19 733.18 732.18<br>731.17 720.19 719.18 718.17 717.17 707.18 706.2 705.19 704.18<br>703.19 702.18 701.17 700.16 691.19 690.18 680.18 679.19 675.19<br>674.18 673.18 667.19 666.2 665.17 664.16 663.19 662.18 661.18<br>651.19 650.18 649.18 648.19 647.16 646.18 645.18 644.17 643.17<br>637.15 636.14 635.14 633.18 632.17 631.17 629.15 621.16 620.15<br>619.14 618.13 617.12 615.17 614.16 609.16 608.15 607.14 605.16<br>603.15 602.14 601.13 600.12 599.11 597.16 595.14 593.19 591.15<br>590.14 589.13 587.15 585.14 584.13 583.12 581.19 579.15 578.16<br>577.13 576.18 575.18 574.14 573.14 572.13 571.12 569.14 567.15<br>565.13 564.12 563.18 562.14 561.14 560.15 558.17 557.17 556.13<br>555.12 551.15 550.14 549.14 547.12 546.11 545.17 544.13 543.12<br>535.16 533.14 532.13 531.12 529.11 523.16 521.14 517.15 515.13<br>514.12 513.11 511.1 505.15 503.13 499.13 497.12 495.1 493.15<br>487.13 485.12 481.12 479.13 475.13 469.12 463.13 461.12 457.12<br>445.12 444.11 443.11 442.1 441.09 428.09 427.09 426.1 425.1<br>424.09 423.08 412.1 411.09 410.08 409.08 394.09 393.08 392.07<br>389.1 388.09 387.08 386.07 383.1 375.12 373.1 372.1 371.09<br>370.08 369.07 365.09 361.1 360.1 359.12 357.11 356.1 355.09<br>354.09 353.09 351.06 349.1 347.09 345.11 343.13 342.08 341.11<br>339.1 338.06 337.08 336.08 335.08 331.09 329.11 327.1 326.09<br>325.09 324.08 323.08 321.06 319.09 317.08 316.07 315.1 314.09<br>313.08 306.07 303.1 301.08 300.07 299.07 287.1 285.09 284.08<br>283.07 281.08 280.07 273.1 271.11 269.09 267.06 265.08 264.07<br>263.07 262.06 255.09 253.08 251.07 249.05 247.07 245.06 237.09<br>235.07 233.06 231.04 223.07 211.07 207.08 205.06 196.06 194.04<br>193.04 182.08 180.04 179.03 178.05 177.04 165.05 164.05 163.04<br>147.04 146.04 135.04 134.04 130.04 123.04 119.05 111.04 109.03<br>108.02 107.05 106.04
**References**	Kobayashi, N.; Schmidt, J.; Nimtz, M.; Wray, V.; Schliemann, W. Betalains from *Christmas cactus*. *Phytochemistry* **2000**, 54, 419–426.<br>Schliemann, W.; Joy, R.W.; Komamine, A.; Metzger, J.W.; Nimtz, M.; Wray, V.; Strack, D. Betacyanins from plants and cell cultures of *Phytolacca americana*. *Phytochemistry* **1996**, 42, 1039–1046.<br>Slimen, I.B.; Najar, T.; Abderrabba, M. Chemical and Antioxidant Properties of Betalains. *J. Agric. Food Chem.* **2017**, 65, 675–689.<br>Kumorkiewicz-Jamro, A.; Świergosz, T.; Sutor, K.; Spórna-Kucab, A.; Wybraniec, S. Multi-

	colored shades of betalains: Recent advances in betacyanin chemistry. *Nat. Prod. Rep.* **2021**, 38, 2315–2346.

17-decarboxy-2’-*O*-apiosyl-phyllocactin

**Chemical structure**	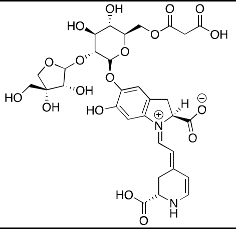
**Name:**	17-decarboxy-2’-O-apiosyl-phyllocactin
**Chemical Formula**	$C_{31}H_{36}N_2O_{18}$
**Molecular weight**	724.62
**Monoisotopic mass**	724.1963
***m/z* [M+H]**	725.2036
**Theorical fragments (*m/z*)**	725.2 724.2 723.19 722.18 709.21 708.2 707.19 706.19 705.18 697.21<br>696.2 695.19 693.21 691.2 690.19 689.18 688.17 687.17<br>685.21 683.19 681.21 680.21 679.2 678.19 677.18 675.2<br>673.19 672.18 671.17 669.16 667.2 665.22 663.2 662.2<br>661.19 660.18 659.17 657.19 655.2 653.18 652.17 651.2<br>650.2 649.19 645.19 644.18 643.18 639.2 638.2 637.19<br>636.18 635.17 634.16 633.19 632.18 631.18 623.21 621.19<br>620.18 619.18 617.16 611.21 609.19 607.21 605.2 603.18<br>602.17 601.17 600.16 599.15 595.21 593.16 592.15 591.15<br>589.2 587.19 585.17 583.16 581.2 577.17 576.16 575.15<br>574.14 573.13 569.2 567.18 565.17 564.16 563.15 561.17<br>560.14 559.16 558.15 557.14 556.13 555.12 553.17 551.19<br>549.17 547.16 546.15 545.14 544.14 543.13 542.13 541.15<br>540.14 539.13 537.11 535.16 533.18 531.16 530.15 529.15<br>528.13 527.14 526.13 525.12 524.12 523.16 521.14 520.13<br>519.16 518.15 517.15 515.14 514.13 513.15 512.14 511.13<br>507.16 506.15 505.15 504.13 503.14 502.12 501.15 500.14<br>499.14 498.14 497.13 491.14 490.16 489.12 488.12 487.14<br>486.14 485.13 479.17 477.15 475.14 474.14 473.13 472.14<br>471.14 470.13 469.12 468.13 467.12 461.16 459.14 457.13<br>456.13 455.14 453.13 451.11 449.16 444.11 443.14 442.1<br>441.09 439.12 438.12 437.13 435.14 431.14 429.14 428.09<br>427.09 426.1 425.13 424.09 423.08 419.14 417.14 413.13<br>412.1 411.09 410.08 409.08 405.14 403.12 402.12 401.13<br>400.14 399.13 394.09 393.08 392.07 387.13 385.11 384.11<br>383.1 382.13 381.12 371.1 369.12 367.1 365.09 359.1<br>357.08 356.07 355.1 354.09 353.09 345.11 344.1 343.09<br>342.09 341.09 339.07 338.06 337.09 336.08 335.08 331.13<br>329.11 328.11 327.1 326.09 325.08 324.08 323.08 321.06<br>317.11 316.11 315.13 313.12 312.11 311.1 310.09 309.09<br>307.07 306.07 305.11 303.1 301.12 299.14 298.09 297.12<br>295.11 293.09 291.1 287.1 285.12 283.11 282.1 281.09<br>275.1 273.09 272.08 271.11 270.1 269.09 267.09 259.11<br>257.09 256.08 255.08 253.07 249.08 243.11 241.1 240.09<br>239.08 237.09 236.08 235.06 227.12 225.1 223.07 221.09<br>220.08 219.08 218.07 209.09 207.08 205.06 203.08 201.07<br>196.06 194.04 193.1 191.08 189.07 187.05 182.08 180.04<br>179.08 178.05 177.04 167.08 165.05 164.05 163.09 161.07<br>152.07 150.05 147.04 146.04 135.04 134.04 130.04 123.04<br>119.05 111.04 109.03 108.02 107.05 106.04
**References**	Kobayashi, N.; Schmidt, J.; Nimtz, M.; Wray, V.; Schliemann, W. Betalains from *Christmas cactus*. *Phytochemistry* **2000**, 54, 419–426.<br>Kumorkiewicz-Jamro, A.; Świergosz, T.; Sutor, K.; Spórna-Kucab, A.; Wybraniec, S. Multi-colored shades of betalains: Recent advances in betacyanin chemistry. *Nat. Prod. Rep.* **2021**, 38, 2315–2346.

17-decarboxy-2'-*O*-apiosyl-isophyllocactin

**Chemical structure**	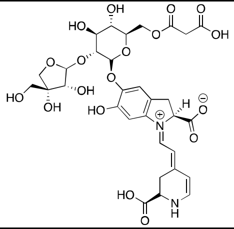
**Name:**	17-decarboxy-2'-O-apiosyl-isophyllocactin
**Chemical Formula**	$C_{31}H_{36}N_2O_{18}$
**Molecular weight**	724.62
**Monoisotopic mass**	724.1963
***m/z* [M+H]**	725.2036
**Theorical fragments (*m/z*)**	725.2 724.2 723.19 722.18 709.21 708.2 707.19 706.19 705.18 697.21<br>696.2 695.19 693.21 691.2 690.19 689.18 688.17 687.17<br>685.21 683.19 681.21 680.21 679.2 678.19 677.18 675.2<br>673.19 672.18 671.17 669.16 667.2 665.22 663.2 662.2<br>661.19 660.18 659.17 657.19 655.2 653.18 652.17 651.2<br>650.2 649.19 645.19 644.18 643.18 639.2 638.2 637.19<br>636.18 635.17 634.16 633.19 632.18 631.18 623.21 621.19<br>620.18 619.18 617.16 611.21 609.19 607.21 605.2 603.18<br>602.17 601.17 600.16 599.15 595.21 593.16 592.15 591.15<br>589.2 587.19 585.17 583.16 581.2 577.17 576.16 575.15<br>574.14 573.13 569.2 567.18 565.17 564.16 563.15 561.17<br>560.14 559.16 558.15 557.14 556.13 555.12 553.17 551.19<br>549.17 547.16 546.15 545.14 544.14 543.13 542.13 541.15<br>540.14 539.13 537.11 535.16 533.18 531.16 530.15 529.15<br>528.13 527.14 526.13 525.12 524.12 523.16 521.14 520.13<br>519.16 518.15 517.15 515.14 514.13 513.15 512.14 511.13<br>507.16 506.15 505.15 504.13 503.14 502.12 501.15 500.14<br>499.14 498.14 497.13 491.14 490.16 489.12 488.12 487.14<br>486.14 485.13 479.17 477.15 475.14 474.14 473.13 472.14<br>471.14 470.13 469.12 468.13 467.12 461.16 459.14 457.13<br>456.13 455.14 453.13 451.11 449.16 444.11 443.14 442.1<br>441.09 439.12 438.12 437.13 435.14 431.14 429.14 428.09<br>427.09 426.1 425.13 424.09 423.08 419.14 417.14 413.13<br>412.1 411.09 410.08 409.08 405.14 403.12 402.12 401.13<br>400.14 399.13 394.09 393.08 392.07 387.13 385.11 384.11<br>383.1 382.13 381.12 371.1 369.12 367.1 365.09 359.1<br>357.08 356.07 355.1 354.09 353.09 345.11 344.1 343.09<br>342.09 341.09 339.07 338.06 337.09 336.08 335.08 331.13<br>329.11 328.11 327.1 326.09 325.08 324.08 323.08 321.06<br>317.11 316.11 315.13 313.12 312.11 311.1 310.09 309.09<br>307.07 306.07 305.11 303.1 301.12 299.14 298.09 297.12<br>295.11 293.09 291.1 287.1 285.12 283.11 282.1 281.09<br>275.1 273.09 272.08 271.11 270.1 269.09 267.09 259.11<br>257.09 256.08 255.08 253.07 249.08 243.11 241.1 240.09<br>239.08 237.09 236.08 235.06 227.12 225.1 223.07 221.09<br>220.08 219.08 218.07 209.09 207.08 205.06 203.08 201.07<br>196.06 194.04 193.1 191.08 189.07 187.05 182.08 180.04<br>179.08 178.05 177.04 167.08 165.05 164.05 163.09 161.07<br>152.07 150.05 147.04 146.04 135.04 134.04 130.04 123.04<br>119.05 111.04 109.03 108.02 107.05 106.04
**References**	Kobayashi, N.; Schmidt, J.; Nimtz, M.; Wray, V.; Schliemann, W. Betalains from *Christmas cactus*. *Phytochemistry* **2000**, 54, 419–426.<br>Kumorkiewicz-Jamro, A.; Świergosz, T.; Sutor, K.; Spórna-Kucab, A.; Wybraniec, S. Multi-colored shades of betalains: Recent advances in betacyanin chemistry. *Nat. Prod. Rep.* **2021**, 38, 2315–2346.

Gomphrenin-type

Gomphrenin I

**Chemical structure**	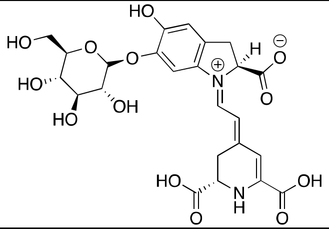
**Name:**	Gomphrenin-I
**Chemical Formula**	$C_{24}H_{26}N_2O_{13}$
**Molecular weight**	550.47
**Monoisotopic mass**	550.1435
***m/z* [M+H]**	551.1508
**Theorical fragments (*m/z*)**	551.15 550.14 549.14 548.13 535.16 534.15 533.14 532.13 531.12 523.16<br>522.15 521.14 519.16 518.15 517.15 516.14 515.13 514.12 513.11<br>511.16 509.14 508.13 507.16 506.15 505.15 504.14 503.13 501.15<br>499.13 497.12 495.1 493.15 491.17 490.16 489.15 488.14 487.13<br>486.13 485.12 483.14 481.15 479.13 478.12 477.15 475.13 473.16<br>471.14 469.12 465.15 463.13 462.13 461.12 460.11 459.14 457.12<br>447.14 445.12 443.11 427.11 425.1 389.1 388.09 387.08 386.07<br>382.11 375.12 374.11 373.1 372.1 371.09 370.08 369.07 368.1<br>366.12 364.1 361.1 360.1 359.12 358.11 357.11 356.1 355.09<br>354.08 353.08 352.1 351.06 349.1 347.09 345.11 344.1 343.13<br>342.09 341.11 340.1 339.1 338.09 337.08 336.07 335.09 334.08<br>331.09 330.08 329.11 328.1 327.1 326.1 325.09 324.08 323.1<br>322.09 320.08 319.09 317.08 316.07 315.1 314.09 313.09 312.11<br>311.1 310.09 309.1 308.09 307.08 306.07 303.1 301.08 300.07<br>299.07 298.06 297.1 296.09 294.1 288.11 287.1 286.09 285.1<br>283.07 281.08 280.07 279.09 273.1 271.08 270.07 269.1 268.08<br>267.09 265.08 264.07 263.07 262.06 257.1 255.09 254.08 253.07<br>252.06 251.07 249.08 247.07 245.06 239.09 237.08 235.07 233.06<br>223.07 220.06 211.07 209.06 208.05 207.05 205.06 196.06 195.05<br>194.04 193.04 191.06 182.08 181.05 180.04 179.03 178.05 177.04<br>176.03 175.06 166.09 165.05 164.05 163.04 162.03 150.05 149.06<br>148.05 147.04 139.04 135.04 124.04 123.04 122.04 121.03 119.05<br>118.04 111.04 110.06 109.03 108.02 107.05 106.04
**References**	Minale, L.; Piattelli, M.; de Stefano, S. Pigments of Centrospermae VII. Betacyanins from *Gomphrena globosa* L. *Phytochemistry* **1967**, 6, 703–708.<br>Heuer, S.; Wray, V.; Metzger, J.W.; Strack, D. Betacyanins from flowers of *Gomphrena globosa*. *Phytochemistry* **1992**, 31, 1801–1807.<br>Wu, J.Y.; Chen, W.C.; Wu, Y.Y.; Chen, J.T.; Chen, L.G.; Chiou, R.Y.Y. NMR-based elucidation of the positional C6-*O*-glucopyranosyl substitution of gomphrenin I isolated from *Basella alba* fruits. *J. Sci. Food Agric.* **2013**, 2, 8–11.<br>Spórna-Kucab, A.; Wróbel, N.; Kumorkiewicz-Jamro, A.; Wybraniec, S. Separation of betacyanins from Iresine herbstii Hook. ex Lindl. leaves by high-speed countercurrent chromatography in a polar solvent system. *J. Chromatogr. A* **2020**, 1626, 461370.<br>Slimen, I.B.; Najar, T.; Abderrabba, M. Chemical and Antioxidant Properties of Betalains. *J. Agric. Food Chem.* **2017**, 65, 675–689.<br>Kumorkiewicz-Jamro, A.; Świergosz, T.; Sutor, K.; Spórna-Kucab, A.; Wybraniec, S. Multi-colored shades of betalains: Recent advances in betacyanin chemistry. *Nat. Prod. Rep.* **2021**, 38, 2315–2346.

Isogomphrenin I

**Chemical structure**	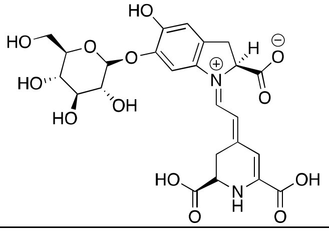
**Name:**	Isogomphrenin-I
**Chemical Formula**	$C_{24}H_{26}N_2O_{13}$
**Molecular weight**	550.47
**Monoisotopic mass**	550.1435
***m/z* [M+H]**	551.1508
**Theorical fragments (*m/z*)**	551.15 550.14 549.14 548.13 535.16 534.15 533.14 532.13 531.12 523.16<br>522.15 521.14 519.16 518.15 517.15 516.14 515.13 514.12 513.11<br>511.16 509.14 508.13 507.16 506.15 505.15 504.14 503.13 501.15<br>499.13 497.12 495.1 493.15 491.17 490.16 489.15 488.14 487.13<br>486.13 485.12 483.14 481.15 479.13 478.12 477.15 475.13 473.16<br>471.14 469.12 465.15 463.13 462.13 461.12 460.11 459.14 457.12<br>447.14 445.12 443.11 427.11 425.1 389.1 388.09 387.08 386.07<br>382.11 375.12 374.11 373.1 372.1 371.09 370.08 369.07 368.1<br>366.12 364.1 361.1 360.1 359.12 358.11 357.11 356.1 355.09<br>354.08 353.08 352.1 351.06 349.1 347.09 345.11 344.1 343.13<br>342.09 341.11 340.1 339.1 338.09 337.08 336.07 335.09 334.08<br>331.09 330.08 329.11 328.1 327.1 326.1 325.09 324.08 323.1<br>322.09 320.08 319.09 317.08 316.07 315.1 314.09 313.09 312.11<br>311.1 310.09 309.1 308.09 307.08 306.07 303.1 301.08 300.07<br>299.07 298.06 297.1 296.09 294.1 288.11 287.1 286.09 285.1<br>283.07 281.08 280.07 279.09 273.1 271.08 270.07 269.1 268.08<br>267.09 265.08 264.07 263.07 262.06 257.1 255.09 254.08 253.07<br>252.06 251.07 249.08 247.07 245.06 239.09 237.08 235.07 233.06<br>223.07 220.06 211.07 209.06 208.05 207.05 205.06 196.06 195.05<br>194.04 193.04 191.06 182.08 181.05 180.04 179.03 178.05 177.04<br>176.03 175.06 166.09 165.05 164.05 163.04 162.03 150.05 149.06<br>148.05 147.04 139.04 135.04 124.04 123.04 122.04 121.03 119.05<br>118.04 111.04 110.06 109.03 108.02 107.05 106.04
**References**	Minale, L.; Piattelli, M.; de Stefano, S. Pigments of Centrospermae VII. Betacyanins from *Gomphrena globosa* L. *Phytochemistry* **1967**, 6, 703–708.<br>Heuer, S.; Wray, V.; Metzger, J.W.; Strack, D. Betacyanins from flowers of *Gomphrena globosa*. *Phytochemistry* **1992**, 31, 1801–1807.<br>Wu, J.Y.; Chen, W.C.; Wu, Y.Y.; Chen, J.T.; Chen, L.G.; Chiou, R.Y.Y. NMR-based elucidation of the positional C6-*O*-glucopyranosyl substitution of gomphrenin I isolated from *Basella alba* fruits. *J. Sci. Food Agric.* **2013**, 2, 8–11.<br>Spórna-Kucab, A.; Wróbel, N.; Kumorkiewicz-Jamro, A.; Wybraniec, S. Separation of betacyanins from Iresine herbstii Hook. ex Lindl. leaves by high-speed countercurrent chromatography in a polar solvent system. *J. Chromatogr. A* **2020**, 1626, 461370.<br>Slimen, I.B.; Najar, T.; Abderrabba, M. Chemical and Antioxidant Properties of Betalains. *J. Agric. Food Chem.* **2017**, 65, 675–689.<br>Kumorkiewicz-Jamro, A.; Świergosz, T.; Sutor, K.; Spórna-Kucab, A.; Wybraniec, S. Multi-colored shades of betalains: Recent advances in betacyanin chemistry. *Nat. Prod. Rep.* **2021**, 38, 2315–2346.

Gomphrenin II

Chemical structure	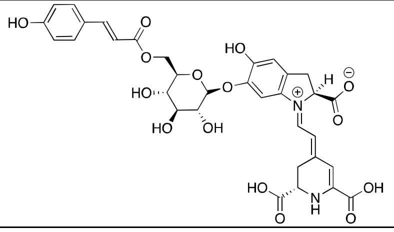									
**Name:**	Gomphrenin II									
**Chemical Formula**	$C_{33}H_{32}N_2O_{15}$									
**Molecular weight**	696.62									
**Monoisotopic mass**	696.1803									
***m/z* [M+H]**	697.1875									
**Theorical fragments (*m/z*)**	697.19	696.18	695.17	694.16	681.19	680.18	679.18	678.17	677.16	669.19
		668.18	667.18	665.2	664.19	663.18	662.17	661.17	660.16	659.15
		657.19	655.18	654.17	653.2	652.19	651.18	650.17	649.17	647.19
		645.17	643.16	641.14	639.18	637.2	636.19	635.19	634.18	633.17
		632.16	631.16	629.18	627.18	625.17	624.16	623.19	621.17	619.19
		617.18	615.16	611.19	609.17	608.16	607.16	606.15	605.18	603.16
		593.18	591.16	589.15	573.15	571.13	533.14	532.13	531.12	528.15
		517.15	516.14	515.13	514.12	513.11	512.16	510.14	505.15	504.15
		503.13	502.13	501.13	499.13	498.14	497.12	495.1	493.15	491.13
		489.15	488.13	487.13	486.14	485.13	484.12	483.12	481.12	479.11
		476.16	475.13	474.14	473.16	472.14	471.13	470.12	469.12	468.13
		466.11	463.13	461.12	460.11	459.13	458.14	457.14	456.13	455.13
		454.13	453.12	452.11	447.14	446.14	445.12	444.13	443.13	442.13
		440.13	434.14	432.13	431.13	427.14	425.12	419.13	417.12	416.11
		415.14	414.12	413.12	403.14	401.12	400.12	399.11	398.1	397.13
		395.11	389.1	388.09	387.08	386.07	385.13	383.11	381.1	375.12
		374.11	373.1	372.1	371.09	370.08	369.07	364.1	361.1	360.1
		359.12	358.12	357.11	356.1	355.09	354.08	353.08	351.06	349.1
		347.09	345.11	344.1	343.13	342.08	341.11	340.1	339.1	338.09
		337.08	336.07	335.09	334.08	331.09	330.08	329.11	327.1	326.09
		325.12	324.08	323.1	322.09	320.08	319.09	317.08	316.07	315.1
		314.09	313.08	312.07	310.09	309.1	308.09	307.08	306.07	303.1
		301.08	300.07	299.07	298.06	296.08	294.1	287.1	285.09	283.07
		281.08	280.07	279.09	268.08	267.09	265.08	264.07	263.07	262.06
		255.09	253.07	252.06	251.07	249.08	247.07	245.06	239.09	237.08
		235.07	233.06	223.07	220.06	211.07	209.06	208.05	207.05	205.06
		196.06	195.05	194.04	193.04	191.06	182.08	181.05	180.04	179.03
		178.05	177.04	176.03	175.06	166.09	165.05	164.05	163.04	162.03
		150.05	149.06	148.05	147.04	139.04	135.04	124.04	123.04	122.04
		121.03	119.05	118.04	111.04	110.06	109.03	108.02	107.05	106.04
**References**	Minale, L.; Piattelli, M.; de Stefano, S. Pigments of Centrospermae VII. Betacyanins from *Gomphrena globosa* L. *Phytochemistry* **1967**, 6, 703–708.<br>Heuer, S.; Wray, V.; Metzger, J.W.; Strack, D. Betacyanins from flowers of *Gomphrena globosa*. *Phytochemistry* **1992**, 31, 1801–1807.<br>Wu, J.Y.; Chen, W.C.; Wu, Y.Y.; Chen, J.T.; Chen, L.G.; Chiou, R.Y.Y. NMR-based elucidation of the positional C6-*O*-glucopyranosyl substitution of gomphrenin I isolated from *Basella alba* fruits. *J. Sci. Food Agric.* **2013**, 2, 8–11.<br>Spórna-Kucab, A.; Wróbel, N.; Kumorkiewicz-Jamro, A.; Wybraniec, S. Separation of betacyanins from Iresine herbstii Hook. ex Lindl. leaves by high-speed countercurrent chromatography in a polar solvent system. *J. Chromatogr. A* **2020**, 1626, 461370.<br>Slimen, I.B.; Najar, T.; Abderrabba, M. Chemical and Antioxidant Properties of Betalains. *J. Agric. Food Chem.* **2017**, 65, 675–689.<br>Kumorkiewicz-Jamro, A.; Świergosz, T.; Sutor, K.; Spórna-Kucab, A.; Wybraniec, S. Multi-colored shades of betalains: Recent advances in betacyanin chemistry. *Nat. Prod. Rep.* **2021**, 38, 2315–2346.									

Isogomphrenin-II

**Chemical structure**	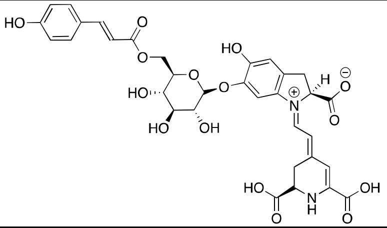
**Name:**	Isogomphrenin-II
**Chemical Formula**	$C_{33}H_{32}N_2O_{15}$
**Molecular weight**	696.62
**Monoisotopic mass**	696.1803
***m/z* [M+H]**	697.1875
**Theorical fragments (*m/z*)**	697.19 696.18 695.17 694.16 681.19 680.18 679.18 678.17 677.16 669.19<br>668.18 667.18 665.2 664.19 663.18 662.17 661.17 660.16 659.15<br>657.19 655.18 654.17 653.2 652.19 651.18 650.17 649.17 647.19<br>645.17 643.16 641.14 639.18 637.2 636.19 635.19 634.18 633.17<br>632.16 631.16 629.18 627.18 625.17 624.16 623.19 621.17 619.19<br>617.18 615.16 611.19 609.17 608.16 607.16 606.15 605.18 603.16<br>593.18 591.16 589.15 573.15 571.13 533.14 532.13 531.12 528.15<br>517.15 516.14 515.13 514.12 513.11 512.16 510.14 505.15 504.15<br>503.13 502.13 501.13 499.13 498.14 497.12 495.1 493.15 491.13<br>489.15 488.13 487.13 486.14 485.13 484.12 483.12 481.12 479.11<br>476.16 475.13 474.14 473.16 472.14 471.13 470.12 469.12 468.13<br>466.11 463.13 461.12 460.11 459.13 458.14 457.14 456.13 455.13<br>454.13 453.12 452.11 447.14 446.14 445.12 444.13 443.13 442.13<br>440.13 434.14 432.13 431.13 427.14 425.12 419.13 417.12 416.11<br>415.14 414.12 413.12 403.14 401.12 400.12 399.11 398.1 397.13<br>395.11 389.1 388.09 387.08 386.07 385.13 383.11 381.1 375.12<br>374.11 373.1 372.1 371.09 370.08 369.07 364.1 361.1 360.1<br>359.12 358.12 357.11 356.1 355.09 354.08 353.08 351.06 349.1<br>347.09 345.11 344.1 343.13 342.08 341.11 340.1 339.1 338.09<br>337.08 336.07 335.09 334.08 331.09 330.08 329.11 327.1 326.09<br>325.12 324.08 323.1 322.09 320.08 319.09 317.08 316.07 315.1<br>314.09 313.08 312.07 310.09 309.1 308.09 307.08 306.07 303.1<br>301.08 300.07 299.07 298.06 296.08 294.1 287.1 285.09 283.07<br>281.08 280.07 279.09 268.08 267.09 265.08 264.07 263.07 262.06<br>255.09 253.07 252.06 251.07 249.08 247.07 245.06 239.09 237.08<br>235.07 233.06 223.07 220.06 211.07 209.06 208.05 207.05 205.06<br>196.06 195.05 194.04 193.04 191.06 182.08 181.05 180.04 179.03<br>178.05 177.04 176.03 175.06 166.09 165.05 164.05 163.04 162.03<br>150.05 149.06 148.05 147.04 139.04 135.04 124.04 123.04 122.04<br>121.03 119.05 118.04 111.04 110.06 109.03 108.02 107.05 106.04
**References**	Minale, L.; Piattelli, M.; de Stefano, S. Pigments of Centrospermae VII. Betacyanins from *Gomphrena globosa* L. *Phytochemistry* **1967**, 6, 703–708.<br>Heuer, S.; Wray, V.; Metzger, J.W.; Strack, D. Betacyanins from flowers of *Gomphrena globosa*. *Phytochemistry* **1992**, 31, 1801–1807.<br>Wu, J.Y.; Chen, W.C.; Wu, Y.Y.; Chen, J.T.; Chen, L.G.; Chiou, R.Y.Y. NMR-based elucidation of the positional C6-*O*-glucopyranosyl substitution of gomphrenin I isolated from *Basella alba* fruits. *J. Sci. Food Agric.* **2013**, 2, 8–11.<br>Spórna-Kucab, A.; Wróbel, N.; Kumorkiewicz-Jamro, A.; Wybraniec, S. Separation of betacyanins from Iresine herbstii Hook. ex Lindl. leaves by high-speed countercurrent chromatography in a polar solvent system. *J. Chromatogr. A* **2020**, 1626, 461370.<br>Slimen, I.B.; Najar, T.; Abderrabba, M. Chemical and Antioxidant Properties of Betalains. *J. Agric. Food Chem.* **2017**, 65, 675–689.<br>Kumorkiewicz-Jamro, A.; Świergosz, T.; Sutor, K.; Spórna-Kucab, A.; Wybraniec, S. Multi-colored shades of betalains: Recent advances in betacyanin chemistry. *Nat. Prod. Rep.* **2021**, 38, 2315–2346.

Gomphrenin-III

**Chemical structure**	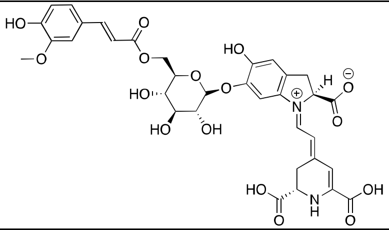
**Name:**	Gomphrenin-III
**Chemical Formula**	$C_{34}H_{34}N_2O_{16}$
**Molecular weight**	726.64
**Monoisotopic mass**	726.1908
***m/z* [M+H]**	727.1981
**Theorical fragments (*m/z*)**	727.2 726.19 725.18 724.17 711.2 710.2 709.19 708.18 707.17 699.2<br>698.2 697.19 695.21 694.2 693.19 692.18 691.18 690.17 689.16<br>687.2 685.19 684.18 683.21 682.2 681.19 680.18 679.18 677.2<br>675.18 673.17 671.15 669.19 667.21 666.21 665.2 664.19 663.18<br>662.17 661.17 659.19 657.19 655.18 654.17 653.2 651.18 649.2<br>647.19 645.17 641.2 639.18 638.17 637.17 636.16 635.19 633.17<br>623.19 621.17 619.16 603.16 601.15 558.16 545.15 544.14 542.17<br>540.15 534.16 533.14 532.14 531.14 529.16 528.15 527.14 519.15<br>518.14 517.15 516.15 515.13 514.13 513.11 506.17 505.15 504.14<br>503.13 502.15 501.14 500.13 499.13 498.14 497.12 496.12 495.1<br>493.15 491.13 489.15 488.16 487.13 486.14 485.14 484.14 483.13<br>482.12 481.12 479.11 476.16 475.13 474.14 473.14 472.14 471.14<br>470.14 469.12 464.16 463.13 462.14 461.14 460.11 459.14 457.15<br>455.13 449.14 447.13 446.12 445.15 444.13 443.13 433.15 431.13<br>430.13 429.12 428.11 427.14 425.12 415.14 413.12 411.11 389.1<br>388.09 387.08 386.07 375.12 374.11 373.1 372.1 371.09 370.08<br>369.07 364.1 361.1 360.1 359.12 358.12 357.11 356.1 355.09<br>354.08 353.08 351.06 349.1 347.09 345.11 344.1 343.13 342.08<br>341.11 340.1 339.1 338.09 337.08 336.07 335.09 334.08 331.09<br>330.08 329.11 327.1 326.09 325.12 324.08 323.1 322.09 320.08<br>319.09 317.08 316.07 315.1 314.09 313.08 312.07 310.09 308.09<br>307.08 306.07 303.1 301.08 300.07 299.07 298.06 296.08 294.1<br>287.1 285.09 283.07 281.08 280.07 279.09 268.08 267.09 265.08<br>264.07 263.07 262.06 255.09 253.07 252.06 251.07 249.08 247.07<br>245.06 239.09 237.08 235.07 233.06 223.07 220.06 211.07 209.06<br>208.05 207.05 205.06 196.06 195.05 194.04 193.04 191.06 182.08<br>181.05 180.04 179.03 178.05 177.04 176.03 175.06 166.09 165.05<br>164.05 163.04 162.03 150.05 149.06 148.05 147.04 139.04 135.04<br>124.04 123.04 122.04 121.03 119.05 118.04 111.04 110.06 109.03<br>108.02 107.05 106.04
**References**	Minale, L.; Piattelli, M.; de Stefano, S. Pigments of Centrospermae VII. Betacyanins from *Gomphrena globosa* L. *Phytochemistry* **1967**, 6, 703–708.<br>Heuer, S.; Wray, V.; Metzger, J.W.; Strack, D. Betacyanins from flowers of *Gomphrena globosa*. *Phytochemistry* **1992**, 31, 1801–1807.<br>Wu, J.Y.; Chen, W.C.; Wu, Y.Y.; Chen, J.T.; Chen, L.G.; Chiou, R.Y.Y. NMR-based elucidation of the positional C6-*O*-glucopyranosyl substitution of gomphrenin I isolated from *Basella alba* fruits. *J. Sci. Food Agric.* **2013**, 2, 8–11.<br>Spórna-Kucab, A.; Wróbel, N.; Kumorkiewicz-Jamro, A.; Wybraniec, S. Separation of betacyanins from Iresine herbstii Hook. ex Lindl. leaves by high-speed countercurrent chromatography in a polar solvent system. *J. Chromatogr. A* **2020**, 1626, 461370.<br>Slimen, I.B.; Najar, T.; Abderrabba, M. Chemical and Antioxidant Properties of Betalains. *J. Agric. Food Chem.* **2017**, 65, 675–689.<br>Kumorkiewicz-Jamro, A.; Świergosz, T.; Sutor, K.; Spórna-Kucab, A.; Wybraniec, S. Multi-colored shades of betalains: Recent advances in betacyanin chemistry. *Nat. Prod. Rep.* **2021**, 38, 2315–2346.

Isogomphrenin-III

**Chemical structure**	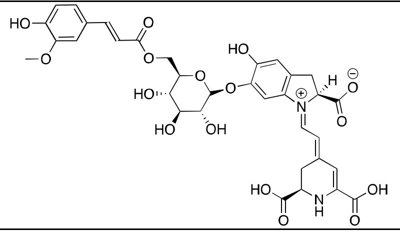
**Name:**	Isogomphrenin-III
**Chemical Formula**	$C_{34}H_{34}N_2O_{16}$
**Molecular weight**	726.64
**Monoisotopic mass**	726.1908
***m/z* [M+H]**	727.1981
**Theorical fragments (*m/z*)**	727.2 726.19 725.18 724.17 711.2 710.2 709.19 708.18 707.17 699.2<br>698.2 697.19 695.21 694.2 693.19 692.18 691.18 690.17 689.16<br>687.2 685.19 684.18 683.21 682.2 681.19 680.18 679.18 677.2<br>675.18 673.17 671.15 669.19 667.21 666.21 665.2 664.19 663.18<br>662.17 661.17 659.19 657.19 655.18 654.17 653.2 651.18 649.2<br>647.19 645.17 641.2 639.18 638.17 637.17 636.16 635.19 633.17<br>623.19 621.17 619.16 603.16 601.15 558.16 545.15 544.14 542.17<br>540.15 534.16 533.14 532.14 531.14 529.16 528.15 527.14 519.15<br>518.14 517.15 516.15 515.13 514.13 513.11 506.17 505.15 504.14<br>503.13 502.15 501.14 500.13 499.13 498.14 497.12 496.12 495.1<br>493.15 491.13 489.15 488.16 487.13 486.14 485.14 484.14 483.13<br>482.12 481.12 479.11 476.16 475.13 474.14 473.14 472.14 471.14<br>470.14 469.12 464.16 463.13 462.14 461.14 460.11 459.14 457.15<br>455.13 449.14 447.13 446.12 445.15 444.13 443.13 433.15 431.13<br>430.13 429.12 428.11 427.14 425.12 415.14 413.12 411.11 389.1<br>388.09 387.08 386.07 375.12 374.11 373.1 372.1 371.09 370.08<br>369.07 364.1 361.1 360.1 359.12 358.12 357.11 356.1 355.09<br>354.08 353.08 351.06 349.1 347.09 345.11 344.1 343.13 342.08<br>341.11 340.1 339.1 338.09 337.08 336.07 335.09 334.08 331.09<br>330.08 329.11 327.1 326.09 325.12 324.08 323.1 322.09 320.08<br>319.09 317.08 316.07 315.1 314.09 313.08 312.07 310.09 308.09<br>307.08 306.07 303.1 301.08 300.07 299.07 298.06 296.08 294.1<br>287.1 285.09 283.07 281.08 280.07 279.09 268.08 267.09 265.08<br>264.07 263.07 262.06 255.09 253.07 252.06 251.07 249.08 247.07<br>245.06 239.09 237.08 235.07 233.06 223.07 220.06 211.07 209.06<br>208.05 207.05 205.06 196.06 195.05 194.04 193.04 191.06 182.08<br>181.05 180.04 179.03 178.05 177.04 176.03 175.06 166.09 165.05<br>164.05 163.04 162.03 150.05 149.06 148.05 147.04 139.04 135.04<br>124.04 123.04 122.04 121.03 119.05 118.04 111.04 110.06 109.03<br>108.02 107.05 106.04
**References**	Minale, L.; Piattelli, M.; de Stefano, S. Pigments of Centrospermae VII. Betacyanins from *Gomphrena globosa* L. *Phytochemistry* **1967**, 6, 703–708.<br>Heuer, S.; Wray, V.; Metzger, J.W.; Strack, D. Betacyanins from flowers of *Gomphrena globosa*. *Phytochemistry* **1992**, 31, 1801–1807.<br>Wu, J.Y.; Chen, W.C.; Wu, Y.Y.; Chen, J.T.; Chen, L.G.; Chiou, R.Y.Y. NMR-based elucidation of the positional C6-*O*-glucopyranosyl substitution of gomphrenin I isolated from *Basella alba* fruits. *J. Sci. Food Agric.* **2013**, 2, 8–11.<br>Spórna-Kucab, A.; Wróbel, N.; Kumorkiewicz-Jamro, A.; Wybraniec, S. Separation of betacyanins from Iresine herbstii Hook. ex Lindl. leaves by high-speed countercurrent chromatography in a polar solvent system. *J. Chromatogr. A* **2020**, 1626, 461370.<br>Slimen, I.B.; Najar, T.; Abderrabba, M. Chemical and Antioxidant Properties of Betalains. *J. Agric. Food Chem.* **2017**, 65, 675–689.<br>Kumorkiewicz-Jamro, A.; Świergosz, T.; Sutor, K.; Spórna-Kucab, A.; Wybraniec, S. Multi-colored shades of betalains: Recent advances in betacyanin chemistry. *Nat. Prod. Rep.* **2021**, 38, 2315–2346.

Gomphrenin-IV

**Chemical structure**	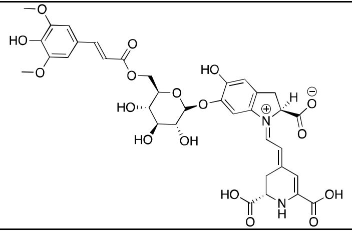
**Name:**	Gomphrenin-IV
**Chemical Formula**	$C_{35}H_{36}N_2O_{17}$
**Molecular weight**	756.67
**Monoisotopic mass**	756.2014
***m/z* [M+H]**	757.2087
**Theorical fragments (*m/z*)**	757.21 756.2 755.19 754.19 741.21 740.21 739.2 738.19 737.18 729.21 728.21 727.2 725.22 724.21 723.2 722.2 721.19 720.18 719.17 717.21 715.2 714.19 713.22 712.21 711.2 710.2 709.19 707.21 705.19 703.18 701.16 699.2 697.22 696.22 695.21 694.2 693.19 692.18 691.18 689.2 687.2 685.19 684.18 683.21 681.19 679.21 677.2 675.18 671.21 669.19 668.18 667.18 666.17 665.2 663.18 653.2 651.18 649.17 633.17 631.16 588.17 575.16 574.16 572.18 570.16 564.17 563.16 562.16 561.15 559.17 558.16 557.15 549.16 548.15 547.14 546.16 545.15 544.14 543.14 536.18 534.16 533.14 532.16 531.12 530.14 528.15 526.13 519.15 518.17 517.15 516.15 515.13 514.15 513.11 512.13 506.17 505.15 504.14 503.15 502.15 501.15 500.16 499.13 497.12 495.1 494.17 493.15 492.15 491.15 489.15 488.14 487.13 486.13 485.14 483.14 481.12 479.15 477.14 476.13 475.16 474.14 473.14 471.14 469.12 463.16 461.12 460.14 459.13 458.12 457.15 455.13 447.14 445.12 443.11 441.12 427.11 425.1 389.1 388.09 387.08 386.07 375.12 374.11 373.1 372.1 371.09 370.08 369.07 364.1 361.1 360.1 359.12 358.12 357.11 356.1 355.09 354.08 353.08 351.06 349.1 347.09 345.11 344.1 343.13 342.08 341.11 340.1 339.1 338.09 337.08 336.07 335.09 334.08 331.09 330.08 329.11 327.1 326.09 325.12 324.08 323.1 322.09 320.08 319.09 317.08 316.07 315.1 314.09 313.08 312.07 310.09 308.09 307.08 306.07 303.1 301.08 300.07 299.07 298.06 296.08 294.1 287.1 285.09 283.07 281.08 280.07 279.09 268.08 267.09 265.08 264.07 263.07 262.06 255.09 253.07 252.06 251.07 249.08 247.07 245.06 239.09 237.08 235.07 233.06 223.07 220.06 211.07 209.06 208.05 207.05 205.06 196.06 195.05 194.04 193.04 191.06 182.08 181.05 180.04 179.03 178.05 177.04 176.03 175.06 166.09 165.05 164.05 163.04 162.03 150.05 149.06 148.05 147.04 139.04 135.04 124.04 123.04 122.04 121.03 119.05 118.04 111.04 110.06 109.03 108.02 107.05 106.04
**References**	Minale, L.; Piattelli, M.; de Stefano, S. Pigments of Centrospermae VII. Betacyanins from *Gomphrena globosa* L. *Phytochemistry* **1967**, 6, 703–708.<br>Heuer, S.; Wray, V.; Metzger, J.W.; Strack, D. Betacyanins from flowers of *Gomphrena globosa*. *Phytochemistry* **1992**, 31, 1801–1807.<br>Wu, J.Y.; Chen, W.C.; Wu, Y.Y.; Chen, J.T.; Chen, L.G.; Chiou, R.Y.Y. NMR-based elucidation of the positional C6-*O*-glucopyranosyl substitution of gomphrenin I isolated from *Basella alba* fruits. *J. Sci. Food Agric.* **2013**, 2, 8–11.<br>Spórna-Kucab, A.; Wróbel, N.; Kumorkiewicz-Jamro, A.; Wybraniec, S. Separation of betacyanins from Iresine herbstii Hook. ex Lindl. leaves by high-speed countercurrent chromatography in a polar solvent system. *J. Chromatogr. A* **2020**, 1626, 461370.<br>Slimen, I.B.; Najar, T.; Abderrabba, M. Chemical and Antioxidant Properties of Betalains. *J. Agric. Food Chem.* **2017**, 65, 675–689.<br>Kumorkiewicz-Jamro, A.; Świergosz, T.; Sutor, K.; Spórna-Kucab, A.; Wybraniec, S. Multi-colored shades of betalains: Recent advances in betacyanin chemistry. *Nat. Prod. Rep.* **2021**, 38, 2315–2346.

Isogomphrenin-IV

**Chemical structure**	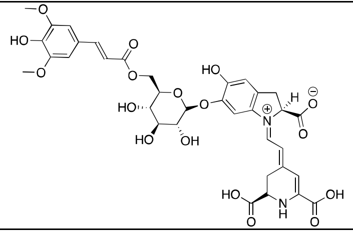
**Name:**	Isogomphrenin-IV
**Chemical Formula**	$C_{35}H_{36}N_2O_{17}$
**Molecular weight**	756.67
**Monoisotopic mass**	756.2014
***m/z* [M+H]**	757.2087
**Theorical fragments (*m/z*)**	757.21 756.2 755.19 754.19 741.21 740.21 739.2 738.19 737.18 729.21 728.21 727.2 725.22 724.21 723.2 722.2 721.19 720.18 719.17 717.21 715.2 714.19 713.22 712.21 711.2 710.2 709.19 707.21 705.19 703.18 701.16 699.2 697.22 696.22 695.21 694.2 693.19 692.18 691.18 689.2 687.2 685.19 684.18 683.21 681.19 679.21 677.2 675.18 671.21 669.19 668.18 667.18 666.17 665.2 663.18 653.2 651.18 649.17 633.17 631.16 588.17 575.16 574.16 572.18 570.16 564.17 563.16 562.16 561.15 559.17 558.16 557.15 549.16 548.15 547.14 546.16 545.15 544.14 543.14 536.18 534.16 533.14 532.16 531.12 530.14 528.15 526.13 519.15 518.17 517.15 516.15 515.13 514.15 513.11 512.13 506.17 505.15 504.14 503.15 502.15 501.15 500.16 499.13 497.12 495.1 494.17 493.15 492.15 491.15 489.15 488.14 487.13 486.13 485.14 483.14 481.12 479.15 477.14 476.13 475.16 474.14 473.14 471.14 469.12 463.16 461.12 460.14 459.13 458.12 457.15 455.13 447.14 445.12 443.11 441.12 427.11 425.1 389.1 388.09 387.08 386.07 375.12 374.11 373.1 372.1 371.09 370.08 369.07 364.1 361.1 360.1 359.12 358.12 357.11 356.1 355.09 354.08 353.08 351.06 349.1 347.09 345.11 344.1 343.13 342.08 341.11 340.1 339.1 338.09 337.08 336.07 335.09 334.08 331.09 330.08 329.11 327.1 326.09 325.12 324.08 323.1 322.09 320.08 319.09 317.08 316.07 315.1 314.09 313.08 312.07 310.09 308.09 307.08 306.07 303.1 301.08 300.07 299.07 298.06 296.08 294.1 287.1 285.09 283.07 281.08 280.07 279.09 268.08 267.09 265.08 264.07 263.07 262.06 255.09 253.07 252.06 251.07 249.08 247.07 245.06 239.09 237.08 235.07 233.06 223.07 220.06 211.07 209.06 208.05 207.05 205.06 196.06 195.05 194.04 193.04 191.06 182.08 181.05 180.04 179.03 178.05 177.04 176.03 175.06 166.09 165.05 164.05 163.04 162.03 150.05 149.06 148.05 147.04 139.04 135.04 124.04 123.04 122.04 121.03 119.05 118.04 111.04 110.06 109.03 108.02 107.05 106.04
**References**	Minale, L.; Piattelli, M.; de Stefano, S. Pigments of Centrospermae VII. Betacyanins from *Gomphrena globosa* L. *Phytochemistry* **1967**, 6, 703–708.<br>Heuer, S.; Wray, V.; Metzger, J.W.; Strack, D. Betacyanins from flowers of *Gomphrena globosa*. *Phytochemistry* **1992**, 31, 1801–1807.<br>Wu, J.Y.; Chen, W.C.; Wu, Y.Y.; Chen, J.T.; Chen, L.G.; Chiou, R.Y.Y. NMR-based elucidation of the positional C6-*O*-glucopyranosyl substitution of gomphrenin I isolated from *Basella alba* fruits. *J. Sci. Food Agric.* **2013**, 2, 8–11.<br>Spórna-Kucab, A.; Wróbel, N.; Kumorkiewicz-Jamro, A.; Wybraniec, S. Separation of betacyanins from Iresine herbstii Hook. ex Lindl. leaves by high-speed countercurrent chromatography in a polar solvent system. *J. Chromatogr. A* **2020**, 1626, 461370.<br>Slimen, I.B.; Najar, T.; Abderrabba, M. Chemical and Antioxidant Properties of Betalains. *J. Agric. Food Chem.* **2017**, 65, 675–689.<br>Kumorkiewicz-Jamro, A.; Świergosz, T.; Sutor, K.; Spórna-Kucab, A.; Wybraniec, S. Multi-colored shades of betalains: Recent advances in betacyanin chemistry. *Nat. Prod. Rep.* **2021**, 38, 2315–2346.

2-decarboxy-2,3-dehydro-gomphrenin

**Chemical structure**	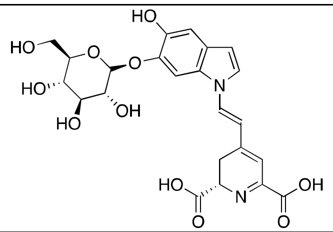
**Name:**	2-decarboxy-2,3-dehydro-gomphrenin (2-decarboxy-xangomphrenin)
**Chemical Formula**	$C_{23}H_{24}N_2O_{11}$
**Molecular weight**	504.45
**Monoisotopic mass**	504.1380
***m/z* [M+H]**	505.1453
**Theorical fragments (*m/z*)**	505.15 504.14 503.13 502.12 501.11 500.11 489.15 488.14 487.13 486.13<br>485.12 484.11 483.1 479.13 478.12 477.15 475.13 474.13 473.12<br>472.11 471.14 470.13 469.12 468.12 467.11 465.15 463.13 461.16<br>460.11 459.14 458.1 457.12 456.12 455.11 454.1 453.13 451.11<br>449.12 447.14 445.16 444.12 443.14 442.1 441.13 440.12 439.11<br>438.11 437.1 435.12 434.14 433.12 432.13 431.11 430.1 429.09<br>428.12 427.11 426.11 425.1 424.09 423.12 419.12 415.11 414.11<br>413.1 412.09 411.08 410.11 409.1 408.1 403.11 401.1 400.09<br>399.08 398.11 397.1 396.1 395.09 389.1 385.1 384.1 383.09<br>382.08 381.07 376.14 374.12 373.1 371.09 369.07 368.1 367.09<br>366.08 362.12 357.11 355.09 354.08 353.08 343.09 342.08 341.08<br>339.1 338.09 337.08 336.11 329.11 327.1 326.09 325.08 324.07<br>323.07 318.1 317.08 315.1 313.12 312.11 311.1 310.09 309.09<br>307.07 303.1 301.08 300.09 299.1 297.09 296.11 295.11 294.1<br>293.09 287.1 286.09 285.09 283.11 281.09 276.09 271.08 270.07<br>254.08 253.07 194.04 180.06 179.06 178.05 177.04 176.03 175.02<br>174.05 168.03 165.08 163.06 162.05 161.04 160.04 159.03 152.03<br>151.06 150.02 149.04 148.04 147.07 146.06 145.05 144.04 143.03<br>139.06 136.08 135.07 134.06 133.05 132.04 131.04 130.03 129.05<br>128.03 127.04 126.02 124.04 122.06 121.05 120.04 119.03 118.07<br>117.02 116.05 115.04 114.02 113.02 110.06 109.03 108.04 107.07<br>105.05 104.05 103.04 102.03 101.02 100.04
**References**	Kumorkiewicz-Jamro, A.; Świergosz, T.; Sutor, K.; Spórna-Kucab, A.; Wybraniec, S. Multi-colored shades of betalains: Recent advances in betacyanin chemistry. *Nat. Prod. Rep.* **2021**, 38, 2315–2346.

Neogomphrenin

**Chemical structure**	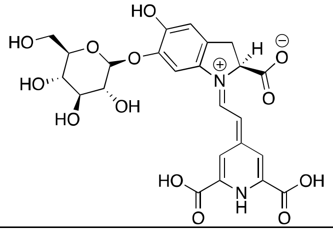
**Name:**	Neogomphrenin
**Chemical Formula**	$C_{24}H_{24}N_2O_{13}$
**Molecular weight**	548.46
**Monoisotopic mass**	548.1278
***m/z* [M+H]**	549.1351
**Theorical fragments (*m/z*)**	549.14 548.13 547.12 546.11 533.14 532.13 531.12 530.12 529.11 521.14 520.13 519.12 517.15 516.14 515.13 514.12 513.11 512.11 511.1 509.14 507.12 506.12 505.15 504.14 503.13 502.12 501.11 499.13 497.12 495.1 493.09 491.13 490.12 489.15 488.14 487.13 485.12 484.11 483.1 479.13 477.11 476.11 473.12 471.14 469.12 467.11 461.12 459.1 458.1 455.11 451.11 443.11 441.09 433.12 423.08 417.09 387.08 386.07 385.07 384.06 373.1 372.1 371.09 370.08 369.07 368.06 367.06 359.09 358.08 357.11 356.1 355.09 354.08 353.08 352.07 351.09 350.09 347.09 345.07 343.09 342.09 341.11 340.1 339.1 338.09 337.08 336.07 334.09 333.07 332.06 329.08 328.07 327.1 326.1 325.09 324.08 323.08 322.09 321.08 320.08 319.07 317.08 315.06 314.05 313.09 312.07 311.1 310.09 309.1 308.09 307.08 306.07 303.1 302.07 301.08 299.07 298.09 297.1 296.09 295.07 294.1 293.09 285.1 283.07 281.1 279.06 278.05 277.05 276.09 273.1 271.08 270.07 269.1 267.09 265.05 264.04 263.07 262.06 261.05 257.1 255.09 254.08 253.07 252.06 251.09 249.05 247.03 239.09 237.08 235.06 233.06 221.06 209.06 207.05 206.03 194.04 193.04 192.03 191.06 190.05 181.05 180.04 179.03 178.05 177.04 176.03 175.06 165.05 164.05 163.04 162.03 150.05 149.06 148.05 147.04 146.04 145.05 141.05 139.04 136.08 135.04 134.06 132.04 123.04 122.04 121.03 119.05 118.04 111.04 110.06 109.03 108.02 107.05 106.04 105.03
**References**	Kumorkiewicz-Jamro, A.; Świergosz, T.; Sutor, K.; Spórna-Kucab, A.; Wybraniec, S. Multi-colored shades of betalains: Recent advances in betacyanin chemistry. *Nat. Prod. Rep.* **2021**, 38, 2315–2346.

Neogomphrenin III

**Chemical structure**	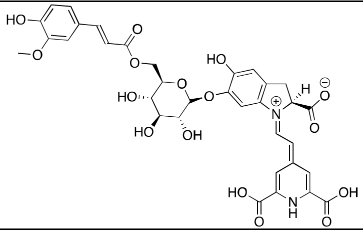
**Name:**	Neogomphrenin III
**Chemical Formula**	$C_{34}H_{32}N_2O_{16}$
**Molecular weight**	724.63
**Monoisotopic mass**	724.1752
***m/z* [M+H]**	725.1825
**Theorical fragments (*m/z*)**	725.18 724.17 723.17 722.16 709.19 708.18 707.17 706.16 705.16 697.19 696.18 695.17 693.19 692.18 691.18 690.17 689.16 688.15 687.15 685.19 683.17 682.16 681.19 680.18 679.18 678.17 677.16 675.18 673.17 671.15 669.14 667.18 666.17 665.2 664.19 663.18 661.17 660.16 659.15 655.18 653.16 652.15 649.17 647.19 645.17 643.16 637.17 635.15 634.14 631.16 619.16 617.14 609.17 545.15 544.14 543.14 532.14 531.12 530.12 529.11 528.15 527.14 526.13 519.15 518.14 517.13 516.15 515.13 514.13 513.11 512.11 511.1 510.14 503.13 502.15 501.14 500.13 499.13 498.14 497.12 496.12 495.1 493.09 491.13 489.14 488.16 487.15 486.14 485.12 484.14 483.13 482.12 479.11 477.09 475.12 474.14 473.14 472.14 471.14 470.14 469.12 467.11 461.14 459.1 458.1 457.15 455.13 452.13 451.11 449.14 447.13 446.12 445.15 443.13 441.09 433.15 431.13 430.13 429.12 428.11 427.14 425.12 423.08 417.09 415.14 413.12 411.11 387.08 386.07 385.07 384.06 373.1 372.1 371.09 370.08 369.07 368.06 367.06 359.09 358.08 357.11 356.1 355.09 354.08 353.08 352.07 351.09 350.09 347.09 345.07 343.09 342.08 341.11 340.07 339.1 338.09 337.08 336.07 334.09 333.07 332.06 329.08 328.07 327.1 325.08 324.08 323.08 322.09 321.08 320.08 319.07 317.08 315.06 314.05 312.07 308.09 307.08 306.07 303.1 302.07 301.08 299.07 295.07 294.1 293.09 283.07 281.05 279.06 278.05 277.05 276.09 267.09 265.05 264.04 263.07 262.06 261.05 257.09 255.09 253.07 252.06 251.09 249.05 247.03 239.09 237.08 235.06 233.06 221.06 209.06 207.05 206.03 194.04 193.04 192.03 191.06 190.05 181.05 180.04 179.03 178.05 177.04 176.03 175.06 165.05 164.05 163.04 162.03 150.05 149.06 148.05 147.04 146.04 145.05 141.05 139.04 136.08 135.04 134.06 132.04 123.04 122.04 121.03 119.05 118.04 111.04 110.06 109.03 108.02 107.05 106.04 105.03
**References**	Kumorkiewicz-Jamro, A.; Świergosz, T.; Sutor, K.; Spórna-Kucab, A.; Wybraniec, S. Multi-colored shades of betalains: Recent advances in betacyanin chemistry. *Nat. Prod. Rep.* **2021**, 38, 2315–2346.

Neogomphrenin IV

**Chemical structure**	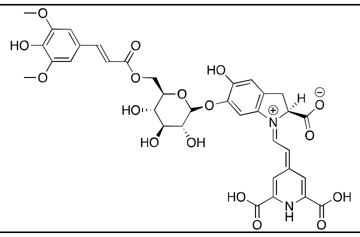
**Name:**	Neogomphrenin IV
**Chemical Formula**	$C_{35}H_{34}N_2O_{17}$
**Molecular weight**	754.65
**Monoisotopic mass**	754.1857
***m/z* [M+H]**	755.1930
**Theorical fragments (*m/z*)**	755.19 754.19 753.18 752.17 739.2 738.19 737.18 736.17 735.17 727.2<br>726.19 725.18 723.2 722.2 721.19 720.18 719.17 718.16 717.16<br>715.2 713.18 712.17 711.2 710.2 709.19 708.18 707.17 705.19<br>703.18 701.16 699.15 697.19 696.18 695.21 694.2 693.19 691.18<br>690.17 689.16 685.19 683.17 682.16 679.18 677.2 675.18 673.17<br>667.18 665.16 664.15 661.17 649.17 647.15 639.18 575.16 574.16<br>573.15 562.16 561.15 559.17 558.16 557.15 556.14 549.16 548.15<br>547.14 546.16 545.15 544.14 543.14 540.15 533.17 532.16 531.12<br>530.14 529.11 528.15 527.14 526.13 525.13 519.15 518.17 517.16<br>516.15 515.13 514.15 513.11 512.13 511.1 505.13 504.15 503.15<br>502.15 501.11 500.16 499.13 497.12 495.1 493.09 491.15 489.11<br>487.13 485.12 484.11 483.1 482.14 479.15 477.14 476.13 475.16<br>473.14 471.14 469.12 467.11 463.16 461.14 460.14 459.1 458.12<br>457.15 455.13 451.11 445.15 443.13 441.09 423.08 417.09 387.08<br>386.07 385.07 384.06 373.1 372.1 371.09 370.08 369.07 368.06<br>367.06 359.09 358.08 357.11 356.1 355.09 354.08 353.08 352.07<br>351.09 350.09 347.09 345.07 343.09 342.08 341.11 340.07 339.1<br>338.09 337.08 336.07 334.09 333.07 332.06 329.08 328.07 327.1<br>325.08 324.08 323.08 322.09 321.08 320.08 319.07 317.08 315.06<br>314.05 312.07 308.09 307.08 306.07 303.1 302.07 301.08 299.07<br>295.07 294.1 293.09 283.07 281.05 279.06 278.05 277.05 276.09<br>267.09 265.05 264.04 263.07 262.06 261.05 257.09 255.09 253.07<br>252.06 251.09 249.05 247.03 239.09 237.08 235.06 233.06 221.06<br>209.06 207.05 206.03 194.04 193.04 192.03 191.06 190.05 181.05<br>180.04 179.03 178.05 177.04 176.03 175.06 165.05 164.05 163.04<br>162.03 150.05 149.06 148.05 147.04 146.04 145.05 141.05 139.04<br>136.08 135.04 134.06 132.04 123.04 122.04 121.03 119.05 118.04<br>111.04 110.06 109.03 108.02 107.05 106.04 105.03
**References**	Kumorkiewicz-Jamro, A.; Świergosz, T.; Sutor, K.; Spórna-Kucab, A.; Wybraniec, S. Multi-colored shades of betalains: Recent advances in betacyanin chemistry. *Nat. Prod. Rep.* **2021**, 38, 2315–2346.

Glabranin-type
Glabranin

**Chemical structure**	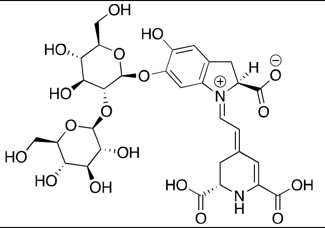
**Name:**	Glabranin (Betanidin 6'-*O*-β-D-sophoroside)
**Chemical Formula**	$C_{30}H_{36}N_2O_{18}$
**Molecular weight**	712.61
**Monoisotopic mass**	712.1963
***m/z* [M+H]**	713.2036
**Theorical fragments (*m/z*)**	713.2 712.2 711.19 710.18 697.21 696.2 695.19 694.19 693.18 685.21<br>684.2 683.19 681.21 680.21 679.2 678.19 677.18 676.17 675.17<br>673.21 671.19 669.21 668.21 667.2 666.19 665.18 663.2 661.19<br>659.17 657.16 655.2 653.22 651.2 650.2 649.19 648.18 647.17<br>643.2 641.18 640.17 639.2 637.19 633.19 631.18 627.2 625.19<br>624.18 623.17 622.16 621.19 619.18 609.19 607.18 605.16 589.17<br>587.15 551.15 550.14 549.14 544.17 535.16 534.15 533.14 532.13<br>531.12 523.16 522.15 521.14 520.17 519.16 518.15 517.14 516.14<br>515.13 514.12 513.11 511.16 509.14 507.16 505.15 504.15 503.13<br>502.16 501.15 500.14 499.13 497.12 495.1 493.15 491.13 489.15<br>488.15 487.14 486.14 485.12 481.15 479.13 478.12 477.15 475.13<br>474.16 473.15 472.14 471.15 470.14 469.13 468.13 465.15 463.13<br>461.12 460.11 459.15 458.14 457.12 448.14 447.15 445.12 443.11<br>441.14 435.15 433.13 432.13 431.15 429.14 427.11 425.1 419.15<br>417.14 416.13 415.12 414.12 413.14 411.13 401.14 399.13 397.11<br>389.1 388.09 387.08 386.07 375.12 374.11 373.1 372.1 371.09<br>370.08 369.07 361.1 360.1 359.12 358.11 357.11 356.1 355.09<br>354.08 353.08 351.06 349.1 347.09 345.11 343.13 342.09 341.11<br>340.1 339.1 338.09 337.08 336.07 335.09 331.09 330.08 329.11<br>327.1 326.09 325.09 324.08 323.1 319.09 317.08 316.07 315.1<br>314.09 313.08 312.07 310.09 308.09 307.08 306.07 303.1 301.08<br>300.07 299.07 298.06 297.1 296.08 287.1 285.1 283.07 281.08<br>280.07 279.09 273.1 271.08 270.07 269.1 267.09 265.08 264.07<br>263.07 262.06 257.1 255.09 253.07 252.06 251.07 249.08 247.07<br>245.06 239.09 237.08 235.07 233.06 223.07 211.07 196.06 195.05<br>194.04 193.04 182.08 180.04 179.03 178.05 177.04 165.05 164.05<br>163.04 162.03 149.06 148.05 147.04 139.04 135.04 123.04 122.04<br>119.05 118.04 111.04 109.03 108.02 107.05 106.04
**References**	Piattelli, M; Imperato, F. Pigments of *Bougainvillea glabra. Phytochemistry* **1970**, 9, 2557–2560.<br>Heuer, S.; Richter, S.; Metzger, J.W.; Wray, V.; Nimtz, M.; Strack, D. Betacyanins from bracts of *Bougainvillea glabra. Phytochemistry* **1994**, 37, 761–767.<br>Slimen, I.B.; Najar, T.; Abderrabba, M. Chemical and Antioxidant Properties of Betalains. *J. Agric. Food Chem.* **2017**, 65, 675–689.<br>Kumorkiewicz-Jamro, A.; Świergosz, T.; Sutor, K.; Spórna-Kucab, A.; Wybraniec, S. Multi-colored shades of betalains: Recent advances in betacyanin chemistry. *Nat. Prod. Rep.* **2021**, 38, 2315–2346.

Isoglabranin

**Chemical structure**	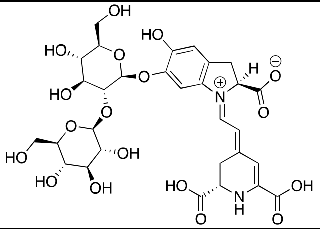
**Name:**	Isoglabranin (Isobetanidin 6’-*O*-β-D-sophoroside)
**Chemical Formula**	$C_{30}H_{36}N_2O_{18}$
**Molecular weight**	712.61
**Monoisotopic mass**	712.1963
***m/z* [M+H]**	713.2036
**Theorical fragments (*m/z*)**	713.2 712.2 711.19 710.18 697.21 696.2 695.19 694.19 693.18 685.21<br>684.2 683.19 681.21 680.21 679.2 678.19 677.18 676.17 675.17<br>673.21 671.19 669.21 668.21 667.2 666.19 665.18 663.2 661.19<br>659.17 657.16 655.2 653.22 651.2 650.2 649.19 648.18 647.17<br>643.2 641.18 640.17 639.2 637.19 633.19 631.18 627.2 625.19<br>624.18 623.17 622.16 621.19 619.18 609.19 607.18 605.16 589.17<br>587.15 551.15 550.14 549.14 544.17 535.16 534.15 533.14 532.13<br>531.12 523.16 522.15 521.14 520.17 519.16 518.15 517.14 516.14<br>515.13 514.12 513.11 511.16 509.14 507.16 505.15 504.15 503.13<br>502.16 501.15 500.14 499.13 497.12 495.1 493.15 491.13 489.15<br>488.15 487.14 486.14 485.12 481.15 479.13 478.12 477.15 475.13<br>474.16 473.15 472.14 471.15 470.14 469.13 468.13 465.15 463.13<br>461.12 460.11 459.15 458.14 457.12 448.14 447.15 445.12 443.11<br>441.14 435.15 433.13 432.13 431.15 429.14 427.11 425.1 419.15<br>417.14 416.13 415.12 414.12 413.14 411.13 401.14 399.13 397.11<br>389.1 388.09 387.08 386.07 375.12 374.11 373.1 372.1 371.09<br>370.08 369.07 361.1 360.1 359.12 358.11 357.11 356.1 355.09<br>354.08 353.08 351.06 349.1 347.09 345.11 343.13 342.09 341.11<br>340.1 339.1 338.09 337.08 336.07 335.09 331.09 330.08 329.11<br>327.1 326.09 325.09 324.08 323.1 319.09 317.08 316.07 315.1<br>314.09 313.08 312.07 310.09 308.09 307.08 306.07 303.1 301.08<br>300.07 299.07 298.06 297.1 296.08 287.1 285.1 283.07 281.08<br>280.07 279.09 273.1 271.08 270.07 269.1 267.09 265.08 264.07<br>263.07 262.06 257.1 255.09 253.07 252.06 251.07 249.08 247.07<br>245.06 239.09 237.08 235.07 233.06 223.07 211.07 196.06 195.05<br>194.04 193.04 182.08 180.04 179.03 178.05 177.04 165.05 164.05<br>163.04 162.03 149.06 148.05 147.04 139.04 135.04 123.04 122.04<br>119.05 118.04 111.04 109.03 108.02 107.05 106.04
**References**	Piattelli, M; Imperato, F. Pigments of *Bougainvillea glabra. Phytochemistry* **1970**, 9, 2557–2560.<br>Heuer, S.; Richter, S.; Metzger, J.W.; Wray, V.; Nimtz, M.; Strack, D. Betacyanins from bracts of *Bougainvillea glabra. Phytochemistry* **1994**, 37, 761–767.<br>Slimen, I.B.; Najar, T.; Abderrabba, M. Chemical and Antioxidant Properties of Betalains. *J. Agric. Food Chem.* **2017**, 65, 675–689.<br>Kumorkiewicz-Jamro, A.; Świergosz, T.; Sutor, K.; Spórna-Kucab, A.; Wybraniec, S. Multi-colored shades of betalains: Recent advances in betacyanin chemistry. *Nat. Prod. Rep.* **2021**, 38, 2315–2346.

Coumglabranin

**Chemical structure**	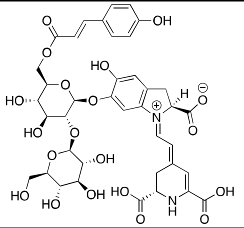
**Name:**	Coumglabranin (6'-*O*-*E*-4-Coumaroyl-glabranin)
**Chemical Formula**	$C_{39}H_{42}N_{2}O_{20}$
**Molecular weight**	858.76
**Monoisotopic mass**	858.2331
***m/z* [M+H]**	859.2403
**Theorical fragments (*m/z*)**	859.24 858.23 857.22 856.22 843.25 842.24 841.23 840.22 839.21 831.25<br>830.24 829.23 827.25 826.24 825.23 824.23 823.22 822.21 821.2<br>819.25 817.23 815.25 814.24 813.23 812.23 811.22 809.24 807.22<br>805.21 803.19 801.23 799.26 797.24 796.23 795.22 794.22 793.21<br>789.23 787.22 786.21 785.24 783.22 779.23 777.21 773.24 771.22<br>770.22 769.21 768.2 767.23 765.21 755.23 753.21 751.2 735.2<br>733.19 697.19 696.18 695.19 694.19 693.18 690.2 681.19 680.18<br>679.18 678.17 677.16 676.17 675.17 669.19 668.18 667.18 666.2<br>665.18 664.19 663.18 662.17 661.17 660.16 659.15 657.19 655.18<br>653.2 651.18 650.18 649.17 648.19 647.19 646.18 645.17 643.16<br>641.14 639.18 637.19 635.19 634.19 633.18 632.17 631.16 627.18<br>625.17 624.16 623.17 621.17 620.2 619.19 618.18 617.19 616.18<br>615.17 614.16 611.19 609.17 607.16 606.15 605.19 604.18 603.16<br>594.18 593.19 591.16 589.15 587.18 581.19 579.17 578.16 577.19<br>575.18 573.15 571.13 565.19 563.18 562.17 561.16 560.15 559.18<br>557.17 547.18 545.17 543.15 533.14 532.13 531.12 517.15 515.13<br>514.12 513.11 505.15 504.15 503.13 502.13 501.13 500.14 499.13<br>497.12 495.1 488.13 487.13 486.14 485.12 484.12 483.12 481.12<br>479.11 475.13 472.14 471.13 470.12 469.13 468.13 463.13 461.12<br>457.12 454.13 453.12 452.11 445.12 443.11 441.14 431.13 429.14<br>427.11 425.12 419.13 417.14 416.11 415.12 414.12 413.12 411.13<br>403.14 401.12 399.11 398.1 397.13 395.11 389.1 388.09 387.08<br>386.07 385.13 383.11 381.1 375.12 374.11 373.1 372.1 371.09<br>370.08 369.07 361.1 360.1 359.12 358.12 357.11 356.1 355.09<br>354.08 353.08 351.06 349.1 347.09 345.11 343.13 342.08 341.11<br>339.1 338.09 337.08 336.07 335.09 331.09 330.08 329.11 327.1<br>326.09 325.12 324.08 323.1 319.09 317.08 316.07 315.1 314.09<br>313.08 312.07 310.09 308.08 306.07 303.1 301.08 300.07 299.07<br>298.06 296.08 287.1 285.09 283.07 281.08 280.07 267.06 265.08<br>264.07 263.07 262.06 255.09 253.07 251.07 249.08 247.07 245.06<br>237.08 235.07 233.06 223.07 211.07 196.06 195.05 194.04 193.04<br>182.08 180.04 179.03 178.05 177.04 165.05 164.05 163.04 162.03<br>149.06 148.05 147.04 139.04 135.04 123.04 122.04 119.05 118.04<br>111.04 109.03 108.02 107.05 106.04
**References**	Heuer, S.; Richter, S.; Metzger, J.W.; Wray, V.; Nimtz, M.; Strack, D. Betacyanins from bracts of *Bougainvillea glabra. Phytochemistry* **1994**, 37, 761–767.<br>Slimen, I.B.; Najar, T.; Abderrabba, M. Chemical and Antioxidant Properties of Betalains. *J. Agric. Food Chem.* **2017**, 65, 675–689.<br>Kumorkiewicz-Jamro, A.; Świergosz, T.; Sutor, K.; Spórna-Kucab, A.; Wybraniec, S. Multi-colored shades of betalains: Recent advances in betacyanin chemistry. *Nat. Prod. Rep.* **2021**, 38, 2315–2346.

Isocoumglabranin

**Chemical structure**	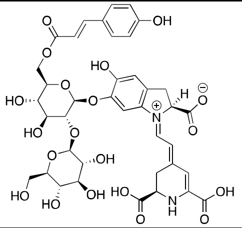
**Name:**	6’-O-E-4-Coumaroyl-isoglabranin (isocoumglabranin)
**Chemical Formula**	$C_{39}H_{42}N_2O_{20}$
**Molecular weight**	858.76
**Monoisotopic mass**	858.2331
***m/z* [M+H]**	859.2403
**Theorical fragments (*m/z*)**	859.24 858.23 857.22 856.22 843.25 842.24 841.23 840.22 839.21 831.25<br>830.24 829.23 827.25 826.24 825.23 824.23 823.22 822.21 821.2<br>819.25 817.23 815.25 814.24 813.23 812.23 811.22 809.24 807.22<br>805.21 803.19 801.23 799.26 797.24 796.23 795.22 794.22 793.21<br>789.23 787.22 786.21 785.24 783.22 779.23 777.21 773.24 771.22<br>770.22 769.21 768.2 767.23 765.21 755.23 753.21 751.2 735.2<br>733.19 697.19 696.18 695.19 694.19 693.18 690.2 681.19 680.18<br>679.18 678.17 677.16 676.17 675.17 669.19 668.18 667.18 666.2<br>665.18 664.19 663.18 662.17 661.17 660.16 659.15 657.19 655.18<br>653.2 651.18 650.18 649.17 648.19 647.19 646.18 645.17 643.16<br>641.14 639.18 637.19 635.19 634.19 633.18 632.17 631.16 627.18<br>625.17 624.16 623.17 621.17 620.2 619.19 618.18 617.19 616.18<br>615.17 614.16 611.19 609.17 607.16 606.15 605.19 604.18 603.16<br>594.18 593.19 591.16 589.15 587.18 581.19 579.17 578.16 577.19<br>575.18 573.15 571.13 565.19 563.18 562.17 561.16 560.15 559.18<br>557.17 547.18 545.17 543.15 533.14 532.13 531.12 517.15 515.13<br>514.12 513.11 505.15 504.15 503.13 502.13 501.13 500.14 499.13<br>497.12 495.1 488.13 487.13 486.14 485.12 484.12 483.12 481.12<br>479.11 475.13 472.14 471.13 470.12 469.13 468.13 463.13 461.12<br>457.12 454.13 453.12 452.11 445.12 443.11 441.14 431.13 429.14<br>427.11 425.12 419.13 417.14 416.11 415.12 414.12 413.12 411.13<br>403.14 401.12 399.11 398.1 397.13 395.11 389.1 388.09 387.08<br>386.07 385.13 383.11 381.1 375.12 374.11 373.1 372.1 371.09<br>370.08 369.07 361.1 360.1 359.12 358.12 357.11 356.1 355.09<br>354.08 353.08 351.06 349.1 347.09 345.11 343.13 342.08 341.11<br>339.1 338.09 337.08 336.07 335.09 331.09 330.08 329.11 327.1<br>326.09 325.12 324.08 323.1 319.09 317.08 316.07 315.1 314.09<br>313.08 312.07 310.09 308.08 306.07 303.1 301.08 300.07 299.07<br>298.06 296.08 287.1 285.09 283.07 281.08 280.07 267.06 265.08<br>264.07 263.07 262.06 255.09 253.07 251.07 249.08 247.07 245.06<br>237.08 235.07 233.06 223.07 211.07 196.06 195.05 194.04 193.04<br>182.08 180.04 179.03 178.05 177.04 165.05 164.05 163.04 162.03<br>149.06 148.05 147.04 139.04 135.04 123.04 122.04 119.05 118.04<br>111.04 109.03 108.02 107.05 106.04
**References**	Heuer, S.; Richter, S.; Metzger, J.W.; Wray, V.; Nimtz, M.; Strack, D. Betacyanins from bracts of *Bougainvillea glabra*. *Phytochemistry* **1994**, 37, 761–767.<br>Slimen, I.B.; Najar, T.; Abderrabba, M. Chemical and Antioxidant Properties of Betalains. *J. Agric. Food Chem.* **2017**, 65, 675–689.<br>Kumorkiewicz-Jamro, A.; Świergosz, T.; Sutor, K.; Spórna-Kucab, A.; Wybraniec, S. Multi-colored shades of betalains: Recent advances in betacyanin chemistry. *Nat. Prod. Rep.* **2021**, 38, 2315–2346.

Neocoumglabranin

**Chemical structure**	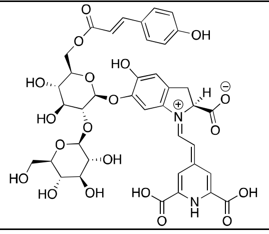
**Name:**	Neocoumglabranin
**Chemical Formula**	$C_{39}H_{40}N_2O_{20}$
**Molecular weight**	856.74
**Monoisotopic mass**	856.2174
***m/z* [M+H]**	857.2247
**Theorical fragments (*m/z*)**	857.22 856.22 855.21 854.2 841.23 840.22 839.21 838.21 837.2 829.23<br>828.22 827.21 825.23 823.22 822.21 821.2 820.2 819.19 817.23<br>815.21 814.21 813.23 811.22 810.21 809.2 807.22 805.21 803.19<br>801.18 799.22 797.24 795.22 793.21 792.2 791.19 787.22 785.2<br>784.2 781.21 777.21 775.2 769.21 767.19 766.19 763.2 751.2<br>749.18 731.17 695.17 694.16 693.18 692.17 691.16 679.18 678.17<br>677.16 676.15 675.15 674.16 673.15 667.18 666.17 665.16 664.19<br>663.18 661.17 660.16 659.15 658.14 657.14 655.18 653.16 651.18<br>650.18 649.17 648.19 647.15 646.18 645.17 643.16 641.14 639.12<br>637.17 635.17 634.19 633.18 632.17 631.16 630.15 629.14 628.17<br>625.17 623.15 622.14 621.16 620.2 619.16 618.18 617.19 616.18<br>615.17 614.16 613.15 607.16 605.14 604.18 603.15 602.19 601.15<br>593.19 589.19 587.13 585.14 581.19 579.17 578.16 577.19 575.18<br>565.19 563.18 562.17 561.16 560.15 559.18 557.17 547.18 545.17<br>543.15 531.12 530.12 529.11 515.13 513.11 512.11 511.1 503.13<br>502.13 501.13 500.14 499.13 497.12 495.1 493.09 488.13 487.13<br>486.14 485.12 484.12 483.12 482.13 479.11 477.09 473.12 472.14<br>471.13 470.12 469.13 468.13 467.11 466.11 461.12 459.1 458.14<br>456.15 455.11 454.13 453.12 452.11 443.13 441.09 440.13 431.13<br>429.14 425.12 423.08 419.13 417.14 416.11 415.12 414.12 413.12<br>411.13 403.14 401.12 399.11 398.1 397.13 395.11 387.08 386.07<br>385.07 384.06 383.11 381.1 373.1 372.1 371.09 370.08 369.07<br>368.06 367.06 359.09 358.08 357.11 356.1 355.09 354.08 353.08<br>352.07 347.09 345.07 343.09 341.11 340.07 339.1 338.09 337.08<br>333.07 332.06 329.08 328.07 327.1 325.08 324.07 320.08 317.08<br>315.06 314.05 312.07 306.07 301.08 299.07 283.07 279.06 278.05<br>267.09 265.05 263.07 261.05 255.09 253.07 251.07 249.05 247.03<br>237.08 235.06 233.06 221.06 209.06 207.05 194.04 193.04 191.06<br>181.05 180.04 179.03 178.05 177.04 175.06 165.05 164.05 163.04<br>162.03 150.05 149.06 148.05 147.04 139.04 135.04 123.04 122.04<br>121.03 119.05 118.04 111.04 109.03 108.02 107.05 106.04
**References**	Heuer, S.; Richter, S.; Metzger, J.W.; Wray, V.; Nimtz, M.; Strack, D. Betacyanins from bracts of *Bougainvillea glabra*. *Phytochemistry* **1994**, 37, 761–767.<br>Slimen, I.B.; Najar, T.; Abderrabba, M. Chemical and Antioxidant Properties of Betalains. *J. Agric. Food Chem.* **2017**, 65, 675–689.<br>Kumorkiewicz-Jamro, A.; Świergosz, T.; Sutor, K.; Spórna-Kucab, A.; Wybraniec, S. Multi-colored shades of betalains: Recent advances in betacyanin chemistry. *Nat. Prod. Rep.* **2021**, 38, 2315–2346.

6’’-*O*-Rhamnosy-glabranin

**Chemical structure**	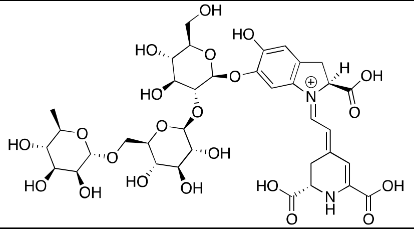
**Name:**	6’’-*O*-Rhamnosyl-glabranin
**Chemical Formula**	$C_{36}H_{46}N_2O_{22}$
**Molecular weight**	858.76
**Monoisotopic mass**	858.2542
***m/z* [M+H]**	859.2615
**Theorical fragments (*m/z*)**	859.26 858.25 857.25 856.24 843.27 842.26 841.25 840.24 839.24 831.27<br>830.26 829.25 827.27 825.26 823.24 821.22 819.27 817.25 815.27<br>814.26 813.26 812.25 811.24 809.26 807.25 805.23 801.26 799.28<br>797.26 795.25 789.26 787.24 786.23 785.26 783.25 773.26 771.25<br>769.23 753.23 751.22 713.2 712.2 711.19 697.21 695.19 694.19<br>693.18 690.22 685.21 683.19 681.21 679.2 677.18 675.17 669.21<br>667.2 666.22 665.18 664.21 663.2 661.19 659.17 655.2 651.2<br>650.21 649.19 648.21 647.21 646.2 645.19 643.2 641.18 637.19<br>634.21 633.2 632.19 625.19 623.17 621.2 620.22 619.21 618.2<br>617.21 616.2 615.19 614.18 607.18 605.21 604.2 594.2 593.21<br>589.21 587.2 581.21 579.19 578.18 577.21 575.2 565.21 563.2<br>562.19 561.18 560.17 559.2 557.19 551.15 550.14 549.14 547.2<br>545.19 535.16 533.14 532.13 531.12 523.16 521.14 520.17 519.16<br>518.15 517.15 515.13 513.11 507.16 505.15 504.15 503.13 502.16<br>501.15 500.14 499.13 497.12 493.15 489.15 488.15 487.13 486.14<br>481.15 479.13 475.13 471.17 470.14 469.13 468.13 463.13 461.12<br>459.15 447.15 445.12 443.11 441.14 435.15 433.13 432.13 431.15<br>429.14 419.15 417.14 415.12 414.12 413.14 411.13 401.14 399.13<br>389.1 388.09 387.08 386.07 375.12 374.11 373.1 372.1 371.09<br>370.08 369.07 361.1 360.1 359.12 358.11 357.11 356.1 355.09<br>354.08 353.08 351.06 349.1 347.09 345.11 343.13 342.09 341.11<br>340.1 339.1 338.09 337.08 335.09 331.09 330.08 329.11 327.1<br>326.09 325.09 324.08 323.1 319.09 317.08 316.07 315.1 314.09<br>313.08 308.09 307.08 306.07 303.1 301.08 300.07 299.07 298.06<br>297.1 287.1 285.1 283.07 281.08 280.07 279.09 273.1 271.08<br>270.07 269.1 267.09 265.08 264.07 263.07 262.06 257.1 255.09<br>253.07 252.06 251.07 249.08 247.07 245.06 239.09 237.08 235.07<br>233.06 223.07 211.07 196.06 195.05 194.04 193.04 182.08 180.04<br>179.03 178.05 177.04 165.05 164.05 163.04 162.03 149.06 148.05<br>147.04 139.04 135.04 123.04 122.04 119.05 118.04 111.04 109.03<br>108.02 107.05 106.04
**References**	Imperato, F. *Phytochemistry* **1975**, 14, 2526–2527.<br>Slimen, I.B.; Najar, T.; Abderrabba, M. Chemical and Antioxidant Properties of Betalains. *J. Agric. Food Chem.* **2017**, 65, 675–689.<br>Kumorkiewicz-Jamro, A.; Świergosz, T.; Sutor, K.; Spórna-Kucab, A.; Wybraniec, S. Multi-colored shades of betalains: Recent advances in betacyanin chemistry. *Nat. Prod. Rep.* **2021**, 38, 2315–2346.

6’’-*O*-Rhamnosy-isoglabranin

**Chemical structure**	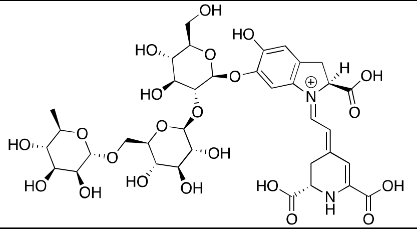
**Name:**	6’’-*O*-Rhamnosyl-isoglabranin
**Chemical Formula**	$C_{36}H_{46}N_2O_{22}$
**Molecular weight**	858.76
**Monoisotopic mass**	858.2542
***m/z* [M+H]**	859.2615
**Theorical fragments (*m/z*)**	859.26 858.25 857.25 856.24 843.27 842.26 841.25 840.24 839.24 831.27<br>830.26 829.25 827.27 825.26 823.24 821.22 819.27 817.25 815.27<br>814.26 813.26 812.25 811.24 809.26 807.25 805.23 801.26 799.28<br>797.26 795.25 789.26 787.24 786.23 785.26 783.25 773.26 771.25<br>769.23 753.23 751.22 713.2 712.2 711.19 697.21 695.19 694.19<br>693.18 690.22 685.21 683.19 681.21 679.2 677.18 675.17 669.21<br>667.2 666.22 665.18 664.21 663.2 661.19 659.17 655.2 651.2<br>650.21 649.19 648.21 647.21 646.2 645.19 643.2 641.18 637.19<br>634.21 633.2 632.19 625.19 623.17 621.2 620.22 619.21 618.2<br>617.21 616.2 615.19 614.18 607.18 605.21 604.2 594.2 593.21<br>589.21 587.2 581.21 579.19 578.18 577.21 575.2 565.21 563.2<br>562.19 561.18 560.17 559.2 557.19 551.15 550.14 549.14 547.2<br>545.19 535.16 533.14 532.13 531.12 523.16 521.14 520.17 519.16<br>518.15 517.15 515.13 513.11 507.16 505.15 504.15 503.13 502.16<br>501.15 500.14 499.13 497.12 493.15 489.15 488.15 487.13 486.14<br>481.15 479.13 475.13 471.17 470.14 469.13 468.13 463.13 461.12<br>459.15 447.15 445.12 443.11 441.14 435.15 433.13 432.13 431.15<br>429.14 419.15 417.14 415.12 414.12 413.14 411.13 401.14 399.13<br>389.1 388.09 387.08 386.07 375.12 374.11 373.1 372.1 371.09<br>370.08 369.07 361.1 360.1 359.12 358.11 357.11 356.1 355.09<br>354.08 353.08 351.06 349.1 347.09 345.11 343.13 342.09 341.11<br>340.1 339.1 338.09 337.08 335.09 331.09 330.08 329.11 327.1<br>326.09 325.09 324.08 323.1 319.09 317.08 316.07 315.1 314.09<br>313.08 308.09 307.08 306.07 303.1 301.08 300.07 299.07 298.06<br>297.1 287.1 285.1 283.07 281.08 280.07 279.09 273.1 271.08<br>270.07 269.1 267.09 265.08 264.07 263.07 262.06 257.1 255.09<br>253.07 252.06 251.07 249.08 247.07 245.06 239.09 237.08 235.07<br>233.06 223.07 211.07 196.06 195.05 194.04 193.04 182.08 180.04<br>179.03 178.05 177.04 165.05 164.05 163.04 162.03 149.06 148.05<br>147.04 139.04 135.04 123.04 122.04 119.05 118.04 111.04 109.03<br>108.02 107.05 106.04
**References**	Imperato, F. *Phytochemistry* **1975**, 14, 2526–2527.<br>Slimen, I.B.; Najar, T.; Abderrabba, M. Chemical and Antioxidant Properties of Betalains. *J. Agric. Food Chem.* **2017**, 65, 675–689.<br>Kumorkiewicz-Jamro, A.; Świergosz, T.; Sutor, K.; Spórna-Kucab, A.; Wybraniec, S. Multi-colored shades of betalains: Recent advances in betacyanin chemistry. *Nat. Prod. Rep.* **2021**, 38, 2315–2346.

Cafglabranin

**Chemical structure**	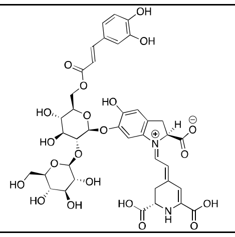
**Name:**	Cafglabranin (6’-*O*-*E*-Caffeoyl-glabranin)
**Chemical Formula**	$C_{39}H_{42}N_2O_{21}$
**Molecular weight**	874.76
**Monoisotopic mass**	874.2280
***m/z* [M+H]**	875.2353
**Theorical fragments (*m/z*)**	875.24 874.23 873.22 872.21 859.24 858.23 857.22 856.22 855.21 847.24 846.23 845.22 843.25 842.24 841.23 840.22 839.21 838.21 837.2 835.24 833.22 831.25 830.24 829.23 828.22 827.21 825.23 823.22 821.2 819.19 817.23 815.25 813.23 812.23 811.22 810.21 809.2 805.23 803.21 802.21 801.23 799.22 795.22 793.21 789.23 787.22 786.21 785.2 784.2 783.22 781.21 771.22 769.21 767.19 751.2 749.18 713.18 712.17 711.17 706.2 697.19 696.18 695.17 694.16 693.16 685.19 684.18 683.17 682.2 681.19 680.18 679.17 678.17 677.16 676.15 675.15 673.19 671.17 669.19 667.18 666.18 665.16 664.19 663.18 662.17 661.17 659.15 657.14 655.18 653.16 651.18 650.18 649.18 648.17 647.15 643.18 641.16 640.15 639.18 637.17 636.19 635.18 634.18 633.18 632.17 631.17 630.16 627.18 625.17 623.15 622.14 621.18 620.17 619.16 610.18 609.18 607.16 605.14 603.17 597.18 595.17 594.16 593.19 591.17 589.15 587.13 581.19 579.17 578.16 577.16 576.15 575.18 573.16 563.18 561.16 559.14 533.14 532.13 531.12 520.14 518.13 517.15 515.13 514.12 513.11 505.15 504.13 503.13 502.13 500.12 499.13 497.12 495.1 488.13 487.12 486.12 485.12 481.12 479.11 475.13 470.12 469.11 468.11 463.13 461.12 459.13 457.12 447.13 445.12 443.11 441.12 435.13 433.11 432.11 431.13 429.12 427.11 425.1 419.13 417.12 415.1 414.09 413.12 411.11 401.12 399.11 397.09 389.1 388.09 387.08 386.07 375.12 374.11 373.1 372.1 371.09 370.08 369.07 361.1 360.1 359.12 358.12 357.11 356.1 355.09 354.08 353.08 351.06 349.1 347.09 345.11 343.13 342.08 341.11 339.1 338.09 337.08 336.07 335.09 331.09 330.08 329.11 327.1 326.09 325.12 324.08 323.1 319.09 317.08 316.07 315.1 314.09 313.08 312.07 310.09 308.08 306.07 303.1 301.08 300.07 299.07 298.06 296.08 287.1 285.09 283.07 281.08 280.07 267.06 265.08 264.07 263.07 262.06 255.09 253.07 251.07 249.08 247.07 245.06 237.08 235.07 233.06 223.07 211.07 196.06 195.05 194.04 193.04 182.08 180.04 179.03 178.05 177.04 165.05 164.05 163.04 162.03 149.06 148.05 147.04 139.04 135.04 123.04 122.04 119.05 118.04 111.04 109.03 108.02 107.05 106.04
**References**	Heuer, S.; Richter, S.; Metzger, J.W.; Wray, V.; Nimtz, M.; Strack, D. Betacyanins from bracts of *Bougainvillea glabra*. *Phytochemistry* **1994**, 37, 761–767.<br>Slimen, I.B.; Najar, T.; Abderrabba, M. Chemical and Antioxidant Properties of Betalains. *J. Agric. Food Chem.* **2017**, 65, 675–689.<br>Kumorkiewicz-Jamro, A.; Świergosz, T.; Sutor, K.; Spórna-Kucab, A.; Wybraniec, S. Multi-colored shades of betalains: Recent advances in betacyanin chemistry. *Nat. Prod. Rep.* **2021**, 38, 2315–2346.

Isocafglabranin

**Chemical structure**	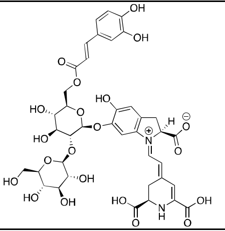
**Name:**	Isocafglabranin (6’-*O*-*E*-Caffeoyl-isoglabranin)
**Chemical Formula**	$C_{39}H_{42}N_2O_{21}$
**Molecular weight**	874.76
**Monoisotopic mass**	874.2280
***m/z* [M+H]**	875.2353
**Theorical fragments (*m/z*)**	875.24 874.23 873.22 872.21 859.24 858.23 857.22 856.22 855.21 847.24<br>846.23 845.22 843.25 842.24 841.23 840.22 839.21 838.21 837.2<br>835.24 833.22 831.25 830.24 829.23 828.22 827.21 825.23 823.22<br>821.2 819.19 817.23 815.25 813.23 812.23 811.22 810.21 809.2<br>805.23 803.21 802.21 801.23 799.22 795.22 793.21 789.23 787.22<br>786.21 785.2 784.2 783.22 781.21 771.22 769.21 767.19 751.2<br>749.18 713.18 712.17 711.17 706.2 697.19 696.18 695.17 694.16<br>693.16 685.19 684.18 683.17 682.2 681.19 680.18 679.17 678.17<br>677.16 676.15 675.15 673.19 671.17 669.19 667.18 666.18 665.16<br>664.19 663.18 662.17 661.17 659.15 657.14 655.18 653.16 651.18<br>650.18 649.18 648.17 647.15 643.18 641.16 640.15 639.18 637.17<br>636.19 635.18 634.18 633.18 632.17 631.17 630.16 627.18 625.17<br>623.15 622.14 621.18 620.17 619.16 610.18 609.18 607.16 605.14<br>603.17 597.18 595.17 594.16 593.19 591.17 589.15 587.13 581.19<br>579.17 578.16 577.16 576.15 575.18 573.16 563.18 561.16 559.14<br>533.14 532.13 531.12 520.14 518.13 517.15 515.13 514.12 513.11<br>505.15 504.13 503.13 502.13 500.12 499.13 497.12 495.1 488.13<br>487.12 486.12 485.12 481.12 479.11 475.13 470.12 469.11 468.11<br>463.13 461.12 459.13 457.12 447.13 445.12 443.11 441.12 435.13<br>433.11 432.11 431.13 429.12 427.11 425.1 419.13 417.12 415.1<br>414.09 413.12 411.11 401.12 399.11 397.09 389.1 388.09 387.08<br>386.07 375.12 374.11 373.1 372.1 371.09 370.08 369.07 361.1<br>360.1 359.12 358.12 357.11 356.1 355.09 354.08 353.08 351.06<br>349.1 347.09 345.11 343.13 342.08 341.11 339.1 338.09 337.08<br>336.07 335.09 331.09 330.08 329.11 327.1 326.09 325.12 324.08<br>323.1 319.09 317.08 316.07 315.1 314.09 313.08 312.07 310.09<br>308.08 306.07 303.1 301.08 300.07 299.07 298.06 296.08 287.1<br>285.09 283.07 281.08 280.07 267.06 265.08 264.07 263.07 262.06<br>255.09 253.07 251.07 249.08 247.07 245.06 237.08 235.07 233.06<br>223.07 211.07 196.06 195.05 194.04 193.04 182.08 180.04 179.03<br>178.05 177.04 165.05 164.05 163.04 162.03 149.06 148.05 147.04<br>139.04 135.04 123.04 122.04 119.05 118.04 111.04 109.03 108.02<br>107.05 106.04
**References**	Heuer, S.; Richter, S.; Metzger, J.W.; Wray, V.; Nimtz, M.; Strack, D. Betacyanins from bracts of *Bougainvillea glabra*. *Phytochemistry* **1994**, 37, 761–767.<br>Slimen, I.B.; Najar, T.; Abderrabba, M. Chemical and Antioxidant Properties of Betalains. *J. Agric. Food Chem.* **2017**, 65, 675–689.<br>Kumorkiewicz-Jamro, A.; Świergosz, T.; Sutor, K.; Spórna-Kucab, A.; Wybraniec, S. Multi-colored shades of betalains: Recent advances in betacyanin chemistry. *Nat. Prod. Rep.* **2021**, 38, 2315–2346.

Bicoumglabranin

**Chemical structure**	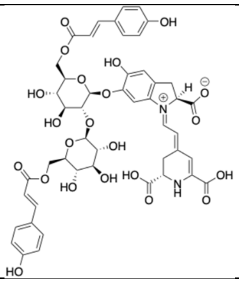
**Name:**	Bicoumglabranin (6’.6’’-Di-*O*-*E*-4-coumaroyl-glabranin)
**Chemical Formula**	$C_{48}H_{48}N_2O_{22}$
**Molecular weight**	1004.90
**Monoisotopic mass**	1004.2699
***m/z* [M+H]**	1005.2772
**Theorical fragments (*m/z*)**	1005.28 1004.27 1003.26 1002.25 989.28 988.27 987.27 986.26 985.25 977.28<br>976.27 975.27 973.29 972.28 971.27 970.26 969.26 968.25 967.24<br>965.28 963.27 961.29 960.28 959.27 958.26 957.26 955.28 953.26<br>951.25 949.23 947.27 945.29 943.28 942.27 941.26 940.25 939.25<br>935.27 933.26 932.25 931.28 929.26 925.27 923.25 919.28 917.26<br>916.25 915.25 914.24 913.27 911.25 901.27 899.25 897.23 881.24<br>879.22 841.23 840.22 839.21 836.24 825.23 824.23 823.22 822.21<br>821.2 813.23 812.24 811.22 810.22 809.22 807.22 805.21 803.19<br>797.24 796.22 795.22 794.23 793.22 792.21 791.21 789.21 787.2<br>783.22 780.23 779.22 778.21 777.21 771.22 769.21 767.22 766.23<br>765.23 764.22 763.22 762.22 761.21 760.2 755.23 753.21 751.22<br>750.22 740.22 739.22 735.23 733.21 727.22 725.21 724.2 723.23<br>721.21 711.23 709.21 708.2 707.2 706.19 705.22 703.2 697.19<br>696.18 695.17 693.22 691.2 689.19 681.19 680.18 679.18 678.17<br>677.16 675.17 669.19 668.18 667.18 665.2 663.18 662.17 661.17<br>660.16 659.15 657.19 655.18 653.2 651.18 650.17 649.17 648.19<br>647.19 646.18 645.17 643.16 641.14 639.18 637.17 635.19 633.17<br>632.17 631.16 627.18 625.17 624.16 623.19 621.17 617.19 616.18<br>615.17 614.16 611.19 609.17 607.16 606.15 605.16 603.16 593.18<br>591.16 589.15 587.18 575.18 573.15 571.13 563.18 561.16 560.15<br>559.18 557.17 547.18 545.17 543.15 533.14 532.13 531.12 517.15<br>515.13 514.12 513.11 505.15 504.15 503.13 502.13 501.13 499.13<br>497.12 495.1 488.13 487.13 486.14 485.12 484.12 483.12 481.12<br>479.11 475.13 472.14 471.13 470.12 469.12 468.13 463.13 461.12<br>457.12 454.13 453.12 452.11 445.12 443.11 431.13 427.11 425.12<br>419.13 417.12 416.11 415.14 413.12 411.13 403.14 401.12 399.11<br>398.1 397.13 395.11 389.1 388.09 387.08 386.07 385.13 383.11<br>381.1 375.12 374.11 373.1 372.1 371.09 370.08 369.07 361.1<br>360.1 359.12 358.12 357.11 356.1 355.09 354.08 353.08 351.06<br>349.1 347.09 345.11 343.13 342.08 341.11 339.1 338.09 337.08<br>336.07 335.09 331.09 330.08 329.11 327.1 326.09 325.12 324.08<br>323.1 319.09 317.08 316.07 315.1 314.09 313.08 312.07 310.09<br>308.08 306.07 303.1 301.08 300.07 299.07 298.06 296.08 287.1<br>285.09 283.07 281.08 280.07 267.06 265.08 264.07 263.07 262.06<br>255.09 253.07 251.07 249.08 247.07 245.06 237.08 235.07 233.06<br>223.07 211.07 196.06 195.05 194.04 193.04 182.08 180.04 179.03<br>178.05 177.04 165.05 164.05 163.04 162.03 149.06 148.05 147.04<br>139.04 135.04 123.04 122.04 119.05 118.04 111.04 109.03 108.02<br>107.05 106.04
**References**	Kumorkiewicz-Jamro, A.; Świergosz, T.; Sutor, K.; Spórna-Kucab, A.; Wybraniec, S. Multi-colored shades of betalains: Recent advances in betacyanin chemistry. *Nat. Prod. Rep.* **2021**, 38, 2315–2346.

2’’-*O*-[(6´´-*O*-*E*-4-coumaroyl)]-glucosyl-cafglabranin

**Chemical structure**	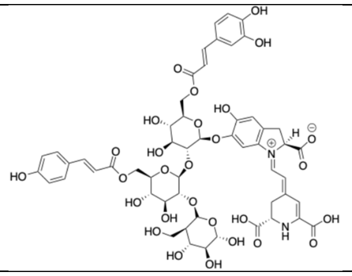
**Name:**	2’’-*O*-[(6´´-*O*-*E*-4-coumaroyl)]-glucosyl-cafglabranin
**Chemical Formula**	$C_{54}H_{58}N_2O_{28}$
**Molecular weight**	1183.04
**Monoisotopic mass**	1182.3176
***m/z* [M+H]**	1183.3289
**Theorical fragments (*m/z*)**	1183.32 1182.32 1181.31 1180.3 1167.33 1166.32 1165.31 1164.31 1163.3 1155.33<br>1154.32 1153.31 1151.34 1149.32 1147.3 1145.29 1143.33 1141.31 1139.34<br>1138.33 1137.32 1136.31 1135.3 1133.32 1131.31 1129.29 1125.32 1123.34<br>1121.32 1119.31 1113.32 1111.3 1110.3 1109.32 1107.31 1097.32 1095.31<br>1093.29 1077.3 1075.28 1021.27 1020.26 1019.28 1018.27 1017.26 1014.29<br>1005.28 1003.28 1002.27 1001.27 999.25 993.28 991.26 990.29 989.27<br>988.27 987.26 985.27 983.26 977.28 975.29 974.27 973.27 972.28<br>971.29 970.26 969.28 967.24 963.27 961.27 959.29 958.27 957.27<br>956.26 951.27 949.25 947.26 945.28 944.28 943.27 942.27 941.27<br>940.26 939.26 938.25 933.26 931.26 929.27 928.26 918.27 917.27<br>915.27 913.25 911.26 905.27 903.26 902.25 901.28 899.26 889.28<br>887.26 886.25 885.24 884.24 883.27 881.25 871.27 869.25 857.22<br>855.21 841.23 839.24 837.22 828.23 826.22 825.23 824.22 823.22<br>821.22 812.22 810.22 808.23 807.22 805.21 796.22 795.21 794.23<br>793.22 792.21 785.2 778.23 777.22 776.22 769.21 767.21 765.22<br>755.22 753.22 751.2 749.23 743.22 741.22 740.19 739.21 738.2<br>737.23 735.21 727.22 725.23 723.21 722.21 721.23 719.22 713.18<br>712.17 711.17 709.23 707.22 697.19 695.17 694.16 693.16 685.19<br>683.17 681.19 679.18 677.16 675.15 669.19 667.18 665.16 663.18<br>661.17 659.15 655.18 651.18 649.17 648.17 643.18 641.16 637.17<br>632.17 630.18 625.17 623.15 614.16 607.16 605.14 591.17 579.17<br>575.18 573.18 563.18 561.18 557.17 545.17 533.14 531.12 520.14<br>518.13 517.15 515.13 513.11 504.13 502.13 500.12 499.13 497.12<br>488.13 487.12 486.12 470.12 469.11 468.11 461.12 459.13 447.13<br>443.11 441.12 435.13 433.11 432.11 431.13 429.12 419.13 417.12<br>415.1 414.09 413.12 411.11 401.12 399.11 389.1 388.09 387.08<br>386.07 375.12 374.11 373.1 372.1 371.09 370.08 369.07 361.1<br>360.1 359.12 358.12 357.11 356.1 355.09 354.08 353.08 351.06<br>349.1 347.09 345.11 343.13 342.08 341.11 339.1 337.08 335.09<br>331.09 330.08 329.11 327.1 326.09 325.12 324.08 323.1 319.09<br>317.08 316.07 315.1 314.09 313.08 306.07 303.1 301.08 300.07<br>299.07 298.06 287.1 285.09 283.07 281.08 280.07 267.06 265.08<br>264.07 263.07 262.06 255.09 251.07 249.08 247.07 245.06 237.08<br>235.07 233.06 223.07 211.07 196.06 195.05 194.04 193.04 182.08<br>180.04 179.03 178.05 177.04 165.05 164.05 163.04 162.03 149.06<br>148.05 147.04 139.04 135.04 123.04 122.04 119.05 118.04 111.04<br>109.03 108.02 107.05 106.04
**References**	Kumorkiewicz-Jamro, A.; Świergosz, T.; Sutor, K.; Spórna-Kucab, A.; Wybraniec, S. Multi-colored shades of betalains: Recent advances in betacyanin chemistry. *Nat. Prod. Rep.* **2021**, 38, 2315–2346.

2’’-*O*-glucosyl-bicoumglabranin

**Chemical structure**	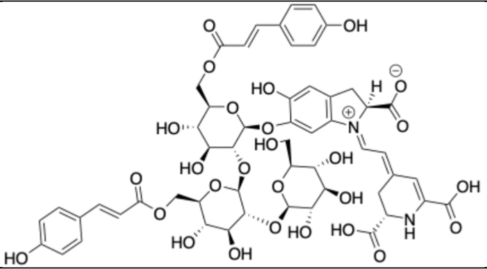
**Name:**	2’’-*O*-glucosyl-bicoumglabranin
**Chemical Formula**	$C_{54}H_{58}N_2O_{27}$
**Molecular weight**	1167.04
**Monoisotopic mass**	1166.3227
***m/z* [M+H]**	1167.3300
**Theorical fragments (*m/z*)**	1167.33 1166.32 1165.31 1164.31 1151.34 1150.33 1149.32 1148.31 1147.3 1139.34 1138.33 1137.32 1135.34 1133.32 1131.31 1129.29 1127.34 1125.32 1123.34 1122.33 1121.32 1120.32 1119.31 1117.33 1115.31 1113.3 1109.32 1107.35 1105.33 1103.31 1097.32 1095.31 1094.3 1093.33 1091.31 1081.33 1079.31 1077.3 1061.3 1059.29 1005.28 1004.27 1003.28 1002.27 1001.27 998.29 989.28 987.27 986.26 985.27 983.26 977.28 975.29 974.29 973.27 972.28 971.27 969.26 967.26 961.29 959.27 958.27 957.28 956.28 955.28 954.27 953.26 951.25 947.27 945.28 943.28 942.28 941.27 940.26 935.27 933.26 931.26 929.26 928.29 927.28 926.27 925.28 924.27 923.26 922.25 917.26 915.25 913.28 912.27 902.27 901.28 899.25 897.23 895.27 889.28 887.26 886.25 885.28 883.27 873.28 871.27 870.26 869.25 868.24 867.27 865.25 855.27 853.25 841.23 839.24 837.22 825.23 823.22 821.22 812.24 810.22 809.22 808.23 807.22 805.21 796.22 794.23 792.21 791.21 780.23 779.22 778.21 777.22 776.22 769.21 767.21 762.22 761.21 760.2 751.2 749.23 739.22 737.23 733.21 727.22 725.23 724.2 723.21 722.21 721.21 719.22 711.23 709.21 707.2 706.19 705.22 703.2 697.19 696.18 695.17 693.22 691.2 681.19 679.18 678.17 677.16 669.19 667.18 665.2 663.18 661.17 659.15 653.2 651.18 649.17 647.19 645.17 643.16 639.18 635.19 633.17 632.17 630.18 627.18 625.17 621.17 614.16 609.17 607.16 591.16 589.15 575.18 573.18 563.18 561.18 557.17 545.17 533.14 531.12 517.15 515.13 513.11 504.15 502.13 501.13 499.13 497.12 488.13 486.14 484.12 483.12 472.14 471.13 470.12 461.12 454.13 453.12 452.11 443.11 431.13 425.12 419.13 417.12 416.11 415.14 413.12 403.14 401.12 399.11 398.1 397.13 395.11 389.1 388.09 387.08 386.07 385.13 383.11 375.12 374.11 373.1 372.1 371.09 370.08 369.07 361.1 360.1 359.12 358.12 357.11 356.1 355.09 354.08 353.08 351.06 349.1 347.09 345.11 343.13 342.08 341.11 339.1 337.08 335.09 331.09 330.08 329.11 327.1 326.09 325.12 324.08 323.1 319.09 317.08 316.07 315.1 314.09 313.08 306.07 303.1 301.08 300.07 299.07 298.06 287.1 285.09 283.07 281.08 280.07 267.06 265.08 264.07 263.07 262.06 255.09 251.07 249.08 247.07 245.06 237.08 235.07 233.06 223.07 211.07 196.06 195.05 194.04 193.04 182.08 180.04 179.03 178.05 177.04 165.05 164.05 163.04 162.03 149.06 148.05 147.04 139.04 135.04 123.04 122.04 119.05 118.04 111.04 109.03 108.02 107.05 106.04 98.02
**References**	Kumorkiewicz-Jamro, A.; Świergosz, T.; Sutor, K.; Spórna-Kucab, A.; Wybraniec, S. Multi-colored shades of betalains: Recent advances in betacyanin chemistry. *Nat. Prod. Rep.* **2021**, 38, 2315–2346.

2’’-*O*-[(6’’-*O*-*E*-4-coumaroyl)]-sophorosyl-cafglabranin

**Chemical structure**	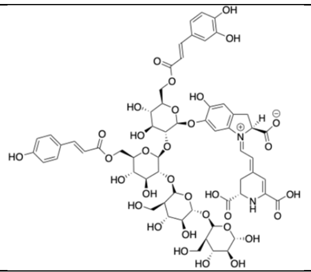
**Name:**	2’’-*O*-[(6’’-*O*-*E*-4-coumaroyl)]-sophorosyl-cafglabranin
**Chemical Formula**	$C_{60}H_{68}N_2O_{33}$
**Molecular weight**	1345.19
**Monoisotopic mass**	1344.3704
***m/z* [M+H]**	1345.3777
**Theorical fragments (*m/z*)**	1345.38 1344.37 1343.36 1329.38 1328.37 1327.37 1326.36 1325.35 1317.38 1316.37 1315.37 1313.39 1311.37 1309.36 1307.34 1305.38 1303.37 1301.39 1300.38 1299.37 1298.36 1297.36 1295.38 1293.36 1291.35 1287.37 1285.39 1283.38 1281.36 1275.37 1273.36 1272.35 1271.38 1269.36 1259.38 1257.36 1255.35 1239.35 1237.34 1183.32 1182.32 1181.33 1180.32 1179.31 1176.34 1167.33 1165.34 1164.33 1163.32 1161.3 1155.33 1153.34 1152.34 1151.32 1150.32 1149.32 1147.32 1145.31 1139.34 1137.34 1136.32 1135.32 1134.33 1133.35 1132.31 1131.33 1129.29 1125.32 1123.32 1121.35 1120.33 1119.32 1118.31 1113.32 1111.3 1109.31 1107.33 1106.33 1105.33 1104.32 1095.31 1093.31 1091.32 1080.32 1079.32 1077.32 1075.3 1067.32 1065.31 1064.3 1063.33 1061.31 1051.33 1049.31 1047.3 1021.27 1020.26 1019.26 1017.26 1005.28 1003.26 1002.25 1001.29 999.27 993.28 991.26 990.29 989.28 988.27 987.27 986.28 985.25 983.28 977.28 975.27 974.27 973.25 972.27 971.27 970.28 969.26 967.24 963.27 959.27 957.26 956.28 951.27 949.25 947.26 945.26 933.26 931.24 929.27 917.27 915.28 913.23 905.27 903.28 901.26 899.28 887.28 885.27 857.22 855.21 841.23 839.21 837.2 828.23 826.22 825.23 823.22 821.2 812.22 810.22 808.21 807.22 805.21 794.21 785.2 769.21 767.19 755.22 751.2 743.22 741.2 737.21 725.21 723.19 713.18 712.17 711.17 697.19 695.17 694.16 693.16 685.19 683.17 681.19 679.18 677.16 675.15 669.19 667.18 665.16 663.18 661.17 659.15 655.18 651.18 649.17 643.18 641.16 637.17 625.17 623.15 607.16 605.14 533.14 531.12 520.14 518.13 517.15 515.13 513.11 504.13 502.13 500.12 499.13 497.12 486.12 469.11 468.11 461.12 447.13 443.11 441.12 435.13 433.11 429.12 417.12 415.1 414.09 411.11 399.11 389.1 388.09 387.08 375.12 374.11 373.1 372.1 371.09 370.08 369.07 361.1 359.12 358.12 357.11 356.1 355.09 354.08 353.08 345.11 343.13 341.11 339.1 337.08 331.09 330.08 329.11 327.1 326.09 319.09 317.08 316.07 314.09 306.07 303.1 301.08 300.07 299.07 298.06 287.1 285.09 283.07 281.08 280.07 267.06 265.08 263.07 251.07 249.08 237.08 235.07 233.06 196.06 195.05 194.04 193.04 182.08 180.04 179.03 178.05 177.04 165.05 164.05 163.04 162.03 149.06 148.05 147.04 139.04 135.04 123.04 122.04 119.05 111.04 109.03 108.02 107.05 106.04
**References**	Kumorkiewicz-Jamro, A.; Świergosz, T.; Sutor, K.; Spórna-Kucab, A.; Wybraniec, S. Multi-colored shades of betalains: Recent advances in betacyanin chemistry. *Nat. Prod. Rep.* **2021**, 38, 2315–2346.

Betaxanthins

Positively charged-type

Histamine-bx

Chemical structure	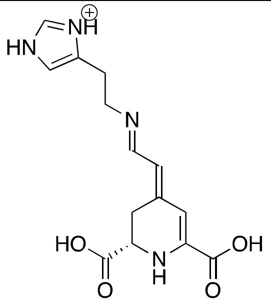
**Name:**	Histamine-bx
**Chemical Formula**	$C_{14}H_{16}N_4O_4$
**Molecular weight**	304.31
**Monoisotopic mass**	304.1172
***m/z* [M+H]**	305.1244
**Theorical fragments (*m/z*)**	305.12 304.12 303.11 302.1 301.09 290.11 289.13 288.12 287.11 286.11<br>285.1 284.09 279.12 278.11 277.13 276.12 275.11 274.12 273.13<br>272.1 271.12 270.09 269.1 268.1 266.11 265.13 264.1 263.11<br>262.12 261.13 260.13 259.12 258.09 257.1 251.11 249.13 248.13<br>247.12 246.11 245.14 244.11 243.12 242.12 241.11 239.09 237.09<br>236.08 235.12 234.11 233.1 232.11 231.09 230.09 229.06 228.11<br>227.09 224.08 223.07 222.06 221.09 220.11 219.08 218.09 217.14<br>216.14 215.13 214.1 213.09 212.08 211.07 210.06 209.06 208.08<br>207.08 206.07 205.06 204.11 203.13 202.1 201.11 200.08 199.1<br>198.09 197.09 196.06 195.08 194.04 193.06 192.05 191.05 190.04<br>189.11 188.12 187.1 186.1 184.06 183.05 182.04 181.06 180.07<br>179.08 178.05 177.07 176.03 175.05 174.1 170.04 169.05 168.03<br>167.08 166.07 165.07 164.03 163.05 162.1 161.09 160.09 158.04<br>157.04 156.03 154.05 153.07 152.07 151.09 150.1 149.07 148.09<br>147.08 146.07 145.04 144.03 143.02 142.05 141.07 140.03 139.05<br>138.05 137.03 136.09 135.08 134.06 133.08 132.03 130.05 129.04<br>128.03 126.02 125.05 124.09 123.08 122.07 121.06 120.06 119.06<br>116.03 115.03 114.05 113.05 112.09 111.03 110.07 109.06 108.06<br>107.05 106.07 105.03 102.02 100
**References**	F. Kugler, F. C. Stintzing, R. Carle, *J. Agric. Food Chem.* **2004**, *52*, 2975–2981.<br>M. Piattelli, L. Minale, R. A. Nicolaus, *Phytochemistry* **1965**, *4*, 817–823.<br>I. Belhadj Slimen, T. Najar, M. Abderrabba, *J. Agric. Food Chem.* **2017**, *65*, 675–689.<br>L. C. Esteves, C. O. Machado, L. C. P. Gonçalves, V. F. Cavalcante, G. Obeid, T. C. Correra, E. L. Bastos, *Antioxidants* **2022**, *11*, 2259.

Histidine-bx

**Chemical structure**	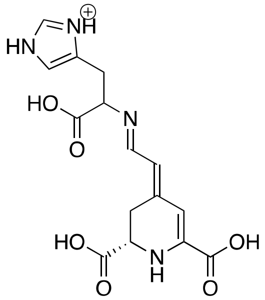
**Name:**	Histidine-bx (Muscaaurin VII)
**Chemical Formula**	$C_{15}H_{16}N_4O_6$
**Molecular weight**	348.32
**Monoisotopic mass**	348.1070
***m/z* [M+H]**	349.1143
**Theorical fragments (*m/z*)**	349.11 348.11 347.1 346.09 345.08 334.1 333.12 332.11 331.1 330.1<br>329.09 322.1 321.12 320.11 319.1 318.11 317.12 316.09 315.11<br>314.1 313.09 312.09 311.08 309.12 307.1 306.11 305.12 304.12<br>303.11 302.1 301.09 300.1 299.11 298.08 297.1 295.08 293.12<br>292.12 291.11 290.1 289.13 288.12 287.11 286.11 285.1 284.07<br>283.08 282.08 281.08 280.07 279.11 278.1 277.09 276.1 275.11<br>274.11 273.13 272.09 271.12 269.1 268.07 267.06 266.09 265.08<br>264.07 263.07 262.08 261.1 260.1 259.08 258.09 257.07 253.08<br>252.07 251.07 250.06 249.05 248.08 247.07 246.09 245.06 244.07<br>243.09 242.09 241.11 240.08 239.07 238.09 237.09 236.08 235.07<br>234.06 233.06 232.05 231.04 230.09 225.09 224.08 223.07 222.06<br>221.09 219.08 218.09 217.06 215.09 213.09 212.08 211.07 210.06<br>209.06 208.05 207.08 206.09 205.06 204.08 197.09 196.06 195.08<br>194.04 193.06 192.05 191.07 190.06 189.09 188.08 187.07 186.07<br>184.06 183.05 182.04 181.06 180.07 179.07 178.05 177.07 176.03<br>175.05 174.07 173.06 172.05 170.04 169.05 168.03 167.07 166.06<br>165.07 164.05 163.05 162.07 158.04 157.04 156.08 155.07 154.06<br>153.05 152.07 151.07 150.07 149.06 148.05 147.06 144.03 142.05<br>141.07 140.03 139.05 138.04 137.03 136.05 134.07 132.03 130.05<br>129.04 128.08 126.07 125.05 124.04 123.06 122.05 121.04 120.04<br>119.02 118.04 116.03 115.03 114.05 113.05 112.09 111.06 110.07<br>109.04 108.06 107.05 106.07 105.03
	Musso, H. Pigments of fly agaric, *Amanita muscaria*. *Tetrahedron* **1979**, 35, 2843–2853.<br>Kugler, F.; Stintzing, F.C.; Carle, R. Identification of betalains from petioles of differently colored Swiss chard (*Beta vulgaris* L. ssp. *cicla* Alef. Cv. Bright Lights) by high-performance liquid chromatography-electrospray ionization mass spectrometry. *J. Agric. Food Chem.* **2004**, 52, 2975–2981.<br>Slimen, I.B.; Najar, T.; Abderrabba, M. Chemical and Antioxidant Properties of Betalains. *J. Agric. Food Chem.* **2017**, 65, 675–689.<br>Esteves, L.C.; Machado, C.O.; Gonçalves, L.C.P.; Cavalcante, V.F.; Obeid, G.; Correra, T.C.; Bastos, E.L. Structural Effects on the Antioxidant Properties of Amino Acid Betaxanthins. *Antioxidants* **2022**, 11, 2259.

Arginine-bx

**Chemical structure**	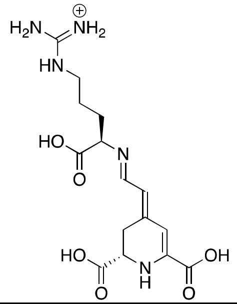
**Name:**	Arginine-bx
**Chemical Formula**	$C_{15}H_{21}N_5O_6$
**Molecular weight**	367.36
**Monoisotopic mass**	367.1492
***m/z* [M+H]**	368.1565
**Theorical fragments (*m/z*)**	368.16 367.15 366.14 365.13 364.13 353.15 352.16 351.13 350.15 349.14<br>348.13 347.12 346.11 340.16 339.15 338.15 337.14 336.17 335.12<br>334.1 333.14 332.14 331.13 330.12 328.16 326.13 325.13 324.17<br>323.16 322.15 321.14 320.14 319.13 318.16 317.12 316.14 315.11<br>314.12 312.17 311.16 310.15 309.11 308.1 307.09 306.16 305.08<br>304.14 303.13 302.12 298.15 297.14 296.14 295.09 294.16 293.11<br>292.11 291.1 290.09 289.08 288.15 287.07 283.09 282.08 281.08<br>280.13 279.12 278.12 277.11 276.11 275.1 274.09 273.09 272.08<br>271.07 270.08 269.08 268.07 267.06 266.16 265.12 264.11 263.1<br>262.06 261.09 260.08 259.11 257.09 255.1 253.08 252.05 251.07<br>250.06 249.05 248.08 247.11 246.1 245.09 241.08 239.07 238.08<br>237.09 236.09 235.07 234.06 233.06 232.05 231.11 229.1 225.09<br>224.08 223.07 222.06 221.09 220.07 219.08 218.08 217.06 213.09<br>212.08 211.07 210.06 209.1 208.05 207.08 206.07 205.06 204.05<br>199.12 198.11 197.09 196.06 195.08 194.04 193.06 192.05 191.09<br>190.04 187.12 186.11 185.1 184.06 183.05 182.04 181.11 180.07<br>179.08 178.05 177.07 176.03 175.12 174.11 173.1 172.1 171.09<br>170.04 169.11 168.1 167.08 166.07 165.07 164.03 163.05 160.11<br>159.12 158.09 157.08 156.08 155.09 154.05 153.11 152.07 151.09<br>150.05 149.07 147.12 145.11 144.09 143.08 142.1 141.07 140.08<br>139.06 138.07 137.08 136.08 134.06 132.04 131.08 130.1 129.11<br>128.08 127.1 126.05 125.05 124.09 123.07 122.06 121.05 120.04<br>118.1 116.07 115.03 114.1 113.05 112.09 111.08 110.07 109.05<br>108.04 105.03
	Kugler, F.; Graneis, S.; Stintzing, F.C.; Carle, R. Studies on betaxanthin profiles of vegetables and fruits from the Chenopodi-aceae and Cactaceae. *Z. Fur. Naturforschung Sect. C J. Biosci.* **2007**, 62, 311–318.<br>Slimen, I.B.; Najar, T.; Abderrabba, M. Chemical and Antioxidant Properties of Betalains. *J. Agric. Food Chem.* **2017**, 65, 675–689.<br>Esteves, L.C.; Machado, C.O.; Gonçalves, L.C.P.; Cavalcante, V.F.; Obeid, G.; Correra, T.C.; Bastos, E.L. Structural Effects on the Antioxidant Properties of Amino Acid Betaxanthins. *Antioxidants* **2022**, 11, 2259.

Lysine-bx

**Chemical structure**	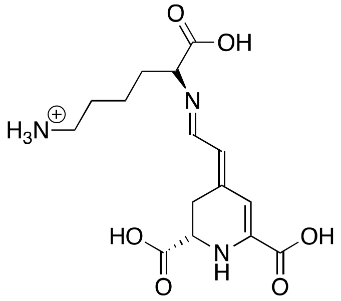
**Name:**	Lysine-bx
**Chemical Formula**	$C_{15}H_{21}N_3O_6$
**Molecular weight**	339.35
**Monoisotopic mass**	339.1430
***m/z* [M+H]**	340.1503
**Theorical fragments (*m/z*)**	340.15 339.14 338.13 337.13 336.12 325.14 324.16 323.12 322.14 321.11<br>320.12 319.09 318.11 312.16 311.15 310.14 309.11 308.16 307.13<br>306.14 305.11 304.13 303.1 302.11 301.08 300.16 298.14 297.11<br>296.16 295.09 294.14 293.14 292.13 291.13 290.15 289.12 288.13<br>287.1 286.12 285.09 284.16 283.09 282.14 281.08 280.17 279.13<br>278.15 277.12 276.13 275.1 274.12 273.12 271.11 270.08 269.08<br>268.13 267.06 266.15 265.12 264.17 263.07 262.16 261.12 260.14<br>259.11 257.09 255.1 254.09 253.08 252.13 251.07 250.12 249.05<br>248.08 247.11 246.06 245.06 243.11 241.08 239.07 238.16 237.05<br>236.09 235.07 234.12 233.09 232.05 231.08 225.09 224.08 223.07<br>222.06 221.06 219.08 217.06 213.09 212.08 211.07 210.06 209.06<br>208.05 207.08 206.07 205.06 204.05 198.08 197.13 196.06 195.08<br>194.04 193.06 192.05 191.05 190.04 185.13 184.06 183.05 182.1<br>181.1 180.07 179.08 178.05 177.07 176.03 175.05 171.11 170.1<br>168.03 167.08 166.07 165.07 164.07 163.09 159.11 158.1 157.1<br>156.09 155.08 154.09 153.08 152.07 151.09 150.02 149.07 148.08<br>147.11 146.1 145.1 144.09 143.08 142.11 141.1 140.07 139.09<br>138.05 137.03 136.08 134.06 132.1 131.12 130.09 129.08 128.07<br>127.09 126.05 125.11 124.08 123.07 122.06 121.05 120.04 119.12<br>117.1 116.03 115.03 114.09 113.06 112.08 111.04 110.06 109.08<br>108.08 105.03
	Svenson, J.; Smallfield, B.M.; Joyce, N.I.; Sanson, C.E.; Perry, N.B. Betalains in red and yellow varieties of the Andean tuber crop ulluco (*Ullucus tuberosus). J. Agric. Food Chem.* **2008**, 56, 7730–7737.<br>Slimen, I.B.; Najar, T.; Abderrabba, M. Chemical and Antioxidant Properties of Betalains. *J. Agric. Food Chem.* **2017**, 65, 675–689.<br>Esteves, L.C.; Machado, C.O.; Gonçalves, L.C.P.; Cavalcante, V.F.; Obeid, G.; Correra, T.C.; Bastos, E.L. Structural Effects on the Antioxidant Properties of Amino Acid Betaxanthins. *Antioxidants* **2022**, 11, 2259.

Polar uncharged-type

Glutamine-bx

**Chemical structure**	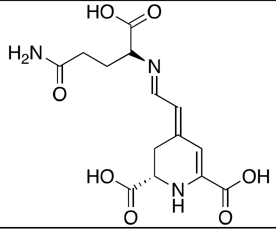
**Name:**	Glutamine-bx (Vulgaxanthin I)
**Chemical Formula**	$C_{14}H_{17}N_3O_7$
**Molecular weight**	339.30
**Monoisotopic mass**	339.1066
***m/z* [M+H]**	340.1139
**Theorical fragments (*m/z*)**	340.11 339.11 338.1 337.09 336.08 325.1 324.12 323.09 322.1 321.07<br>320.09 319.08 318.07 313.1 312.12 311.11 310.1 309.07 308.12<br>307.09 306.11 305.08 304.09 303.06 302.08 300.12 299.09 298.1<br>297.07 296.12 295.09 294.11 293.1 292.09 291.06 290.11 289.08<br>288.1 287.07 286.08 285.07 284.12 283.12 282.11 281.08 280.13<br>279.1 278.11 277.08 276.1 275.09 274.08 273.09 272.1 271.07<br>270.11 269.08 268.09 267.06 266.11 265.07 264.13 263.07 262.12<br>261.09 260.1 259.07 258.09 255.1 254.11 253.08 252.1 251.07<br>250.08 249.05 248.08 247.07 246.12 245.06 244.11 243.08 241.08<br>239.07 238.08 237.05 236.1 235.07 234.09 233.06 232.05 231.08<br>225.09 224.08 223.07 222.06 221.06 220.07 219.08 218.07 217.06<br>213.09 212.08 211.07 210.06 209.06 208.05 207.08 206.07 205.06<br>204.05 198.08 197.09 196.06 195.08 194.04 193.06 192.05 191.05<br>190.04 185.09 184.06 183.05 182.07 181.06 180.07 179.08 178.05<br>177.07 176.03 175.05 171.08 170.07 168.03 167.08 166.07 165.07<br>164.03 163.05 159.08 158.07 157.06 156.05 155.05 154.07 153.04<br>152.07 151.06 150.02 149.07 148.04 147.08 146.07 145.06 144.05<br>143.05 142.07 141.07 140.03 139.05 138.05 137.07 136.08 134.06<br>132.07 131.08 130.05 129.04 128.03 127.05 126.02 125.07 124.04<br>123.06 122.02 121.05 120.04 119.08 117.07 116.03 115.03 114.05<br>113.02 112.04 111.01 110.02 109.08 108.04 105.03 103.09 102.05<br>101.07 100.04
	Piattelli, M.; Minale, L.; Nicolaus, R.A. Pigments of centrospermae—V. *Phytochemistry* **1965**, 4, 817–823.<br>Stintzing, F.C.; Schieber, A.; Carle, R. Identification of betalains from yellow beet (*Beta vulgaris* L.) and cactus pear [*Opuntia ficus-indica* (L.) Mill.] by high-performance liquid chromatography–electrospray ionization mass spectroscopy. *J. Agric. Food Chem.* **2002**, 50, 2302–2307.<br>Slimen, I.B.; Najar, T.; Abderrabba, M. Chemical and Antioxidant Properties of Betalains. *J. Agric. Food Chem.* **2017**, 65, 675–689.<br>Esteves, L.C.; Machado, C.O.; Gonçalves, L.C.P.; Cavalcante, V.F.; Obeid, G.; Correra, T.C.; Bastos, E.L. Structural Effects on the Antioxidant Properties of Amino Acid Betaxanthins. *Antioxidants* **2022**, 11, 2259.

Asparagine-bx

**Chemical structure**	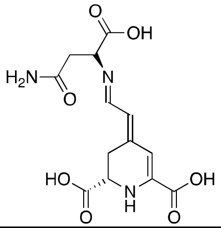
**Name:**	Asparagine-bx (Vulgaxanthin III)
**Chemical Formula**	$C_{13}H_{15}N_3O_7$
**Molecular weight**	325.28
**Monoisotopic mass**	325.0910
***m/z* [M+H]**	326.0983
**Theorical fragments (*m/z*)**	326.1 325.09 324.08 323.07 311.09 310.1 309.07 308.09 307.06 306.07<br>305.06 299.09 298.1 297.1 296.09 295.06 294.11 293.08 292.09<br>291.06 290.08 289.05 288.06 286.1 285.07 284.09 283.06 282.11<br>281.08 280.09 279.08 278.08 277.08 276.1 275.07 274.08 273.05<br>272.07 270.11 269.1 268.09 267.06 266.11 265.08 264.1 263.07<br>262.08 261.09 260.07 259.07 258.09 257.06 256.09 255.04 254.08<br>253.08 252.1 251.07 250.12 249.05 248.1 247.07 246.09 245.06<br>244.07 243.04 241.08 240.1 239.07 238.08 237.05 236.07 235.07<br>234.12 233.06 232.11 231.04 230.09 229.06 225.09 224.1 223.07<br>222.09 221.06 220.07 219.04 218.04 217.06 213.09 212.08 211.07<br>210.06 209.06 208.05 207.08 206.07 205.06 204.05 197.09 196.06<br>195.08 194.04 193.06 192.05 191.05 190.04 184.06 183.05 182.04<br>181.06 180.07 179.08 178.05 177.07 176.03 175.05 171.08 170.04<br>169.06 168.05 167.05 166.07 165.07 164.03 163.05 158.04 157.06<br>156.05 154.05 153.07 152.07 151.09 150.02 149.07 145.06 144.05<br>143.05 142.05 141.03 140.06 139.03 138.05 137.03 136.08 135.06<br>134.02 133.06 132.05 131.05 130.04 129.07 128.06 127.05 126.02<br>125.03 124.04 123.07 122.02 121.05 120.04 118.05 117.07 116.03<br>115.03 114.02 113.03 112.06 111.06 110.02 109.04 108.01 105.07
**Reference**	Kugler, F.; Stintzing, F.C.; Carle, R. Identification of betalains from petioles of differently colored Swiss chard (*Beta vulgaris* L. ssp. cicla Alef. Cv. Bright Lights) by high-performance liquid chromatography-electrospray ionization mass spectrometry. *J. Agric. Food Chem.* **2004**, 52, 2975–2981.<br>Hempel, J.; Bohm, H. Betaxanthin pattern of hairy roots from *Beta vulgaris* var lutea and its alteration by feeding of amino acids. *Phytochemistry* **1997**, 44, 847–852.<br>Slimen, I.B.; Najar, T.; Abderrabba, M. Chemical and Antioxidant Properties of Betalains. *J. Agric. Food Chem.* **2017**, 65, 675–689.<br>Esteves, L.C.; Machado, C.O.; Gonçalves, L.C.P.; Cavalcante, V.F.; Obeid, G.; Correra, T.C.; Bastos, E.L. Structural Effects on the Antioxidant Properties of Amino Acid Betaxanthins. *Antioxidants* **2022**, 11, 2259.

Threonine-bx

**Chemical structure**	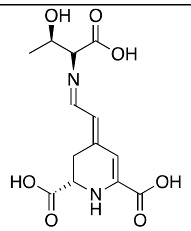
**Name:**	Threonine-bx
**Chemical Formula**	$C_{13}H_{16}N_2O_7$
**Molecular weight**	312.28
**Monoisotopic mass**	312.0958
***m/z* [M+H]**	313.1030
**Theorical fragments (*m/z*)**	313.1 312.1 311.09 310.08 298.08 297.07 296.1 295.09 294.08 293.08<br>292.07 287.09 285.11 284.1 283.09 282.08 281.11 280.11 279.1<br>278.09 277.08 276.07 275.07 273.11 271.09 270.08 269.08 268.07<br>267.06 266.09 265.08 264.07 263.1 262.07 261.09 260.08 259.07<br>258.06 257.11 256.11 255.1 254.09 253.08 252.11 251.07 250.09<br>249.09 248.08 247.07 245.06 243.1 242.09 241.08 240.09 239.07<br>238.09 237.05 236.08 235.07 234.1 233.06 232.08 231.08 230.07<br>229.06 228.09 227.1 226.07 225.09 224.08 223.07 222.06 221.06<br>219.08 217.06 215.08 213.09 212.08 211.07 210.06 209.06 208.05<br>207.08 206.07 205.06 204.05 198.08 197.09 196.06 195.08 194.04<br>193.06 192.05 191.05 190.04 184.06 183.05 182.04 181.06 180.07<br>179.08 178.05 177.07 176.03 175.05 170.04 169.07 168.03 167.08<br>166.07 165.07 164.03 163.05 158.04 157.04 156.07 155.06 154.05<br>153.07 152.07 151.06 150.02 149.07 147.06 144.07 143.06 142.05<br>141.07 140.03 139.05 138.05 137.05 136.04 134.06 132.07 131.06<br>130.05 129.04 128.03 126.05 125.05 124.04 123.04 121.05 120.07<br>119.06 118.05 117.04 116.03 115.06 114.05 113.05 112.04 111.03<br>110.06 108.04 105.05 104.07 103.04 102.03 101.02 100.04
	Kugler, F.; Graneis, S.; Stintzing, F.C.; Carle, R. Studies on betaxanthin profiles of vegetables and fruits from the Chenopodi-aceae and Cactaceae. *Z. Fur. Naturforschung Sect. C J. Biosci.* **2007**, 62, 311–318.<br>Slimen, I.B.; Najar, T.; Abderrabba, M. Chemical and Antioxidant Properties of Betalains. *J. Agric. Food Chem.* **2017**, 65, 675–689.<br>Esteves, L.C.; Machado, C.O.; Gonçalves, L.C.P.; Cavalcante, V.F.; Obeid, G.; Correra, T.C.; Bastos, E.L. Structural Effects on the Antioxidant Properties of Amino Acid Betaxanthins. *Antioxidants* **2022**, 11, 2259.

Serine-bx

**Chemical structure**	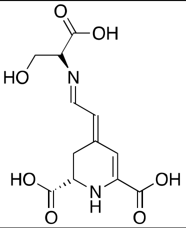
**Name:**	Serine-bx
**Chemical Formula**	$C_{12}H_{14}N_2O_7$
**Molecular weight**	298.25
**Monoisotopic mass**	298.0801
***m/z* [M+H]**	299.0874
**Theorical fragments (*m/z*)**	299.09 298.08 297.07 296.06 295.06 284.08 283.09 282.08 281.08 280.07<br>279.06 277.05 271.09 270.08 269.08 268.07 267.06 266.09 265.08<br>264.07 263.07 262.06 261.05 259.09 257.08 256.08 255.1 254.09<br>253.08 252.07 251.07 250.06 249.05 248.06 247.07 246.06 245.06<br>243.1 242.09 241.08 240.07 239.07 238.09 237.09 236.08 235.07<br>234.06 233.06 232.05 231.04 229.08 228.07 227.07 226.07 225.09<br>224.08 223.07 222.06 221.09 219.08 217.06 214.07 213.09 212.08<br>211.07 210.06 209.06 208.05 207.08 206.07 205.06 204.05 198.08<br>197.09 196.06 195.08 194.04 193.06 192.05 191.05 190.04 184.06<br>183.05 182.04 181.06 180.07 179.08 178.05 177.07 176.03 175.05<br>174.04 170.04 169.05 168.03 167.08 166.07 165.07 164.03 163.05<br>158.04 157.04 156.07 155.06 154.05 153.07 152.07 151.06 150.02<br>149.07 147.06 144.07 142.05 141.04 140.03 139.05 138.05 137.03<br>136.08 134.06 132.03 130.05 129.04 128.03 126.05 125.05 124.04<br>123.04 122.02 121.05 120.04 118.05 117.04 116.03 115.03 114.02<br>113.05 112.04 111.03 110.06 108.04 107.07 106.05 105.04 104.03<br>103.03 102.02 101.05 100.04
**Reference**	Stintzing, F.C.; Schieber, A.; Carle, R. Identification of betalains from yellow beet (*Beta vulgaris* L.) and cactus pear [*Opuntia ficus-indica* (L.) Mill.] by high-performance liquid chromatography–electrospray ionization mass spectroscopy. *J. Agric. Food Chem.* **2002**, 50, 2302–2307.<br>Slimen, I.B.; Najar, T.; Abderrabba, M. Chemical and Antioxidant Properties of Betalains. *J. Agric. Food Chem.* **2017**, 65, 675–689.<br>Esteves, L.C.; Machado, C.O.; Gonçalves, L.C.P.; Cavalcante, V.F.; Obeid, G.; Correra, T.C.; Bastos, E.L. Structural Effects on the Antioxidant Properties of Amino Acid Betaxanthins. *Antioxidants* **2022**, 11, 2259.

Negatively charged-type

Aspartic acid-bx

Chemical structure	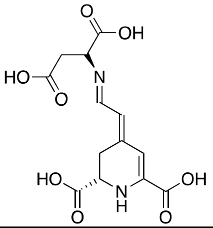
**Name:**	Aspartic acid-bx (Miraxanthin II)
**Chemical Formula**	$C_{13}H_{14}N_2O_8$
**Molecular weight**	326.26
**Monoisotopic mass**	326.0750
***m/z* [M+H]**	327.0823
**Theorical fragments (*m/z*)**	327.08 326.07 325.07 324.06 312.07 311.09 310.08 309.07 308.06 307.06<br>306.05 299.09 298.08 297.07 296.06 295.09 294.08 293.08 292.07<br>291.06 290.05 289.05 287.09 285.07 284.08 283.09 282.08 281.08<br>280.07 279.06 278.05 277.08 276.07 275.07 274.06 273.05 272.04<br>271.09 270.08 269.08 268.07 267.06 266.09 265.08 264.07 263.07<br>262.06 261.05 259.07 257.08 256.07 255.06 254.07 253.08 252.07<br>251.07 250.06 249.05 248.08 247.07 246.06 245.06 244.05 243.04<br>242.07 241.08 240.05 239.07 238.07 237.05 236.06 235.07 234.06<br>233.06 232.05 231.04 229.06 226.07 225.09 224.08 223.07 222.06<br>221.06 220.06 219.04 218.04 217.06 213.09 212.08 211.07 210.06<br>209.06 208.05 207.08 206.07 205.06 204.05 197.09 196.06 195.08<br>194.04 193.06 192.05 191.05 190.04 184.06 183.05 182.04 181.06<br>180.07 179.08 178.05 177.07 176.03 175.05 172.06 170.04 169.04<br>168.03 167.08 166.07 165.07 164.03 163.05 158.04 157.04 156.03<br>154.05 153.07 152.03 151.09 150.02 149.07 146.04 145.04 144.03<br>143.02 142.01 141.04 140.03 139.03 138.05 137.03 136.04 134.04<br>133.04 132.03 131.02 130.05 129.04 128.03 127.03 126.02 125.05<br>124.04 123.07 122.02 121.05 120.04 119.03 118.05 117.02 116.01<br>115 114.02 113.05 112.04 111.03 110.02 109.02 108.01 106.05<br>105.03
**Reference**	Piattelli, M.; Minale, L.; Nicolaus, R.A. Pigments of centrospermae—V. *Phytochemistry* **1965**, 4, 817–823.<br>Gandía-Herrero, F.; García-Carmona, F.; Escribano, J. Development of a protocol for the semisynthesis and purification of betaxanthins. *Phytochem. Anal.* **2006**, 17, 262–269.<br>Slimen, I.B.; Najar, T.; Abderrabba, M. Chemical and Antioxidant Properties of Betalains. *J. Agric. Food Chem.* **2017**, 65, 675–689.<br>Esteves, L.C.; Machado, C.O.; Gonçalves, L.C.P.; Cavalcante, V.F.; Obeid, G.; Correra, T.C.; Bastos, E.L. Structural Effects on the Antioxidant Properties of Amino Acid Betaxanthins. *Antioxidants* **2022**, 11, 2259.

Glutamic acid-bx

Chemical structure	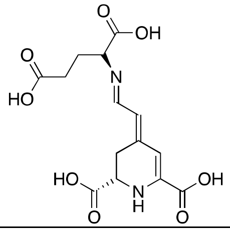
**Name:**	Glutamic acid-bx (Vulgaxanthin II)
**Chemical Formula**	$C_{14}H_{16}N_{2}O_{8}$
**Molecular weight**	340.29
**Monoisotopic mass**	340.0907
***m/z* [M+H]**	341.0979
**Theorical fragments (*m/z*)**	341.1 340.09 339.08 338.07 326.09 325.1 324.1 323.09 322.08 321.07<br>320.06 319.06 313.1 312.1 311.09 310.08 309.11 308.06 307.09<br>306.08 305.08 304.07 303.06 301.1 299.09 298.09 297.11 296.1<br>295.09 294.08 293.08 292.07 291.06 290.09 289.08 288.07 287.07<br>286.06 285.11 284.1 283.09 282.08 281.08 280.07 279.1 278.09<br>277.08 276.07 275.07 273.09 271.09 270.08 269.08 268.07 267.06<br>266.09 265.08 264.07 263.07 262.06 261.09 260.08 259.07 256.08<br>255.1 254.07 253.08 252.05 251.07 250.06 249.05 248.08 247.07<br>246.06 245.06 243.08 241.08 239.07 238.07 237.05 236.08 235.07<br>234.06 233.06 232.05 231.08 225.09 224.08 223.07 222.06 221.06<br>220.08 219.08 218.07 217.06 213.09 212.08 211.07 210.06 209.06<br>208.05 207.08 206.07 205.06 204.05 198.08 197.09 196.06 195.08<br>194.04 193.06 192.05 191.05 190.04 186.08 184.06 183.05 182.04<br>181.06 180.07 179.08 178.05 177.07 176.03 175.05 172.06 171.05<br>170.04 168.03 167.08 166.05 165.07 164.03 163.05 160.06 159.05<br>158.04 157.04 156.03 155.06 154.05 153.04 152.07 151.09 150.05<br>149.07 148.06 147.05 146.04 145.04 144.03 143.06 142.05 141.04<br>140.03 139.05 138.05 137.03 136.08 134.06 133.05 132.07 131.03<br>130.03 129.02 128.03 127 126.05 125.05 124.04 123.03 122.02<br>121.05 120.07 118.05 117.05 116.03 115.04 114.05 113.02 112.02<br>111.01 110.06 108.04 104.07
**Reference**	Piattelli, M.; Minale, L.; Nicolaus, R.A. Pigments of centrospermae—V. *Phytochemistry* **1965**, 4, 817–823.<br>Kugler, F.; Stintzing, F.C.; Carle, R. Identification of betalains from petioles of differently colored Swiss chard (*Beta vulgaris* L. ssp. cicla Alef. Cv. Bright Lights) by high-performance liquid chromatography-electrospray ionization mass spectrometry. *J. Agric. Food Chem.* **2004**, 52, 2975–2981.<br>Slimen, I.B.; Najar, T.; Abderrabba, M. Chemical and Antioxidant Properties of Betalains. *J. Agric. Food Chem.* **2017**, 65, 675–689.<br>Esteves, L.C.; Machado, C.O.; Gonçalves, L.C.P.; Cavalcante, V.F.; Obeid, G.; Correra, T.C.; Bastos, E.L. Structural Effects on the Antioxidant Properties of Amino Acid Betaxanthins. *Antioxidants* **2022**, 11, 2259.

Hydrophobic-type

Alanine-bx

**Chemical structure**	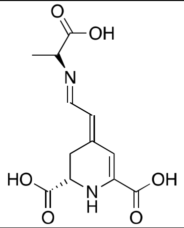
**Name:**	Alanine-bx
**Chemical Formula**	$C_{12}H_{14}N_2O_6$
**Molecular weight**	282.25
**Monoisotopic mass**	282.0852
***m/z* [M+H]**	283.0925
**Theorical fragments (*m/z*)**	283.09 282.08 281.08 280.07 279.06 268.08 267.06 266.09 265.08 264.07<br>263.07 255.1 254.09 253.08 252.07 251.1 250.06 249.09 248.08<br>247.07 246.06 245.06 243.1 241.08 240.09 239.1 238.09 237.09<br>236.08 235.07 234.08 233.09 232.06 231.08 229.06 227.1 226.09<br>225.09 224.08 223.11 222.1 221.09 220.08 219.08 218.07 217.06<br>213.09 212.08 211.07 210.06 209.06 208.05 207.11 206.07 205.1<br>204.05 203.08 201.07 198.08 197.09 196.06 195.08 194.04 193.06<br>192.05 191.05 190.04 184.06 183.05 182.04 181.06 180.07 179.08<br>178.05 177.07 176.03 175.05 174.05 170.04 169.05 168.03 167.08<br>166.07 165.07 164.03 163.05 161.07 158.04 157.04 156.03 154.05<br>153.07 152.07 151.06 150.02 149.07 148.08 147.06 144.03 142.05<br>141.07 140.07 139.05 138.05 137.03 136.08 134.06 132.03 130.05<br>129.04 128.03 126.05 125.05 124.04 123.04 122.06 121.05 120.04<br>116.03 115.03 114.05 113.05 112.04 111.03 110.06 109.05 108.04<br>107.04 106.03 105.03 102.02 101.05 100.04
**References**	Kugler, F.; Stintzing, F.C.; Carle, R. Identification of betalains from petioles of differently colored Swiss chard (*Beta vulgaris* L. ssp. cicla Alef. Cv. Bright Lights) by high-performance liquid chromatography-electrospray ionization mass spectrometry. *J. Agric. Food Chem.* **2004**, 52, 2975–2981.<br>Piattelli, M.; Minale, L.; Nicolaus, R.A. Pigments of centrospermae—V. *Phytochemistry* **1965**, 4, 817–823.<br>Slimen, I.B.; Najar, T.; Abderrabba, M. Chemical and Antioxidant Properties of Betalains. *J. Agric. Food Chem.* **2017**, 65, 675–689.<br>Esteves, L.C.; Machado, C.O.; Gonçalves, L.C.P.; Cavalcante, V.F.; Obeid, G.; Correra, T.C.; Bastos, E.L. Structural Effects on the Antioxidant Properties of Amino Acid Betaxanthins. *Antioxidants* **2022**, 11, 2259.

Valine-bx

**Chemical structure**	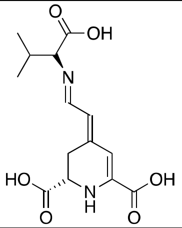
**Name:**	Valine-bx
**Chemical Formula**	$C_{14}H_{18}N_2O_6$
**Molecular weight**	310.31
**Monoisotopic mass**	310.1165
***m/z* [M+H]**	311.1238
**Theorical fragments (*m/z*)**	311.12 310.12 309.11 308.1 297.11 296.1 295.13 294.12 293.11 292.11<br>291.1 290.09 289.08 283.13 282.12 281.11 280.11 279.13 278.09<br>277.12 276.11 275.1 274.09 273.09 271.13 270.08 269.08 268.07<br>267.06 266.13 265.12 264.11 263.1 262.11 261.12 260.09 259.11<br>257.09 255.13 254.13 253.12 252.11 251.07 250.13 249.12 248.12<br>247.11 246.1 245.09 241.12 240.11 239.1 238.11 237.09 236.12<br>235.14 234.1 233.13 232.05 231.11 230.07 229.1 226.11 225.09<br>224.08 223.11 222.06 221.09 220.1 219.08 217.06 213.09 212.08<br>211.07 210.06 209.06 208.05 207.08 206.07 205.06 204.05 203.12<br>202.09 198.08 197.09 196.06 195.08 194.04 193.06 192.05 191.05<br>190.04 184.06 183.05 182.04 181.06 180.07 179.08 178.05 177.07<br>176.03 175.05 174.04 170.04 169.05 168.1 167.08 166.07 165.07<br>164.03 163.05 158.04 157.04 156.1 154.05 153.08 152.07 151.06<br>150.09 149.07 148.08 147.06 144.03 142.09 141.08 140.03 139.05<br>138.05 137.08 136.08 135.07 134.06 132.03 130.05 129.08 128.07<br>127.06 126.05 125.05 124.08 123.04 122.08 121.05 120.04 118.09<br>117.08 116.07 115.06 114.05 113.08 112.08 111.07 110.06 108.04<br>107.07 106.07 105.03 103.08 102.02 101.06 100.05
**References**	Stintzing, F.C.; Schieber, A.; Carle, R. Identification of betalains from yellow beet (*Beta vulgaris* L.) and cactus pear [*Opuntia ficus-indica* (L.) Mill.] by high-performance liquid chromatography–electrospray ionization mass spectroscopy. *J. Agric. Food Chem.* **2002**, 50, 2302–2307.<br>Slimen, I.B.; Najar, T.; Abderrabba, M. Chemical and Antioxidant Properties of Betalains. *J. Agric. Food Chem.* **2017**, 65, 675–689.<br>Esteves, L.C.; Machado, C.O.; Gonçalves, L.C.P.; Cavalcante, V.F.; Obeid, G.; Correra, T.C.; Bastos, E.L. Structural Effects on the Antioxidant Properties of Amino Acid Betaxanthins. *Antioxidants* **2022**, 11, 2259.

Isoleucine-bx

**Chemical structure**	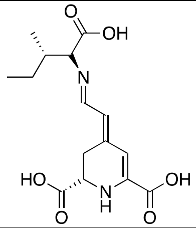
**Name:**	Isoleucine-betaxanthin (Isovulgaxanthin IV)
**Chemical Formula**	$C_{15}H_{20}N_2O_6$
**Molecular weight**	324.33
**Monoisotopic mass**	324.1321
***m/z* [M+H]**	325.1394
**Theorical fragments (*m/z*)**	325.14 324.13 323.12 322.12 311.12 310.12 309.14 308.14 307.13 306.12<br>305.11 304.11 303.1 297.14 296.14 295.13 294.12 293.15 292.11<br>291.13 290.13 289.12 288.11 287.1 285.14 283.13 282.08 281.15<br>280.14 279.13 278.13 277.12 276.11 275.14 274.09 273.12 271.11<br>270.08 269.08 268.07 267.06 266.13 265.15 264.15 263.14 262.13<br>261.12 260.12 259.11 257.09 255.13 254.13 253.12 252.12 251.07<br>250.06 249.05 248.08 247.14 246.06 245.13 244.08 243.11 241.08<br>240.12 239.07 238.11 237.12 236.13 235.11 234.11 233.06 232.05<br>231.04 225.09 224.08 223.07 222.06 221.09 220.1 219.08 218.12<br>217.06 216.1 213.09 212.08 211.07 210.06 209.06 208.05 207.08<br>206.07 205.06 204.05 198.08 197.09 196.06 195.08 194.04 193.06<br>192.05 191.05 190.04 184.06 183.05 182.12 181.06 180.07 179.08<br>178.05 177.07 176.03 175.05 170.04 168.03 167.08 166.09 165.07<br>164.11 163.05 162.09 158.04 157.04 156.1 155.09 154.05 153.07<br>152.07 151.09 150.09 149.07 148.08 147.06 144.1 143.09 142.09<br>141.08 140.07 139.05 138.09 137.03 136.08 134.06 132.1 131.09<br>130.09 129.08 128.03 127.1 126.09 125.08 124.08 123.07 121.05<br>120.04 117.08 116.03 115.08 114.07 113.06 112.08 111.07 110.1<br>108.04 105.03 104.11 102.05 101.05 100.04
**References**	Stintzing, F.C.; Schieber, A.; Carle, R. Identification of betalains from yellow beet (*Beta vulgaris* L.) and cactus pear [*Opuntia ficus-indica* (L.) Mill.] by high-performance liquid chromatography–electrospray ionization mass spectroscopy. *J. Agric. Food Chem.* **2002**, 50, 2302–2307.<br>Slimen, I.B.; Najar, T.; Abderrabba, M. Chemical and Antioxidant Properties of Betalains. *J. Agric. Food Chem.* **2017**, 65, 675–689.<br>Esteves, L.C.; Machado, C.O.; Gonçalves, L.C.P.; Cavalcante, V.F.; Obeid, G.; Correra, T.C.; Bastos, E.L. Structural Effects on the Antioxidant Properties of Amino Acid Betaxanthins. *Antioxidants* **2022**, 11, 2259.

Leucine-bx

**Chemical structure**	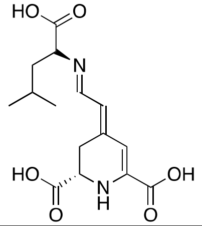
**Name:**	Leucine-bx (Vulgaxanthin IV)
**Chemical Formula**	$C_{15}H_{20}N_2O_6$
**Molecular weight**	324.33
**Monoisotopic mass**	324.1321
***m/z* [M+H]**	325.1394
**Theorical fragments (*m/z*)**	325.14 324.13 323.12 322.12 310.13 309.14 308.14 307.13 306.12 305.11<br>304.11 303.1 297.14 296.14 295.13 294.12 293.15 292.11 291.13<br>290.13 289.12 288.11 287.1 285.14 283.09 282.08 281.15 280.14<br>279.13 278.13 277.12 276.11 275.14 274.09 273.12 271.11 270.08<br>269.08 268.07 267.06 266.13 265.15 264.15 263.14 262.13 261.12<br>260.12 259.11 257.09 255.13 254.13 253.12 252.12 251.07 250.06<br>249.05 248.12 247.14 246.06 245.13 243.11 241.08 240.12 239.07<br>238.11 237.12 236.13 235.11 234.11 233.06 232.05 231.04 225.09<br>224.08 223.07 222.06 221.06 220.1 219.11 218.12 217.06 216.1<br>213.09 212.08 211.07 210.06 209.06 208.05 207.08 206.07 205.06<br>204.05 198.08 197.09 196.06 195.08 194.04 193.06 192.05 191.05<br>190.04 184.06 183.05 182.12 181.06 180.07 179.08 178.05 177.07<br>176.03 175.05 174.04 170.04 169.05 168.03 167.08 166.09 165.07<br>164.11 163.05 162.09 158.04 157.04 156.1 155.09 154.05 153.07<br>152.07 151.09 150.09 149.07 148.08 147.06 144.1 143.09 142.09<br>141.08 140.07 139.05 138.09 137.03 136.08 134.06 132.1 131.09<br>130.09 129.08 128.03 127.1 126.09 125.08 124.08 123.07 122.08<br>121.05 120.04 117.08 116.03 115.08 114.07 113.06 112.08 111.07<br>110.1 108.04 107.07 106.07 105.03 104.11 102.02 101.06 100
**References**	Kugler, F.; Stintzing, F.C.; Carle, R. Identification of betalains from petioles of differently colored Swiss chard (*Beta vulgaris* L. ssp. cicla Alef. Cv. Bright Lights) by high-performance liquid chromatography-electrospray ionization mass spectrometry. *J. Agric. Food Chem.* **2004**, 52, 2975–2981.<br>Hempel, J.; Bohm, H. Betaxanthin pattern of hairy roots from *Beta vulgaris* var lutea and its alteration by feeding of amino acids. *Phytochemistry* **1997**, 44, 847–852.<br>Slimen, I.B.; Najar, T.; Abderrabba, M. Chemical and Antioxidant Properties of Betalains. *J. Agric. Food Chem.* **2017**, 65, 675–689.<br>Esteves, L.C.; Machado, C.O.; Gonçalves, L.C.P.; Cavalcante, V.F.; Obeid, G.; Correra, T.C.; Bastos, E.L. Structural Effects on the Antioxidant Properties of Amino Acid Betaxanthins. *Antioxidants* **2022**, 11, 2259.

Methionine-bx

**Chemical structure**	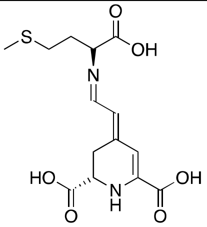
**Name:**	Methionine-bx (Miraxanthin I)
**Chemical Formula**	$C_{14}H_{18}N_2O_6S$
**Molecular weight**	342.37
**Monoisotopic mass**	342.0886
***m/z* [M+H]**	343.0958
**Theorical fragments (*m/z*)**	343.1 342.09 341.08 340.07 328.08 327.1 326.09 325.09 324.08 323.07<br>322.06 321.05 315.1 314.09 313.09 312.08 311.11 310.07 309.09<br>308.08 307.07 306.07 305.06 303.1 301.09 300.09 299.11 298.1<br>297.09 296.08 295.09 294.08 293.08 292.06 291.08 289.06 287.11<br>286.1 285.09 284.08 283.11 282.08 281.1 280.09 279.1 278.09<br>277.08 276.07 275.07 273.09 272.08 271.07 270.08 269.08 268.07<br>267.06 266.05 265.1 264.07 263.07 262.06 261.09 260.08 259.07<br>258.08 257.1 256.06 255.08 254.08 253.06 252.05 251.07 250.09<br>249.09 248.04 247.07 246.06 245.06 243.08 242.08 241.1 240.07<br>239.07 238.05 237.05 236.08 235.07 234.1 233.09 232.08 231.08<br>229.06 227.08 225.09 224.08 223.07 222.06 221.06 219.11 217.1<br>215.08 213.09 212.08 211.07 210.06 209.06 208.05 207.08 206.07<br>205.06 204.05 200.07 199.07 198.08 197.09 196.06 195.08 194.04<br>193.06 192.05 191.05 190.04 188.07 186.06 185.05 184.04 183.05<br>182.04 181.06 180.07 179.08 178.05 177.07 176.03 175.05 174.06<br>173.05 170.04 169.06 168.05 167.08 166.07 165.07 164.03 163.05<br>162.06 161.05 160.04 159.03 158.04 157.04 156.05 154.05 153.07<br>152.07 151.06 150.06 149.07 148.04 147.03 146.03 145.06 144.05<br>143.04 142.03 141.07 140.03 139.05 138.05 137.05 136.04 135.05<br>134.06 133.03 132.02 131.02 130.03 129 128.05 126.05 125.05<br>124.04 123.04 122.06 121.05 120.05 119.02 118.01 117.04 116.03<br>115.02 114.05 113.05 112.04 111.03 110.06 108.04 106.07 105.04<br>104.05 103.02 102.04 101.01 100
**References**	Piattelli, M.; Minale, L.; Nicolaus, R.A. Pigments of centrospermae—V. *Phytochemistry* **1965**, 4, 817–823.<br>Gandía-Herrero, F.; García-Carmona, F.; Escribano, J. Development of a protocol for the semisynthesis and purification of betaxanthins. *Phytochem. Anal.* **2006**, 17, 262–269.<br>Slimen, I.B.; Najar, T.; Abderrabba, M. Chemical and Antioxidant Properties of Betalains. *J. Agric. Food Chem.* **2017**, 65, 675–689.<br>Esteves, L.C.; Machado, C.O.; Gonçalves, L.C.P.; Cavalcante, V.F.; Obeid, G.; Correra, T.C.; Bastos, E.L. Structural Effects on the Antioxidant Properties of Amino Acid Betaxanthins. *Antioxidants* **2022**, 11, 2259.

Phenylalanine-bx

**Chemical structure**	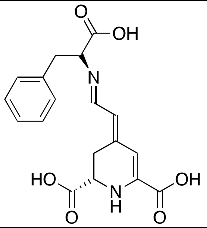
**Name:**	Phenylalanine-bx
**Chemical Formula**	$C_{18}H_{18}N_2O_6$
**Molecular weight**	358.3358.35005
**Monoisotopic mass**	358.1165
***m/z* [M+H]**	359.1238
**Theorical fragments (*m/z*)**	359.12 358.12 357.11 356.1 344.11 343.13 342.12 341.11 340.11 339.1<br>338.09 337.08 331.13 330.12 329.11 328.12 327.13 326.1 325.12<br>324.11 323.1 322.09 321.09 320.08 319.13 317.11 316.12 315.13<br>314.13 313.12 312.11 311.1 310.09 309.12 308.09 307.11 305.09<br>303.13 302.13 301.12 300.11 299.14 298.13 297.12 296.12 295.11<br>294.1 293.09 289.12 288.11 287.1 286.11 285.12 284.12 283.14<br>282.08 281.08 280.07 279.11 277.1 274.11 273.12 272.09 271.11<br>270.11 269.09 268.1 267.06 266.09 265.08 264.07 263.07 262.06<br>261.05 258.11 257.13 256.1 255.15 254.08 253.08 252.07 251.07<br>250.06 249.05 248.08 247.07 246.06 245.06 243.11 242.12 241.1<br>240.1 239.07 238.09 237.09 236.08 235.07 234.06 233.06 232.05<br>231.04 228.1 227.12 225.1 224.08 223.07 222.06 221.09 219.08<br>217.06 216.1 215.09 214.09 213.09 212.08 211.07 210.06 209.06<br>208.05 207.08 206.07 205.06 204.1 202.09 201.08 200.07 199.1<br>198.09 197.09 196.06 195.08 194.04 193.06 192.05 191.05 190.09<br>189.08 185.08 184.08 183.05 182.04 181.06 180.07 179.08 178.05<br>177.08 176.07 175.05 174.05 172.08 170.04 169.05 168.03 167.08<br>166.09 165.07 164.07 163.06 162.05 161.08 160.08 159.07 158.04<br>157.04 156.03 154.05 153.07 152.07 151.09 150.09 149.06 148.05<br>147.04 146.06 144.08 142.05 141.07 140.03 139.05 138.09 137.03<br>136.08 135.07 134.06 133.06 132.06 131.05 130.05 129.03 128.03<br>126.02 125.05 124.04 123.07 122.1 121.06 120.08 119.05 118.07<br>117.06 116.03 115.03 114.05 113.05 112.04 111.03 110.02 109.06<br>108.04 107.05 106.07 105.03 103.05 102.02 101.04 100
**References**	Stintzing, F.C.; Schieber, A.; Carle, R. Identification of betalains from yellow beet (*Beta vulgaris* L.) and cactus pear [*Opuntia ficus-indica* (L.) Mill.] by high-performance liquid chromatography–electrospray ionization mass spectroscopy. *J. Agric. Food Chem.* **2002**, 50, 2302–2307.<br>Slimen, I.B.; Najar, T.; Abderrabba, M. Chemical and Antioxidant Properties of Betalains. *J. Agric. Food Chem.* **2017**, 65, 675–689.<br>Esteves, L.C.; Machado, C.O.; Gonçalves, L.C.P.; Cavalcante, V.F.; Obeid, G.; Correra, T.C.; Bastos, E.L. Structural Effects on the Antioxidant Properties of Amino Acid Betaxanthins. *Antioxidants* **2022**, 11, 2259.

Tyrosine-bx

**Chemical structure**	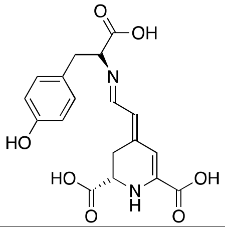
**Name:**	Tyrosine-bx (Portulacaxanthin II)
**Chemical Formula**	$C_{18}H_{18}N_2O_7$
**Molecular weight**	374.35
**Monoisotopic mass**	374.1114
***m/z* [M+H]**	375.1187
**Theorical fragments (*m/z*)**	375.12 374.11 373.1 372.1 360.11 359.12 358.12 357.11 356.1 355.09<br>354.08 353.08 347.12 346.12 345.11 344.11 343.13 342.1 341.11<br>340.11 339.1 338.09 337.08 336.07 335.12 333.11 332.11 331.13<br>330.12 329.11 328.11 327.1 326.09 325.12 324.09 323.1 322.09<br>321.09 320.08 319.13 318.12 317.11 316.11 315.13 314.13 313.12<br>312.11 311.1 310.09 309.09 305.11 304.11 303.1 302.1 301.12<br>300.11 299.14 298.09 297.12 295.11 293.09 290.1 289.12 288.09<br>287.1 286.11 285.09 284.09 283.07 282.08 281.08 280.07 279.06<br>274.11 273.12 272.09 271.14 270.08 269.09 268.07 267.06 266.09<br>265.08 264.07 263.07 262.06 261.05 259.11 258.11 257.09 256.1<br>253.08 252.07 251.07 250.06 249.05 248.08 247.07 246.06 245.06<br>244.1 243.11 241.1 239.07 238.09 237.09 236.08 235.07 234.06<br>233.06 232.1 231.04 230.08 225.09 224.08 223.07 222.06 221.09<br>220.1 219.08 218.08 217.07 216.07 215.05 214.09 213.09 212.08<br>211.07 210.06 209.06 208.05 207.08 206.08 205.07 204.05 201.08<br>200.07 199.06 198.05 197.09 196.06 195.08 194.04 193.07 192.07<br>191.05 190.05 188.07 184.06 183.05 182.08 181.06 180.07 179.06<br>178.05 177.08 176.07 175.06 174.05 170.04 169.05 168.03 167.08<br>166.07 165.05 164.05 163.04 162.05 161.05 160.08 158.04 157.04<br>156.03 154.09 153.07 152.07 151.09 150.02 149.06 148.05 147.04<br>146.04 145.03 144.04 142.05 141.07 140.03 139.05 138.09 137.06<br>136.08 135.04 134.06 133.05 132.03 131.05 130.05 129.04 128.03<br>126.02 125.06 124.04 123.04 122.08 121.03 120.04 119.05 118.04<br>117.03 116.03 115.03 114.05 113.05 112.04 111.04 110.02 108.04<br>107.05 106.04 105.03
**References**	Trezzini, G.F.; Zrÿd, J.-P. Two betalains from *Portulaca grandiflora*. *Phytochemistry* **1991**, 30, 1897–1899.<br>Kugler, F.; Stintzing, F.C.; Carle, R. Identification of betalains from petioles of differently colored Swiss chard (*Beta vulgaris* L. ssp. cicla Alef. Cv. Bright Lights) by high-performance liquid chromatography-electrospray ionization mass spectrometry. *J. Agric. Food Chem.* **2004**, 52, 2975–2981.<br>Slimen, I.B.; Najar, T.; Abderrabba, M. Chemical and Antioxidant Properties of Betalains. *J. Agric. Food Chem.* **2017**, 65, 675–689.<br>Esteves, L.C.; Machado, C.O.; Gonçalves, L.C.P.; Cavalcante, V.F.; Obeid, G.; Correra, T.C.; Bastos, E.L. Structural Effects on the Antioxidant Properties of Amino Acid Betaxanthins. *Antioxidants* **2022**, 11, 2259.

Tryptophan-bx

**Chemical structure**	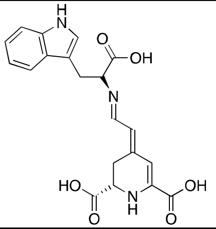
**Name:**	Tryptophan-bx
**Chemical Formula**	$C_{20}H_{19}N_3O_6$
**Molecular weight**	397.39
**Monoisotopic mass**	397.1274
***m/z* [M+H]**	398.1347
**Theorical fragments (*m/z*)**	398.13 397.13 396.12 395.11 383.12 382.14 381.13 380.12 379.12 378.11<br>377.1 371.12 370.14 369.13 368.12 367.13 366.14 365.11 364.13<br>363.12 362.11 361.11 360.1 358.14 356.12 355.13 354.14 353.14<br>352.13 351.12 350.11 349.12 348.13 347.1 346.12 344.1 343.13<br>342.14 341.14 340.13 339.12 338.15 337.14 336.13 335.13 334.12<br>333.09 332.1 328.13 327.12 326.11 325.12 324.13 323.13 322.16<br>321.1 320.14 319.08 318.12 316.11 313.12 312.13 311.1 310.12<br>309.12 308.1 307.11 306.09 305.1 304.09 303.08 302.08 297.12<br>296.14 295.11 294.16 293.09 292.11 291.11 290.13 289.1 287.09<br>285.07 282.08 281.08 280.07 279.06 268.07 267.06 266.09 265.08<br>264.07 263.07 262.06 261.05 255.11 254.1 253.08 252.07 251.07<br>250.06 249.05 248.08 247.07 246.06 245.06 243.11 241.1 240.09<br>239.07 238.09 237.09 236.09 235.07 234.06 233.06 232.05 231.04<br>229.1 228.09 225.09 224.08 223.07 222.06 221.07 219.08 217.1<br>216.09 215.08 214.07 213.09 212.08 211.07 210.06 209.06 208.05<br>207.08 206.07 205.1 204.09 203.08 202.07 201.1 200.09 199.09<br>198.08 197.07 196.06 195.08 194.04 193.06 192.05 191.05 190.04<br>189.1 188.07 187.06 186.05 185.07 184.06 183.05 182.04 181.06<br>180.07 179.08 178.05 177.1 176.03 175.05 174.05 173.11 172.08<br>171.07 170.06 169.08 168.04 167.08 166.07 165.07 164.03 163.05<br>161.11 160.08 159.09 158.04 157.08 156.07 154.05 153.07 152.07<br>151.09 150.02 149.07 148.08 147.06 146.06 144.08 143.07 142.07<br>141.07 140.05 139.05 138.05 137.03 136.08 134.06 132.08 131.07<br>130.07 129.04 128.03 126.02 125.05 124.04 123.07 122.08 121.05<br>120.04 118.07 117.06 116.05 115.03 114.05 113.05 112.04 111.03<br>110.02 108.04 107.07 106.07 105.06 104.05 103.05 102.02 100
**References**	Cai, Y.-Z.; Sun, M.; Corke, H. Characterization and application of betalain pigments from plants of the Amaranthaceae. *Trends Food Sci. Technol.* **2005**, 16, 370–376.<br>Slimen, I.B.; Najar, T.; Abderrabba, M. Chemical and Antioxidant Properties of Betalains. *J. Agric. Food Chem.* **2017**, 65, 675–689.<br>Esteves, L.C.; Machado, C.O.; Gonçalves, L.C.P.; Cavalcante, V.F.; Obeid, G.; Correra, T.C.; Bastos, E.L. Structural Effects on the Antioxidant Properties of Amino Acid Betaxanthins. *Antioxidants* **2022**, 11, 2259.

Glycine-bx

**Chemical structure**	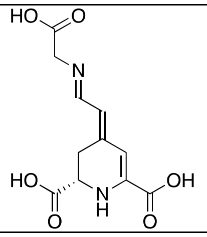
**Name:**	Glycine-bx (Portulacaxanthin III)
**Chemical Formula**	$C_{11}H_{12}N_2O_6$
**Molecular weight**	268.22
**Monoisotopic mass**	268.0695
***m/z* [M+H]**	269.0768
**Theorical fragments (*m/z*)**	269.08 268.07 267.06 266.05 265.05 254.07 253.08 252.07 251.07 250.06<br>249.05 241.08 240.07 239.07 238.07 237.09 236.06 235.07 234.06<br>233.06 232.05 231.04 229.08 227.07 226.07 225.09 224.08 223.07<br>222.06 221.09 220.06 219.08 218.04 217.06 215.05 213.09 212.08<br>211.07 210.06 209.09 208.08 207.08 206.07 205.06 204.05 203.05<br>199.07 198.06 197.06 196.06 195.08 194.04 193.1 192.05 191.08<br>190.04 189.07 187.05 184.06 183.05 182.04 181.06 180.07 179.05<br>178.05 177.03 176.03 175.09 174.04 173.07 170.04 169.04 168.07<br>167.06 166.05 165.04 164.03 163.05 162.05 161.07 160.04 158.04<br>157.04 156.03 154.05 153.07 152.07 151.06 150.05 149.07 148.06<br>147.06 146.05 144.03 142.05 140.03 139.06 138.05 137.07 136.08<br>135.06 134.06 132.03 130.05 129.04 128.03 126.05 125.05 124.04<br>123.04 121.05 116.03 115.03 114.05 113.05 112.04 111.03 110.02<br>109.05 108.04 107.07 106.03 105.03 102.02 100.04
**References**	Trezzini, G.F.; Zrÿd, J.-P. Two betalains from *Portulaca grandiflora*. *Phytochemistry* **1991**, 30, 1897–1899.<br>Kugler, F.; Stintzing, F.C.; Carle, R. Identification of betalains from petioles of differently colored Swiss chard (*Beta vulgaris* L. ssp. cicla Alef. Cv. Bright Lights) by high-performance liquid chromatography-electrospray ionization mass spectrometry. *J. Agric. Food Chem.* **2004**, 52, 2975–2981.<br>Slimen, I.B.; Najar, T.; Abderrabba, M. Chemical and Antioxidant Properties of Betalains. *J. Agric. Food Chem.* **2017**, 65, 675–689.<br>Esteves, L.C.; Machado, C.O.; Gonçalves, L.C.P.; Cavalcante, V.F.; Obeid, G.; Correra, T.C.; Bastos, E.L. Structural Effects on the Antioxidant Properties of Amino Acid Betaxanthins. *Antioxidants* **2022**, 11, 2259.

Cysteine-bx

**Chemical structure**	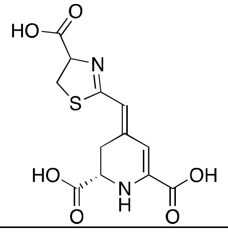
**Name:**	Cysteine-bx
**Chemical Formula**	$C_{12}H_{12}N_2O_6S$
**Molecular weight**	312.30
**Monoisotopic mass**	312.0416
***m/z* [M+H]**	313.0489
**Theorical fragments (*m/z*)**	313.05 312.04 311.03 310.03 309.02 299.03 298.04 297.05 296.05 295.04<br>294.03 293.02 285.05 284.05 283.04 282.04 281.06 280.03 279.04<br>278.04 277.03 276.02 275.01 273.05 271.04 270.04 269.06 268.05<br>267.04 266.04 265.06 264.03 263.05 262.02 261.03 259.02 257.06<br>256.05 255.04 254.04 253.06 252.06 251.05 250.04 249.03 248.04<br>247.02 243.04 242.04 241.03 240.03 239.05 238.04 237.07 236.03<br>235.05 234.06 233.04 231.04 228.03 227.05 226.02 225.03 224.04<br>223.02 222.02 221 219.08 217.06 215.05 213.03 212.04 211.05<br>210.02 209.06 208.01 207.02 206.03 205.04 204.01 199.05 197.04<br>196.04 195.02 194.04 193.06 191.05 189.07 184.06 183.05 182.04<br>181.04 180.05 179.03 178.03 170.03 169.02 168.01 167.06 166.05<br>165.04 164.03 163.05 158.04 157.04 156.01 154.03 153.02 152.02<br>151.01 149.07 147.06 144.01 142.05 140.03 139.06 138.05 137.03<br>136.04 132.01 131 130 128.99 127.98 126 124.04 123.07<br>121.05 116.03 115.03 114 113.05 111.99 111.03 109.97 105
**References**	Esteves, L.C.; Machado, C.O.; Gonçalves, L.C.P.; Cavalcante, V.F.; Obeid, G.; Correra, T.C.; Bastos, E.L. Structural Effects on the Antioxidant Properties of Amino Acid Betaxanthins. *Antioxidants* **2022**, 11, 2259.

Proline-bx

**Chemical structure**	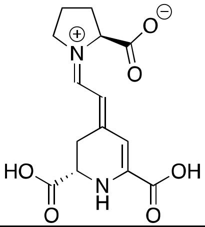
**Name:**	Proline-bx (Indicaxanthin)
**Chemical Formula**	$C_{14}H_{16}N_2O_6$
**Molecular weight**	308.29
**Monoisotopic mass**	308.1008
***m/z* [M+H]**	309.1081
**Theorical fragments (*m/z*)**	309.11 308.1 307.09 306.08 305.08 297.11 295.09 294.08 293.11 292.11<br>291.1 290.09 289.08 287.07 283.09 282.08 281.08 280.07 279.1<br>278.09 277.12 276.11 275.1 274.09 273.09 272.08 271.07 269.08<br>268.07 267.06 266.05 265.08 264.07 263.07 262.06 261.09 259.11<br>257.09 255.1 254.09 253.08 252.07 251.07 250.06 249.09 248.12<br>247.07 246.1 245.06 244.08 243.08 241.08 239.07 238.09 237.09<br>236.08 235.07 234.1 233.06 232.08 231.04 229.06 225.09 224.08<br>223.07 222.06 221.09 220.08 219.08 218.07 217.06 213.09 211.07<br>210.06 209.06 208.05 207.08 206.07 205.06 204.13 203.12 202.11<br>201.1 200.07 197.07 196.06 195.08 194.04 193.06 192.05 191.05<br>190.04 182.04 180.07 179.08 178.05 177.1 176.03 175.09 170.04<br>168.03 167.08 166.07 165.07 164.06 163.05 162.04 161.07 154.09<br>152.07 151.09 150.05 149.07 140.07 138.09 136.08 127.06 126.05<br>125.05 124.08 122.06 116.07 115.06 114.05 113.05 112.04 111.07<br>110.06 109.05 108.04 101.06 100.05
**References**	Impellizzeri, G.; Piattelli, M. Biosynthesis of indicaxanthin in *Opuntia ficus-indica* fruits. *Phytochemistry* **1972**, 11, 2499–2502.<br>Stintzing, F.C.; Schieber, A.; Carle, R. Identification of betalains from yellow beet (*Beta vulgaris* L.) and cactus pear [*Opuntia ficus-indica* (L.) Mill.] by high-performance liquid chromatography–electrospray ionization mass spectroscopy. *J. Agric. Food Chem.* **2002**, 50, 2302–2307.Slimen, I.B.; Najar, T.; Abderrabba, M. Chemical and Antioxidant Properties of Betalains. *J. Agric. Food Chem.* **2017**, 65, 675–689.<br>Esteves, L.C.; Machado, C.O.; Gonçalves, L.C.P.; Cavalcante, V.F.; Obeid, G.; Correra, T.C.; Bastos, E.L. Structural Effects on the Antioxidant Properties of Amino Acid Betaxanthins. *Antioxidants* **2022**, 11, 2259.

Special cases

γ-aminobutyric acid-bx

**Chemical structure**	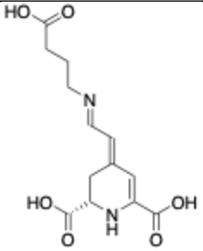
**Name:**	γ-aminobutyric acid-bx
**Chemical Formula**	$C_{13}H_{16}N_2O_6$
**Molecular weight**	296.28
**Monoisotopic mass**	296.1008
***m/z* [M+H]**	297.1081
**Theorical fragments (*m/z*)**	297.11 296.1 295.09 294.08 283.09 282.08 281.11 280.11 279.1 278.09<br>277.08 276.07 275.07 270.08 269.11 268.07 267.06 266.09 265.12<br>264.07 263.1 262.09 261.09 260.08 259.07 257.11 255.1 254.1<br>253.12 252.11 251.1 250.09 249.05 248.08 247.11 246.06 245.09<br>243.08 241.12 240.11 239.07 238.09 237.12 236.12 235.11 234.1<br>233.09 232.08 231.08 227.1 226.09 225.09 224.08 223.07 222.06<br>221.13 220.08 219.11 217.1 215.08 213.09 212.08 211.07 210.06<br>209.06 208.05 207.08 206.07 205.06 204.05 198.08 197.09 196.06<br>195.08 194.04 193.06 192.05 191.05 190.04 189.1 188.07 184.06<br>183.05 182.04 181.06 180.07 179.08 178.05 177.07 176.03 175.05<br>174.04 170.04 169.05 168.03 167.08 166.07 165.07 164.03 163.05<br>158.04 157.04 156.03 154.05 153.07 152.07 151.06 150.02 149.07<br>147.06 144.03 142.05 141.07 140.03 139.05 138.05 137.03 136.08<br>135.07 134.06 132.03 130.05 129.04 128.07 127.06 126.05 125.05<br>124.04 123.04 122.06 121.05 120.04 116.03 115.06 114.05 113.05<br>112.04 111.03 110.06 108.04 107.07 106.07 105.03 104.07 103.06<br>102.05 101.05 100
**References**	Spórna-Kucab, A.; Tekieli, A.; Grzegorczyk, A.; Świątek, Ł.; Rajtar, B.; Skalicka-Woźniak, K.; Starzak, K.; Nemzer, B.; Pietrzkowski, Z.; Wybraniec, S. Metabolite profiling analysis and the correlation with biological activity of betalain-rich *Portulaca grandiflora* Hook. extracts. *Antioxidants* **2022**, 11, 1654.<br>Wybraniec, S.; Nowak-Wydra, B.; Mitka, K.; Kowalski, P.; Mizrahi, Y. Minor betalains in fruits of *Hylocereus* species. *Phytochemistry* **2007**, 68, 251–259.<br>Kugler, F.; Stintzing, F.C.; Carle, R. Identification of betalains from petioles of differently colored Swiss chard (*Beta vulgaris* L. ssp. cicla Alef. Cv. Bright Lights) by high-performance liquid chromatography-electrospray ionization mass spectrometry. *J. Agric. Food Chem.* **2004**, 52, 2975–2981.

Dopamine-bx

**Chemical structure**	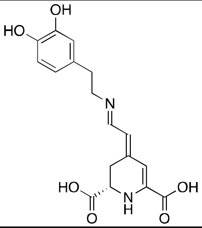
**Name:**	Dopamine-bx (Miraxanthin V)
**Chemical Formula**	$C_{17}H_{18}N_2O_6$
**Molecular weight**	346.34
**Monoisotopic mass**	346.1165
***m/z* [M+H]**	347.1238
**Theorical fragments (*m/z*)**	347.12 346.12 345.11 344.1 343.09 332.11 331.13 330.12 329.11 328.11<br>327.1 326.09 325.08 319.13 318.12 317.11 316.12 315.13 314.1<br>313.12 312.09 311.1 310.09 309.09 307.13 305.11 304.12 303.13<br>302.13 301.12 300.11 299.1 293.11 291.13 290.13 289.12 288.11<br>287.14 286.11 285.12 284.12 283.11 281.09 277.12 276.11 275.1<br>274.11 273.09 272.09 271.11 270.11 262.11 261.1 260.09 259.14<br>258.14 257.13 256.1 246.11 245.13 244.1 243.11 242.08 241.1<br>240.09 237.09 236.08 235.07 231.11 230.12 229.1 228.1 224.08<br>223.07 222.06 221.09 219.08 218.12 216.1 213.09 212.08 211.07<br>210.06 209.06 208.08 207.08 206.07 205.06 204.1 203.09 202.09<br>197.09 196.06 195.08 194.04 193.06 192.1 191.05 190.04 189.08<br>188.07 184.06 183.05 182.04 181.06 180.07 179.08 178.05 177.08<br>176.03 175.05 174.04 170.04 169.05 168.03 167.08 166.07 165.08<br>164.07 163.06 162.05 161.07 158.04 157.04 156.03 154.09 153.07<br>152.07 151.06 150.05 149.05 148.04 147.07 146.05 145.04 144.03<br>143.02 142.05 141.07 140.03 139.05 138.05 137.06 136.08 135.04<br>134.06 133.05 132.03 130.05 129.04 128.03 126.05 125.05 124.04<br>123.04 122.04 121.05 120.04 119.05 118.04 116.03 115.03 114.05<br>113.05 112.04 111.04 110.06 109.03 108.02 107.05 106.04 105.03<br>102.02 100
**References**	Piattelli, M.; Minale, L.; Nicolaus, R.A. Pigments of centrospermae—V. *Phytochemistry* **1965**, 4, 817–823.<br>Kobayashi, N.; Schmidt, J.; Wray, V.; Schliemann, W. Formation and occurrence of dopamine derived betacyanins. *Phytochemistry* **2001**, 56, 429–436.<br>Slimen, I.B.; Najar, T.; Abderrabba, M. Chemical and Antioxidant Properties of Betalains. *J. Agric. Food Chem.* **2017**, 65, 675–689.<br>Esteves, L.C.; Machado, C.O.; Gonçalves, L.C.P.; Cavalcante, V.F.; Obeid, G.; Correra, T.C.; Bastos, E.L. Structural Effects on the Antioxidant Properties of Amino Acid Betaxanthins. *Antioxidants* **2022**, 11, 2259.

L-DOPA-bx

Chemical structure	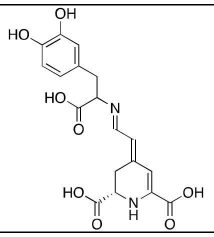
**Name:**	L-DOPA-bx (Dopaxanthin)
**Chemical Formula**	$C_{18}H_{18}N_2O_8$
**Molecular weight**	390.35
**Monoisotopic mass**	390.1063
***m/z* [M+H]**	391.1136
**Theorical fragments (*m/z*)**	391.11 390.11 389.1 388.09 376.1 375.12 374.11 373.1 372.1 371.09<br>370.08 363.12 362.11 361.1 360.11 359.12 358.09 357.11 356.1<br>355.09 354.08 353.08 352.07 351.12 349.1 348.11 347.12 346.12<br>345.11 344.1 343.09 342.08 341.11 340.08 339.1 338.09 337.08<br>336.07 335.12 334.12 333.11 332.1 331.13 330.12 329.11 328.11<br>327.1 326.09 325.08 321.11 320.1 319.09 318.1 317.11 316.11<br>315.13 314.09 313.12 311.1 309.09 306.1 305.11 304.08 303.1<br>302.1 301.08 300.09 299.07 290.1 289.12 288.09 287.14 286.07<br>285.09 284.09 283.11 282.08 281.08 280.07 279.06 275.1 274.11<br>273.09 272.09 269.08 268.07 267.06 266.09 265.08 264.07 263.07<br>262.06 261.05 260.09 259.11 257.09 253.08 252.07 251.07 250.06<br>249.05 248.09 247.07 246.08 245.06 239.07 238.09 237.09 236.09<br>235.07 234.08 233.07 232.06 231.04 230.08 229.07 228.07 225.09<br>224.08 223.07 222.08 221.07 219.08 217.07 216.07 215.06 214.05<br>213.09 212.08 211.07 210.06 209.07 208.06 207.08 206.04 205.06<br>204.07 198.08 197.07 196.06 195.05 194.04 193.06 192.07 191.06<br>190.05 184.06 183.05 182.04 181.05 180.07 179.03 178.05 177.07<br>176.07 175.05 174.04 170.04 169.05 168.07 167.08 166.07 165.07<br>164.05 163.04 162.03 161.02 160.04 158.04 157.04 156.03 154.09<br>153.05 152.07 151.04 150.05 149.05 148.04 147.04 146.04 144.03<br>142.05 141.05 140.03 139.05 138.05 137.02 136.08 135.04 134.04<br>133.03 130.05 129.04 128.03 127.04 126.02 125.05 124.04 123.04<br>122.04 121.05 120.04 119.05 118.04 116.03 115.03 114.05 113.05<br>112.04 111.04 110.02 109.03 108.02 107.05 106.04 105.03
**References**	Gandía-Herrero, F.; Escribano, J.; García-Carmona, F. Betaxanthins as substrates for tyrosinase. An approach to the role of tyrosinase in the biosynthetic pathway of betalains. *Plant Physiol.* **2005**, 138, 421–432.<br>Slimen, I.B.; Najar, T.; Abderrabba, M. Chemical and Antioxidant Properties of Betalains. *J. Agric. Food Chem.* **2017**, 65, 675–689.<br>Esteves, L.C.; Machado, C.O.; Gonçalves, L.C.P.; Cavalcante, V.F.; Obeid, G.; Correra, T.C.; Bastos, E.L. Structural Effects on the Antioxidant Properties of Amino Acid Betaxanthins. *Antioxidants* **2022**, 11, 2259.

Tyramine-bx

**Chemical structure**	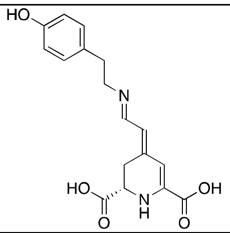
**Name:**	Tyramine-bx (Miraxanthin III)
**Chemical Formula**	$C_{17}H_{18}N_2O_5$
**Molecular weight**	330.34
**Monoisotopic mass**	330.1216
***m/z* [M+H]**	331.1288
**Theorical fragments (*m/z*)**	331.13 330.12 329.11 328.11 327.1 316.12 315.13 314.13 313.12 312.11<br>311.1 310.09 309.09 303.13 302.13 301.12 300.12 299.14 298.11<br>297.12 296.09 295.11 294.1 293.09 291.13 289.12 288.12 287.14<br>286.13 285.12 284.12 283.11 277.12 275.14 274.13 273.12 272.12<br>271.14 270.11 269.13 268.12 267.11 265.1 261.12 260.12 259.11<br>258.11 257.09 256.1 255.11 254.12 253.1 246.11 245.1 244.1<br>243.15 242.14 241.13 240.1 239.12 237.09 236.08 235.07 232.13<br>230.12 229.13 228.1 227.12 226.09 225.1 224.08 223.07 222.06<br>221.09 219.08 215.12 214.12 213.09 212.08 211.07 210.06 209.06<br>208.08 207.08 206.07 205.06 204.05 203.05 202.12 200.11 197.09<br>196.06 195.08 194.04 193.06 192.05 191.05 190.04 188.11 187.1<br>186.09 184.06 183.05 182.04 181.06 180.07 179.08 178.05 177.07<br>176.11 175.05 174.09 173.08 172.08 170.04 169.05 168.03 167.08<br>166.07 165.07 164.03 163.05 162.09 161.08 160.08 158.04 157.04<br>156.03 154.05 153.07 152.07 151.09 150.09 149.08 148.08 147.07<br>146.06 145.04 144.03 143.02 142.05 141.07 140.03 139.05 138.09<br>137.03 136.08 135.07 134.06 133.05 132.04 131.07 130.05 129.04<br>128.03 126.02 125.05 124.04 123.07 122.08 121.06 120.04 119.05<br>118.04 117.06 116.03 115.03 114.05 113.05 112.04 111.03 110.02<br>109.03 108.04 107.05 106.04 105.03 104.06 102.02 100
**References**	Piattelli, M.; Minale, L.; Nicolaus, R.A. Pigments of centrospermae—V. *Phytochemistry* **1965**, 4, 817–823.<br>Schliemann, W.; Cai, Y.; Degenkold, T.; Schmidt, J.; Corke, H. Betalains of *Celosia argentea*. *Phytochemistry* **2001**, 58, 159–165.<br>Slimen, I.B.; Najar, T.; Abderrabba, M. Chemical and Antioxidant Properties of Betalains. *J. Agric. Food Chem.* **2017**, 65, 675–689.<br>Esteves, L.C.; Machado, C.O.; Gonçalves, L.C.P.; Cavalcante, V.F.; Obeid, G.; Correra, T.C.; Bastos, E.L. Structural Effects on the Antioxidant Properties of Amino Acid Betaxanthins. *Antioxidants* **2022**, 11, 2259.

3-methoxy-tyramine-bx

**Chemical structure**	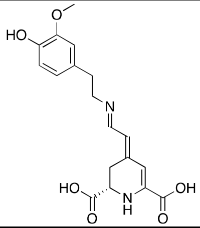
**Name:**	3-methoxy-tyramine-bx
**Chemical Formula**	$C_{18}H_{20}N_2O_6$
**Molecular weight**	360.37
**Monoisotopic mass**	360.1321
***m/z* [M+H]**	361.1394
**Theorical fragments (*m/z*)**	361.14 360.13 359.12 358.12 357.11 346.13 345.14 344.14 343.13 342.12<br>341.11 340.11 339.1 333.14 332.14 331.13 330.12 329.11 328.11<br>327.13 326.1 325.12 324.11 323.1 321.14 319.13 318.13 317.15<br>316.14 315.13 314.13 313.12 312.11 311.1 309.09 307.13 305.15<br>304.14 303.13 302.13 301.15 300.12 299.14 298.13 297.12 295.11<br>291.13 290.13 289.12 288.12 287.1 286.11 285.12 284.13 283.11<br>276.12 275.12 274.11 273.16 272.15 271.14 270.11 260.13 259.14<br>258.11 257.13 256.1 255.11 254.1 245.13 244.13 243.11 242.12<br>237.09 236.08 235.07 232.13 230.12 224.08 223.07 222.06 221.09<br>219.08 218.12 217.11 216.1 213.09 212.08 211.07 210.06 209.06<br>208.08 207.08 206.12 205.06 204.1 203.09 202.09 197.09 196.06<br>195.08 194.04 193.06 192.1 191.09 190.04 184.06 183.05 182.04<br>181.06 180.07 179.09 178.09 177.07 176.07 175.05 174.04 170.04<br>169.05 168.1 167.08 166.09 165.08 164.07 163.06 162.05 161.08<br>158.04 157.04 156.03 154.05 153.07 152.07 151.08 150.02 149.06<br>148.05 147.07 146.05 145.04 144.03 143.02 142.05 141.07 140.03<br>139.05 138.05 137.06 136.05 135.09 134.06 133.06 132.06 130.05<br>129.04 128.03 126.02 125.06 124.04 123.04 122.04 121.06 120.06<br>119.05 116.03 115.03 114.05 113.05 112.04 111.03 110.02 109.06<br>108.02 107.05 106.04 105.07 103.05 102.02 100
**References**	Piattelli, M.; Minale, L.; Nicolaus, R.A. Pigments of centrospermae—V. *Phytochemistry* **1965**, 4, 817–823.<br>Schliemann, W.; Cai, Y.; Degenkold, T.; Schmidt, J.; Corke, H. Betalains of *Celosia argentea*. *Phytochemistry* **2001**, 58, 159–165.<br>Belhadj Slimen, I.; Najar, T.; Abderrabba, M. Chemical and Antioxidant Properties of Betalain. *J. Agric. Food Chem.* **2017**, 65, 675–689, Correction in **2017**, 65, 1466.

5-hydroxynorvaline-bx

**Chemical structure**	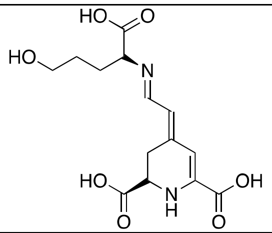
**Name:**	5-hydroxynorvaline-bx
**Chemical Formula**	$C_{14}H_{18}N_2O_7$
**Molecular weight**	326.30
**Monoisotopic mass**	326.1114
***m/z* [M+H]**	327.1187
**Theorical fragments (*m/z*)**	327.12 326.11 325.1 324.1 312.11 311.12 310.12 309.11 308.1 307.09<br>306.08 305.08 299.12 298.12 297.11 296.1 295.13 294.08 293.11<br>292.11 291.1 290.09 289.08 287.12 285.11 284.11 283.13 282.12<br>281.11 280.11 279.1 278.09 277.12 276.11 275.1 274.09 273.09<br>272.08 271.13 270.12 269.08 268.07 267.06 266.13 265.12 264.11<br>263.1 262.09 261.09 260.08 259.11 257.11 256.11 255.1 254.1<br>253.08 252.05 251.07 250.06 249.05 248.12 247.11 246.1 245.09<br>243.08 242.1 241.08 240.09 239.07 238.11 237.09 236.09 235.07<br>234.06 233.06 232.05 231.11 229.1 226.11 225.09 224.08 223.07<br>222.06 221.09 220.1 219.08 218.08 217.06 213.09 212.08 211.07<br>210.06 209.06 208.05 207.08 206.07 205.06 204.05 198.08 197.09<br>196.06 195.08 194.04 193.06 192.05 191.05 190.04 184.1 183.05<br>182.04 181.06 180.07 179.08 178.05 177.07 176.03 175.05 172.1<br>170.04 169.07 168.07 167.08 166.07 165.07 164.03 163.05 158.04<br>157.07 156.03 154.05 153.07 152.07 151.09 150.05 149.07 146.08<br>145.07 144.07 143.06 142.05 141.07 140.03 139.06 138.05 137.03<br>136.08 134.08 133.07 132.07 131.06 130.05 129.08 128.07 127.06<br>126.05 125.05 124.08 123.07 122.06 121.05 120.04 119.07 118.09<br>117.05 116.05 115.04 114.05 113.05 112.08 111.07 110.06 109.05<br>108.04 106.09 105.03 104.07 103.06 102.02 101.06 100
**References**	Strack, D., Schmitt, D., Reznik, H., Boland, W., Grotjahn, L. and Wray, V. Humilixanthin a new betaxanthin from *Rivina humilis*. *Phytochemistry* 1987, 26, 2285-2287.<br>Belhadj Slimen, I.; Najar, T.; Abderrabba, M. Chemical and Antioxidant Properties of Betalain. *J. Agric. Food Chem.* **2017**, 65, 675–689, Correction in **2017**, 65, 1466.

Methionine sulfoxide-bx

**Chemical structure**	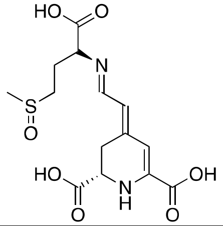
**Name:**	Methionine sulfoxide-bx (Miraxanthin I)
**Chemical Formula**	$C_{14}H_{18}N_2O_7S$
**Molecular weight**	358.37
**Monoisotopic mass**	358.0835
***m/z* [M+H]**	359.0907
**Theorical fragments (*m/z*)**	359.09 358.08 357.08 356.07 344.08 343.1 342.09 341.08 340.07 339.06<br>338.06 337.05 331.1 330.09 329.08 328.07 327.1 326.07 325.09<br>324.08 323.07 322.06 321.05 319.1 317.08 316.08 315.1 314.09<br>313.09 312.08 311.07 310.06 309.09 308.06 307.07 305.06 303.1<br>302.09 301.09 300.08 299.11 298.1 297.09 296.08 295.09 294.08<br>293.08 289.09 288.08 287.07 286.07 285.09 284.08 283.11 282.08<br>281.08 280.07 279.1 278.09 277.08 276.07 275.07 274.07 273.09<br>272.06 271.07 270.08 269.08 268.07 267.06 266.09 265.08 264.07<br>263.07 262.06 261.09 259.07 258.08 257.1 256.06 255.1 254.05<br>253.08 252.05 251.07 250.06 249.05 248.08 247.07 246.06 245.06<br>243.08 242.08 241.08 240.07 239.07 238.09 237.05 236.08 235.07<br>234.06 233.06 232.05 231.08 229.06 228.07 227.08 225.09 224.08<br>223.07 222.06 221.06 219.08 217.06 216.07 215.06 214.05 213.09<br>212.08 211.07 210.06 209.06 208.05 207.08 206.07 205.06 204.07<br>202.05 201.05 200.04 199.07 198.08 197.09 196.06 195.08 194.04<br>193.06 192.05 191.05 190.05 189.05 185.05 184.04 183.05 182.04<br>181.06 180.07 179.08 178.05 177.05 176.04 175.05 174.02 172.04<br>170.04 169.05 168.03 167.08 166.05 165.07 164.04 163.03 162.02<br>161.05 160.04 159.03 158.04 157.04 156.03 154.05 153.07 152.07<br>151.09 150.06 149.03 148.02 147.01 146.03 145 144.05 142.05<br>141.07 140.03 139.05 138.06 137.03 136.04 135.01 134.06 133.03<br>132.02 131.02 130.05 129 128.03 126.02 125.05 124.04 123.07<br>122.06 121.03 120.05 119.02 118.03 117 116.03 115.03 114.05<br>113.05 112.04 111.03 110.02 109.03 108.04 107.02 106.03 105.04<br>104.03 103.02 102.05 101.01 100
**References**	Gandía-Herrero, F.; García-Carmona, F.; Escribano, J. Development of a protocol for the semisynthesis and purification of betaxanthins. *Phytochem. Anal.* **2006**, 17, 262–269.Piattelli, M.; Minale, L.; Nicolaus, R.A. Pigments of centrospermae—V. *Phytochemistry* **1965**, 4, 817–823. Belhadj Slimen, I.; Najar, T.; Abderrabba, M. Chemical and Antioxidant Properties of Betalain. *J. Agric. Food Chem.* **2017**, 65, 675–689, Correction in **2017**, 65, 1466.

Table S2. List of ion Fragments obtained in the Mass profile fingerprint of betalains and observed in HCD at 50 eV (Ions Relative Abundance > 5%)

Signal	*m/z*	Chemical structure of fragments
1	389.09	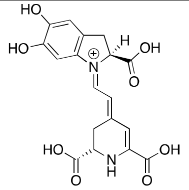
2	387.07	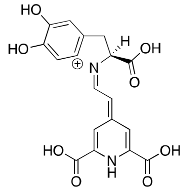
3	345.10	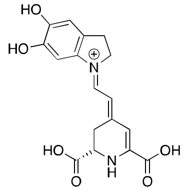
4	343.09	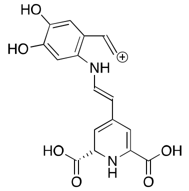
5	341.07	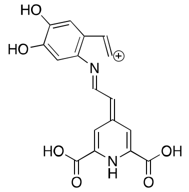
6	313.08	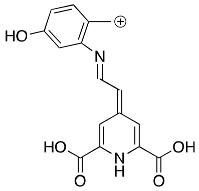
7	299.10	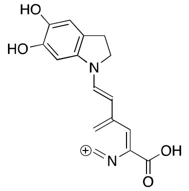
8	297.08	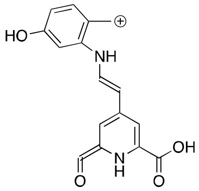

9	281.09	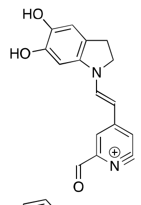
10	269.09	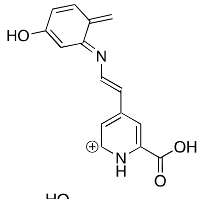
11	255.11	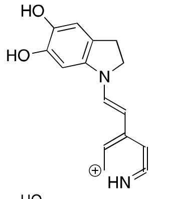
12	255.08	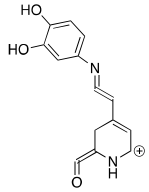
13	253.09	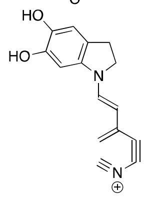
14	211.07	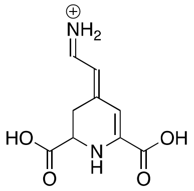
15	194.04	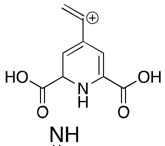
16	179.08	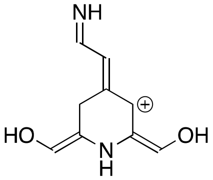
17	178.05	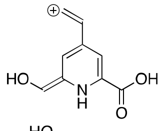
18	176.07	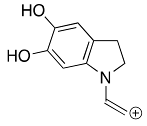

19	166.05	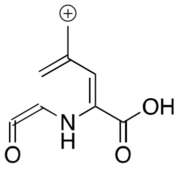
20	150.05	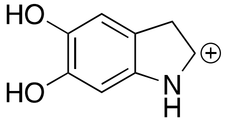 or 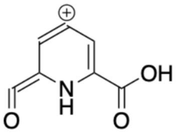
21	138.05	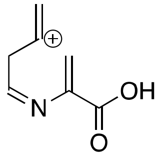
22	132.04	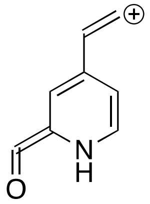
23	106.06	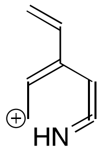

Table S3. Chromatographic and HRMS data for betalains from *Beta vulgaris* extract

#	Compound	Retention time (Rt)	Relative Rt from betanin	Chemical Formula	theorical m/z $[M+H]^+$	Observed m/z $[M+H]^+$	mass accuracy (ppm)	Fragments
1	Betalamic acid	3.23	0.94	$C_9H_9NO_5$	212.0553	212.0545	-3.77	194.04, 166.05, 148.04, 138.05, 120.04, 106.03
**Betanin-type**								
**2**	Prebetanin	3.19	0.93	$C_{24}H_{26}N_2O_{16}S$	631.1076	631.1052	-3.8	389.09, 345.10, 343.09, 299.10, 297.08, 281.09, 269.09, 255.11, 253.09, 194.04, 166.05, 178.05, 176.07, 166.05, 150.05, 138.05, 132.04, 106.6
**3**	Betanin	3.43	1	$C_{24}H_{26}N_2O_{13}$	551.1508	551.1483	-4.54	389.09, 345.10, 343.09, 299.10, 297.08, 281.09, 269.09, 255.11, 253.09, 194.04, 166.05, 178.05, 176.07, 166.05, 150.05, 138.05, 132.04, 106.6
**4**	Betanidin	3.44	1	$C_{18}H_{16}N_2O_8$	389.0979	389.0964	-3.86	389.09, 345.10, 343.09, 299.10, 297.08, 281.09, 269.09, 255.11, 253.09, 194.04, 166.05, 178.05, 176.07, 166.05, 150.05, 138.05, 132.04, 106.6
**5**	2-decarboxy-2,3-dehydro-betanin (2-decarboxy-xanbetanin)	3.62	1.06	$C_{23}H_{24}N_2O_{11}$	505.1453	505.1429	-4.75	343.09, 299.10, 297.08, 281.09, 269.09, 255.08, 253.09, 194.04, 178.05, 176.07, 150.05, 138.05, 132.04, 106.06
**6**	17-decarboxy-betanin	5.83	1.7	$C_{23}H_{26}N_2O_{11}$	507.1609	507.1591	-3.55	345.10, 299.10, 297.08, 281.09, 255.11, 253.09, 194.04, 178.05, 176.07, 150.05, 138.05, 132.04, 106.06
**7**	Isoprebetanin	6.01	1.75	$C_{24}H_{26}N_2O_{16}S$	631.1076	631.1047	-4.6	389.09, 345.10, 343.09, 299.10, 297.08, 281.09, 269.09, 255.11, 253.09, 194.04, 166.05, 178.05, 176.07, 166.05, 150.05, 138.05, 132.04, 106.6
**8**	Isobetanin	6.59	1.92	$C_{24}H_{26}N_2O_{13}$	551.1508	551.1481	-4.9	389.09, 345.10, 343.09, 299.10, 297.08, 281.09, 269.09, 255.11, 253.09, 194.04, 166.05, 178.05, 176.07, 166.05, 150.05, 138.05, 132.04, 106.6
**9**	2-decarboxy-2,3-dehydro-isobetanin (2-decarboxy-isoxanbetanin)	6.6	1.92	$C_{23}H_{24}N_2O_{11}$	505.1453	505.1433	-3.96	343.09, 299.10, 297.08, 281.09, 269.09, 255.08, 253.09, 194.04, 178.05, 176.07, 150.05, 138.05, 132.04, 106.06
**10**	2,17-bidecarboxy-2,3-dehydro-betanin (2,17-bidecarboxy-xanbetanin)	7.61	2.22	$C_{22}H_{24}N_2O_9$	461.1555	461.1534	-4.55	343.09, 299.10, 297.08, 281.09, 269.09, 255.08, 253.09, 194.04, 178.05, 176.07, 150.05, 138.05, 132.04, 106.06
**11**	17-decarboxy-isobetanin	7.69	2.24	$C_{23}H_{26}N_2O_{11}$	507.1609	507.1587	-4.34	345.10, 299.10, 297.08, 281.09, 255.11, 253.09, 194.04, 178.05, 176.07, 150.05, 138.05, 132.04, 106.06
**12**	Phyllocactin	7.71	2.25	$C_{27}H_{28}N_2O_{16}$	637.1512	637.1497	-2.35	389.09, 345.10, 343.09, 299.10, 297.08, 281.09, 269.09, 255.11, 253.09, 194.04, 166.05, 178.05, 176.07, 166.05, 150.05, 138.05, 132.04, 106.6
**13**	17-decarboxy-phyllocactin	8.01	2.34	$C_{26}H_{28}N_2O_{14}$	593.1613	593.1591	-3.71	345.10, 299.10, 297.08, 281.09, 255.11, 253.09, 194.04, 178.05, 176.07, 150.05, 138.05, 132.04, 106.06
**14**	15-decarboxy-betanin	8.29	2.42	$C_{23}H_{26}N_2O_{11}$	507.1609	507.1591	-3.55	345.10, 299.10, 297.08, 281.09, 255.11, 253.09, 194.04, 178.05, 176.07, 150.05, 138.05, 132.04, 106.06
**15**	Neobetanin	8.31	2.42	$C_{24}H_{24}N_2O_{13}$	549.1351	549.1336	-2.73	387.07, 341.07, 313.08, 299.10, 299.06, 287.08, 281.09, 269.09, 255.08, 253.09, 194.04, 178.05, 176.07, 166.05, 150.05, 132.04, 106.06
**16**	17-decarboxy-neobetanin	8.39	2.45	$C_{23}H_{24}N_2O_{11}$	505.1453	505.1438	-2.97	343.09, 299.10, 297.08, 281.09, 269.09, 255.08, 253.09, 194.04, 178.05, 176.07, 150.05, 138.05, 132.04, 106.06

**17**	Isophyllocactin	8.46	2.47	$C_{27}H_{28}N_2O_{16}$	637.1512	637.1491	-3.3	389.09, 345.10, 343.09, 299.10, 297.08, 281.09, 269.09, 255.11, 253.09, 194.04, 166.05, 178.05, 176.07, 166.05, 150.05, 138.05, 132.04, 106.6
**18**	2-decarboxy-betanin	8.83	2.57	$C_{23}H_{26}N_2O_{11}$	507.1609	507.1596	-2.56	345.10, 299.10, 297.08, 281.09, 255.11, 253.09, 194.04, 178.05, 176.07, 150.05, 138.05, 132.04, 106.06
**19**	17-decarboxy-isophyllocactin	9.21	2.69	$C_{26}H_{28}N_2O_{14}$	593.1613	593.1588	-4.21	345.10, 299.10, 297.08, 281.09, 255.11, 253.09, 194.04, 178.05, 176.07, 150.05, 138.05, 132.04, 106.06
**20**	2-decarboxy-2,3-dehydro-neobetanin	10.06	2.93	$C_{23}H_{22}N_2O_{11}$	503.1296	503.1277	-3.78	341.07, 327.06, 313.08, 295.07, 277.07, 267.07, 253.06, 251.08, 221.07, 195.09, 132.04, 106.06
**21**	2,17-bidecarboxy-2,3-dehydro-neobetanin	11.14	3.25	$C_{22}H_{22}N_2O_9$	459.1398	459.1383	-3.27	297.08, 269.09, 251.08, 223.08, 195.09, 133.08
**22**	Lampranthin II	11.01	3.21	$C_{34}H_{34}N_2O_{16}$	727.1981	727.1956	-3.44	389.09, 345.10, 343.09, 299.10, 297.08, 281.09, 269.09, 255.11, 253.09, 194.04, 166.05, 178.05, 176.07, 166.05, 150.05, 138.05, 132.04, 106.6
**23**	Isolampranthin II	11.34	3.31	$C_{34}H_{34}N_2O_{16}$	727.1981	727.1948	-4.54	389.09, 345.10, 343.09, 299.10, 297.08, 281.09, 269.09, 255.11, 253.09, 194.04, 166.05, 178.05, 176.07, 166.05, 150.05, 138.05, 132.04, 106.6
**Melocactin-type**								
**24**	Melocactin	4.81	1.4	$C_{30}H_{36}N_2O_{18}$	713.2036	713.2005	-4.35	389.09, 345.10, 343.09, 299.10, 297.08, 281.09, 269.09, 255.11, 253.09, 194.04, 166.05, 178.05, 176.07, 166.05, 150.05, 138.05, 132.04, 106.6
**25**	Isomelocactin	7.18	2.09	$C_{30}H_{36}N_2O_{18}$	713.2036	713.2009	-3.79	389.09, 345.10, 343.09, 299.10, 297.08, 281.09, 269.09, 255.11, 253.09, 194.04, 166.05, 178.05, 176.07, 166.05, 150.05, 138.05, 132.04, 106.6
**Apiocactin-type**								
**26**	2'-O-apiosyl-phyllocactin	8.31	2.42	$C_{32}H_{36}N_2O_{20}$	769.1934	769.1912	-2.86	389.09, 345.10, 343.09, 299.10, 297.08, 281.09, 269.09, 255.11, 253.09, 194.04, 166.05, 178.05, 176.07, 166.05, 150.05, 138.05, 132.04, 106.6

Continue Table S3.

#	Compound	Retention time (Rt)	Relative Rt from betanin	Chemical Formula	theorical m/z $[M+H]^+$	Observed m/z $[M+H]^+$	mass accuracy (ppm)	Fragments
**Betaxhanitns**								
**Positively charged-type**								
**27**	Histamine-bx	9.04	2.64	$C_{14}H_{16}N4O_4$	305.1244	305.1232	-3.93	287.12, 261.13, 256.89, 211.07, 194.04, 166.05, 150.05, 138.05, 132.04, 130.05, 106.06
**Polar uncharged-type**								
**28**	Glutamine-bx	1.2	0.35	$C_{14}H_{17}N3O_7$	340.1139	340.1129	-2.94	323.08, 277.08, 249.08, 233.09, 231.07, 211.07, 194.04, 166.05, 150.05, 138.05, 132.04, 130.05, 106.06
**29**	Threonine-bx	1.52	0.44	$C_{13}H_{16}N2O_7$	313.103	313.1022	-2.56	269.09, 267.09, 211.07, 194.04, 166.05, 150.05, 138.05, 132.04, 130.05, 106.06
**30**	Serine-bx	4.77	1.39	$C_{12}H_{14}N2O_7$	299.0874	299.0861	-4.35	255.09, 253.09, 211.07, 194.04, 166.05, 150.05, 138.05, 132.04, 130.05, 106.06
**Hydrophobic-type**								
**31**	Alanine-bx	2.02	0.59	$C_{12}H_{14}N_2O_6$	283.0925	283.0915	-3.53	237.08, 211.07, 194.04, 166.05, 150.05, 138.05, 132.04, 130.05, 106.06
**32**	Proline-bx	3.04	0.89	$C_{14}H_{16}N_2O_6$	309.1081	309.1068	-4.21	265.11, 263.10, 235.1, 219.11, 217.09, 211.07, 194.04, 166.05, 150.05, 138.05, 132.04, 130.05, 106.06
**33**	Glycine-bx	6.49	1.89	$C_{11}H_{12}N_2O_6$	269.0768	269.0758	-3.72	331.12, 239.11, 285.12, 283.10, 239.11, 211.07, 194.04, 166.05, 150.05, 138.05, 132.04, 130.05, 106.06
**34**	Valine-bx	7.97	2.32	$C_{14}H_{18}N_2O_6$	311.1238	311.1229	-2.89	267.13, 265.11, 237.12, 221.12, 219.11, 211.07, 194.04, 166.05, 150.05, 138.05, 132.04, 130.05, 106.06
**35**	Isoleucine-bx	9.87	2.88	$C_{15}H_{20}N_2O_6$	325.1394	325.1384	-3.08	279.13, 251.13, 235.14, 233.12, 211.07, 194.04, 166.05, 150.05, 138.05, 132.04, 130.05, 106.06
**36**	Leucine-bx	10.01	2.92	$C_{15}H_{20}N_2O_6$	325.1394	325.1385	-2.77	281.14, 279.13, 251.13, 235.14, 233.12, 211.07, 194.04, 166.05, 150.05, 138.05, 132.04, 130.05, 106.06
**37**	Tryptophan-bx	10.12	2.95	$C_{20}H_{19}N_3O_6$	398.1347	398.1331	-4.02	269.07, 223.07, 211.07, 194.04, 166.05, 150.05, 138.05, 132.04, 130.05, 106.06
**38**	Phenylalanine-bx	10.15	2.96	$C_{18}H_{18}N_2O_6$	359.1238	359.1225	-3.62	315.13, 313.11, 269.12, 267.11, 223.12, 211.07, 194.04, 166.05, 150.05, 138.05, 132.04, 130.05, 106.06
**Special case**								
**39**	Methionine sulfoxide-bx	10.09	2.94	$C_{14}H_{18}N_2O_7S$	359.0907	359.0892	-4.18	267.11, 223.12, 211.07, 194.04, 166.05, 150.05, 138.05, 132.04, 130.05, 106.06
